# In Silico Evaluation of Quinolone–Triazole and Conazole–Triazole Hybrids as Promising Antimicrobial and Anticancer Agents

**DOI:** 10.3390/ijms26146752

**Published:** 2025-07-14

**Authors:** Humaera Noor Suha, Mansour H. Almatarneh, Raymond A. Poirier, Kabir M. Uddin

**Affiliations:** 1 Department of Biochemistry and Microbiology, North South University, Dhaka 1229, Bangladesh; mohammed.uddin11@northsouth.edu; 2 Department of Chemistry, College of Science, Imam Mohammad Ibn Saud Islamic University (IMSIU), Riyadh 11623, Saudi Arabia; 3 Department of Chemistry, Memorial University, St. John’s, NL A1B 3X7, Canada; rpoirier@mun.ca

**Keywords:** quinolone, conazole, triazole, anticancer, antimicrobial

## Abstract

Cancer remains one of the leading causes of death globally, highlighting the urgent need for novel anticancer therapies with higher efficacy and reduced toxicity. Similarly, the rise in multidrug-resistant pathogens and emerging infectious diseases underscores the critical demand for new antimicrobial agents that target resistant infections through unique mechanisms. This study used computational approaches to investigate twenty quinolone–triazole and conazole–triazole hybrid derivatives as antimicrobial and anticancer agents (**1**–**20**) with nine reference drugs. By studying their interactions with 6 bacterial DNA gyrase and 10 cancer-inducing target proteins (*E. faecalis*, *M. tuberculosis*, *S. aureus*, *E. coli*, *M. smegmatis*, *P. aeruginosa* and EGFR, MPO, VEGFR, CDK6, MMP1, Bcl-2, LSD1, HDAC6, Aromatase, ALOX15) and comparing them with established drugs such as ampicillin, cefatrizine, fluconazole, gemcitabine, itraconazole, ribavirin, rufinamide, streptomycin, and tazobactam, compounds **15** and **16** emerged as noteworthy antimicrobial and anticancer agents, respectively. In molecular dynamics simulations, compounds **15** and **16** had the strongest binding at −10.6 kcal mol^−1^ and −12.0 kcal mol^−1^ with the crucial 5CDQ and 2Z3Y proteins, respectively, exceeded drug-likeness criteria, and displayed extraordinary stability within the enzyme’s pocket over varied temperatures (300–320 K). In addition, we used density functional theory (DFT) to calculate dipole moments and molecular orbital characteristics and analyze the thermodynamic stability of putative antimicrobial and anticancer derivatives. This finding reveals a well-defined, possibly therapeutic relationship, supported by theoretical and future in vitro and in vivo studies. Compounds **15** and **16**, thus, emerged as intriguing contenders in the fight against infectious diseases and cancer.

## 1. Introduction

Cancer is one of the deadliest illnesses in the world, and despite amazing medical progress, it has been shown to be a major cause of death in both developing and industrialized nations [1]. At the top of the list of the most important health-related issues worldwide, cancer is one of the most common illnesses since it is the worst potentially fatal condition [2]. A multitude of anticancer drugs are available. On the other hand, novel prospective drugs with a high therapeutic index must be developed due to drug-induced toxicity, poor selectivity, and tolerance (multidrug resistance) [3,4]. The management of infectious diseases continues to be a significant and difficult challenge due to a number of reasons, such as the rise in multidrug-resistant microbial pathogens and newly emerging infectious diseases [5]. Despite the fact that there are many antibiotics and chemotherapeutics accessible for medical use, the emergence of both old and novel antibiotic resistance in recent decades has shown that there is a significant medical need for new classes of antimicrobial medicines [5]. New antimicrobial compounds with modes of action distinct from well-known antibacterial drug classes, to which many clinically important infections are now resistant, are desperately needed, as is perceived [5].

The triazole moiety has been introduced as a linking scaffold between two or more pharmacophores as a result of contemporary drug discovery research. Triazoles have drawn a lot of attention owing to their intriguing biological and physical characteristics, as well as their incredible stability, which makes them attractive drug core structures [2,6]. Their affinity to effortlessly bind with numerous enzymes and receptors in the biological organization makes them a unique entity among five-membered heterocycles and portrays a wide variety of pharmacological activities [7]. Many researchers have paid close attention to their potent therapeutic properties, such as their antibacterial, antifungal, antiviral, antitubercular, anticancer, anticonvulsant, anti-inflammatory, analgesic, and antidepressant activity [8]. Several triazole-based therapeutic medicines have been extensively explored and commercially used, including antivirals (ribavirin), antifungals (fluconazole and voriconazole), anticancer (anastrozole and letrozole), and antimigraine medications (rizatriptan) [9]. Plant growth regulators paclobutrazol and diniconazole, pesticides such as insecticide triazophos, fungicides triadimefon and prothioconazole, and herbicides sulfentrazone and flupoxam have been widely utilized in agricultural production [10]. Several biologically active natural chemicals include the 2-imino-1,3-thiazoline nucleus, which has strong antibacterial properties against mold, yeast, and bacteria [11]. It is also hypothesized that the presence of Schiff base contributes to antibacterial action because of its capacity to interact with metal ions within the active site of many enzymes, resulting in a complex [12]. Furthermore, it has been demonstrated that the thiazole ring is a fundamental structural component in a variety of natural compounds, including vitamin B1 thiamine, the thiamine pyrophosphate TPP co-enzyme throughout the Krebs cycle, carboxylase, and a large family of macrocyclic thiopeptide antibiotics such as thiostrepton and micrococcin [13]. Similarly, multiple investigations have shown that the benzothiazole nucleus acts as an antibacterial against a variety of species, including *Staphylococcus aureus*, *Escherichia coli*, and *Plasmodium* [14]. A literature review found that there are very few publications on 1,2,3-triazole-tethered 1,4-dihydropyridines. Prasad and colleagues showed that symmetric bis-triazolylated-1,4-dihydropyridines 1 were a strong anti-breast cancer agent [15]. Kumbhare et al. described the synthesis of triazole-aryl groups attached to 1,4-dihydropyridines 2 as angiotensin-converting enzyme (ACE) inhibitors [16]. Aneja and colleagues described the synthesis of 1,2,3-triazole-linked pentasubstituted 1,4-dihydropyridines as antibacterial, antifungal, and antioxidant agents [17]. Vijesh et al. reported that pyrazole-based 1,4-dihydropyridine derivatives are powerful antibacterial and antioxidant agents [18]. Quinoline containing 1,4-dihydropyridine [19] and 1,4-dihydropyridine [20] showed antioxidant action. The C2-symmetric bis-1,2,3-triazole and acridinedione grafted macromolecule [21] and 1,2,3-triazole [22] demonstrated antioxidant activity. Another group of researchers reported [23,24,25] on the synthesis and antioxidant evaluation of aryl-1,2,3-triazole-appended heterocycles. Another study found that aryl-1,2,3-triazole-integrated 1,4-dihydropyridines had strong antioxidant activity [26].

Mycobacterium tuberculosis (Mtb) infection has been linked to an increased incidence of both pulmonary and non-pulmonary malignancies [27,28,29]. Vascular endothelial-derived growth factor (VEGF) acts on VEGFR-2 (represented by Thiazoles I and II, and Oxazole III), a crucial protein involved in the etiology of both cancer and *Mycobacterium tuberculosis* (Mtb) [30,31]. Benzoxazole, benzothiazole, 1,2,4-triazole, and triazolothiadiazole exhibit a variety of pharmacological actions, particularly anticancer and antimycobacterial properties [32,33,34]. Thiazole, oxazole, and triazolothiadiazole scaffolds have shown strong inhibitory efficacy against VEGFR-2 with IC50 = 91 nM, 0.503 μM, and 0.08 μM, respectively [33]. Triazolothiadiazole IV showed substantial antiproliferative action against NCI 60 cell lines, with GI50 values ranging from 0.20 to 2.58 μM [34]. Benzoxazoles V and VI showed promising anti-mycobacterial action, with MIC values of 6.25 μM and 0.24 μM. Significant anti-mycobacterial action was shown for 1,2,4-triazoles VII and VIII, as well as [1,2,4]triazolo[3,4-b][1,3,4]thiadiazole IX, at concentrations of 5.5 μM, 0.19 μM, and 0.5 μM, respectively [35,36,37].

Abnormalities in the cyclin-dependent kinases (CDKs) pathway have been reported in a number of cancers, including breast, lung, melanoma, and gastrointestinal cancers [38,39,40]. The advent of a novel class of highly selective ATP-competitive CDK4 and/or CDK6 inhibitors has recently caught researchers’ interest due to their improved efficacy and low side effects [41,42]. A study found that bis-oxindole and bis-spiro-triazole-oxindole compounds significantly reduced the growth of MCF-7 (IC50 = 2.81–17.61 μM) and MDA-MB-231 (IC50 = 3.23–7.98 μM) breast cancer cell lines, with low inhibitory activity against normal WI-38 cells [43]. The standard doxorubicin had IC50 scores of 7.43 μM against MCF-7 and 5.71 μM against MDA-MB-231 cells. Furthermore, some of the synthesized compounds showed considerable anti-CDK4 activity (IC50 = 0.157–0.618 μM) compared to palbociclib (IC50 = 0.071 μM) [43].

Aromatase is a cytochrome P450 enzyme that catalyzes the conversion of androgens into estrogens [44,45]. Breast cancer cells have been found to have elevated estrogen levels. Aromatase inhibitors are one of the most successful current therapy methods for estrogen-dependent breast cancer. As a result, aromatase inhibitors (AIs) are one of the most extensively utilized medication groups for the treatment of estrogen-dependent cancer [44,45,46,47]. Aromatase inhibitors that have been used clinically can be classified as first-, second-, and third-generation, depending on their evolutionary period, or as steroidal or non-steroidal aromatase inhibitors (NSAIs) based on their structural similarities to steroids [48,49]. In an earlier work, 3-[4-(5-methyl-1H-benzo[d]imidazol-2-yl)phenyl]-6-(substituted phenyl)-7H-[1,2,4]triazolo[3,4-b][1,3,4]thiadiazines derivatives were produced and shown substantial aromatase inhibition (IC50 = 0.037 ± 0.001 μM). Interestingly, one of the compounds, with a difluoro substituent at positions 2 and 4 of the phenyl ring, exhibited the most powerful aromatase inhibitory activity without causing considerable damage to a normal healthy cell line (NIH3T3) [50]. In another work, novel benzimidazole-triazolothiadiazine compounds were produced and demonstrated significant aromatase inhibitory action against letrozole (IC50 = 0.032 ± 0.042 µM) [51].

Overactivation of the epidermal growth factor receptor (EGFR) is a prevalent genetic mutation in lung cancer, particularly non-small-cell lung cancer (NSCLC) [52]. Mutations in the EGFR gene can result in overproduction of the EGFR protein, causing cells to divide and expand uncontrollably, ultimately leading to cancer [53]. Benzofuran-1,2,3-triazole hybrids have been studied as possible medicines for treating lung cancer since they have anticancer characteristics [54]. Certain studies have found that some benzofuran-1,2,3-triazoles can suppress lung cancer cell proliferation and induce apoptosis [55]. One study demonstrated that a specific benzofuran-1,2,3-triazole hybrid molecule might reduce the growth of non-small-cell lung cancer cells, while another reported that the same chemical inhibited the formation of lung cancer stem cells [56].

Matrix metalloproteinases (MMPs) have critical roles in tumor growth, such as angiogenesis, tissue invasion, and migration [57]. As a result, MMPs have been identified as possible diagnostic and prognostic biomarkers in a wide range of cancers [57]. In one study, novel oxadiazole, thiadiazole, and triazole compounds were synthesized and tested for anticancer activity against A549 human lung adenocarcinoma and C6 rat glioma cell lines [57]. To investigate the association between their anticancer activity and MMP-9, the drugs’ inhibitory effects on MMPs were assessed [57]. Compounds **8** and **9**, which are N-(1,3-Benzodioxol-5-ylmethyl)-2-{[5-(((5,6,7,8-tetrahydronaphthalen-2-yl)oxy)methyl)-1,3,4-oxadiazol-2-yl]thio}acetamide and N-(1,3-benzodioxol-5-ylmethyl)-2-[(5-phenyl-1,3,4-oxadiazol-2-yl)thio]acetamide, respectively, showed promising cytotoxic effects on A549 and C6 cell lines, similar to cisplatin, without generating toxicity to the NIH/3T3 mouse embryonic fibroblast cell line [57]. Compounds **8** and **9** were the most potent MMP-9 inhibitors in this series [57]. Furthermore, docking investigations revealed that compounds **8** and **9** exhibited a high affinity for the active region of the MMP-9 enzyme [57]. Docking studies and in vitro investigations revealed that compounds **8** and **9**’s MMP-9 inhibitory activities may be relevant in the treatment of lung cancer and glioma [57].

In another work, a sequence of 3-(6-substituted phenyl-[1,2,4]-triazolo[3,4-b]-[1,3,4]-thiadiazol-3-yl)-1H-indoles (**5a**–**l**) was developed, manufactured, and tested for anti-apoptotic Bcl-2 inhibitory action [56]. The new series exhibited a selective sub-micromolar IC50 growth inhibitory effect against Bcl-Bcl-2-expressing human cancer cell lines [58]. The most powerful 6-(2,4-dimethoxyphenyl) substituted analogue 3-[6-(2,4-dimethoxyphenyl)-[1,2,4]-triazolo[3,4-b]-[1,3,4]-thiadiazol-3-yl]-1H-indole (**5k**) selectively inhibited Bcl-2-expressing cell lines, with IC50 values of 0.31–0.7 µM, while not inhibiting the Bcl-2-negative Jurkat cell line [58]. The ELISA binding affinity experiment (interrupting the Bcl-2-Bim interaction) demonstrated strong binding affinity for compound **5k** with an IC50 value of 0.32 µM [58].

The first reported histone demethylase, lysine-specific demethylase 1 (LSD1), is vital to the epigenetic regulation of gene activation and repression [59]. Several malignant tumors have shown upregulated LSD1 expression [59]. In a study, scientists developed and synthesized five series of 1,2,3-triazole-dithiocarbamate hybrids and tested their inhibitory effectiveness against LSD1 [59]. It was found that some of these compounds, especially compound tert-Butyl 4-(((1-(4-Methylbenzyl)-1H-1,2,3-triazol-4-yl)-methylthio)carbonothioyl)piperazine-1-carboxylate, demonstrated the most precise and strong inhibition of LSD1 [59]. Interestingly, compound **26** demonstrated effective and specific cytotoxicity toward LSD1 excessively expressing gastric cancer cell lines MGC-803 and HGC-27, as well as a significant reduction of cell migration and invasion when compared with 2-PCPA [59]. Furthermore, compound **26** significantly decreased tumor development in human gastric cancer cells in vivo while exhibiting no unfavorable side effects [59].

Histone deacetylases (HDACs) are key members of the epigenetics community and well-validated targets for the development of anticancer medicines [60]. In a study, a group of scientists developed and synthesized 27 new hydroxamic acid-based HDAC inhibitors (HDACis) with benzyl-triazole as the core skeleton [60]. The majority of target chemicals inhibited HDACs with high efficiency [60]. Among them, compounds N-(6-(hydroxyamino)-6-oxohexyl)-1-(4-((4-methylphenyl)sulfonamido)benzyl)-1H-1,2,3-triazole-4-carboxamide (ZM-22), 1-(4-((4-fluorophenyl)sulfonamido)benzyl)-N-(6-(hydroxyamino)-6-oxohexyl)-1H-1,2,3-triazole-4-carboxamide (ZM-23), N-(6-(hydroxyamino)-6-oxohexyl)-1-(4-(naphthalene-1-sulfonamido)benzyl)-1H-1,2,3-triazole-4-carboxamide (ZM-24), 1-(4-((4-(tert-butyl)phenyl)sulfonamido)benzyl)-N-(6-(hydroxyamino)-6-oxohexyl)-1H-1,2,3-triazole-4-carboxamide (ZM-25), 1-(4-((4-bromophenyl)sulfonamido)benzyl)-N-(6-(hydroxyamino)-6-oxohexyl)-1H-1,2,3-triazole-4-carboxamide (ZM-26), and N-(6-(hydroxyamino)-6-oxohexyl)-1-(4-(pyridine-3-sulfonamido)benzyl)-1H-1,2,3-triazole-4-carboxamide (ZM-27) with inhibition rates greater than 90% against HDACs demonstrated a significant inhibitory effect against HDAC6, with ZM-23 showing the highest selectivity toward HDAC6 over HDAC1 [60].

There has been no documented in silico research on the DFT, docking, or MD simulation of novel quinolone–triazole and conazole–triazole hybrid derivatives. Uddin et al. recently investigated the potential cancer-fighting powers of various chemical derivatives as cancer agents, utilizing computational approaches such as DFT, molecular docking, and molecular dynamics [61]. These in silico investigations provide useful information about the ligands’ potential for medical and biological uses. This study investigates the in vitro cytotoxic effect of the biological activity, pharmacology, and toxicity profiles of potential antibacterial and anticancer compounds (**1**–**20**) [62].

**Figure 1:**
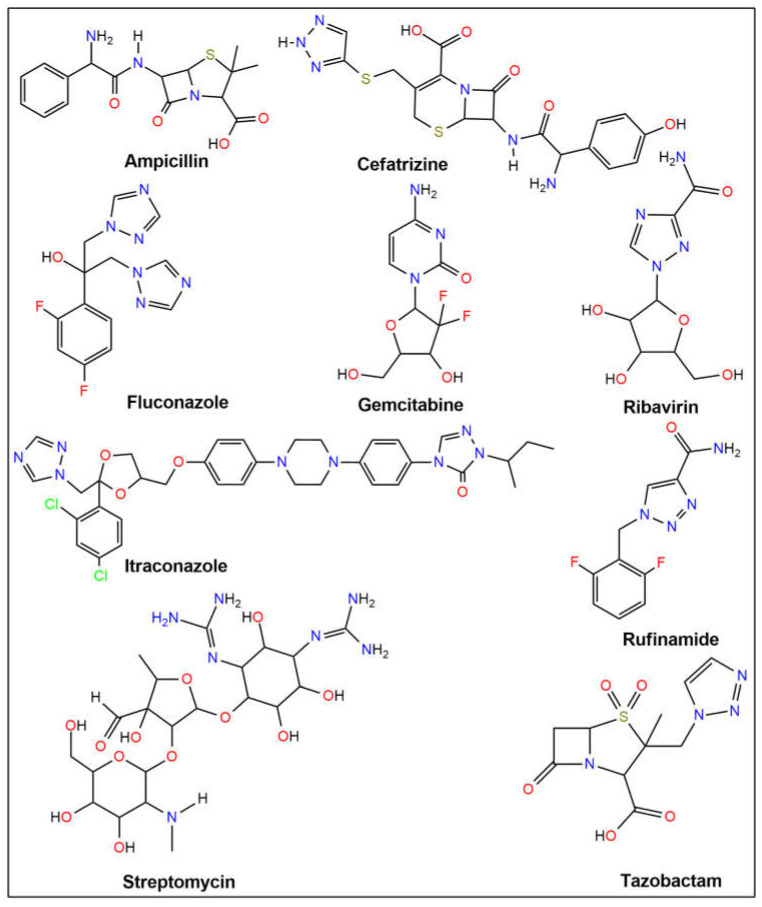
Structures of the reference drugs.

The dipole moment, chemical potential, HOMO (highest occupied molecular orbital)-LUMO (lowest unoccupied molecular orbital) gap, hardness, softness, and thermal stability were all evaluated using density functional theory (DFT). Using ampicillin, cefatrizine, fluconazole, gemcitabine, itraconazole, ribavirin, rufinamide, streptomycin, and tazobactam as reference drugs (see Figure 1), molecular docking was used to assess the samples’ binding affinities against nine different proteins, three of which are associated with each type of cancer. To forecast the possible anticancer activity spectrum of these drugs, we used the prediction of activity spectra for substances (PASS) algorithm. The protein-ligand complex’s stability was evaluated using MD simulation analysis at the active location of the protein-ligand interaction. Furthermore, molecular simulations were utilized to validate our findings and assess the entropic strengths of the medication candidates. Our research aims to create a novel class of physiologically active antibacterial and anticancer medicines.

## 2. Results and Discussion

### 2.1. In Silico Molecular Docking

**Figure 2:**
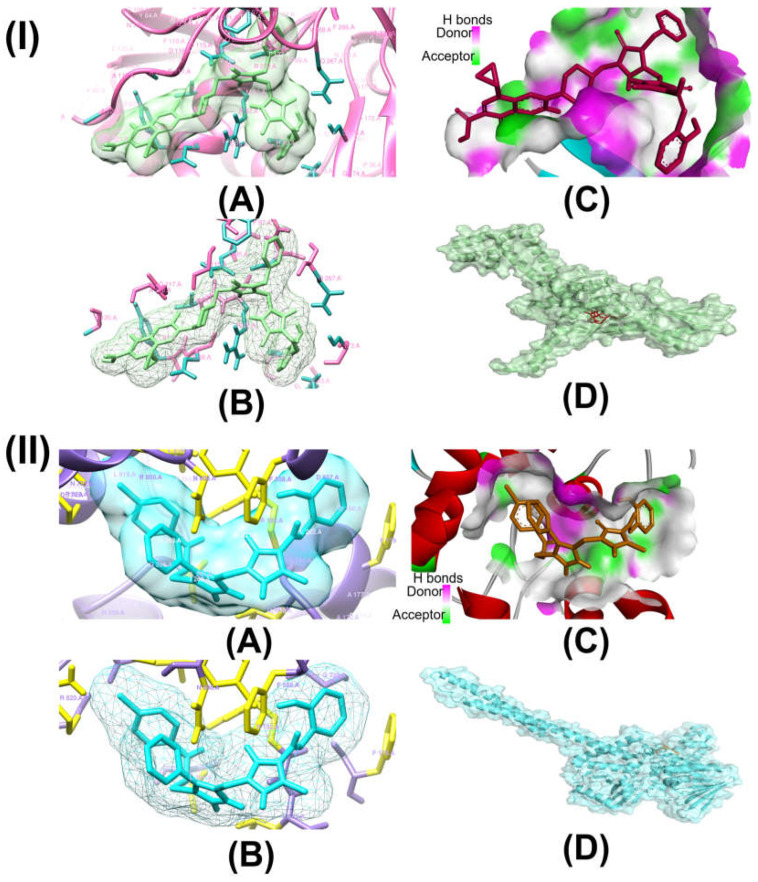
Molecular docking configurations: (**A**) a compound positioned within the protein pocket; (**B**) active site visualization; (**C**) hydrogen bonding in the solid state (Purple = Hydrogen bond donor surface, Green = Hydrogen bond acceptor surface); (**D**) protein-ligand interaction displayed in a 2D diagram for (**I**) ligand 15 with modeled protein topoisomerase II (5CDQ) and (**II**) ligand 16 with modeled protein LSD1 (2Z3Y).

We evaluated the potential of antimicrobial and anticancer chemicals and reference medications using computational docking simulations. To determine the antimicrobial properties of these compounds, we chose DNA gyrase of six microbes, which include *Enterococcus faecalis* (PDB ID:4KSG), *Mycobacterium tuberculosis* (PDB ID: 5BS8), *Staphylococcus aureus* (PDB ID: 5CDQ), *Escherichia coli* (PDB ID: 6RKU), *Mycobacterium smegmatis* (6ZT3), and *Pseudomonas aeruginosa* (PDB ID: 8BN6). We chose ten different proteins to analyze the anticancer activities of these compounds: epidermal growth factor receptor (EFGR, PDB ID: 1M17), myeloperoxidase (MPO, PDB ID: 1MHL), vascular endothelial growth factor receptor (VEGFR, PDB ID: 1Y6A), cyclin-dependent kinase 6 (CDK6, PDB ID: 2EUF), matrix metalloproteinase 1 (MMP1, PDB ID: 2J0T), B-cell lymphoma 2 (Bcl-2, PDB ID: 2O21), lysine-specific demethylase1 (LSD1, PDB ID: 2Z3Y), histone deacetylase 6 (HDAC6, PDB ID: 3PHD), aromatase (PDB ID: 3S79), and 15-lioxygenase (ALOX15, PDB ID: 4NRE). The results of these docking simulations are summarized in Table 1 and Table 2 and in Figure 2 and Figures S12–S29 in the Supporting Information. As antimicrobial agents, compounds **10** to **15** demonstrated comparatively stronger binding affinity for all six target proteins, as shown in Table 1. Compound **15** had the highest binding affinity for topoisomerase II of *E. faecalis* (PDB: 4KSG) and *S. aureus* (PDB: 5CDQ) at −9.2 kcal mol^−1^and −10.6 kcal mol^−1^, respectively. For topoisomerase II of both *M. tuberculosis* (PDB: 5BS8) and *M. smegmatis* (PDB: 6ZT3), compound **10** had the strongest binding affinity at −10.2 kcal mol^−1^ and −9.2 kcal mol^−1^, respectively, among all compounds. This could be indicative of compound **10**’s antimicrobial function against the mycobacterium genus. Toward DNA gyrase of *E. coli* (PDB: 6RKU) and *P. aeruginosa* (PDB: 8BN6), compound **12** with −9.2 kcal mol^−1^ and compound **18** with −9.8 kcal mol^−1^, respectively, demonstrated the highest affinity. As anticancer agents, compounds **10** to **17** demonstrated comparatively stronger binding affinity for all nine target proteins, as shown in Table 2. Among the ten target proteins, compound **16** displayed highest binding affinity toward six of them, which include EFGR (PDB: 1M17), MPO (PDB: 1MHL), VEGFR (PDB: 1Y6A), CDK6 (PDB: 2EUF), Bcl-2 (PDB 2O21), and ALOX15 (PDB: 4NRE), with binding affinities of −10.1 kcal mol^−1^, −9.5 kcal mol^−1^, −8.9 kcal mol^−1^, −10.1 kcal mol^−1^, −9.7 kcal mol^−1^, and −10.9 kcal mol^−1^. Compound **16** displayed the highest binding affinity against three of the target proteins, which include MMP1 (PDB: 2J0T) at −10.2 kcal mol^−1^, LSD1 (PDB:2Z3Y) at −12.0 kcal mol^−1^, and ALOX15 (PCB: 4NRE) at −10.9 kcal mol^−1^. Compound **14** showed the highest binding affinity against HDAC6 (PDB: 3PHD) at −7.9 kcal mol^−1^. Interestingly, compared to our possible molecules, the majority of reference medications showed lower binding affinities. Although molecular docking models provide useful insights into possible therapeutic choices, other factors besides binding affinity need to be taken into account when developing new medications. These include toxicity, pharmacokinetics, and metabolism. Research conducted in vivo as well as in vitro is required to completely understand the safety and effectiveness of these exciting new medications. Researchers can optimize molecules and create safe, effective drugs with the necessary qualities by combining experimental and computational approaches.

Table 1 shows information on molecular docking of antimicrobial compounds (**1**–**20**) and reference drugs.

Table 2 represents data regarding molecular docking of anticancer compounds (**1**–**20**) and reference drugs.

Table 3 highlights the effect of compounds **15** and **16** on the active components of protein topoisomerase II and LSD1, respectively, detailing bond lengths and residue counts for various bond types. Compound **15** formed six unique bonds and a total of twelve bonds with the topo II protein. ALA A:118 exhibits a Pi-Alkyl interaction at 4.88 Å, while ALA A:120 forms a carbon-hydrogen bond at 2.62 Å. ARG A:92 is engaged in Pi-Cation and Pi-Alkyl interactions at 4.64 Å, 4.99 Å, and 5.37 Å. GLN A:95 and GLN A:267 establish conventional hydrogen bonds at 2.48 Å and 2.31 Å, respectively, and GLU A:88 is involved in Pi-anion interactions at 3.79 Å and 3.88 Å. Additionally, LYS A:43 forms both Pi-cation and Pi-donor hydrogen bonds at 2.78 Å, while PHE A:97 participates in Pi-Pi T-shaped interactions at 5.08 Å and 5.78 Å. In LSD1, compound **16** formed thirteen bonds, consisting of five unique bond types. ARG A:182 forms two conventional hydrogen bonds with distances of 1.89 Å and 2.58 Å, along with a Pi-Alkyl interaction at 4.19 Å. Additionally, ARG A:820 is involved in a carbon-hydrogen bond at 2.51 Å, an alkyl interaction at 3.73 Å, and a Pi-alkyl interaction at 5.19 Å. Further hydrophobic interactions include ILE A:804 and LEU A:816, which participate in alkyl interactions at 4.24 Å and 4.99 Å, respectively. Aromatic residues such as PHE A:179 and PHE A:558 contribute to Pi-Pi T-shaped interactions at 5.09 Å and 4.95 Å, respectively, while TYR A:807 forms a conventional hydrogen bond at 2.62 Å.

Supplementary Tables S31–S48 provide 2D diagrams for all compounds and reference drugs, illustrating their interactions with protein active components. Notably, Table S35 shows the interactions of compound **14** with topoisomerase II. The interaction of compound **14** with Topo II (PDB ID: 5CDQ) revealed that it had two unique interactions, with a total of six interactions. ALA A:180 participates in a Pi-alkyl interaction at a distance of 5.35 Å, while ARG A:92 forms a Pi-alkyl interaction at 4.978 Å. Notably, conventional hydrogen bonds are observed with ASN A:334 at 2.34 Å, GLN A:95 at 2.46 Å, GLN A:267 at 2.48 Å, and SER A:112 at 2.24 Å. The interaction analysis of compound **17** with LSD1 (PDB ID: 2Z3Y) revealed that there were four unique interactions in a total of nine interactions (Table S47 in the Supporting Information). ARG A:182 engages in a Pi-alkyl interaction at a distance of 5.49 Å, while ARG A:820 participates in both Pi-sigma and Pi-alkyl interactions at distances of 2.27 Å and 4.61 Å, respectively. ASN A:806 forms a conventional hydrogen bond at 2.45 Å, and ASP A:774 contributes to binding through an attractive charge interaction at 5.25 Å. Additional Pi-alkyl interactions are observed with ILE A:804 at 5.44 Å and LEU A:816 at 4.33 Å. LEU A:816 also forms two conventional hydrogen bonds at distances of 2.56 Å and 2.59 Å. According to the results of the molecular docking and interaction analyses, compounds **15** and **16** strongly bind to topoisomerase II and LSD1, respectively. Additionally, all the compounds demonstrated favorable interactions with these two proteins. The SI, as shown in Tables S33–S50, provides 2D diagrams and bond interactions that depict how topoisomerase II and LSD1 interact with these triazole derivatives, potential antimicrobial and anticancer compounds **10** to **17,** and reference drugs fluconazole and gemcitabine.

Table 3 shows the interaction of amino acid residues between compounds and proteins.

This study investigates the complex relationships between 20 possible antibacterial and anticancer drugs and the protein targets topoisomerase II and LSD1, going beyond simple docking assessments. For comparison, nine common antibacterial and anticancer medications were included. Our results show a variety of interaction patterns within the binding sites of these proteins (Figures S30–S35 in the Supporting Information). Compounds bind to proteins in different ways, depending on the carbon-hydrogen, pi-alkyl, and pi-pi interactions. Weak interactions occur between carbon-bound hydrogen atoms and electron-rich protein areas in carbon-hydrogen bonds. By stabilizing the molecule inside the protein’s binding site, these bonds improve the interaction as a whole. Pi-alkyl linkages, which frequently involve alkyl groups in the protein residues, enable the chemicals to bind selectively to nonpolar, hydrophobic portions of the protein. Particularly in hydrophobic regions, this kind of contact helps the molecule and protein fit together more tightly. Pi-pi interactions take place between the protein’s and the compound’s aromatic rings. When aromatic systems are present, these interactions reinforce the binding, usually enhancing the stability and specificity of the compound’s interaction with the protein. All of these interactions play a major role in the compound’s capacity to bind to the target protein in a steady and targeted manner. In topo II, the predominant bonds were 100 conventional hydrogen bonds (35%), 54 pi-alkyl bonds (19%), and 46 carbon-hydrogen bonds (16%). Additionally, there were 17 pi-pi T-shaped bonds (6%), 15 alkyl bonds (5%), 12 pi-anion bonds (4%), 9 pi-donor hydrogen bonds (3%), and 6 pi-sigma bonds (2%). There were also amide-pi stacked bonds, attractive charge, and pi-pi stacked bonds, and all were present at 1%. Key residues, including Ala118, Arg92, Gln267, Gln95, Glu88, Lys43, Met113, Phe97, and Ser98, played central roles in these interactions. In LSD1, the predominant bonds were 110 pi-alkyl bonds (32%), 86 conventional hydrogen bonds (25%), and 37 carbon-hydrogen bonds (11%). Additionally, there were 27 alkyl bonds (8%), 18 pi-pi T-shaped bonds (5%), 13 amide-pi stacked (4%), 13 attractive charge, and 3 pi-anion bonds (3%). There were also pi-cation bonds, pi-donor hydrogen bonds, pi-pi stacked bonds, pi-sulfur bonds, and salt bridges, and all were present at 1%. Key residues, including Ala331, Ala359, Ala809, Arg316, Arg820, Asp555, Gly330, Pro808, Trp552, Trp751, Tyr761, and Val333 played central roles in these interactions. Compounds **15** and **16** were found through in silico research to be intriguing candidates for inhibiting topo II and LSD1, respectively, according to their persistent high performance in the in silico investigation. In comparison to other compounds and reference medications, both compounds showed increased binding affinities. They also demonstrated advantageous pharmacokinetic and physicochemical characteristics, which are critical for drug development. Moreover, compounds **15** and **16** strengthened their potential as potent inhibitors of topo II and LSD1, respectively, by forming stable and strong interactions with these proteins throughout molecular dynamics simulations at various temperatures.

### 2.2. Physicochemical and Pharmacokinetic Characteristics Analysis

Assessing the physicochemical properties of potential antimicrobial and anticancer compounds is essential to ascertaining compliance with Lipinski’s [63] and Veber’s [64] rules. For a chemical to be given orally, it has to satisfy the following Lipinski criteria: The requirements for the material are as follows: (a) minimum molecular weight (MW) of 500 g/mol; (b) minimum octanol-water partition coefficient (log P) of ≤5; (c) maximum number of H-bond donors (HBD) of five; (d) maximum number of H-bond acceptors (HBA) of ten; and (e) maximum topological polar surface area (TPSA) of 140 Å^2^. Lipinski’s list of requirements for drug bioavailability was expanded by Veber to include two additional requirements: first, the TPSA (as defined by Lipinski) needs to be less than or equal to 140 Å^2^, and second, the nrotb should be less than ten. SwissADME was used to assess whether the compounds with the highest potential biological activity fulfilled the Lipinski and Veber criteria. As Table 4 shows, not all compounds met the exact limits of the Lipinski and Veber requirements. Nonetheless, every potential antimicrobial and anticancer compound with a drug-like (bioavailability) value of 1 demonstrated that it provides a solid theoretical framework for the development of new drugs.

Table 4 summarizes the drug-likeness prediction of the quinolone-triazole and conazole-triazole hybrid derivatives (**1**–**20**) and the reference drugs.

The molecular weight of the studied compounds varied significantly and ranged from 218.21 Da for compound **1** to 764.83 Da for compound **15**. Compounds **1** to **9** had molecular weights within 500 Da, and compound10 to 20 crossed this limit. Itraconazole and streptomycin were the only two reference drugs to break Lipinski’s rule of molecular weight as they turned out to be 705.63 Da and 581.57 DA, respectively. The compounds that adhered to the molecular weight limit also complied with Lipinski’s rule of mLogP, number of hydrogen bond donors, and number of hydrogen bond acceptors, indicating their suitable hydrophilicity and their proper solubility and absorption in the body. None of the reference drugs broke the rule of mLogp, which means they are hydrophilic and have great absorption ability. On the other hand, larger compounds exceeding a molecular weight of 500 Da showed tendencies to break some of Lipinski’s criteria. Compounds **18**, **19**, and **20** exceeded the mLogP value, indicating their higher lipophilicity, low solubility, and limited bioavailability. None of the compounds crossed the number of the hydrogen bond donor limit and remained lower than 5, but the reference drug streptomycin had 12 donors, suggesting poor permeability and absorption in the body. Compounds **12** and **13** had eleven hydrogen bond acceptors, and the reference drug streptomycin had 15, exceeding the preferred amount of 10, which suggests that these two compounds will face complications in their permeability and absorption. Nine of the twenty compounds, which are compounds **1**, **2**, **3**, **7**, **8**, **9**, **18**, **19**, and **20,** complied with Veber’s rule of TPSA and were lower than 140 Å^2^ regardless of their molecular weight. The remaining compounds, which are **4**, **5**, **6**, **10**, **11**, **12**, **13**, **14**, **15**, **16,** and **17**, broke Veber’s criteria and exceeded 140 Å^2^ for TPSA, which indicates that these compounds might struggle to cross cell membranes effectively. Compounds **14**, **15** had 11 non-rotatable bonds, and for compounds **18**, **19**, and **20,** this number was 12. This is indicative of their higher flexibility, which might cause trouble in reaching the rigidity needed for good bioavailability. We also evaluated the Pan Assay Interference Compounds (PAINS) and Brenk alert for each compound. All of the compounds showed 1 PAINS alert, and as for the reference drugs, none of them demonstrated any PAINS alert, except for itraconazole, which had a PAINS alert of 2. Compounds **1**, **2**, **5**, **6**, **8**, **12**, **13**, **16**, **17**, **18**, **19**, and **20** had 1 Brenk alert, while compounds **4, 7**, **9**, **10**, **11**, **14**, and **15** had 2 alerts. Compound **3**, on the other hand, had the highest Brenk alert at 3. For reference drugs, only streptomycin showed a Brenk alert, which was 3. This raises concerns about chemical stability or the potential for side effects of the compounds. However, one or two alerts will not disqualify a compound from development, and the bioavailability potential of a compound cannot be judged on these alerts alone. Nevertheless, the compound’s chemical properties and potential risks require further investigation.

Table 5 offers an extensive assessment of the drug-likeness of eighteen possible anticancer drugs. It does this by evaluating the compounds’ expected activity against six major biological target classes: GPCR ligands (G-protein-coupled receptors), nuclear receptor ligands (NRL), kinase inhibitors (KI), ion channel modulators (ICM), enzyme inhibitors (EI), and protease inhibitors (PI). The table presents the predicted binding or activity scores for each compound in these categories, with values greater than 0 indicating highly active, between −5 and 0 moderately active, and less than −5 inactive [65]. Compounds **1** at −1.28, **3** at −1.02, and **10** to **15** at −1.49, −1.68, −1.35, −1.53, −1.63, and −1.82, respectively, had strongly negative scores against GPCR, indicating their significant ability to bind or modulate GPCRs. GPCRs play an important role in cell signaling and are a common target for many diseases, including cancer and infectious diseases, making these compounds appealing candidates for further analysis. On the other hand, compounds **2** at −0.79, **4** to **9** at −0.95, −0.90, −0.51, −0.76, −0.52, −0.72, respectively, and compounds **16** to **20** at −0.71, −0.59, −089, −0.86, −0.80 to 9 to 20, respectively, indicate they would demonstrate moderate activity against GPCRs. Compounds **10** to **15** showed highly negative ICM scores, including −2.80 for compound **10**, −2.98 for compound **11**, −2.66 for compound **12**, −2.85 for compound **13**, −2.99 for compound **14**, and −3.19 for compound **15,** demonstrating strong anticipated modulation of ion channels. Compounds **1** to **4** at −1.20, −1.14, −1.54, −1.17, respectively, compound **7** at −1.14, compound **9** at −1.11, and compounds **16** to **20** at −1.27, −1.09, −1.89, −1.88, −1.73 also showed moderately high negative ICM values. The other compounds, including compounds **5, 6**, and **8,** also showed impressive affinity toward ISM at −0.97, −0.94, and −0.82. Ion channels control the flow of ions across cell membranes, and these channels are often dysregulated in cancer cells. Also, ion channel regulation can be a key factor in antimicrobial action, particularly in fungal infections. This indicates that these compounds can be considered potential antimicrobial and anticancer compounds. Compound **10** at −2.24, compound **11** at −2.47, compound **12** at −2.08, compound **13** at −2.30, compound **14** at −2.41, and compound **15** at −2.65 demonstrated some of the most negative KI scores. Compounds **1**, **3**, **4**, **7**, **9**, **18**, **19**, and **20** also showed comparatively higher KI values at −1.07, −1.08, −1.03, −1.10, −1.04, −1.44, −1.44, and −1.31. As kinase enzymes are involved in signal transduction and play a significant role in cell growth regulation, they are key targets for cancer treatments. Kinases are also involved in the regulation of toxin secretion, virulence, and antibiotic resistance in bacteria. This suggests that most of the compounds might act as strong kinase inhibitors, making them appealing candidates for further analysis as antimicrobial and anticancer compounds. Compounds **2**, **5**, **6**, **8**, **16**, and **17,** with their values of −0.98, −0.66, −0.73, −0.69, −0.94, and, −0.86, respectively, would also be very promising candidates as antimicrobial and anticancer compounds. Nuclear receptor ligands are involved in the regulation of gene expression, which makes them an excellent target for cancer therapies. Inhibition of NRL can also reduce the virulence of pathogens. Compounds **10** to **15** with NRL scores of −2.32, −2.59, −2.20, −2.48, −2.48, and −2.76 could be chosen as appealing candidates for microbial treatment and cancer therapies. NRL values of −1.56 for compound **1**, −1.45 for compound **3**, −1.26 for compound **4**, −1.11 for compound **5**, −1.02 for compound **6**, −1.04 for compound **16**, −1.41 for compound **18**, −1.44 for compound **19**, and −1.27 for compound **20** are also indicative of their potential as antimicrobial and anticancer agents. Compounds **2**, **7**, **8**, **9**, and **17** at −0.92, −0.98, −0.76, −0.90, and −0.82 NRL scores also demonstrate their potential as antimicrobial and anticancer compounds. Compounds **1** and **10** through **15** show comparatively higher negative EI scores at −1.54, −1.51, −1.60, −1.38, −1.47, −1.59, and −1.69, respectively. The remaining compounds **2** to **9** and **16** to **20** show comparatively lower negative EI scores at −0.85, −0.98, −0.86, −0.62, −0.48, −0.96, −0.75, −0.88, −0.79, −0.73, −0.81, −0.81, and −0.72. Enzyme inhibitors (EIs) block specific enzyme functions, often a key strategy in cancer treatment. Enzyme inhibition is also crucial in antimicrobial therapies, as many microbes rely on specific enzymes to build cell walls, replicate, or defend against host immune systems. The value of the studied compounds indicates that they could become useful in fighting against infection and cancer. Proteases are enzymes that break down proteins, and inhibiting them can disrupt cancer cell function and prevent microbes from invading cells or evading the immune system. PI scores of −1.05 for compound **1**, −1.89 for compound **10**, −2.01 for compound **11**, −1.78 for compound **12**, −1.91 for compound **13**, −2.01 for compound **14**, −2.15 for compound **15**, −1.23 for compound **18**, −1.22 for compound **19**, and −1.09 for compound **20** indicate that there is a moderate chance of them functioning as protease inhibitors. Compound **2** with PI value of −0.73, compound **3** with −0.83, compound **4** with −0.87, compound **5** with −0.68, compound **6** with −0.61, compound **7** with −0.87, compound **8** with −0.69, compound **9** −0.75, compound **16** with −0.74, and compound **17** with −0.71 also indicate a higher probability of these compounds functioning as protease inhibitors. The already established referenced drugs, with their values of GPCR, ICM, KI, NRL, EI, and PI, showed that they are also able to function as antimicrobial and anticancer compounds.

Table 5 represents the drug-likeness evaluation of the quinolone–triazole and conazole–triazole hybrid derivatives (**1**–**20**) and the reference drugs.

Comprehensive pharmacokinetic aspects analysis (ADMET) is necessary to guarantee the efficient and economical development of novel medications. ADMET stands for absorption, distribution, metabolism, excretion, and toxicity. AdmetSAR (http://lmmd.ecust.edu.cn/admetsar1/, accessed on 14 September 2024) and SwissADME (http://www.swissadme.ch/index.php, accessed on 14 September 2024) were the online tools used in this study to assess the ADMET properties of all fourteen potential antimicrobial and anticancer medications. Table 6 lists the main ADMET qualities that were evaluated. These features include subcellular localization, synthetic accessibility (SA) score, cytochrome P450 enzyme inhibition (CYP2C19 and CYP3A4), blood-brain barrier (BBB) hERG inhibition, and human intestinal absorption (HIA) [66].

Table 6 presents the in silico prediction of ADMET parameters of the quinolone–triazole and conazole–triazole hybrid derivatives (**1**–**20**) and the reference drugs.

HIA score reflects how well each compound is absorbed in the human intestine, where positive values close to +1 indicate excellent absorption, and negative values suggest poor absorption potential. Compounds **1**, **3**, **8**, **12**, **13**, **17** with HIA values of +1.0000, +0.9964, and +0.9968, +9947, +9900, +9925, respectively, and compounds **18**, **19**, and **20** with +9904 exhibit almost perfect HIA, making them excellent candidates for oral bioavailability. Compounds **2**, **4**, **5**, **7**, **9**, **10**, and **14** also show good absorption potential with values of +0.9483, +0.9625, +0.9493, 0.9875, +0.9475, respectively, and compounds **10** and **14** both with +0.9638. Their absorption capability is comparatively lower but still favorable. Compounds **6** with +0.9126 and compounds **11** and **15,** both with +0.9335, indicate moderate but reliable absorption. Most reference drugs show diverse absorption abilities. Rufinamide and itraconazole have near-perfect values at +1.0000 and +0.9973, while antibiotics like ampicillin and cefatrizine exhibit very low values at −0.9270 and −0.9522, suggesting poor absorption.

The blood-brain barrier (BBB) permeability values reflect each compound’s ability to penetrate the central nervous system. Compounds **1**, **2**, **3**, **7**, **8**, and **9** show strong permeability, with values of 0.9391, +0.8026, +0.9002, +0.8430, +0.8669, and +0.7982, respectively. Compounds **4**, **5**, and 6 exhibit lower permeability with values of +0.6926, +0.6651, and +0.6327. Compounds **10**, **11**, **12**, **13**, **14**, and **15** show poor permeability with a negative value of −0.8199, −0.7924, −0.7511, −0.7126, −0.8199, and −0.7924. In contrast, compounds **16** and **17,** with +0.7942 and +0.6399, and compounds **18**, **19**, and **20,** with +0.8728, show better permeability. Fluconazole, gemcitabine, ribavirin, and rufinamide exhibit high permeability values of +0.9382, +0.9693, +0.9381, and +0.9777. On the other hand, ampicillin, cefatrizine, itraconazole, streptomycin, and tazobactam have poor values of −0.9961, −0.9833, −0.6151, −0.9712, and −0.9659, respectively. This range of values indicates differing abilities of the compounds and reference drugs to penetrate the central nervous system.

The hERG_pIC50 values indicate the potential for cardiac toxicity due to inhibition of the hERG channel. Compounds **3**, **4**, **5**, and **7,** with hERG_pIC50 values of 0.8610, 0.8919, 0.8382, and 0.8850, respectively, suggest significant inhibition and a moderate to high potential for cardiac side effects. Compounds **1**, **2**, **6**, **8,** and **9** show hERG inhibition values of 0.7734, 0.7528, 0.7789, 0.7863, and 0.7144, respectively, which reflect a lower inhibition value of reduced cardiac risk compared to others. Compounds **10**, **11**, **12**, **13**, **14**, and **15** exhibited the lowest hERG inhibition values, with scores of 0.6779, 0.6345, 0.5613, 0.6078, 0.6779, and 0.6345, respectively, all signifying minimal risk for cardiotoxicity. Compounds **16** and **17** demonstrated values of 0.8315 and 0.8949, and compounds **18**, **19**, and **20,** with values of 0.8437 indicate significant inhibition. Among the reference drugs, other than itraconazole at 0.5782, all other reference drugs presented a high level of inhibition risk. These values highlight a range of hERG inhibition among the compounds and reference drugs, with some posing higher risks than others.

The values for CYP2C19 inhibition for each compound reflect their potential to inhibit this enzyme. The two most effective compounds against the CYP2C19 enzyme were compounds **1** and **3,** with activity values of 0.8711 and 0.8958, respectively. Compounds **4**, **5**, **6,** and **8,** respectively, demonstrated values of 0.7727, 0.7965, 0.7407, and 0.7570, all of which exhibited moderate enzyme inhibition. Compounds **2**, **7** and **9** demonstrated slightly lower values of 0.6314, 0.6117, and 0.6524, respectively, and weaker inhibition. On the other hand, compounds **10**, **11**, **12**, **14,** and **15** had values of 0.7168, 0.7440, 0.7627, 0.7168, and 0.7440, respectively, which indicate mild inhibition; compound **13** showed moderate to mild inhibition at a value of 0.8121. Compounds **16** and **17** both exhibited weak inhibition with values of 0.5315 and 0.5000, while compounds **18**, **19,** and **20** all had a comparable inhibition value of 0.6195, showing a mild potential for inhibition of CYP2C19. Among the reference drugs, ampicillin, ribavirin, and streptomycin exhibited very high CYP2C19 inhibition values of 0.9399, 0.9095, and 0.9026, respectively, indicating very strong inhibition of the CYP2C19. On the other hand, cefatrizine, gemcitabine, and tazobactam were found to be moderately inhibiting, with inhibition values of 0.7493, 0.8478, and 0.7143, respectively. Fluconazole, itraconazole, and rufinamide, on the other hand, had relatively lower values of 0.5320, 0.5703, and 0.5561, respectively, indicating a weaker inhibition. Overall, values reveal varying levels of CYP2C19 inhibition across the compounds and reference drugs, from strong to weak potential.

The values for CYP3A4 inhibition, which is an important enzyme in drug metabolism, depict the potency of the compound to inhibit a particular enzyme. Compound **1**, compound **2**, compound **3**, and compound **5** indicated the strongest inhibition values of 0.9465, 0.9628, 0.9421, and 0.9006, respectively, for the CYP3A4 inhibition. On the contrary, Compounds **4**, **6**, and **8** produced comparatively lower values of 0.8210, 0.8156, and 0.8270, indicating moderate inhibition. Compounds **7** and **9** showed low inhibiting potential, having scores of 0.6447 and 0.5191, respectively. Compounds **10**, **12**, **13**, **14**, **16**, and **17** had even lower scores of 0.6288, 0.500, 0.6447, 0.6288, 0.6792, and 0.5979, respectively, suggesting a low inhibition possibility. However, compounds **11** and **15** were slightly better in inhibition, where both recorded the value of 0.7540. Compounds **18**, **19,** and **20** have precisely the same inhibition value of 0.6509, which is suggestive of mild to moderate inhibition as well. Among the reference drugs, ampicillin, fluconazole. gemcitabine, ribavirin, streptomycin, and tazobactam all recorded moderate inhibition values of 0.8309, 0.8196, 0.9032, 0.9535, 0.8867, and 0.8596, respectively. Cefatrizine and rufinamide had lower values of 0.7150 and 0.7995, respectively, meaning less inhibition. Itraconazole also exhibited a relatively low inhibition score of 0.5279, which indicates very low inhibition. These values generally indicate a lack of consistency in the degree of CYP3A4 inhibition across the compounds and reference drugs, with some demonstrating strong inhibition while others showing weak inhibition.

The distribution of each compound at the subcellular level allows for the deduction of the cellular effector sites of the compounds. Most of these compounds are believed to accumulate primarily in the mitochondria. Some of these compounds have also been reported to possess strong mitochondrial localization properties. Compounds **1**, **2**, **3**, **4**, **5**, **6**, **7**, **8**, and **9** all showed a significant likelihood of mitochondrial localization, with values of 0.7809, 0.7452, 0.8127, 0.7928, 0.8182, 0.7846, 0.7725, 0.9005, and 0.7270, which indicate a high probability to be present within mitochondria. However, compounds **10**, **11**, **12**, **13**, **14**, and **15** display some differences with the values of 0.4652, 0.5160, 0.5847, 0.6287, 0.4652, and 0.5160, respectively, exhibiting a relatively low probability of being localized in mitochondria. Compounds **16** and **17** are also assumed to possess mitochondrial targeting capacities, with the values of 0.7179 and 0.7157, respectively. Moderate mitochondrial targeting is suggested. Compounds **18**, **19**, and **20** demonstrate the same values for mitochondrial localization, 0.7188, indicating that these compounds are very likely to be found in the mitochondria. Reference drug information showed that ampicillin is expected to be situated in a lysosome with an index of 0.5707, while gemcitabine is expected to be in a nucleus and rated 0.4156. Other reference drugs were thought to be located within the mitochondria. In general, this illustrates a clear trend of most of the compounds being preferentially distributed to the mitochondria, with some being directed toward the lysosomes and nucleus.

The SA scores indicate how easily each compound can be prepared. The compounds **1** to **8** can be considered moderate synthetic difficulty, with calculated scores ranging from 2.59 to 3.72, indicating that such synthesis is easier for them. Compound **9** scored 3.75, while compounds **10** to **15** are highly complex, as seen from their scores ranging from 5.29 to 5.43, implying greater difficulty in their synthesis. Compounds **16** and **17** have simple synthesis with scores of 4.25 and 4.83, respectively. For compounds **18**, **19**, and **20**, they all have the same score of 5.41, which means they are very complicated. With regard to reference drugs, ampicillin scored 4.16, while cefatrizine came second at 4.87, and third is fluconazole at 2.45, which means that fluconazole is easier to synthesize. Gemcitabine got a score of 3.71. Itraconazole scored 5.77, indicating high complexity, and ribavirin scored 3.89. With a score of 2.22, rufinamide will be easy to synthesize; in contrast, streptomycin’s complexity is the highest at 6.92, and tazobactam scored at 4.23.

### 2.3. In Silico Molecular Dynamics (MD)

The stability and interactions of compounds **12**, **14**, **15**, and reference medication fluconazole with the topoisomerase II protein, as well as compounds **13**, **16**, **17**, and reference drug gemcitabine with the LSD1 protein, during a 100 ns period were evaluated using MD simulations. Molecular docking experiments suggested that compound **15** could be a promising treatment candidate since it demonstrated a high binding affinity of −10.6 kcal mol^−1^ with topo II, and compound **16** showed a binding affinity of −12.0 kcal mol^−1^ with LSD1. Thus, a comprehensive MD simulation was run using protein complexes with compounds **12**, **14, 15**, and fluconazole as the reference drug along with the unoccupied topo II (PDB: 5CDQ) and with compounds **13**, **16**, **17**, and gemcitabine as the reference drug along with the unoccupied LSD1 (PDB: 2Z3Y). The MD trajectory provided insight into the structural alterations, stability, and lingering oscillations in the complexes and the unoccupied protein throughout the 100 ns simulation. The final MD trajectories were used to compute and compare the RMSDs, RMSFs, Rg, hydrogen bond (HB) analysis, and principal component analysis (PCA). Furthermore, to shed light on configurational changes that occur at various temperatures, the selected protein-ligand complex was mimicked at four different temperatures (300, 305, 310, and 320 K).

Based on the RMSD values of the protein–compound complexes, compounds **12**, **14**, and **15** with topoisomerase II, as well as compounds **13**, **16**, and **17** with LSD1, demonstrated greater stability during the simulation compared to fluconazole and gemcitabine, showing minimal deviation from their initial positions (Figures S36–S46 and Tables S51–S62 in the Supporting Information). Because the RMSD values of the compounds were greater than those of the protein, the compound–protein complex as a whole had a lower RMSD value than the individual RMSD values of the protein and compounds. These findings suggest that despite the expected dynamic and thermal changes of the atoms, there is a robust and continuous connection between the chemical and the protein that maintains the general structure of the complex. These results also imply that the stability and dynamics of solvent molecules in the four complexes may be affected by differences in the shape and size of the binding sites, as well as the strength of the compound–protein interactions. It is crucial to keep in mind that various simulation parameters may have an impact on the RMSD values of ions and water. The force field, simulation time, and binding site design are some of these parameters. Therefore, more investigation is suggested to determine the root causes of the observed RMSD discrepancies. This study may look at solvent density profiles, residency intervals, and hydrogen bonding patterns. The RMSD values for ions and water essentially show the stability and dynamics of solvent molecules in compound–protein complexes. Since the observed variability in RMSD values may indicate variations in compound–protein interactions, more research and validation techniques are required.

RMSD profiling was used to evaluate the simulated complex’s stability and dynamic behavior during a 100 ns period. The 100 ns MD simulation was conducted at four different temperature conditions: 300, 305, 310, and 320 K. The root-mean-square deviation (RMSD) values for values for the topoisomerase II–compound **15** and LSD1–compound **16** complexes were recorded. Both compounds consistently maintained stable conformations. These findings suggest that because of the strong and persistent connection between the protein and chemical at 310 K, the complex can maintain structural integrity even in the face of spontaneous temperature and dynamic variations. Ultimately, the MD simulations demonstrate the stability of the combination and the likelihood of a strong, long-lasting bond between the chemical and protein.

As shown in Figures S47–S51 and Tables S63–S66 in the Supporting Information, the study highlighted how different parts of the proteins and their bound compounds moved during the simulation. Higher RMSF values pointed to more flexible or mobile regions, while lower values indicated areas that remained relatively rigid.

Minimal changes were seen in all protein–compound complexes, suggesting a significant degree of flexibility. Amino acid flexibility or interactions with the chemicals or solvent molecules could be the cause of the changes seen at amino acid positions 4500 and 6500 for topoisomerase II protein–compound complexes. The origins of these differences may become clearer with more research into how these residues specifically interact with other sections of molecules or proteins. The protein–compound complexes’ different RMSF values indicate differences in their flexibility or mobility, which may have an impact on their biological activity. The observed differences at atoms 4500 and 6500 emphasize the significance of thorough simulation studies, as well as the intricacy of protein–compound dynamics. For the development of therapeutic medicines and efficient drug discovery, an extensive understanding of these dynamics is essential.

The radius of gyration (Rg) is a commonly used indicator of molecular rigidity in molecular dynamics (MD) simulations, frequently utilized to track conformational changes. The Rg values of compounds **12**, **14**, **15**, and fluconazole bound to topoisomerase II and compounds **13**, **16**, **17**, and the reference medication gemcitabine bound to LSD1 were compared in this investigation throughout a 100 ns simulation period as shown in Figures S52–S62 and Tables S67–S78 in the Supporting Information.

The compounds had a range of Rg (radius of gyration) values between 2.925 nm and 3.13 nm when coupled to topoisomerase II and between 3.36 nm and 3.725 nm when attached to LSD1. Rg values for the free (unbound) molecules, on the other hand, ranged from 0.525 nm to 0.678 nm for LSD1 and from 0.55 nm to 0.63 nm for topoisomerase II. This discrepancy suggests that while bound to the protein, the compounds experienced greater mobility and conformational changes, which led to a greater gyration radius. While the compounds remained more compact when unbound, the higher Rg values in the bound state indicate that they dispersed and moved a greater distance as a result of structural alterations mediated by proteins. These little oscillations imply that the complexes changed structurally without losing their stability. The compounds become more compact when attached to the proteins, as seen by the reduced Rg differences in the complexed forms compared to the more significant Rg differences in the free compounds.

The Rg analysis for the topoisomerase II-compound **15** and LSD1-compound **16** complex at various temperatures showed consistent behavior over the course of the 100 ns simulations, with hardly any variations in Rg values. This suggests that despite temperature changes, the topoisomerase II-compound**15** complex and the LSD1-compound **16** complex retained their structural stability. Compounds **15** and **16** appear to adopt a more compact structure when bound to topoisomerase II and LSD1, respectively, which is similar to that of proteins and contributes to their overall stability under varying thermal conditions, based on the slight variations in Rg values between the complexed form and the free compound.

**Figure 3:**
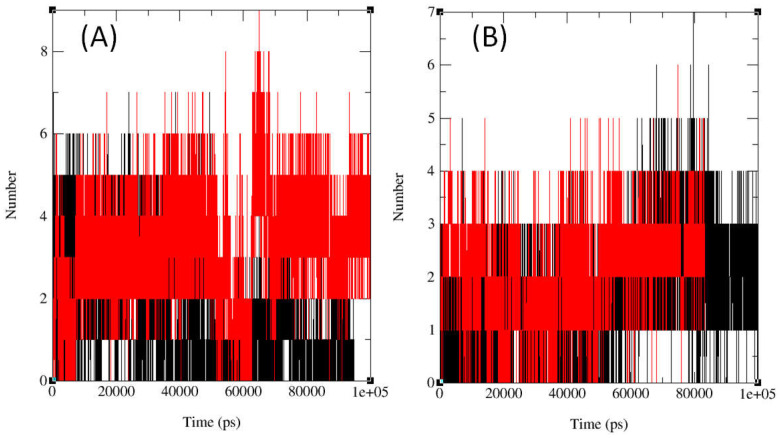
Plots depicting the count of intermolecular hydrogen bonds over time (ps) for hydrogen bond stabilization in (**A**) the protein complex of topoisomerase II and compounds **12**, **14**, **15**, and reference drug fluconazole; and (**B**) the protein complex of LSD1 and compounds **13**, **16**, **17**, and reference drug gemcitabine (Black = protein-compound complex, Red = protein-reference drug complex).

In protein–compound interactions, hydrogen bonds are essential and offer important information about binding strength and selectivity. Figure 3 illustrates variations in the total number of hydrogen bonds formed over 100 ns simulation, varying between 0 to 8.5 for DNA gyrase and 0 to 7 for LSD1, by examining the interactions between compounds **12**, **14**, **15**, fluconazole and the active sites of topo II and compounds **13**, **16**, **17**, gemcitabine and the active sites of LSD1 through MD simulation. This dynamic pattern suggests that the dynamic interaction between compounds and the two proteins is influenced by conformational changes, compound mobility, and protein uniqueness. For the rational development of medications, it is imperative to comprehend this dynamic component. Figure 3 and the Supplementary Figures S63 and S64 display the temporal evaluation of intermolecular HBs across a 100 ns simulation, emphasizing the significance of HB interactions in maintaining system stability. Remarkably, the chemical displayed the highest hydrogen bond prevalence, with the target protein residues between 60 and 80 ns for topo II and 70 and 90 ns for LSD1 at room temperature.

It is essential to keep an eye on the system’s temperature in molecular dynamics simulations to guarantee stability. During the 100 ns simulation period in this work, the temperature of the proteins was found to be reasonably steady. This suggests that the system stayed within the proper temperature range and that the simulation was well managed. Figures S65–S67 in the Supporting Information illustrate how the system’s potential energy, which represents atom-to-atom interactions, fluctuated over the course of the simulation. The potential energy for compound **12** was between −1.3325 × 10^6^ kJ/mol and −1.3225 × 10^6^ kJ/mol, compound **14** ranged from −3.16 × 10^6^ kJ/mol to −3.145 × 10^6^ kJ/mol, compound **15** varied between −3.16 × 10^6^ kJ/mol and −3.1575 × 10^6^ kJ/mol, and fluconazole ranged between −1.3325 × 10^6^ kJ/mol and −1.3225 × 10^6^ kJ/mol. Similarly, the potential energy for compound **13** was between −1.945 × 10^6^ kJ/mol and −1.935 × 10^6^ kJ/mol, compound **16** ranged from −1.945 × 10^6^ kJ/mol to −1.9325 × 10^6^ kJ/mol, compound **17** varied between −1.945 × 10^6^ kJ/mol and −1.935 × 10^6^ kJ/mol, and gemcitabine ranged between −1.945 × 10^6^ kJ/mol and −1.935 × 10^6^ kJ/mol. These fluctuations imply that the interactions between the atoms in the system are dynamic and constantly changing. Therefore, further investigation is needed to understand the basic principles of protein-ligand interactions and identify the reasons behind these variances. In summary, the study’s steady temperature points to a carefully calibrated simulation, but the possible energy fluctuation highlights how dynamic the interactions between proteins and ligands are.

The Coulomb (SR) energy values derived from the MD simulation show that the electrostatic interactions between the protein and ligand are energetically favorable and stable throughout the simulation. These values ranged from −3.68 × 10^6^ kJ/mol to 1.5825 × 10^6^ kJ/mol for compounds **12**, **14**, **15**, and reference drug fluconazole and −2.33 × 10^6^ kJ/mol to −2.315 × 10^6^ kJ/mol for compounds **13**, **16**, **17**, and reference drug gemcitabine (Figures S68–S70 in the Supporting Information). The constant and negative values imply that electrostatic interactions within the protein-ligand complex were not significantly altered. Researchers can acquire an understanding of the particular interactions between the ligand and the protein by examining the Coulomb (SR) energy’s magnitude and distribution. To fully comprehend the energetics of the protein-ligand interaction, more research may be required, such as free energy calculations.

The van der Waals forces between atoms are characterized by the Lennard-Jones short-range (LJ-SR) energy (Figures S71–S73 in the Supporting Information), which stayed consistent between 1.7 × 10^5^ kJ/mol and 4.66 × 10^5^ kJ/mol for compounds **12**, **14**, **15**, and reference drug fluconazole and between 2.55 × 10^5^ kJ/mol and 2.65 × 10^5^ kJ/mol for compounds **13**, **16**, **17**, and reference drug gemcitabine, demonstrating that during the simulation, the attraction and repulsive forces between the atoms stayed constant.

**Figure 4:**
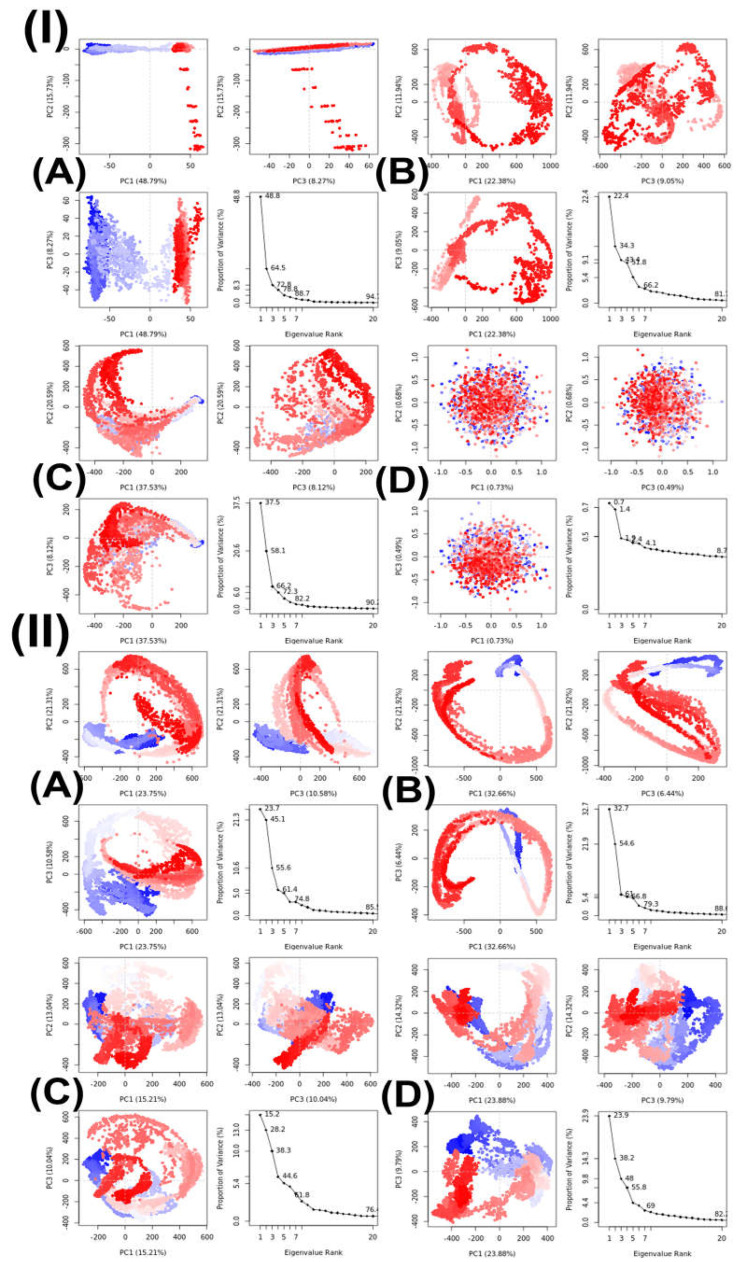
Principal component analysis (PCA) of MD trajectories at (**A**) 300 K, (**B**) 305 K, (**C**) 310 K, and (**D**) 320 K of (**I**) topoisomerase II-compound **15** complex and (**II**) LSD1-compound **16** complex (intermediate states are indicated by white dots, energetically unstable conformations are depicted by blue dots with scattering, and stable conformation states are represented by red dots).

Principal component analysis (PCA) was used to examine the MD trajectories of the protein–compound complexes (topoisomerase II-compound **15** and LSD1-compound **16**) at different temperatures, with a focus on C atoms. Within subsets of the principal components that developed during the MD simulations, this study examined variance, collective motions, and changes in the conformational orientations of proteins, as illustrated in Figure 4. Using the Bio3D tool, the PCA calculated the MD trajectories of the topoisomerase II-compound **15** and the LSD1-compound **16** complex at 300, 305, 310, and 320 K [67,68].

After isolating the trajectory’s primary motion inside a smaller subset, the first three eigenvectors (PC1, PC2, and PC3) were compared. The variance gathered by these eigenvectors is represented by the colored dots, and the complex’s sample count is indicated by the dots’ color change from blue to white to red. Table 6 displays the primary movements identified within the topoisomerase II-compound **15** complex and LSD1-compound **16** complex at different temperatures; these movements were isolated from a smaller dataset and examined using the first three eigenvectors (PC1, PC2, and PC3). Both topoisomerase II-compound **15** complex and LSD1-compound **16** complex exhibited the highest variability in PC1, but for topoisomerase II-compound **15** complex, it was at 300 K (48.79%), and for LSD1-compound **16** complex, it was at 305 K (32.66%). PC1 variability at 305 K (22.98%) for topoisomerase II-compound **15** complex was lower than the variability at 300 K, but at 310 K (31.53%), the variability increased significantly; however, it was still was lower than at 300 K and the variabilities were the lowest at 320 K (0.73%). Both PC2 and PC3 variabilities were the lowest at 320 K (0.94% and 0.49%, respectively), but PC2 was the highest at 310 K (20.59%) and PC3 was the highest at 305 K (9.05%) and the lowest at 320 K (0.49%). For LSD1-compound **16** complex, the lowest PC1 and PC2 variabilities were at 310 K (15.21% and 13.04%), and the lowest PC3 variability was at 305 K (6.44%). The highest PC2 variability was at 305 K (21.92%), and the highest PC3 variability was at 300K (10.58%). Additionally, the values from the principal component analysis (PCA) for the given dataset of topoisomerase II-compound **15** complex were 0.09, 0.48, 0.70, and 0.01 for 300, 305, 310, and 320 K, respectively, and 0.83, 0.89, 0.78, and 32 for 300, 305, 310, and 320 K, respectively, of LSD1-compound **16** complex, establishing convergence in the simulation across the range 0 < H < 0.5 [69]. Important insights have been obtained based on the simulation findings at physiological body temperature (310K), which indicate that compound **15** is significantly associated with topoisomerase II and compound **16** is strongly related to LSD1. Based on in vitro and in silico research, these findings suggest that compound **15** interacts positively with topoisomerase II while compound **16** interacts positively with LSD1. To prove compound **15**’s effectiveness as a topoisomerase II inhibitor and compound **16**’s efficacy as an LSD1 inhibitor, more in vivo studies are required.

Table 7 shows the PCA for compounds with their corresponding target proteins.

In a previous in vitro study, among the 20 synthesized compounds, compounds **10**–**15** showed remarkable activity against 7 of the 9 tested microorganisms. The results for the active derivatives of every compound assessed for antibacterial activity are displayed in Table 8. Low-activity chemicals are not included in the table. Since there are no discernible relationships between structure and activity, the compounds’ antibacterial activity seems to be significantly impacted by their chemical composition. This fact explains the exceptional activity of compounds **10**–**15** against bacteria with MIC values ranging from 0.24 μg/mL to 1.9 μg/mL. These compounds’ strong activity may be explained by the presence of a fluoroquinolone core in their structures. Norfloxacine and ciprofloxacine have MIC values that are similar to those of compounds **10**–**15**. These six compounds, however, showed negligible activity against Candida albicans ATCC 60193 and Saccharomyces cerevisiae RSKK 251. The remaining fourteen compounds showed little to no action against the examined microorganisms.

Table 8 summarizes the antimicrobial activities of several prepared compounds.

## 3. Materials and Methods

### 3.1. Computational Analysis

**Figure 5:**
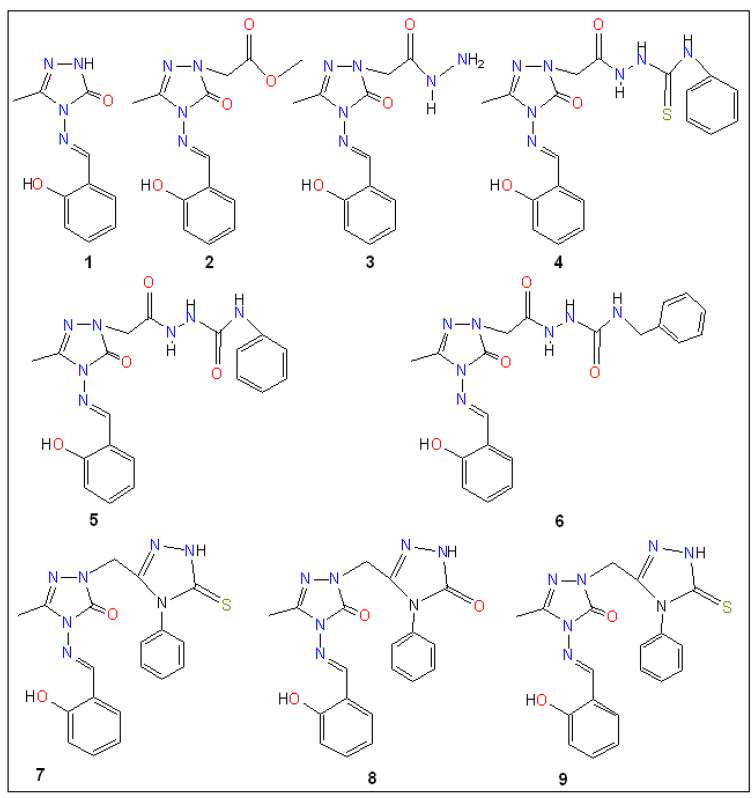
Structures of quinolone–triazole and conazole–triazole hybrid derivatives (**1**–**9**).

**Figure 6:**
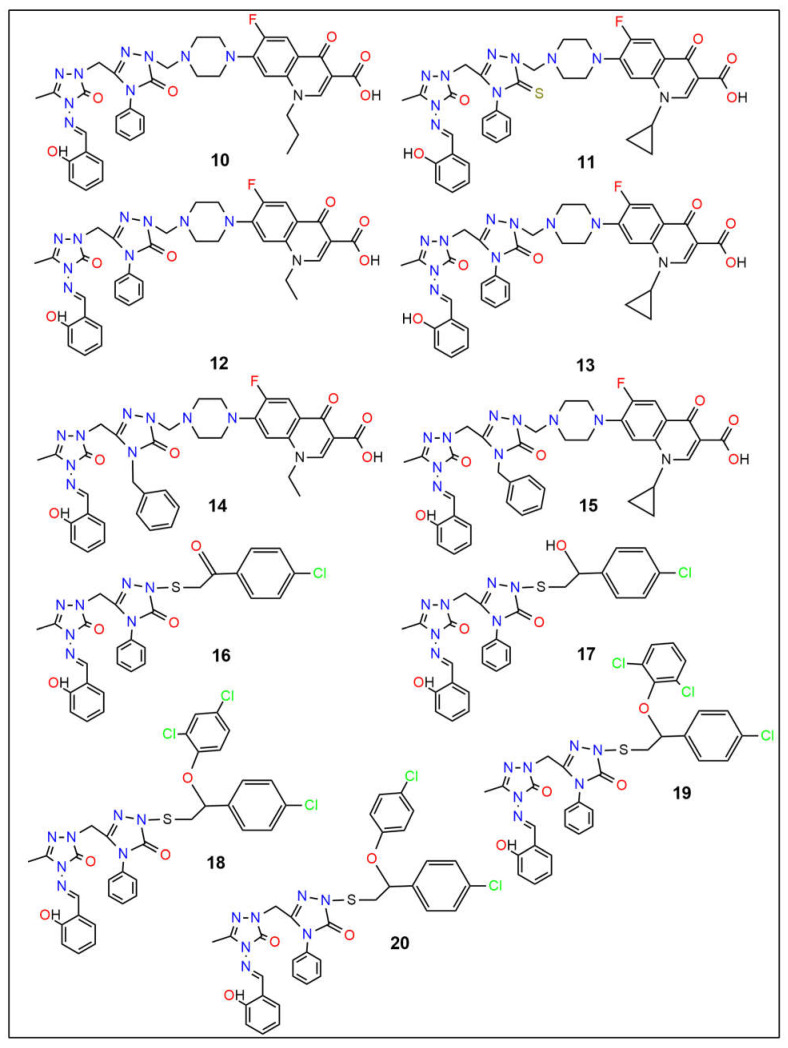
Structures of quinolone–triazole and conazole–triazole hybrid derivatives (**10**–**20**).

The 20 hybrid derivatives of quinolone–triazole and conazole–triazole hybrids generated with microwave assistance are shown in Figure 5 and Figure 6 in ChemBioDraw Ultra 14.0 (Waltham, MA, USA) as antibacterial and anticancer compounds (**1**–**20**) [62]. The SDF structures of the reference medications, which came from the PubChem chemical database (Bethesda, MD, USA), are shown in Figure 1. The geometry optimization results for the structures (**1**–**20**) and reference drugs using Gaussian 16 (Wallingford, CT, USA) are comprehensively summarized in Tables S3–S32 and Figures S4 and S5 in the Supplementary Information (SI) [70]. In order to make sure that every structure was at its energy minimum, several simulations were run. The use of the B3LYP/6-31G(d,p) level of theory is precise and efficient, as demonstrated by earlier studies [71,72,73,74,75,76,77,78]. The energy distribution of frontier molecular orbitals (FMOs), which go from the HOMO to the LUMO, was examined using GaussView 6 software (Wallingford, CT, USA) to create maps of molecular electrostatic potential (MEP) [79]. Comprehensive visual representations of the optimized HOMO and LUMO, MEP maps, and all structures related to docking and molecular dynamics are displayed in Figures S1–S3 and S6–S11 and Table S1 in the Supplementary Information (SI).

An indicator of the stability of a molecule is the FMO energy gap, which is the difference between the LUMO and HOMO energies. These HOMO and LUMO values were used to generate the compounds’ overall chemical reactivity descriptors. These indicators cover a variety of attributes, including electron binding energy (H), ionization potential (IP), chemical potential (µ), maximal charge acceptance (∆N_max_), global chemical softness (σ), global chemical hardness (η), energy change (∆E), electrophilicity (ω), electronegativity (χ), and electron affinity (EA) [80,81,82,83]. The formulas utilized to determine these indicators are as follows:

$$\begin{array}{c} \mathrm{IP}\;=\;-E_{\mathrm{HOMO}};\;\mathrm{EA}\;=\;-E_{\mathrm{LUMO}};\;µ=(\mathrm{IP}+\mathrm{EA})/2;\;H=(\mathrm{IP}-\mathrm{EA});\;\chi =-\eta ;\;\omega ={µ}^{2}/(2\eta );\;\sigma =1/\eta \\ ∆N_{\max}\;=\;-(µ/\eta );\;∆E\;=\;-\omega ;\;E_{\mathrm{gap}}\;=\;E_{\mathrm{LUMO}}\;-\;E_{\mathrm{HOMO}} \end{array}$$

### 3.2. Evaluation of Physicochemical and Pharmacokinetic Properties

AdmetSAR (Shanghai, China; http://lmmd.ecust.edu.cn/admetsar1/, accessed on 14 September 2024) and SwissADME (Vaud, Switzerland; www.swissadme.ch, accessed on 14 September 2024) are publicly accessible web-based tools that we used to estimate and evaluate the physicochemical characteristics, medicinal chemistry, drug-likeness, lipophilicity, pharmacokinetics, and water solubility [84,85]. To make this experiment easier to undertake, we employed the Simplified Molecular Input Line Entry System (SMILES) standard format. First, we used ChemBioDraw Ultra 14.0 (Waltham, MA, USA) to construct the chemical structures of compounds (**1**–**20**). These were then converted to SMILES format so that data in the MDL Molfile format could be assembled.

### 3.3. Pharmacological Attributes

We discovered a wide range of pharmacological effects of the 20 compounds (**1**–**20**) and the reference drugs, using the prediction of activity spectra for substances (PASS, Moscow, Russia; http://www.way2drug.com/PASSOnline/predict.php, accessed on 15 September 2024) [86]. These effects included antibacterial, DNA gyrase inhibitor, antimycobacterial, aminopeptidase inhibitor, protease inhibitor, and DNA synthesis inhibitor. Information regarding biological substances permitted in the USA and Russia can be found on this web server [86].

### 3.4. Molecular Docking

#### 3.4.1. Compound Preparation

We generated three-dimensional (3D) structural data files for antitumor and anticancer triazole derivatives (**1**–**20**) using GaussView version 6 (Wallingford, CT, USA) and reference drugs (ampicillin, cefatrizine, fluconazole, gemcitabine, itraconazole, ribavirin, rufinamide, streptomycin, and tazobactam) in PDB format from the RSCB PDB (San Francisco, CA, USA; https://www.rcsb.org/, accessed on 22 September 2024) [87]. The structures were then optimized using the B3LYP/6-31G(d,p) method using the Gaussian 16 program. By decreasing their respective energies, molecules were prepared for docking using PyRx 0.8 (available at https://pyrx.sourceforge.io/, accessed on 24 September 2024) [88]. The next step was to use the OpenBabel plugin to transform each compound’s LOG file into a PDBQT file [88].

#### 3.4.2. Target Protein Preparation

We used molecular docking methods to identify proteins that would be suitable for potential chemical binding using proteins from the RCSB Protein Data Bank (San Francisco, CA, USA; https://www.rcsb.org/, accessed on 22 September 2024) and the AlphaFold Protein Structure Database (Cambridgeshire, UK; https://alphafold.ebi.ac.uk/, accessed on 22 September 2024) [87,89]. Six microbial DNA gyrase and ten cancer-inducing proteins were selected as targets for the study. The six microbes were *Enterococcus faecalis* (PDB ID:4KSG), *Mycobacterium tuberculosis* (PDB ID: 5BS8), *Staphylococcus aureus* (PDB ID: 5CDQ), *Escherichia coli* (PDB ID: 6RKU), *Mycobacterium smegmatis* (6ZT3), and *Pseudomonas aeruginosa* (PDB ID: 8BN6) and the ten cancer-inducing proteins were epidermal growth factor receptor (EFGR, PDB ID: 1M17), myeloperoxidase (MPO, PDB ID: 1MHL), vascular endothelial growth factor receptor (VEGFR, PDB ID: 1Y6A), cyclin-dependent kinase 6 (CDK6, PDB ID: 2EUF), matrix metalloproteinase 1 (MMP1, PDB ID: 2J0T), B-cell lymphoma 2 (Bcl-2, PDB ID: 2O21), lysine-specific demethylase1 (LSD1, PDB ID: 2Z3Y), histone deacetylase 6 (HDAC6, PDB ID: 3PHD), aromatase (PDB ID: 3S79) and 15-lioxygenase (ALOX15, PDB ID: 4NRE). Ramachandran plots by PROCHECK [90,91,92,93,94,95,96,97,98], overall quality factor by ERRAT [99] and average atomic model (3D) to amino acid sequence (3D-1D) score of 0.1 by Verify3D [100,101,102,103,104] were used to evaluate the quality of these protein structures via the SAVESv6.0 website (Los Angeles, CA, USA; https://saves.mbi.ucla.edu/, accessed on 16 September 2024), as shown in Table S2 in the Supporting Information. ProSA-web (Salzburg, Austria; https://prosa.services.came.sbg.ac.at/prosa.php, accessed on 16 September 2024) was used to determine the Z-scores and local model quality of these proteins [105,106]. STRING (Geneva, Switzerland; https://version-11-0.string-db.org/, accessed on 16 September 2024) database was employed to find the interactions between proteins [107]. We performed structural optimization of these chosen proteins using Chimera version 1.16 (San Francisco, CA, USA; https://www.cgl.ucsf.edu/chimera/download.html, accessed on 16 September 2024) to guarantee the best possible performance in our molecular docking tests [108]. For all compounds, Chimera 1.16’s (San Francisco, CA, USA) AMBER ff14SB option was utilized by default.

#### 3.4.3. Protein and Compound Docking

To perform molecular docking between compounds (**1**–**20**) and reference medicines against the H chain of the 16 targeted proteins, PyRx’s Vina Wizard was utilized. The outcomes were verified using the redocking technique. The following box parameters applied to the compounds and H chain: Center X: 32.2835, Y: −19.8358, Z: 55.6518, with box dimensions in Angstroms of X: 58.6977, Y: 105.2780, and Z: 87.5728 for topoisomerase II and Center X: 37.9146, Y: 65.4133, Z: 70.9087, with box dimensions in Angstroms of X: 76.2085, Y:116.5524, and Z: 123.0756 for LSD1. Compounds **16** and **15**, which exhibit minimal departure from RMSD (root-mean-square deviations) values (0) and significantly greater negative binding affinities against *Staphylococcus aureus* topoisomerase II and lysine-specific demethylase 1, respectively, were chosen for additional molecular dynamics simulation analysis.

UCSF Chimera version 1.16 (San Francisco, CA, USA) was then used to identify the amino acid residues of *Staphylococcus aureus* topoisomerase II and lysine-specific demethylase1 that were interacting with the compounds. PyMOL version 2.5 (New York, NY, USA; https://pymol.org/installers/, accessed on 27 September 2024) was used to create three-dimensional structures, which assisted in the creation of molecular docking images [109]. Three-dimensional visualization of the compound–protein docking was achieved with PyMOL 2.5.3 (New York, NY, USA) [109], BIOVIA Discovery Studio (San Diego, CA, USA) [110], and Chimera 1.16 (San Francisco, CA, USA) [108].

### 3.5. Molecular Dynamics Simulation

Molecular dynamics (MD) simulations were conducted using the GROMACS version 2021.6 (Groningen, The Netherlands) [111] package to investigate interactions between the protein–compound complexes with compounds **13**, **16**, **17** and gemcitabine and the vacant targeted DNA gyrase of S. aureus structure, and the protein–compound complexes with compounds **12**, **14**, **15** and fluconazole and LSD1 structure. The force field AMBER99SB [112], which offers extensive details on atomic interactions, was employed. MD simulations offer a level of precision and insightful understanding of multidimensional system dynamics that is sometimes not achievable through experimental methods [112]. Additional MD simulations were performed to corroborate previous findings, utilizing the H chain of 5CDQ for compounds **12**, **14**, **15**, and fluconazole, and 2Z3Y for compounds **13**, **16**, **17**, and gemcitabine. Protein topological characteristics were generated using Galaxy European Server, a widely used molecular modeling and simulation program [113]. The simulation parameters included the TIP3P water model, the addition of hydrogens to the compound at pH 7.4, the first removal of hydrogen atoms from the GROMACS configuration, the maintenance of the charged state of the molecules at 0 with a multiplicity of 1 during MD topology construction, and the incorporation of the gaff force field for parameterization. By combining protein and chemical files, a structural configuration of one nanometer was produced inside a triclinic box. After the system was solvated with SPC water molecules in a triclinic box, sodium and chloride ions were introduced to achieve standard salt concentrations and neutralize the system [114].

Position-restrained dynamics (NVT) at 300 K for 3000 ps, along with the leapfrog algorithm, ensured system stability during the equilibration process [114,115,116]. The system was operated in production for an extra 3000 ps at a constant pressure and temperature following equilibration. After that, the system was simulated for 100 ns at the same temperature and pressure. GROMACS utility tools, including gmx rmsd, gmx rmsf, gmx gyrate, gmx hbond, gmx gmx pe, gmx Coulomb-SR, and gmx LJ-SR were utilized to generate graphs that depicted the compounds’ RMSD, RMSF (root-mean-square fluctuations), and Rg (radius of gyration) during a 100 ns duration at 300 K. Using the Bio3D package on the GALAXY Europe server, principal component analysis (PCA) was used to assess the stability of protein complexes for compound–protein complexes at 300 K as well as compounds **15**-5CDQ and **16**-2Z3Y at temperature settings of 300, 305, 310, and 320 K [117,118,119]. Additionally, the compounds’ cosine content was investigated. Our knowledge of the fundamental principles governing the structure and function of biological macromolecules has increased as a result of these MD simulations, which offered insightful information about the behavior of protein–compound complexes.

## 4. Conclusions

In order to evaluate the potential of twenty distinct compounds as therapeutic agents that target lysine-specific demethylase 1 (LSD1) and topoisomerase II (topo II) in the treatment of cancer and infectious diseases, we conducted a complete computer analysis of each drug in this study. The inquiry made use of computational techniques such as DFT calculations, MD simulations, drug similarity assessments, molecular docking evaluations, binding force calculations, and HOMO and LUMO feature analysis. In silico experiments showed that compound **15** showed a similar binding affinity to topoisomerase II and compound **16** to lysine-specific demethylase 1 as they did in vitro, indicating potential therapeutic use. Compounds **15** and **16** also had comparable effects on topoisomerase II and lysine-specific demethylase1 cell lines to reference drugs such as ampicillin, cefatrizine, fluconazole, gemcitabine, itraconazole, ribavirin, rufinamide, streptomycin, and tazobactam. Additionally, each molecule met a number of drug-likeness criteria, and their low to moderate acute oral toxicity suggested that they may be taken orally. Both in vitro and in silico studies support the favorable interactions between compound **15** and topoisomerase II and compound **16** and lysine-specific demethylase 1. However, a number of limitations must be considered. First, the entire study rests upon computational predictions without any experimental validation of the biological activities of the compounds. Secondly, the DFT calculations were carried out in the gas phase without considering solvent effects, which could contribute to the value of dipole moment and reactivity parameters. Third, molecular docking and MD simulations provide useful information about binding interactions and stability, but they cannot imitate the full complexity of biological systems. Another limitation lies in the lack of direct correlation between theoretical parameters like FMO values and biological results, reducing the predictive capacity of electronic descriptors relied upon in this study. More in vivo studies are required to assess compound **15**’s efficacy, safety, and viability as a topoisomerase II inhibitor and compound **16**’s as a lysine-specific demethylase 1 inhibitor, as well as their potential as medicinal drugs for the treatment of cancer and infectious diseases.

## Figures and Tables

**Table 1:** Molecular docking simulation results for potential antimicrobial compounds (**1**–**20**) and reference drugs against six targets.

	Binding Affinity (kcal mol^−1^)					
Ligand	4KSG	5BS8	5CDQ	6RKU	6ZT3	8BN6
**1**	−8.1	−6.8	−6.3	−6.6	−6.4	−7.9
**2**	−7.8	−6.5	−6.6	−6.3	−8.1	−7.5
**3**	−7.7	−6.5	−9.2	−6.9	−8.9	−6.6
**4**	−7.3	−7.0	−7.1	−7.2	−7.4	−8.0
**5**	−8.2	−7.8	−7.1	−7.9	−8.5	−8.7
**6**	−8	−6.5	−8.6	−7.9	−7.6	−8.1
**7**	−7.2	−8.8	−7.5	−7.2	−7.4	−6.9
**8**	−7.9	−8.6	−7.6	−7.7	−8.8	−7.8
**9**	−7.7	−9.4	−8.2	−7.5	−6.9	−6.7
**10**	−8.2	−10.2	−9.1	−7.9	−9.2	−7.8
**11**	−7.5	−8.6	−9.1	−8.5	−8.6	−8.4
**12**	−8.7	−9.3	−9.8	−9.0	−8.1	−9.3
**13**	−8.8	−9.4	−9.3	−8.5	−7.8	−9.4
**14**	−8.9	−9.2	−10.1	−7.8	−7.7	−8.0
**15**	−9.2	−9.1	−10.6	−8.9	−8.1	−8.3
**16**	−8.5	−7.6	−9.1	−8.2	−8.4	−9.6
**17**	−9.1	−6.4	−7.9	−8.8	−8.7	−8.9
**18**	−9.0	−8.7	−8.8	−8.5	−7.1	−9.8
**19**	−7.7	−6.7	−5.8	−6.4	−7.6	−7.7
**20**	−8.0	−8.6	−9.2	−8.6	−7.7	−9.0
Ampicillin	−6.2	−6.9	−6.5	−6.6	−6.9	−6.9
Cefatrizine	−7.7	−7.1	−8.2	−7.3	−7.1	−8.0
Fluconazole	−7.0	−6.8	−7.2	−6.3	−5.8	−6.9
Gemcitabine	−6.2	−6.1	−6.6	−5.9	−5.3	−6.3
Itraconazole	−8.3	−8.1	−9.9	−9.0	−8.1	−8.2
Ribavirin	−6.5	−6.4	−6.1	−6.0	−6.5	−6.6
Rufinamide	−7.4	−6.8	−7.0	−6.5	−6.0	−6.6
Streptomycin	−6.8	−8.2	−7.4	−6.9	−6.3	−7.2
Tazobactam	−6.1	−6.7	−6.1	−6.1	−6.0	−5.7

**Table 2:** Molecular docking simulation results for potential anticancer compounds (**1**–**20**) and reference drugs against ten targets.

		Binding Affinity (kcal mol^−1^)								
Ligand	1M17	1MHL	1Y6A	2EUF	2J0T	2O21	2Z3Y	3PHD	3S79	4NRE
**1**	−7.0	−6.1	−6.8	−7.4	−7.1	−6.2	−7.5	−6.8	−7.1	−7.7
**2**	−6.7	−6.1	−6.5	−7.8	−6.8	−6.3	−6.3	−6.3	−7	−7.3
**3**	−7.1	−6.4	−6.9	−8.0	−7.5	−6.3	−6.4	−6.1	−7.9	−8.8
**4**	−7.8	−6.4	−6.9	−8.9	−7.4	−6.9	−7.5	−6.8	−8	−7.1
**5**	−8.1	−7.7	−8.3	−9.3	−8.2	−8.2	−9.6	−7.7	−9.1	−9.8
**6**	−7.9	−7.3	−8.0	−9.9	−7.9	−6.7	−7.3	−7.0	−8.7	−9.0
**7**	−7.8	−7.2	−7.5	−7.1	−8.6	−7.8	−9.0	−6.4	−8.4	−8.1
**8**	−8.4	−7.6	−7.8	−8.5	−8.7	−8.5	−9.4	−6.5	−8.9	−8.1
**9**	−7.5	−7.4	−7.9	−9.4	−7.9	−7.8	−7.8	−6.5	−8.2	−7.6
**10**	−9.7	−8.9	−8.8	−9.9	−9.8	−9.3	−9.5	−7.2	−9.1	−9.7
**11**	−9.9	−8.8	−8.8	−9.6	−9.3	−9.6	−10.9	−7.5	−9.5	−8.3
**12**	−9.9	−9.2	−8.7	−9.9	−10.1	−9.6	−10.6	−7.4	−9.3	−10.7
**13**	−10.1	−9.5	−8.9	−10.1	−9.4	−9.7	−11.4	−7.4	−9.8	−10.9
**14**	−9.8	−8.7	−8.7	−9.6	−9.3	−7.9	−9.9	−7.9	−8.9	−9.8
**15**	−9.7	−8.8	−7.9	−9.9	−8.8	−9.2	−10.9	−7.6	−8.4	−9.8
**16**	−9.2	−8.2	−8.3	−10.0	−10.2	−8.8	−12.0	−7.1	−10.4	−10.9
**17**	−8.9	−7.9	−8.1	−10.1	−10.0	−8.7	−11.2	−6.4	−8.7	−9.8
**18**	−10.0	−8.6	−9.1	−10.7	−8.9	−8.5	−11.8	−6.9	−8.3	−9.3
**19**	−6.9	−6.2	−6.5	−7.7	−7.1	−6.4	−6.5	−6.3	−6.6	−7.6
**20**	−9.5	−8.4	−7.2	−8.3	−8.8	−8.8	−8.4	−6.8	−9.6	−7.5
Ampicillin	−7.9	−6.5	−6.7	−8.2	−6.8	−6.9	−7.0	−6.6	−8	−7.7
Cefatrizine	−8.2	−7.1	−7.4	−8.3	−7.4	−6.9	−7.1	−6.6	−7.8	−8.1
Fluconazole	−7.3	−6.5	−6.6	−8.3	−6.8	−6.8	−7.6	−5.1	−7.1	−8.2
Gemcitabine	−6.6	−6.0	−5.7	−6.7	−5.9	−6.0	−6.5	−5.1	−7.6	−6.2
Itraconazole	−8.7	−7.4	−8.3	−8.8	−8.4	−9.0	−10.7	−7.4	−8.7	−8.6
Ribavirin	−6.3	−5.8	−5.8	−6.6	−6.9	−5.8	−6.9	−5.1	−6.4	−6.8
Rufinamide	−7.0	−5.8	−6.2	−7.9	−6.7	−6.6	−6.9	−7.0	−7.2	−7.5
Streptomycin	−6.4	−7.2	−6.1	−6.2	−7.3	−6.9	−7.7	−5.7	−7.2	−7.3
Tazobactam	−6.0	−5.8	−6.0	−5.7	−5.8	−5.6	−6.4	−5.3	−6.5	−6.5

**Table 3:** Compound–protein interacting amino acid residues of compound **15** against 5CDQ and compound **16** against 2Z3Y.

Complex	Interacting Residues	Distance (Å)	Type of Interaction	2D Diagram of Interaction
Compound **15**-Topo II	ALA A:118	4.88	Pi-Alkyl	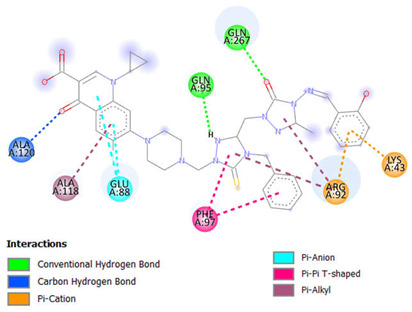
	ALA A:120	2.62	Carbon-Hydrogen Bond	
	ARG A:92	4.64	Pi-Cation	
		4.99, 5.37	Pi-Alkyl	
	GLN A:95	2.48	Conventional Hydrogen Bond	
	GLN A:267	2.31	Conventional Hydrogen Bond	
	GLU A:88	3.79, 3.88	Pi-Anion	
	LYS A:43	2.78	Pi-Cation; Pi-Donor Hydrogen Bond	
	PHE A:97	5.08, 5.78	Pi-Pi T-shaped	
Compound **16**-LSD1	ARG A:182	1.89, 2.58	Conventional Hydrogen Bond	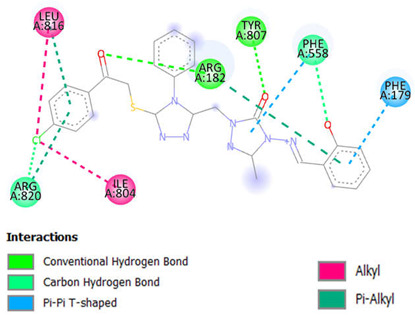
		4.19	Pi-Alkyl	
	ARG A:820	2.51	Carbon-Hydrogen Bond	
		3.73	Alkyl	
		5.19	Pi-Alkyl	
	ILE A:804	4.24	Alkyl	
	LEU A:816	4.36	Pi-Alkyl	
		4.99	Alkyl	
	PHE A:179	5.09	Pi-Pi T-shaped	
	PHE A:558	2.97	Carbon-Hydrogen Bond	
		4.95	Pi-Pi T-shaped	
	TYR A:807	2.62	Conventional Hydrogen Bond	

**Table 4:** Lipinski’s rule of five and Veber’s rule prediction for drug-likeness of potential antimicrobial and anticancer compounds (**1**–**20**) and reference drugs.

Ligand	MW	mLogP	nHBD	nHBA	Lipinski’s Violations	Veber’s Violations	TPSA Å^2^	nRotb	PAINS #Alerts	Brenk #Alerts
**Lipinski**	≤500	≤5	≤5	≤10	-	-	-	-	-	-
**Veber**	-	-	-	-	-	-	≤140	≤10	-	-
**1**	218.21	1.2	2	4	0	0	83.27	2	1	1
**2**	304.3	1.46	1	6	0	0	98.71	6	1	1
**3**	290.28	0.55	3	6	0	0	127.53	5	1	3
**4**	425.46	1.92	4	5	0	1	157.66	9	1	2
**5**	409.4	1.97	4	6	1	1	142.64	9	1	1
**6**	423.43	1.93	4	6	1	1	142.64	10	1	1
**7**	407.45	1.77	2	5	0	0	138.11	5	1	2
**8**	391.38	1.82	2	6	0	0	123.09	5	1	1
**9**	421.48	1.73	2	5	0	0	138.11	6	1	2
**10**	738.79	2.28	2	10	2	1	193.03	10	1	2
**11**	750.8	2.46	2	10	2	1	193.03	10	1	2
**12**	722.72	1.95	2	11	2	1	178.01	10	1	1
**13**	734.74	2.13	2	11	2	1	178.01	10	1	1
**14**	752.82	2.19	2	10	2	2	193.03	11	1	2
**15**	764.83	2.36	2	10	2	2	193.03	11	1	2
**16**	560.03	3.65	1	7	1	1	145.49	9	1	1
**17**	562.04	3.72	2	7	1	1	148.65	9	1	1
**18**	721.06	5.73	1	7	2	1	137.65	12	1	1
**19**	721.06	5.73	1	7	2	1	137.65	12	1	1
**20**	686.61	5.29	1	7	2	1	137.65	12	1	1
Ampicillin	349.4	0.75	3	5	0	0	138.03	5	0	0
Cefatrizine	462.5	0.02	5	8	1	1	225.13	8	0	0
Fluconazole	306.27	1.47	1	7	0	0	81.65	5	0	0
Gemcitabine	263.2	−1.2	3	7	0	0	110.6	2	0	0
Itraconazole	705.63	4.21	0	7	3	1	104.7	11	2	0
Ribavirin	244.2	−2.94	4	7	0	1	143.72	3	0	0
Rufinamide	238.19	1.44	1	5	0	0	73.8	3	0	0
Streptomycin	581.57	−5.96	12	15	3	1	336.43	9	0	3
Tazobactam	300.29	−0.44	1	7	0	0	130.84	3	0	0

**Table 5:** Drug-likeness evaluation of potential antimicrobial and anticancer compounds (**1**–**20**) and reference drugs.

Ligand	GPCR	ICM	KI	NRL	EI	PI
**1**	−1.28	−1.20	−1.07	−1.56	−1.54	−1.05
**2**	−0.79	−1.14	−0.98	−0.92	−0.85	−0.73
**3**	−1.02	−1.54	−1.08	−1.45	−0.98	−0.83
**4**	−0.95	−1.17	−1.03	−1.26	−0.86	−0.87
**5**	−0.60	−0.97	−0.66	−1.11	−0.62	−0.68
**6**	−0.51	−0.94	−0.73	−1.02	−0.48	−0.61
**7**	−0.76	−1.14	−1.10	−0.98	−0.96	−0.87
**8**	−0.52	−0.82	−0.69	−0.76	−0.75	−0.69
**9**	−0.72	−1.11	−1.04	−0.90	−0.88	−0.75
**10**	−1.49	−2.80	−2.24	−2.32	−1.51	−1.89
**11**	−1.68	−2.98	−2.47	−2.59	−1.60	−2.01
**12**	−1.35	−2.66	−2.08	−2.20	−1.38	−1.78
**13**	−1.53	−2.85	−2.30	−2.48	−1.47	−1.91
**14**	−1.63	−2.99	−2.41	−2.48	−1.59	−2.01
**15**	−1.82	−3.19	−2.65	−2.76	−1.69	−2.15
**16**	−0.71	−1.27	−0.94	−1.04	−0.79	−0.74
**17**	−0.59	−1.09	−0.86	−0.82	−0.73	−0.71
**18**	−0.89	−1.89	−1.44	−1.41	−0.81	−1.23
**19**	−0.86	−1.88	−1.44	−1.44	−0.81	−1.22
**20**	−0.80	−1.73	−1.31	−1.27	−0.72	−1.09
Ampicillin	0.04	−0.47	−0.71	−0.61	0.87	0.25
Cefatrizine	−0.23	−0.72	−0.91	−0.98	0.34	0.06
Fluconazole	0.04	0.01	−0.09	0.23	−0.09	0.03
Gemcitabine	0.58	0.11	0.33	−1.00	0.15	1.06
Itraconazole	−0.40	−1.50	−1.30	−1.31	−0.66	−0.97
Ribavirin	0.31	0.21	−0.21	−1.46	−0.20	0.71
Rufinamide	−0.19	−0.27	−0.16	−1.01	−0.48	−0.03
Streptomycin	0.09	−0.16	−0.17	−0.18	0.65	0.38
Tazobactam	0.18	−0.27	−0.29	−0.45	1.35	1.04

**Table 6:** In silico prediction of selected ADMET parameters for compounds (**1**–**20**) and reference drugs.

Ligand	^a^HIA	^a^BBB	^a^hERG_ pIC50	^a^CYP2C19 Inhibition	^a^CYP3A4 Inhibition	^a^Subcellular Localization	^b^SA Score
**1**	+1.0000	+0.9391	0.7734	0.8711	0.9465	M—0.7809	2.59
**2**	+0.9483	+0.8026	0.7528	0.6314	0.9628	M—0.7452	3.31
**3**	+0.9964	+0.9002	0.8610	0.8958	0.9421	M—0.8127	3.19
**4**	+0.9625	+0.6926	0.8919	0.7727	0.8210	M—0.7928	3.71
**5**	+0.9493	+0.6651	0.8382	0.7965	0.9006	M—0.8182	3.66
**6**	+0.9126	+0.6327	0.7789	0.7407	0.8156	M—0.7846	3.72
**7**	+0.9875	+0.8430	0.8850	0.6117	0.6447	M—0.7725	3.64
**8**	+0.9968	+0.8669	0.7863	0.7570	0.8270	M—0.9005	3.65
**9**	+0.9475	+0.7982	0.7144	0.6524	0.5131	M—0.7270	3.75
**10**	+0.9638	−0.8199	0.6779	0.7168	0.6288	M—0.4652	5.29
**11**	+0.9335	−0.7924	0.6345	0.7440	0.7540	M—0.5160	5.33
**12**	+0.9947	−0.7511	0.5613	0.7627	0.5000	M—0.5847	5.30
**13**	+0.9900	−0.7126	0.6078	0.8121	0.6447	M—0.6287	5.34
**14**	+0.9638	−0.8199	0.6779	0.7168	0.6288	M—0.4652	5.39
**15**	+0.9335	−0.7924	0.6345	0.7440	0.7540	M—0.5160	5.43
**16**	+0.9895	+0.7942	0.8315	0.5315	0.6792	M—0.7179	4.25
**17**	+0.9925	+0.6399	0.8949	0.5000	0.5979	M—0.7157	4.83
**18**	+0.9904	+0.8728	0.8437	0.6195	0.6509	M—0.7188	5.41
**19**	+0.9904	+0.8728	0.8437	0.6195	0.6509	M—0.7188	5.41
**20**	+0.9904	+0.8728	0.8437	0.6195	0.6509	M—0.7188	5.39
Ampicillin	−0.9270	−0.9961	0.9998	0.9399	0.8309	L—0.5707	4.16
Cefatrizine	−0.9522	−0.9833	0.9613	0.7493	0.7150	M—0.6074	4.87
Fluconazole	+0.9894	+0.9382	0.8229	0.5320	0.8196	M—0.8498	2.45
Gemcitabine	+0.9814	+0.9693	0.9948	0.8478	0.9032	N—0.4165	3.71
Itraconazole	+0.9973	−0.6151	0.5782	0.5703	0.5279	M—0.4776	5.77
Ribavirin	+0.9852	+0.9381	0.9948	0.9095	0.9535	M—0.4619	3.89
Rufinamide	+1.0000	+0.9777	0.9484	0.5561	0.7995	M—0.8591	2.22
Streptomycin	−0.8824	−0.9712	0.9934	0.9026	0.8867	M—0.4518	6.92
Tazobactam	−0.6161	−0.9659	0.9939	0.7143	0.8596	M—0.4356	4.23

^a^HIA: human intestinal absorption (%); ^a^BBB: blood-brain barrier penetration (%); ^a^hERG_pIC50: human ether-a-go-go-related gene inhibition (%); ^a^CYP2C19: cytochrome P4502C19; ^a^CYP3A4: Cytochrome P450 3A4; ^a^subcellular localization (%): M = mitochondria, N = nucleus, L = lysosome; ^b^SA score: synthetic accessibility score. ^a^ The values use admetSAR. ^b^ Synthetic accessibility score values use swissADME.

**Table 7:** A variability in principal components revealed via PCA for the target topoisomerase II-compound **15** complex and LSD1-compound **16** complex at different temperatures.

		Principal Components			
Complex	Temperature	PC1 (%)	PC2 (%)	PC3 (%)	Cosine Value
Topo II-compound **15**	300	48.79	15.73	8.27	0.09
	305	22.38	11.94	9.05	0.48
	310	37.53	20.59	8.12	0.70
	320	0.73	0.68	0.49	0.01
LSD1-compound **16**	300	23.75	21.31	10.58	0.83
	305	32.66	21.92	6.44	0.79
	310	15.21	13.04	10.04	0.78
	320	23.88	14.32	9.79	0.32

**Table 8:** Antimicrobial activity of the prepared compounds (**10**–**15**) (μg/mL) ^a^.

	Minimal Inhibition Concentration Values (μg/mL)								
Compound	Ec	Yp	Pa	Sa	Ef	Bc	Ms	Ca	Sc
**10**	<0.24	<0.24	<0.24	<0.24	<0.24	<0.24	<0.24	-	-
**11**	<0.24	<0.24	<0.24	<0.24	<0.24	<0.24	<0.24	-	-
**12**	<0.24	<0.24	<0.24	<0.24	<0.24	<0.24	<0.24	-	-
**13**	<0.24	<0.24	<0.24	<0.24	<0.24	<0.24	<0.24	-	-
**14**	<0.24	<0.24	<0.24	<0.24	<0.24	<0.24	<0.24	-	-
**15**	<0.24	<0.24	<0.24	<0.24	<0.24	<0.24	<0.24	-	-

^a^ Ref. [60]; (-): no activity resulting from the test concentrations; Ec: *E. coli* ATCC 35218; Yp: *Y. pseudotuberculosis* ATCC 911; Pa: *P. aeruginosa* ATCC 10145; Sa: *S. aureus* ATCC 25923; Ef: *E. faecalis* ATCC 29212; Bc: *B. cereus* 709 Roma; Ms: *M. smegmatis* ATCC607; Ca: *C. albicans* ATCC 60193; Sc: *S. cerevisiae* RSKK 251.

## Data Availability

Here is a list of software and websites, including AdmetSAR, available at http://lmmd.ecust.edu.cn/admetsar1/ (accessed on 14 September 2024), SwissADME accessible at http://www.swissadme.ch/ (accessed on 14 September 2024), Pass prediction found at http://www.way2drug.com/passonline/ (accessed on 15 September 2024), as well as coordinates of stable local minimum structures included in the Supporting Information. All data generated or analyzed during this study are included in the Supplementary Information SI.

## Supplementary Materials

The following supporting information can be downloaded at: https://www.mdpi.com/article/10.3390/ijms26146752/s1.

## References

[1] Kakkar S., Kumar S., Lim S.M., Ramasamy K., Mani V., Shah S.A.A., Narasimhan B. (2018). Design, synthesis and biological evaluation of 3-(2-aminooxazol-5-yl)-2H-chromen-2-one derivatives. BMC Chem..

[2] Ma X., Yu H. (2006). Cancer issue: Global burden of cancer. Yale J. Biol. Med..

[3] Nesaragi A.R., Kamble R.R., Bayannavar P.K., Shaikh S.K.J., Hoolageri S.R., Kodasi B., Joshi S.D., Kumbar V.M. (2021). Microwave assisted regioselective synthesis of quinoline appended triazoles as potent anti-tubercular and antifungal agents via copper catalyzed cycloaddition. Bioorgan. Med. Chem. Lett..

[4] Ueda S., Nagasawa H. (2009). Facile synthesis of 1, 2, 4-triazoles via a copper-catalyzed tandem addition oxidative cyclization. J. Am. Chem. Soc..

[5] Kaplancikli Z.A., Turan-Zitouni G., Ozdemir A., Revial G. (2008). New triazole and triazolothiadiazine derivatives as possible antimicrobial agents. Eur. J. Med. Chem..

[6] Nesaragi A.R., Kamble R.R., Bayannavar P.K., Metre T.V., Kariduraganavar M.Y., Margankop S.B., Joshi S.D., Kumbar V.M. (2021). Microwave facilitated one-pot three component triazoles: Antimicrobial evaluation, molecular docking and in silico ADME studie. Synth. Commun..

[7] Kavaklı C., Kavaklı P.A., Güven O. (2014). Preparation and characterization of glycidyl methacrylate grafted 4-amino-1, 2, 4-triazole modified nonwoven fiber adsorbent for environmental application. Radiat. Phys. Chem..

[8] Zhou C.H., Wang Y. (2012). Recent researches in triazole compounds as medicinal drugs. Curr. Med. Chem..

[9] Fang B., Zhou C.H., Rao X.C. (2010). Synthesis and biological activities of novel amine-derived bis-azoles as potential antibacterial and antifungal agents. Eur. J. Med. Chem..

[10] Nesaragi A.R., Algethami J.S., Alsaiari M., Alsareii S.A., Mathada B.S., Ningaiah S., Sasidhar B.S., Harraz F.A., Patil S.A. (2024). A comprehensive overview of coumarinyl-triazole hybrids as anticancer agents. J. Mol. Struc..

[11] Saeed A., Shaheen U., Hameed A., Kazmi F. (2010). Synthesis and antimicrobial activity of some novel 2-(substituted fluorobenzoylimino)-3-(substituted fluorophenyl)-4- methyl-1,3-thiazolines. J. Fluor. Chem..

[12] Lemilemu F., Bitew M., Demissie T.B., Eswaramoorthy R., Endale M. (2021). Synthesis, antibacterial and antioxidant activities of Thiazole-based Schiff base derivatives: A combined experimental and computational study. BMC Chem..

[13] Yurttaş L., Özkay Y., Karaca H., Çevik U.A. (2015). Synthesis of some new thiazole derivatives and their biological activity evaluation. J. Chem..

[14] Morsy M.A., Ali E.M., Kandeel M., Venugopala K.N., Nair A.B., Greish K., El-Daly M. (2020). Screening and molecular docking of novel benzothiazole derivatives as potential antimicrobial agents. Antibiotics.

[15] Kumar R., Gahlyan P., Verma A., Jain R., Das S., Konwar R., Prasad A.K. (2018). Design and synthesis of fluorescent symmetric bis-triazolylated-1,4-dihydropyridines as potent antibreast cancer agents. Synth. Commun..

[16] Kumbhare R.M., Kosurkar U.B., Bagul P.K., Kanwal A., Appalanaidu K., Dadmal T.L., Banerjee S.K. (2014). Synthesis and evaluation of novel triazoles and mannich bases functionalized 1,4-dihydropyridine as angiotensin converting enzyme (ACE) inhibitors. Bioorgan. Med. Chem..

[17] Singh H., Sindhu J., Khurana J.M., Sharma C., Aneja K.R. (2013). A facile eco-friendly one-pot five-component synthesis of novel 1,2,3-triazole-linked pentasubstituted 1,4- dihydropyridines and their biological and photophysical studies. Aust. J. Chem..

[18] Vijesh A.M., Isloor A.M., Peethambar S.K., Shivananda K.N., Arulmoli T., Isloor N.A. (2011). Hantzsch reaction: Synthesis and characterization of some new 1,4- dihydropyridine derivatives as potent antimicrobial and antioxidant agents. Eur. J. Med. Chem..

[19] Khan M.A., Kola V.B., Noor B., Acco J. (2020). The antioxidant activity of dihydropyridine derivatives. Curr. Res. Bioorgan. Org. Chem..

[20] da Costa Cabrera D., Santa-Helena E., Leal H.P., de Moura R.R., Nery L.E.M., Goncalves C.A.N., Russowsky D., D’Oca M.G.M. (2019). Synthesis and antioxidant activity of new lipophilic dihydropyridines. Bioorgan. Chem..

[21] Raju R., Rajasekar S., Raghunathan R., Arumugam N., Almansour A.I., Kumar R.S. (2020). Regioselective synthesis and antioxidant activity of a novel class of mono and C2- symmetric bis-1,2,3-triazole and acridinedione grafted macromolecules. J. Saudi Chem. Soc..

[22] Hadjipavlou-Litina D., Głowacka I.E., Marco-Contelles J., Piotrowska D.G. (2023). Synthesis and antioxidant properties of novel 1,2,3-triazole-containing nitrones. Antioxidants.

[23] Khare S.P., Deshmukh T.R., Sangshetti J.N., Krishna V.S., Sriram D., Khedkar V.M., Shingate B.B. (2018). Design, synthesis and molecular docking studies of novel triazole-chromene conjugates as antitubercular, antioxidant and antifungal agents. ChemistrySelect.

[24] Khare S.P., Deshmukh T.R., Akolkar S.V., Sangshetti J.N., Khedkar V.M., Shingate B.B. (2019). New 1,2,3-triazole-linked tetrahydrobenzo[*b*]pyran derivatives: Facile synthesis, biological evaluation and molecular docking study. Res. Chem. Intermed..

[25] Khare S.P., Deshmukh T.R., Sangshetti J.N., Khedkar V.M., Shingate B.B. (2019). Ultrasound assisted rapid synthesis, biological evaluation, and molecular docking study of new 1,2,3-triazolyl pyrano[2,3-*c*]pyrazoles as antifungal and antioxidant agent. Synth. Commun..

[26] Danne A.B., Lathi K.V., Sangshetti J.N., Khedkar V.M., Khalse L.D., Shingate B.B. (2023). New 1,2,3-Triazole Tethered-1,4-Dihydropyridines as Potential Antioxidant Agents: Synthesis and Molecular Docking Study. J. Mol. Struct..

[27] Chen G.L., Guo L., Yang S., Ji D.M. (2021). Cancer risk in tuberculosis patients in a high endemic area. BMC Cancer.

[28] Ho L.J., Yang H.Y., Chung C.H., Chang W.C., Yang S.S., Sun C.A., Chien W.C., Su R.Y., Subbiah S.K. (2021). Increased risk of secondary lung cancer in patients with tuberculosis: A nationwide, population-based cohort study. PLoS ONE.

[29] Luczynski P., Poulin P., Romanowski K., Johnston J.C., Duell E.J. (2022). Tuberculosis and risk of cancer: A systematic review and meta-analysis. PLoS ONE.

[30] Apte R.S., Chen D.S., Ferrara N. (2019). VEGF in Signaling and Disease: Beyond Discovery and Development. Cell.

[31] Saghazadeh A., Rezaei N., Rottenberg M.E. (2022). Vascular endothelial growth factor levels in tuberculosis: A systematic review and meta-analysis. PLoS ONE.

[32] Hoolageri S.R., Kamble R.R., Nesaragi A.R., Bheemayya L., Nadoni V.B., Dixit S., Vootla S., Joshi S.D. (2022). Cu (I) catalyzed A3 cascade coupling via C-H functionalization followed by cyclization: Synthesis, in silico, in vitro, and toxicity studies of imidazo[2,1-*b*]thiazoles. Appl. Organomet. Chem..

[33] Sashidhara K.V., Kumar A., Chatterjee M., Rao K.B., Singh S., Verma A.K., Palit G. (2011). Discovery and synthesis of novel 3-phenylcoumarin derivatives as antidepressant agents. Bioorgan. Med. Chem. Lett..

[34] Nargotra A., Sharma S., Alam M.I., Ahmed Z., Bhagat A., Taneja S.C., Qazi G.N., Koul S. (2011). In silico identification of viper phospholipaseA2 inhibitors: Validation by in vitro, in vivo studies. J. Mol. Model..

[35] Alipour M., Khoobi M., Moradi A., Nadri H., Moghadam F.H., Emami S., Hasanpour Z., Foroumadi A., Shafiee A. (2014). Synthesis and anti-cholinesterase activity of new 7-hydroxycoumarin derivatives. Eur. J. Med. Chem..

[36] Tripathi V.K., Singh J., Ara T., Koul S., Farooq S., Kaul A. (2014). Synthesis and biological evaluation of novel isoxazoles and triazoles linked 6-hydroxycoumarin as potent cytotoxic agents. Bioorgan. Med. Chem. Lett..

[37] Thigulla Y., Kumar T.U., Trivedi P., Ghosh B., Bhattacharya A. (2017). One-step synthesis of fused chromeno[4,3-b]pyrrolo[3,2-h]quinolin-7(1H)-one compounds and their anticancer activity evaluation. ChemistrySelect.

[38] Peyressatre M., Prével C., Pellerano M., Morris M. (2015). Targeting cyclin-dependent kinases in human cancers: From small molecules to Peptide inhibitors. Cancers.

[39] Smalley K.S.M., Contractor R., Nguyen T.K., Xiao M., Edwards R., Muthusamy V., King A.J., Flaherty K.T., Bosenberg M., Herlyn M. (2008). Identification of a novel subgroup of melanomas with KIT/cyclin-dependent kinase-4 overexpression. Cancer Res..

[40] Dobashi Y., Goto A., Fukayama M., Abe A., Ooi A. (2004). Overexpression of cdk4/cyclin D1, a possible mediator of apoptosis and an indicator of prognosis in human primary lung carcinoma. Int. J. Cancer.

[41] Graf F., Mosch B., Koehler L., Bergmann R., Wuest F., Pietzsch J. (2010). Cyclin-dependent kinase 4/6 (cdk4/6) inhibitors: Perspectives in cancer therapy and imaging. Mini Rev. Med. Chem..

[42] Ribnikar D., Volovat S.R., Cardoso F. (2019). Targeting CDK4/6 pathways and beyond in breast cancer. Breast.

[43] Farghaly T.A., Pashameah R.A., Bayazeed A., Al-Soliemy A.M., Alsaedi A.M.R., Harras M.F. (2024). Design and Synthesis of New *bis*-oxindole and Spiro(triazole-oxindole) as CDK4 Inhibitors with Potent Anti-breast Cancer Activity. Med. Chem..

[44] Chamduang C., Pingaew R., Prachayasittikul V., Prachayasittikul S., Ruchirawat S., Prachayasittikul V. (2019). Novel triazole-tetrahydroisoquinoline hybrids as human aromatase inhibitors. Bioorgan. Chem..

[45] Doiron J., Richard R., Touré M.M., Picot N., Richard R., Čuperlović-Culf M., Robichaud G.A., Touaibia M. (2011). Synthesis and structure–activity relationship of 1-and 2-substituted-1, 2, 3-triazole letrozole-based analogues as aromatase inhibitors. Eur. J. Med. Chem..

[46] Henneberta O., Montes M., Favre-Reguillon A., Chermetted H., Ferroudc C., Mortin R. (2009). Epimerase activity of the human 11β-hydroxysteroid dehydrogenase type 1 on 7-hydroxylated C19-steroids. J. Steroid Biochem. Mol. Biol..

[47] Leechaisit R., Pingaew R., Prachayasittikul V., Worachartcheewan A., Prachayasittikul S., Ruchirawat S., Prachayasittikul V. (2019). Synthesis, molecular docking, and QSAR study of bis-sulfonamide derivatives as potential aromatase inhibitors. Bioorgan. Med. Chem..

[48] Brueggemeier R.W., Hackett J.C., Diaz-Cruz E.S. (2005). Aromatase inhibitors in the treatment of breast cancer. Endocr. Rev..

[49] Cepa M.M., da Silva E.J.T., Correia-da-Silva G., Roleira F.M., Teixeira N.A. (2008). Synthesis and biochemical studies of 17-substituted androst-3-enes and 3,4-epoxyandrostanes as aromatase inhibitors. Steroids.

[50] Çevik U.A., Sağlık B.N., Osmaniye D., Levent S., Çavuşoğlu B.K., Karaduman A.B., Ozkay Y., Kaplancıklı Z.A. (2020). Synthesis and docking study of benzimidazole–triazolothiadiazine hybrids as aromatase inhibitors. Arch. Pharm..

[51] Çevik U.A., Çavuşoğlu B.K., Sağlık B.N., Osmaniye D., Levent S., Ilgın S., Özkay Y., Kaplancıklı Z.A. (2020). Synthesis, Docking Studies and Biological Activity of New Benzimidazole- Triazolothiadiazine Derivatives as Aromatase Inhibitor. Molecules.

[52] Sahoo R., Babu V.C., Okaly G.V.P., Rao S., Nargund A., Venkataswamy E., Rao R., Kumar B.S. (2011). Screening for EGFR mutations in lung cancer, a report from India. Lung Cancer.

[53] Sigismund S., Avanzato D., Lanzetti L. (2018). Emerging functions of the EGFR in cancer. Mol. Oncol..

[54] Gariganti N., Loke S.K., Pagadala E., Chinta P., Poola B., Chetti P., Bansal A., Ramachandran B., Srinivasadesikan V., Kottalanka R.K. (2023). Design, synthesis, anticancer activity of new amide derivatives derived from 1,2,3-triazole-benzofuran hybrids: An insights from molecular docking, molecular dynamics simulation and DFT studies. J. Mol. Struct..

[55] Liang T., Sun X., Li W., Hou G., Gao F. (2021). 1,2,3-triazole-containing compounds as anti-lung cancer agents: Current developments, mechanisms of action, and structure-activity relationship. Front. Pharmacol..

[56] Othman E.M., Fayed E.A., Husseiny E.M., Abulkhair H.S. (2022). Rationale design, synthesis, cytotoxicity evaluation, and in silico mechanistic studies of novel 1,2,3-triazoles with potential anticancer activity. New J. Chem..

[57] Özdemir A., Sever B., Altıntop M.D., Temel H.E., Atlı Ö., Baysal M., Demirci F. (2017). Synthesis and Evaluation of New Oxadiazole, Thiadiazole, and Triazole Derivatives as Potential Anticancer Agents Targeting MMP-9. Molecules.

[58] Hamdy R., Jones A.T., El-Sadek M., Hamoda A.M., Shakartalla S.B., ALShareef Z.M., Soliman S.S.M., Westwell A.D. (2021). New Bioactive Fused Triazolothiadiazoles as Bcl-2-Targeted Anticancer Agents. Int. J. Mol. Sci..

[59] Zheng Y.C., Duan Y.C., Ma J.L., Xu R.M., Zi X., Lv W.L., Wang M.M., Ye X.W., Zhu S., Mobley D. (2013). Triazole-dithiocarbamate based selective lysine specific demethylase 1 (LSD1) inactivators inhibit gastric cancer cell growth, invasion, and migration. J. Med. Chem..

[60] Mou Z., Gao J., Miao H., Zhang L., Su L., Wang B., Luan Y. (2019). Design and synthesis of novel histone deacetylase 6 inhibitors with benzyl-triazole as the core skeleton. Biosci. Trends.

[61] Esha N.J.I., Quayum S.T., Saif M.Z., Almatarneh M.H., Rahman S., Alodhayb A., Poirier R.A., Uddin K.M. (2024). Exploring the potential of fluoro-flavonoid derivatives as anti-lung cancer agents: DFT, molecular docking, and molecular dynamics techniques. Int. J. Quantum Chem..

[62] Cebeci Y.U., Ceylan S., Karaoglu S.A., Altun M. (2023). An efficient microwave-assisted synthesis of novel quinolone–triazole and conazole–triazole hybrid derivatives as antimicrobial and anticancer agents. J. Heterocycl. Chem..

[63] Lipinski C.A., Lombardo F., Dominy B.W., Feeney P.J. (2001). Experimental and Computational Approaches to Estimate Solubility and Permeability in Drug Discovery and Development Settings. Adv. Drug Deliv. Rev..

[64] Veber D.F., Johnson S.R., Cheng H.-Y., Smith B.R., Ward K.W., Kopple K.D. (2002). Molecular Properties That Influence the Oral Bioavailability of Drug Candidates. J. Med. Chem..

[65] Khan T., Dixit S., Ahmad R., Raza S., Azad I., Joshi S., Khan A.R. (2017). Molecular docking, PASS analysis, bioactivity score prediction, synthesis, characterization and biological activity evaluation of a functionalized 2-butanone thiosemicarbazone ligand and its complexes. J. Chem. Biol..

[66] Fukunishi Y., Nakamura H. (2011). Definition of Drug-Likeness for Compound Affinity. J. Chem. Inf. Model..

[67] Gurung A.B., Bhattacharjee A., Ali M.A. (2016). Exploring the Physicochemical Profile and the Binding Patterns of Selected Novel Anticancer Himalayan Plant Derived Active Compounds with Macromolecular Targets. Inform. Med. Unlocked.

[68] Shadrack D.M., Ndesendo V.M.K. (2017). Molecular Docking and ADMET Study of Emodin Derivatives as Anticancer Inhibitors of NAT2, COX2 and TOP1 Enzymes. Comput. Mol. Biosci..

[69] Ozkurt T.E., Akgul T., Baykut S. Principal Component Analysis of the Fractional Brownian Motion For 0 < H < 0.5. Proceedings of the 2006 IEEE International Conference on Acoustics Speech and Signal Processing Proceedings.

[70] Frisch M.J., Trucks G.W., Schlegel H.B., Scuseria G.E., Robb M.A., Cheeseman J.R., Scalmani G., Barone V., Mennucci B., Petersson G.A. (2016). Gaussian 16, Revision C.01.

[71] Uddin K.M., Alrawashdeh A.I., Henry D.J., Warburton P.L., Poirier R.A. (2019). Hydrolytic Deamination Reactions of Amidine and Nucleobase Derivatives. Int. J. Quantum Chem..

[72] Uddin K.M., Henry D.J., Alrawashdeh A.I., Warburton P.L., Poirier R.A. (2017). Mechanism for the deamination of ammeline, guanine, and their analogues. Struct. Chem..

[73] Uddin K.M., Almatarneh M.H., Shaw D.M., Poirier R.A. (2011). Mechanistic study of the deamination reaction of guanine: A computational study. J. Phys. Chem. A.

[74] Uddin K.M., Poirier R.A. (2011). Computational study of the deamination of 8-oxoguanine. J. Phys. Chem. B.

[75] Uddin K.M., Flinn C.G., Poirier R.A., Warburton P.L. (2014). Comparative computational investigation of the reaction mechanism for the hydrolytic deamination of cytosine, cytosine butane dimer and 5, 6-saturated cytosine analogues. Comput. Theor. Chem..

[76] Alrawashdeh A.I., Almatarneh M.H., Poirier R.A. (2013). Computational study on the deamination reaction of adenine with OH^−^/nH_2_O (n = 0, 1, 2, 3) and 3H_2_O. Can. J. Chem..

[77] Almatarneh M.H., Flinn C.G., Poirier R.A., Sokalski W.A. (2006). Computational Study of the Deamination Reaction of Cytosine with H_2_O and OH^−^. J. Phys. Chem. A.

[78] Uddin K.M., Hosen M.A., Khan M.F., Ozeki Y., Kawsar S.M.A. (2022). Investigation of Structural, Physicochemical, Pharmacokinetics, PASS Prediction, and Molecular Docking Analysis of Methyl 6-O-Myristoyl-α-D-Glucopyranoside Derivatives against SARS-CoV-2. Philipp. J. Sci..

[79] Chamizo J.A., Morgado J., Sosa P. (1993). Organometallic Aromaticity. Organometallics.

[80] Glasstone S., Laidler K.J., Eyring H. (1941). The Theory of Rate Processes: The Kinetics of Chemical Reactions, Viscosity, Diffusion and Electrochemical Phenomena.

[81] Alberty R.A. (1960). The Foundations of Chemical Kinetics. J. Chem. Educ..

[82] Parr R.G., von Szentpály L., Liu S. (1999). Electrophilicity Index. J. Am. Chem. Soc..

[83] Pal R., Chattaraj P.K. (2023). Electrophilicity Index Revisited. J. Comput. Chem..

[84] Daina A., Michielin O., Zoete V. (2017). SwissADME: A Free Web Tool to Evaluate Pharmacokinetics, Drug-Likeness and Medicinal Chemistry Friendliness of Small Molecules. Sci. Rep..

[85] Yang H., Lou C., Sun L., Li J., Cai Y., Wang Z., Li W., Liu G., Tang Y. (2019). AdmetSAR 2.0: Web-Service for Prediction and Optimization of Chemical Admet Properties. Bioinformatics.

[86] Druzhilovskiy D.S., Rudik A.V., Filimonov D.A., Gloriozova T.A., Lagunin A.A., Dmitriev A.V., Pogodin P.V., Dubovskaya V.I., Ivanov S.M., Tarasova O.A. (2017). Computational Platform Way2Drug: From the Prediction of Biological Activity to Drug Repurposing. Russ. Chem. Bull..

[87] Zardecki C., Dutta S., Goodsell D.S., Voigt M., Burley S.K. (2016). RCSB Protein Data Bank: A Resource for Chemical, Biochemical, and Structural Explorations of Large and Small Biomolecules. J. Chem. Educ..

[88] Dallakyan S., Olson A.J. (2014). Small-Molecule Library Screening by Docking with PyRx. Methods Mol. Biol..

[89] Varadi M., Anyango S., Deshpande M., Nair S., Natassia C., Yordanova G., Yuan D., Stroe O., Wood G., Laydon A. (2022). AlphaFold Protein Structure Database: Massively expanding the structural coverage of protein-sequence space with high-accuracy models. Nucleic Acids Res..

[90] Adobe Systems Inc (1985). Postscript Language Reference Manual.

[91] Allen F.H., Bellard S., Brice M.D., Cartwright B.A., Doubleday A., Higgs H., Hummelink T., Hummelink-Peters B.G., Kennard O., Motherwell W.D.S. (1979). The Cambridge Crystallographic Data Centre: Computer-Based Search, Retrieval, Analysis and Display of Information. Acta Cryst..

[92] Bernstein F.C., Koetzle T.F., Williams G.J.B., Meyer E.F., Brice M.D., Rogers J.R., Kennard O., Shimanouchi T., Tasumi M. (1977). The Protein Data Bank: A Computer-Based Archival File for Macromolecular Structures. J. Mol. Biol..

[93] Engh R.A., Huber R. (1991). Accurate Bond and Angle Parameters for X-Ray Protein Structure Refinement. Acta Cryst..

[94] (1970). IUPAC-IUB Commission on Biochemical Nomenclature Abbreviations and Symbols for the Description of the Conformation of Polypeptide Chains. J. Mol. Biol..

[95] Laskowski R.A., Rullmannn J.A., MacArthur M.W., Kaptein R., Thornton J.M. (1996). AQUA and PROCHECK-NMR: Programs for checking the quality of protein structures solved by NMR. J. Biomol. NMR.

[96] Laskowski R.A., Macarthur M.W., Moss D.S., Thornton J.M. (1993). PROCHECK: A Program to Check the Stereochemical Quality of Protein Structures. J. Appl. Cryst..

[97] Morris A.L., Macarthur M.W., Hutchinson E.G., Thornton J.M. (1992). Stereochemical Quality of Protein Structure Coordinates. Proteins.

[98] Nishikawa K., Ooi T. (1986). Radial Locations of Amino-Acid Residues in A Globular Protein: Correlation with the Sequence. J. Biochem..

[99] Colovos C., Yeates T.O. (1993). Verification of Protein Structures: Patterns of Nonbonded Atomic Interactions. Protein Sci..

[100] Bowie J.U., Lüthy R., Eisenberg D. (1991). A Method to Identify Protein Sequences That Fold into A Known Three-Dimensional Structure. Science.

[101] Lüthy R., Bowie J.U., Eisenberg D. (1992). Assessment of Protein Models with Three-Dimensional Profiles. Nature.

[102] Touw W.G., Baakman C., Black J., te Beek T.A., Krieger E., Joosten R.P., Vriend G. (2015). A Series of Pdb Related Databanks for Everyday Needs. Nucleic Acids Res..

[103] Kabsch W., Sander C. (1983). Dictionary of Protein Secondary Structure: Pattern Recognition of Hydrogen-Bonded and Geometrical Features. Biopolymers.

[104] Wikipedia Contributors (2024). DSSP (Algorithm).

[105] Sippl M.J. (1993). Recognition of Errors in Three-Dimensional Structures of Proteins. Proteins.

[106] Wiederstein M., Sippl M.J. (2007). ProSA-web: Interactive web service for the recognition of errors in three-dimensional structures of proteins. Nucleic Acids Res..

[107] Mering C.V., Huynen M., Jaeggi D., Schmidt S., Bork P., Snel B. (2003). STRING: A database of predicted functional associations between proteins. Nucleic Acids Res..

[108] Goddard T.D., Huang C.C., Ferrin T.E. (2007). Visualizing Density Maps with UCSF Chimera. J. Struct. Biol..

[109] Yuan S., Chan H.C.S., Hu Z. (2017). Using PyMOL as a Platform for Computational Drug Design. WIREs Comput. Mol. Sci..

[110] Baroroh U., Biotek M., Muscifa Z.S., Destiarani W., Rohmatullah F.G., Yusuf M. (2023). Molecular Interaction Analysis and Visualization of Protein-Compound Docking Using Biovia Discovery Studio Visualizer. Indones. J. Comput. Biol. (IJCB).

[111] Van Der Spoel D., Lindahl E., Hess B., Groenhof G., Mark A.E., Berendsen H.J.C. (2005). Gromacs: Fast, Flexible, and Free. J. Comput. Chem..

[112] Showalter S.A., Brüschweiler R. (2007). Validation of Molecular Dynamics Simulations of Biomolecules Using NMR Spin Relaxation as Benchmarks:  Application to the AMBER99SB Force Field. J. Chem. Theory Comput..

[113] Bray S.A., Lucas X., Kumar A., Grüning B.A. (2020). The Chemicaltoolbox: Reproducible, User-Friendly Cheminformatics Analysis on the Galaxy Platform. J. Cheminf..

[114] Cuendet M.A., van Gunsteren W.F. (2007). On the Calculation of Velocity-Dependent Properties in Molecular Dynamics Simulations Using the Leapfrog Integration Algorithm. J. Chem. Phys..

[115] Uddin K.M., Sakib M., Siraji S., Uddin R., Rahman S., Alodhayb A., Alibrahim K.A., Kumer A., Matin M.M., Bhuiyan M.M. (2023). Synthesis of New Derivatives of Benzylidinemalononitrile and Ethyl 2-Cyano-3-phenylacrylate: In Silico Anticancer Evaluation. ACS Omega.

[116] Quayum S.T., Esha N.J.I., Siraji S., Abbad S.S.A., Alsunaidi Z.H.A., Almatarneh M.H., Rahman S., Alodhayb A.N., Alibrahim K.A., Kawsar S.M.A. (2024). Exploring the effectiveness of flavone derivatives for treating liver diseases: Utilizing DFT, molecular docking, and molecular dynamics techniques. MethodsX.

[117] Afgan E., Baker D., Batut B., van den Beek M., Bouvier D., Cech M., Chilton J., Clements D., Coraor N., Grüning B.A. (2018). The Galaxy Platform for Accessible, Reproducible and Collaborative Biomedical Analyses: 2018 Update. Nucleic Acids Res..

[118] Grant B.J., Rodrigues A.P., ElSawy K.M., McCammon J.A., Caves L.S. (2006). Bio3D: An R Package for the Comparative Analysis of Protein Structures. Bioinformatics.

[119] Kumar N., Awasthi A., Kumari A., Sood D., Jain P., Singh T., Sharma N., Grover A., Chandra R. (2022). Antitussive Noscapine and Antiviral Drug Conjugates as Arsenal Against COVID-19: A Comprehensive Chemoinformatics Analysis. J. Biomol. Struct. Dyn..

