# Supplementary material for: In Silico Evaluation of Quinolone–Triazole and Conazole–Triazole Hybrids as Promising Antimicrobial and Anticancer Agents
Source: Int J Mol Sci. 2025 Jul 14;26(14):6752. doi: 10.3390/ijms26146752 (PMC12294901; doi:10.3390/ijms26146752)
Supplement: Supplementary file 1 [file ijms-26-06752-s001.zip › ijms-3678157-supplementary.pdf]

Supporting Information

In Silico Evaluation of Quinolone–Triazole and Conazole–Triazole Hybrids as Promising Antimicrobial and Anticancer Agents

Humaera Noor Suha [1], Mansour H. Almatarneh [2,*], Raymond A. Poirier [3] and Kabir M. Uddin [1,*]

[1] Department of Biochemistry and Microbiology, North South University, Dhaka 1229, Bangladesh; mohammed.uddin11@northsouth.edu
[2] Department of Chemistry, College of Science, Imam Mohammad Ibn Saud Islamic University (IMSIU), Riyadh 11623, Saudi Arabia
[3] Department of Chemistry, Memorial University, St. John's, NL A1B 3X7, Canada; rpoirier@mun.ca
* Correspondence: mhalmatarneh@imamu.edu.sa (M.H.A.); kabirmuddin@gmail.com (K.M.U.); Tel.: +88-017-9658-5904 (K.M.U.); Fax: +88-02-5566-8202 (K.M.U.)

S.1. Frontier Molecular Orbital Analysis

Examining the FMOs of quinolone-triazole and conazole-triazole hybrid derivatives can reveal important information about their stability and reactivity as possible antibacterial and anticancer drugs (1–20) [96]. The energy gap (Egap) between the HOMO and LUMO is crucial for regulating chemical reactivity, softness, hardness, electrophilicity, and chemical potential [96]. A big Egap indicates good stability but constrained chemical reactivity, whereas a small Egap indicates softness, which results in reduced stability but greater reactivity. Comprehending the energy scales associated with the HOMO and LUMO is a necessary step towards understanding the electron-donating and electron-accepting properties of the molecules. Some compounds have a higher energy gap while some of the compounds have a comparatively smaller energy gap. Compounds **1** and **2** with Egaps of 4.300 eV and 4.245 eV, respectively display a higher energy gap meanwhile compounds **19** and **20** showed smaller energy gaps at 2.177 eV and 2.748 eV, respectively. The Egap values vary between substances, ranging from 2.918 eV to 6.488 eV. Compounds with a wider gap are more stable but less reactive, whereas those with a narrower gap are more reactive but less stable. The Egap for the compounds is in the following ascending order: **19** (2.177 eV) < **20** (2.784 eV) < **6** (3.184 eV) < **5** (3.32 eV) < **11** (3.429 eV) < **13** (3.510 eV) < **15** (3.510 eV) < **14** (3.565 eV) < **12** (3.592 eV) < **7** (3.701 eV) < **10** (3.701 eV) < **9** (3.728 eV) < **4** (3.973 eV) < **18** (4.000 eV) < **17** (4.054 eV) < 16 (4.109 eV) < **8** (4.191 eV) < **3** (4.218 eV) < **2** (4.245 eV) < **1** (4.300 eV). The ascending order of the Egap for the reference drugs is as follows: itraconazole (3.918 eV) < cefatrizine (4.218 eV) < streptomycin (4.844 eV) < gemcitabine (5.307 eV) < rufinamide (5.579 eV) < ampicillin (5.769 eV) < ribavirin (5.878 eV) < fluconazole (6.286 eV) < tazobactam (6.341 eV) as shown in Table S1.

Table S1. Molecular Orbital Data

Ligand	Egap (eV)	IP (eV)	EA (eV)	μ (eV)	H (eV)	χ (eV)	η (eV)	σ (eV)	ω (eV)	ΔNmax (eV)	dipole (D)
**1**	4.300	5.769	1.469	-3.619	4.300	3.619	2.150	0.173	3.046	1.683	2.85
**2**	4.245	5.932	1.687	-3.810	4.245	3.810	2.123	0.169	3.419	1.795	2.08
**3**	4.218	5.960	1.742	-3.851	4.218	3.851	2.109	0.168	3.516	1.826	2.78
**4**	3.973	6.286	2.313	-4.300	3.973	4.300	1.987	0.159	4.653	2.164	6.81
**5**	3.320	5.796	2.476	-4.136	3.320	4.136	1.660	0.173	5.153	2.492	3.99
**6**	3.184	5.932	2.748	-4.340	3.184	4.340	1.592	0.169	5.916	2.726	7.46
**7**	3.701	5.361	1.66	-3.511	3.701	3.511	1.851	0.187	3.330	1.897	8.52
**8**	4.191	5.769	1.578	-3.674	4.191	3.674	2.096	0.173	3.220	1.753	6.19
**9**	3.728	5.470	1.742	-3.606	3.728	3.606	1.864	0.183	3.488	1.935	8.53
**10**	3.701	5.470	1.769	-3.620	3.701	3.620	1.851	0.183	3.540	1.956	14.77
**11**	3.429	5.388	1.959	-3.674	3.429	3.674	1.715	0.186	3.935	2.142	15.79
**12**	3.592	5.361	1.769	-3.565	3.592	3.565	1.796	0.187	3.538	1.985	13.32
**13**	3.510	5.388	1.878	-3.633	3.510	3.633	1.755	0.186	3.760	2.070	14.51
**14**	3.565	5.388	1.823	-3.606	3.565	3.606	1.783	0.186	3.646	2.022	14.44
**15**	3.510	5.388	1.878	-3.633	3.510	3.633	1.755	0.186	3.760	2.070	15.46
**16**	4.109	5.769	1.66	-3.715	4.109	3.715	2.055	0.173	3.358	1.808	6.54
**17**	4.054	5.660	1.606	-3.633	4.054	3.633	2.027	0.177	3.256	1.792	3.51
**18**	4.000	5.687	1.687	-3.687	4.000	3.687	2.000	0.176	3.398	1.844	11.22
**19**	2.177	4.708	2.531	-3.620	2.177	3.620	1.089	0.212	6.017	3.324	9.14
**20**	2.784	4.871	2.123	-3.497	2.784	3.497	2.748	0.364	2.225	1.273	11.07
Ampicillin	5.769	6.286	0.517	-3.402	5.769	3.402	2.885	0.159	2.006	1.179	4.37
Cefatrizine	4.218	5.932	1.714	-3.823	4.218	3.823	2.109	0.169	3.465	1.813	3.74
Fluconazole	6.286	6.885	0.599	-3.742	6.286	3.742	3.143	0.145	2.228	1.191	3.43

Gemcitabine	5.307	6.368	1.061	-3.715	5.307	3.715	2.654	0.157	2.600	1.400	5.47
Itraconazole	3.918	4.925	1.007	-2.966	3.918	2.966	1.959	0.203	2.245	1.514	4.58
Ribavirin	5.878	6.83	0.952	-3.891	5.878	3.891	2.939	0.146	2.576	1.324	6.90
Rufinamide	5.579	6.749	1.17	-3.960	5.579	3.960	2.790	0.148	2.810	1.419	9.00
Streptomycin	4.844	5.742	0.898	-3.320	4.844	3.320	2.422	0.174	2.275	1.371	3.77
Tazobactam	6.341	7.293	0.952	-4.123	6.341	4.123	3.171	0.137	2.680	1.300	6.30

[a]Calculated by: HOMO energy (ELUMO), LUMO energy (ELUMO), Energy gap (Egap)= ELUMO − EHOMO, Ionization potential (IP) = −EHOMO, Electron affinity (EA) = −ELUMO, Electronegativity (χ) = (IP + EA)/2, Chemical potential (μ) = $-\chi$, H = (IP − EA), Hardness (η) = (IP − EA)/2, Softness (σ) = $1/\eta$, Electrophilicity (ω) = $\mu^2/2\eta$, ΔNmax = $-(\mu/\eta)$.

In comparison to reference drugs ampicillin, cefatrizine, fluconazole, gemcitabine, itraconazole, ribavirin, rufinamide, streptomycin, and tazobactam, study indicates that potential, naturally derived anti-cancer chemicals are more reactive. This is demonstrated by their lower hardness, higher softness, electronegativity, and electrophilicity index. The energy reduction caused by electron transfer between the HOMO and LUMO is measured by the electrophilicity index, a typical metric for biological activity and reactive sites. Higher binding affinities and interactions between the putative anti-cancer compounds and the target proteins, the electron-donating group (EDG) and the electron-withdrawing group (EWG), are indicated by a dipole moment that is greater than that of the reference medications. Compounds **15** and **16** proved to be a good choice as antibacterial and anticancer agents during the course of the investigation into their biological functions. Visual examination of the electron density distributions and atom affiliations, as demonstrated by the MEP maps in Figures S1 and S6 to S11 in the Supplementary Information, can reveal the compounds' electrostatic potential. The colors on the MEP maps indicate the strength of the electric potential; blue indicates electrophilic zones and red indicates nucleophilic regions. In conclusion, this study contributes significantly to our understanding of the chemical and reactive properties of the compounds under investigation.

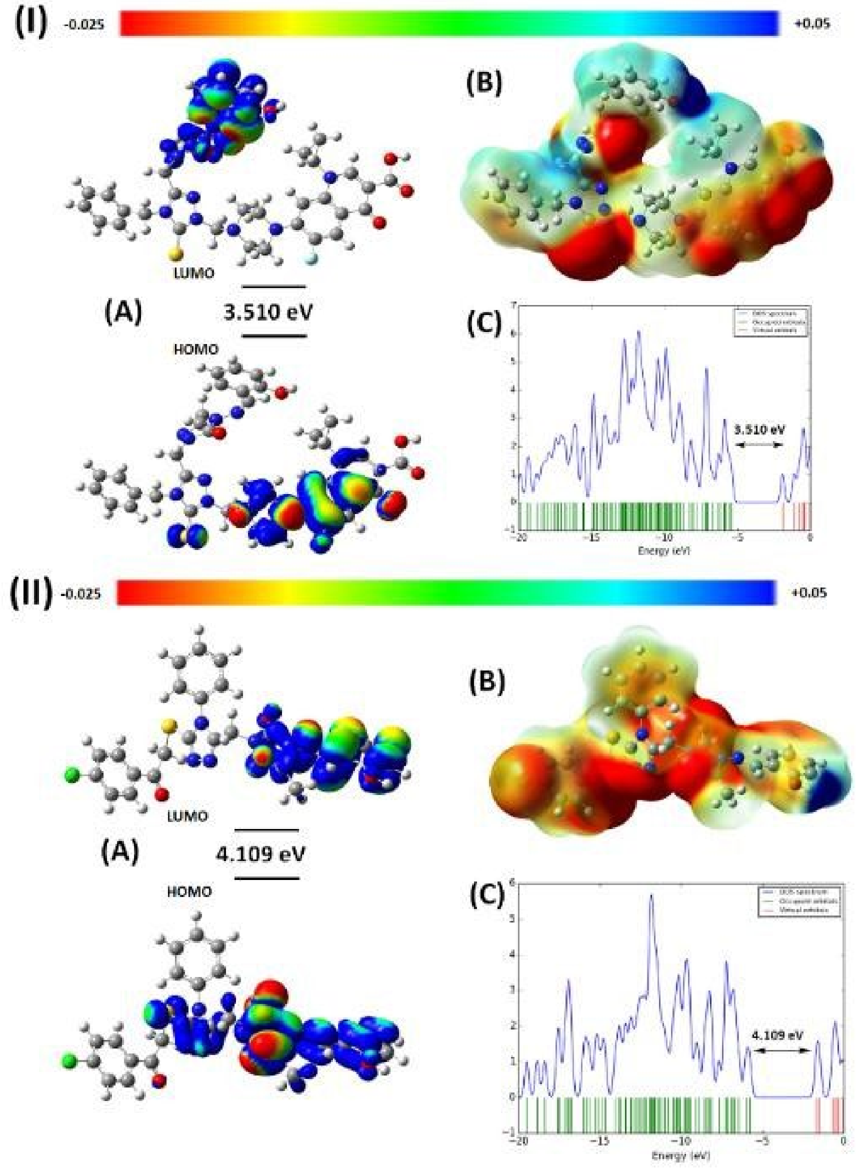

Figure S1. (A) Molecular orbitals of isodensity surfaces (0.02 electrons Bohr−3 surface) (red = electron-rich, blue = electron-deficient) of HOMO and LUMO; (B) Maps of electrostatic potential (0.02 electrons Bohr−3 surface) (red = electron-rich, blue = electron-deficient); and (C) DOS plot and HOMO-LUMO energy gap of (I) compound **15** and (II) compound **16**.

S.2. Validation of Protein Structures

We employed several online resources to investigate the structural integrity of Staphylococcus aureus DNA Gyrase (Topoisomerase II, PDB ID: 5CDQ) and Lysine-specific demethylase1 (LSD1, PDB ID: 2Z3Y) proteins. The resulting protein structure from Topo II and LSD1 was quality assessed using a variety of online methods. PROCHECK was used to generate a Ramachandran plot for Topo II and LSD1, which provide information about changes in protein structure after manufacturing, as shown in Figures S2 (A) and S3 (A), respectively. PROCHECK's Ramachandran map [90-98] shows that 91.4% of the type II topoisomerase and 91.7% of the LSD1 residues are in favorable regions as shown in Table S2 in the SI. The overall quality factor estimated by ERRAT [99] for the protein structures was 94.85% for topoisomerase II and 92.28% for LSD1, as shown in Figures S2 (B) and S3 (B). According

to the Verify3D [100-104] score data, 72.92% of topoisomerase II amino acid residues and 83.98% of LSD1 amino acid residues had an average atomic model (3D) to amino acid sequence (3D- 1D) score of 0.1, signifying a satisfactory score, as illustrated in Figures S2 (C) and S3 (C), respectively. Z scores for all chains in topoisomerase II and LSD1 were obtained from the ProSA-web [105,106] server by NMR spectroscopy (dark blue) or X-ray crystallography (light blue), as indicated in Figures S2 (D) and S3 (D). An overall model quality of -9.77 for topoisomerase II and -11.33 for LSD1 is shown. The local model quality as determined by the ProSA-web energy plots of topoisomerase II and LSD1, respectively, is displayed in Figures S2 (E) and S3 (E). The average energy throughout a 10-residue window size is shown by the thin line in the plot's backdrop, while the thick line shows the average energy throughout each 40-residue fragment. Using PyRx's Open Babel package, compounds underwent energy reduction, laying the groundwork for further in silico studies.

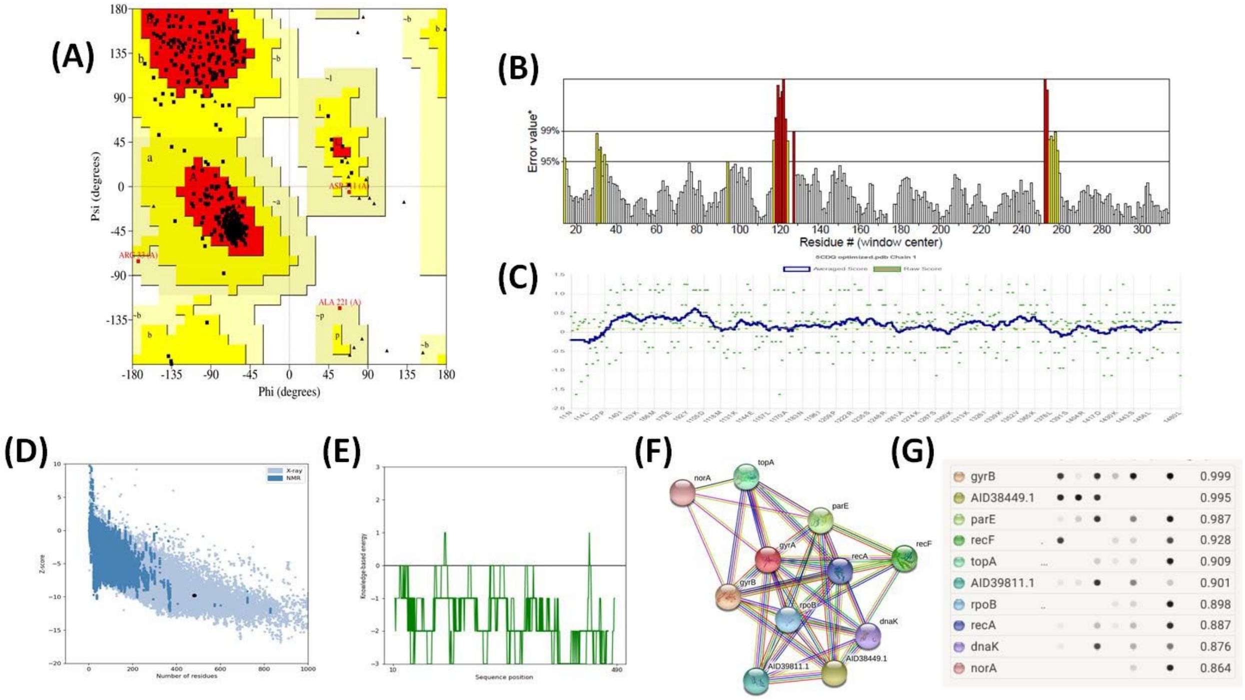

Figure S2. Validation and quality evaluation of the prepared protein structure of toposinerase II (5CDQ) using (**A**) a Ramachandran plot generated via SAVESv6.0, (**B**) an ERRAT plot generated via SAVESv6.0, (**C**) a Verify3D plot showing amino acids in favored regions generated via SAVESv6.0, (**D**) overall model quality determined via ProSAweb, and (**E**) local model quality determined via ProSAweb; (**F**) STRINGS output of topoisomerase II and (**G**) Interaction partner of 5CDQ.

The goal of the Search Tool Retrieval of Interacting Genes/Proteins (STRING) (http://string-db.org/) [107] database is to collect, score, and integrate all publicly available sources of information on protein-protein interaction and complement these with computational estimations in order to create an objective and comprehensive global network that includes both direct (physical) and indirect (functional) interaction. Protein-protein interactions between topoisomerase II and LSD1 have been investigated with the aid of the publicly available, free STRING database. Predicting much of the aggregate data for protein-protein related clusters is one advantage of the STRING database. The PPI (protein-protein interaction) network of the elements in this example was constructed using the STRING database, and any interaction with a total score higher than 0.4 was assumed to be statistically significant. As evidenced by the highest scores of 0.999 for topoisomerase II (Figures S2F and S2G) and LSD1 II (Figures S3F and S3G), the results obtained here are statistically highly significant.

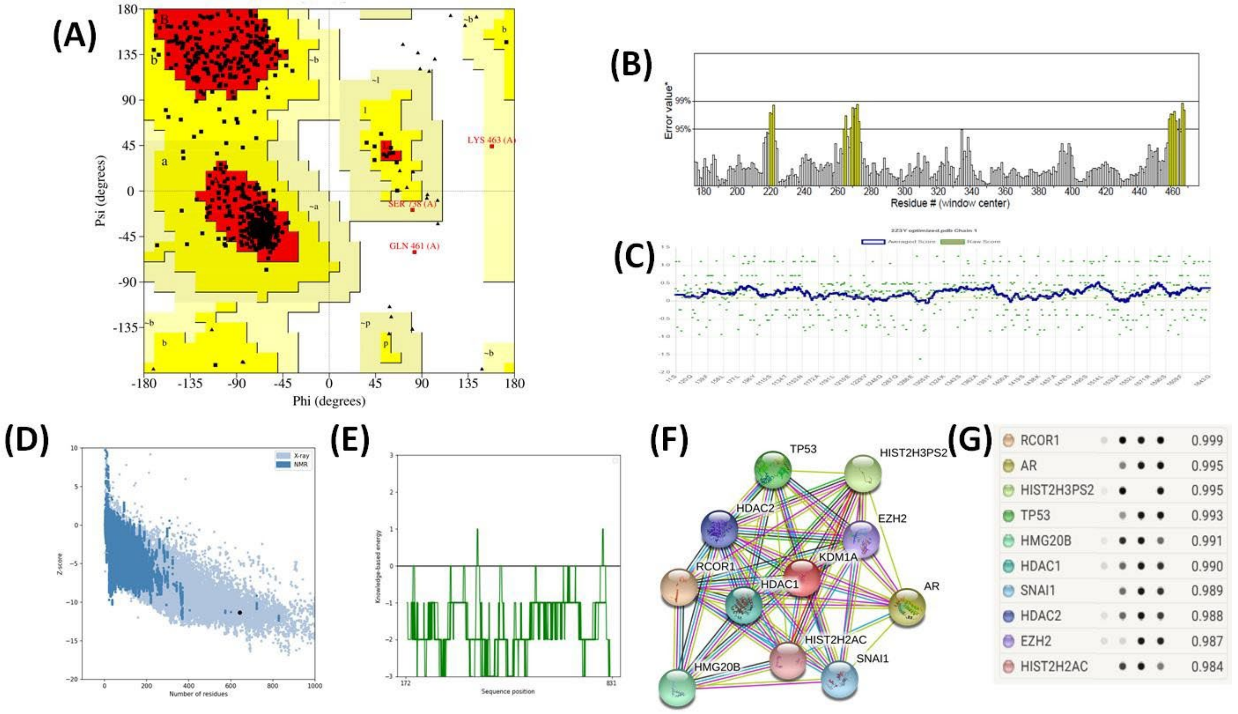

Figure S3. Validation and quality evaluation of the prepared protein structure of LSD1 using (**A**) a Ramachandran plot generated via SAVESv6.0, (**B**) an ERRAT plot generated via SAVESv6.0, (**C**) a Verify3D plot showing amino acids in favored regions generated via SAVESv6.0, (D) overall model quality determined via ProSAweb, and (**E**) local model quality determined via ProSAweb; (**F**) STRINGS output of LSD1 and (**G**) Interaction partner of 2Z3Y.

Table S2. Validation parameters of structures of proteins topoisomerase II and LSD1.

Parameters	Topoisomerase II (5CDQ)	LSD1 (2Z3Y)
Ramachandran Favored	91.4%	91.7%
ERRAT Quality Factor	94.85%	92.28%
Verify3D Score	72.92%	83.98%
ProSA-web Z Score	-9.77	-11.33
ProSA-web Energy Plot	Stable (10 & 40-residue windows)	Stable (10 & 40-residue windows)
SMILES	0.999	0.999

Table S3. Optimized structure for Compound **1** and Cartesian Z-matrix.

**Compound 1**		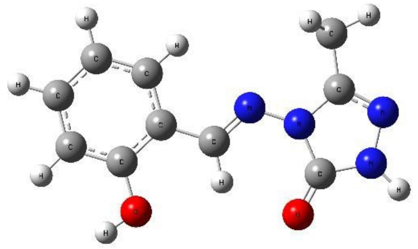		
Center Number	Atom	Standard orientation: Coordinates (Angstroms)		
		X	Y	Z
1	C	-2.74674	-0.8996	-0.0004
2	C	-4.08964	-0.5078	-0.0004
3	C	-4.42322	0.843743	0.000136
4	C	-3.41889	1.817133	0.000675
5	C	-2.08595	1.425734	0.000661
6	C	-1.71944	0.06882	0.000132
7	H	-4.86905	-1.26679	-0.00086
8	H	-5.46919	1.135552	0.000131
9	H	-3.67839	2.870816	0.001111
10	H	-1.29149	2.163986	0.00108
11	O	-2.37807	-2.21296	-0.00102
12	H	-3.16802	-2.76874	-0.00071
13	C	-0.32185	-0.36801	0.000156
14	H	-0.1164	-1.43439	0.000123
15	N	0.61704	0.518301	0.000151
16	N	1.923151	0.093562	-6.9E-05
17	C	2.970209	1.004547	-0.00037
18	C	2.772705	2.478196	-0.00066
19	H	2.208598	2.7976	-0.882
20	H	2.208557	2.797979	0.880534
21	H	3.750672	2.961345	-0.00071
22	N	4.124586	0.405126	-0.00034
23	C	2.469766	-1.2153	0.000466
24	O	1.889683	-2.29168	0.00111
25	N	3.813273	-0.94143	-0.00026
26	H	4.543758	-1.63397	0.001826

Table S4. Optimized structure for Compound **2** and Cartesian Z-matrix.

**Compound 2**		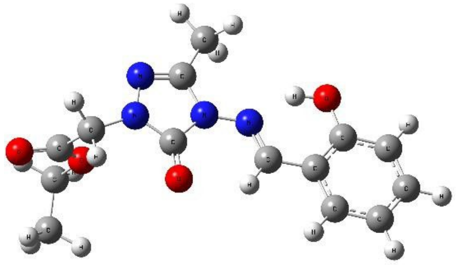		
Center Number	Atom	Standard orientation: Coordinates (Angstroms)		
		X	Y	Z
1	C	5.469769	0.056607	-0.7729
2	C	6.036239	-1.11764	-0.29267
3	C	5.27135	-2.04075	0.434583
4	C	3.932829	-1.76981	0.672722
5	C	3.326856	-0.58797	0.199844
6	C	4.117319	0.336985	-0.53569
7	H	6.05068	0.778692	-1.33662
8	H	7.086072	-1.31873	-0.48566
9	H	5.72058	-2.95539	0.806551
10	H	3.323995	-2.4734	1.234587
11	O	3.613362	1.487666	-1.02115
12	H	2.662302	1.529145	-0.76803
13	C	1.921267	-0.36666	0.484002
14	H	1.382962	-1.12254	1.051942
15	N	1.326862	0.709674	0.067087
16	N	-0.00407	0.901942	0.345393
17	C	-0.68657	2.032824	-0.0806
18	C	-0.90714	0.079491	1.054779
19	O	-0.70748	-1.00789	1.58255
20	N	-2.05994	0.825281	1.013399
21	N	-1.92899	2.003842	0.299868
22	C	-0.05741	3.128302	-0.8649
23	H	0.769288	3.587101	-0.31297
24	H	0.342678	2.756469	-1.81354
25	H	-0.81366	3.887089	-1.06941
26	C	-3.34703	0.372583	1.467099
27	H	-3.87773	1.189192	1.960679
28	H	-3.171	-0.41971	2.201244
29	C	-4.26861	-0.19231	0.385623
30	O	-5.45872	-0.32931	0.563313
31	O	-3.60131	-0.53651	-0.72615
32	C	-4.38659	-1.1248	-1.79664
33	H	-5.35973	-0.62974	-1.82726
34	H	-3.8263	-0.88507	-2.70283
35	C	-4.53361	-2.6256	-1.60688
36	H	-5.11275	-2.84502	-0.70676

37	H	-5.05871	-3.05644	-2.46566
38	H	-3.55456	-3.10604	-1.52529

Table S5. Optimized structure for Compound **3** and Cartesian Z-matrix.

**Compound 3**		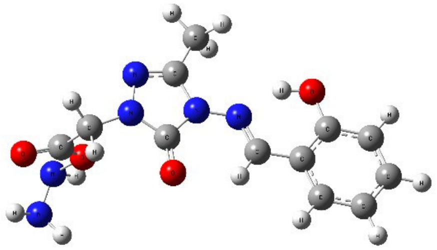		
Center Number	Atom	Standard orientation: Coordinates (Angstroms)		
		X	Y	Z
1	C	5.486325	0.036164	-0.77108
2	C	6.040907	-1.15483	-0.31916
3	C	5.266087	-2.08857	0.383699
4	C	3.929714	-1.81119	0.626131
5	C	3.335609	-0.61234	0.181662
6	C	4.136069	0.32325	-0.52926
7	H	6.075043	0.766587	-1.31561
8	H	7.089213	-1.3608	-0.51519
9	H	5.706207	-3.0162	0.733731
10	H	3.313355	-2.52275	1.169485
11	O	3.643952	1.490306	-0.98665
12	H	2.692921	1.534914	-0.73419
13	C	1.931587	-0.38515	0.468499
14	H	1.38493	-1.15054	1.015385
15	N	1.347862	0.706815	0.077893
16	N	0.017929	0.903888	0.357913
17	C	-0.64995	2.05799	-0.0284
18	C	-0.89909	0.063756	1.02695
19	O	-0.71657	-1.04808	1.509158
20	N	-2.04332	0.822901	1.008079
21	N	-1.89521	2.02705	0.342404
22	C	-0.00429	3.176933	-0.76466
23	H	0.824044	3.604111	-0.19032
24	H	0.397103	2.83919	-1.72544
25	H	-0.75133	3.951292	-0.94287
26	C	-3.33698	0.367151	1.441414
27	H	-3.88675	1.197009	1.889897
28	H	-3.17803	-0.39773	2.207138
29	C	-4.23354	-0.24301	0.356743
30	O	-5.41095	-0.45476	0.554813
31	O	-3.54898	-0.55531	-0.74805
32	N	-4.48208	-1.08279	-1.86855

33	H	-3.75038	-1.14683	-2.57586
34	N	-4.91764	-2.35449	-1.61127
35	H	-5.67176	-2.30021	-0.93388
36	H	-4.19177	-2.99997	-1.29707

Table S6. Optimized structure for Compound **4** and Cartesian Z-matrix.

**Compound 4**	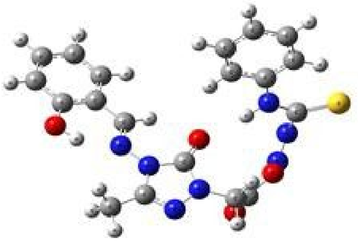			
Center Number	Atom	Standard orientation: Coordinates (Angstroms)		
		X	Y	Z
1	C	1.553112	2.493714	1.942247
2	C	1.777844	3.652436	1.183896
3	C	0.750201	4.158634	0.405126
4	C	-0.51427	3.534116	0.361841
5	C	-0.72496	2.36099	1.137831
6	H	0.13539	0.957763	2.503807
7	H	2.351542	2.08775	2.55649
8	H	2.743453	4.1458	1.206638
9	H	0.905726	5.05516	-0.18938
10	O	-1.90138	1.707881	1.152434
11	H	-2.52082	2.183871	0.553961
12	C	-1.54509	4.114353	-0.47412
13	H	-1.29677	5.005106	-1.04694
14	N	-2.7262	3.578443	-0.53928
15	N	-3.68154	4.148397	-1.35117
16	C	-5.0189	3.783697	-1.27524
17	C	-3.54296	5.08804	-2.3832
18	O	-2.51367	5.653404	-2.77479
19	N	-4.82333	5.217254	-2.83919
20	N	-5.72788	4.426188	-2.15651
21	C	-5.5523	2.795641	-0.30103
22	H	-5.11108	1.806141	-0.45844
23	H	-5.3348	3.098692	0.727609
24	H	-6.63262	2.727381	-0.43358
25	C	-5.25689	6.14123	-3.87122
26	H	-6.34581	6.174024	-3.82068
27	H	-4.94173	5.798985	-4.85993
28	C	-4.68851	7.540152	-3.55939
29	O	-4.93447	8.141977	-2.54605
30	O	-3.85622	7.912695	-4.55084
31	N	-2.88915	9.081108	-4.09667
32	N	-1.85132	8.993047	-4.69421
33	C	-1.49915	8.07565	-5.7663

34	N	-1.30212	6.820476	-5.33042
35	H	-1.66698	6.608299	-4.39881
36	S	-1.31172	8.809825	-7.23323
37	C	-0.72529	5.696663	-5.97846
38	C	-0.32598	5.672946	-7.32211
39	C	-0.5544	4.55236	-5.17962
40	C	0.250626	4.513324	-7.84168
41	H	-0.4705	6.541054	-7.94785
42	C	0.016227	3.404378	-5.71767
43	H	-0.87653	4.573918	-4.14384
44	C	0.426307	3.378015	-7.05252
45	H	0.55848	4.504605	-8.88294
46	H	0.142754	2.52827	-5.08873
47	H	0.873057	2.481799	-7.47154

Table S7. Optimized structure for Compound **5** and Cartesian Z-matrix.

**Compound 5**	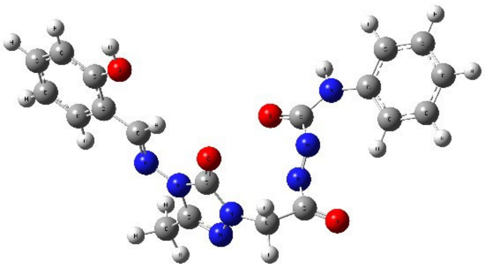			
Center Number	Atom	Standard orientation: Coordinates (Angstroms)		
		X	Y	Z
1	C	-6.43972	1.487802	-1.20284
2	C	-5.32814	0.691888	-0.95667
3	C	-4.47518	0.939703	0.132621
4	C	-4.77505	2.0272	0.981373
5	C	-5.89385	2.830064	0.735261
6	C	-6.72132	2.561088	-0.35136
7	H	-7.084	1.278426	-2.05054
8	H	-5.09032	-0.14418	-1.60514
9	H	-6.11107	3.664131	1.399124
10	H	-7.58676	3.191561	-0.53232
11	O	-3.94044	2.256027	2.03581
12	H	-4.25084	3.025458	2.530286
13	C	-3.29748	0.116008	0.41126
14	H	-2.67662	0.379972	1.262349
15	N	-3.03422	-0.8897	-0.3537
16	N	-1.92483	-1.652	-0.07729
17	C	-1.57675	-2.72547	-0.88425
18	C	-0.97875	-1.55655	0.96435
19	N	-0.50801	-3.32505	-0.45032

20	N	-0.14726	-2.6197	0.688194
21	O	-0.90889	-0.75423	1.886203
22	C	-2.34572	-3.11962	-2.09336
23	H	-3.38299	-3.35612	-1.83862
24	H	-2.36456	-2.30377	-2.82186
25	H	-1.87069	-3.99392	-2.54002
26	C	1.062814	-2.92215	1.400568
27	H	1.154438	-3.99774	1.572175
28	H	1.000581	-2.41418	2.370266
29	C	2.336689	-2.43026	0.720808
30	N	2.134482	-1.44888	-0.31735
31	N	2.442982	-0.28926	0.008336
32	O	3.440548	-2.8606	0.950728
33	C	2.21831	0.660719	-1.08376
34	O	1.174287	0.698558	-1.70317
35	N	3.236204	1.557811	-1.24631
36	H	2.992471	2.279721	-1.91273
37	C	4.506949	1.675146	-0.63009
38	C	5.081161	2.954478	-0.59249
39	C	5.216936	0.584433	-0.11021
40	C	6.339781	3.144734	-0.02985
41	H	4.530906	3.800264	-0.99662
42	C	6.467858	0.792924	0.470718
43	H	4.807522	-0.41641	-0.14692
44	C	7.038358	2.064661	0.512458
45	H	6.76964	4.141646	-0.00912
46	H	7.003884	-0.05854	0.878628
47	H	8.017646	2.212271	0.956534

Table S8. Optimized structure for Compound **6** and Cartesian Z-matrix.

**Compound 6**		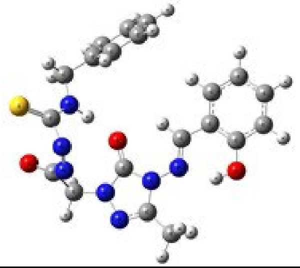		
Center Number	Atom	Standard orientation: Coordinates (Angstroms)		
		X	Y	Z
1	C	5.900284	0.138401	0.786204
2	C	5.945423	1.523508	0.693675
3	C	4.807854	2.259513	0.330359
4	C	3.625641	1.58906	0.060585
5	C	3.546439	0.182758	0.144959
6	C	4.70884	-0.54814	0.515593
7	H	6.772832	-0.44153	1.066799
8	H	6.877001	2.039564	0.907173

9	H	4.853095	3.341129	0.261352
10	H	2.733806	2.143801	-0.21944
11	O	4.714501	-1.89013	0.618777
12	H	3.811353	-2.21439	0.401622
13	C	2.280628	-0.45643	-0.15219
14	H	1.441961	0.176694	-0.43329
15	N	2.160668	-1.74796	-0.08463
16	N	0.949872	-2.3368	-0.37331
17	C	0.771082	-3.71171	-0.29404
18	C	-0.26546	-1.76295	-0.76863
19	O	-0.53517	-0.57028	-0.95424
20	N	-1.08011	-2.84992	-0.90163
21	N	-0.44707	-4.04369	-0.60732
22	C	1.842943	-4.66217	0.102442
23	H	2.694857	-4.60632	-0.58245
24	H	2.209144	-4.44418	1.11051
25	H	1.43432	-5.67305	0.082921
26	C	-2.46792	-2.81476	-1.30594
27	H	-2.82453	-3.84761	-1.27783
28	H	-2.57315	-2.44163	-2.32878
29	C	-3.36779	-1.95843	-0.4152
30	O	-4.44881	-1.56305	-0.78731
31	N	-2.90343	-1.86774	0.944487
32	N	-2.99405	-0.82238	1.624261
33	C	-3.47347	0.422405	1.079554
34	N	-2.66502	1.018674	0.202279
35	H	-1.86146	0.497565	-0.16104
36	S	-4.87827	0.963346	1.778933
37	C	-2.90003	2.356254	-0.33848
38	H	-3.4133	2.275245	-1.3041
39	H	-3.59249	2.854973	0.348352
40	C	-1.60981	3.134084	-0.48758
41	C	-0.78952	3.368778	0.624895
42	C	-1.22755	3.648483	-1.73069
43	C	0.386767	4.105651	0.494281
44	H	-1.07816	2.974706	1.595553
45	C	-0.05193	4.389821	-1.86391
46	H	-1.85449	3.468472	-2.60015
47	C	0.758046	4.62008	-0.75178
48	H	1.008772	4.287438	1.365978
49	H	0.230089	4.78473	-2.83545
50	H	1.66966	5.202025	-0.85203

Table S9. Optimized structure for Compound **7** and Cartesian Z-matrix.

**Compound 7**		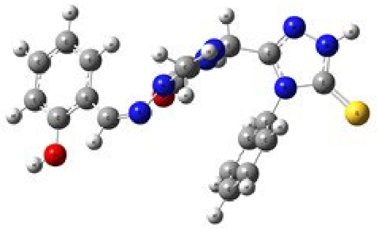		
Center Number	Atom	Standard orientation: Coordinates (Angstroms)		
		X	Y	Z
1	N	0.313728	1.341619	0.092927
2	N	0.591196	1.661115	1.41144
3	C	-0.32106	1.038635	2.099873
4	C	-0.42825	1.042939	3.58253
5	H	-1.36465	1.505256	3.911541
6	H	-0.41732	0.019363	3.968685
7	H	0.412264	1.601418	3.996412
8	N	-1.22523	0.382175	1.277594
9	N	-2.07598	-0.59984	1.782055
10	C	-3.21996	-0.79454	1.218406
11	H	-3.73452	-1.65229	1.648799
12	C	-3.99169	-0.0805	0.194961
13	C	-3.86677	1.294813	-0.06784
14	C	-5.01713	-0.79017	-0.46952
15	C	-4.71824	1.944984	-0.95306
16	H	-3.10469	1.8623	0.452293
17	C	-5.86514	-0.14228	-1.37154
18	C	-5.71498	1.220529	-1.61133
19	H	-4.60568	3.00905	-1.13178
20	H	-6.63938	-0.70981	-1.88312
21	H	-6.37908	1.716022	-2.31304
22	C	-0.77412	0.507465	-0.05771
23	O	-1.22934	0.010578	-1.07444
24	C	1.139585	1.810282	-0.99728
25	H	1.043407	2.89271	-1.12687
26	H	0.746728	1.316158	-1.89113
27	C	2.601353	1.536679	-0.80927
28	N	3.497955	2.478829	-0.87757
29	N	4.679336	1.823097	-0.66785
30	H	5.553397	2.32475	-0.65728
31	C	4.558942	0.483753	-0.45204
32	N	3.178526	0.300559	-0.55798
33	O	-5.13641	-2.12235	-0.19896
34	H	-5.89337	-2.47958	-0.68142
35	C	2.498396	-0.95386	-0.41262
36	C	2.476722	-1.58135	0.83478
37	C	1.86857	-1.52842	-1.5164
38	C	1.804918	-2.79387	0.973878

39	H	2.985133	-1.12101	1.673852
40	C	1.188297	-2.73661	-1.36392
41	H	1.914771	-1.03742	-2.48281
42	C	1.157439	-3.36964	-0.12187
43	H	1.784946	-3.28784	1.940238
44	H	0.685296	-3.1795	-2.21724
45	H	0.629106	-4.31096	-0.00606
46	S	5.770726	-0.62128	-0.15241
47	N	0.313728	1.341619	0.092927

Table S10. Optimized structure for Compound **8** and Cartesian Z-matrix.

**Compound 8**		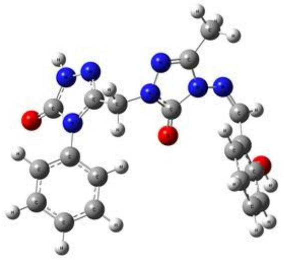		
Center Number	Atom	Standard orientation: Coordinates (Angstroms)		
		X	Y	Z
1	N	-0.05605	1.57131	1.06702
2	N	0.378306	2.835441	1.432741
3	C	1.535943	2.9697	0.856045
4	C	2.404708	4.17281	0.942275
5	H	3.356698	3.943192	1.432217
6	H	2.63489	4.548822	-0.05896
7	H	1.884235	4.943272	1.512498
8	N	1.896059	1.821866	0.161375
9	N	2.919012	1.843281	-0.7842
10	C	3.59561	0.768325	-1.00898
11	H	4.289847	0.900588	-1.83736
12	C	3.679216	-0.54288	-0.35489
13	C	3.393067	-0.76328	1.003479
14	C	4.209607	-1.61837	-1.10229
15	C	3.618205	-1.99745	1.60181
16	H	3.011418	0.05647	1.600077
17	C	4.422795	-2.86469	-0.50725
18	C	4.127817	-3.05186	0.840122
19	H	3.396868	-2.13869	2.654421
20	H	4.819055	-3.68358	-1.10359
21	H	4.298343	-4.0232	1.294495
22	C	0.806121	0.925706	0.212172
23	O	0.670228	-0.1492	-0.35614
24	C	-1.40751	1.139275	1.355122
25	H	-1.74044	1.735046	2.208069

26	H	-1.39158	0.089699	1.652453
27	C	-2.36027	1.329507	0.199532
28	N	-2.50642	2.465447	-0.41505
29	N	-3.50958	2.234666	-1.3257
30	H	-3.818	2.967837	-1.94251
31	C	-4.0317	0.962828	-1.28521
32	N	-3.24344	0.364684	-0.26936
33	O	4.494657	-1.38897	-2.41641
34	H	4.876892	-2.18649	-2.80525
35	C	-3.34945	-1.01487	0.084039
36	C	-4.60614	-1.537	0.406046
37	C	-2.21533	-1.83446	0.073778
38	C	-4.72294	-2.88571	0.736686
39	H	-5.47662	-0.89237	0.378606
40	C	-2.34552	-3.17841	0.42714
41	H	-1.25059	-1.42576	-0.21354
42	C	-3.59398	-3.70718	0.75711
43	H	-5.69901	-3.29324	0.982005
44	H	-1.46546	-3.8145	0.42684
45	H	-3.6893	-4.75619	1.021284
46	O	-4.93288	0.468199	-1.9387

Table S11. Optimized structure for Compound **9** and Cartesian Z-matrix.

**Compound 9**		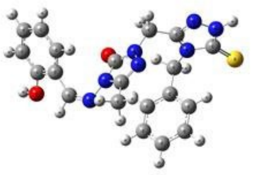		
Center Number	Atom	Standard orientation: Coordinates (Angstroms)		
		X	Y	Z
1	N	0.185968	1.01542	1.040274
2	N	0.37946	0.341691	2.234778
3	C	-0.52741	-0.5914	2.234997
4	C	-0.6925	-1.62844	3.286229
5	H	-1.68563	-1.57915	3.743652
6	H	-0.57851	-2.62584	2.850828
7	H	0.067659	-1.47703	4.053498
8	N	-1.35644	-0.4998	1.124999
9	N	-2.18924	-1.55262	0.749764
10	C	-3.29515	-1.28856	0.139843
11	H	-3.7991	-2.1975	-0.18533
12	C	-4.03484	-0.05283	-0.14216
13	C	-3.9659	1.102811	0.655058
14	C	-4.97256	-0.07391	-1.19866
15	C	-4.78768	2.198	0.417067

16	H	-3.27288	1.126915	1.487498
17	C	-5.7899	1.031376	-1.44943
18	C	-5.69636	2.162372	-0.64363
19	H	-4.72123	3.074873	1.052292
20	H	-6.49566	1.000887	-2.27652
21	H	-6.33613	3.016183	-0.84508
22	C	-0.84609	0.509468	0.283888
23	O	-1.20886	0.83991	-0.83792
24	C	0.964443	2.176378	0.677561
25	H	0.911967	2.927319	1.470394
26	H	0.483708	2.580122	-0.21988
27	C	2.423301	1.937129	0.422369
28	N	3.340054	2.632192	1.037979
29	N	4.504742	2.232879	0.44587
30	H	5.388786	2.627941	0.725518
31	C	4.347216	1.310904	-0.54132
32	N	2.972809	1.106733	-0.53863
33	O	-5.03666	-1.20591	-1.9571
34	H	-5.73479	-1.10983	-2.61805
35	S	5.524931	0.633162	-1.51777
36	C	2.281248	0.183592	-1.44737
37	H	1.321569	0.630202	-1.7198
38	H	2.910785	0.142331	-2.3428
39	C	2.066591	-1.21576	-0.89301
40	C	3.066074	-1.88739	-0.17673
41	C	0.860286	-1.87491	-1.15772
42	C	2.854489	-3.19061	0.271797
43	H	4.015169	-1.39888	0.015675
44	C	0.651206	-3.18114	-0.71023
45	H	0.07272	-1.35821	-1.69888
46	C	1.647925	-3.84215	0.007161
47	H	3.63828	-3.6997	0.825306
48	H	-0.29681	-3.67037	-0.91056
49	H	1.487463	-4.85799	0.357019

Table S12. Optimized structure for Compound **10** and Cartesian Z-matrix.

**Compound 10**	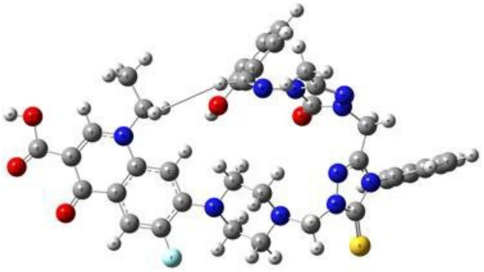		
Center Number	Atom	Standard orientation: Coordinates (Angstroms)		

Center Number	Atom	X	Y	Z
1	N	3.931199	1.946277	1.287782
2	N	4.016608	2.415304	2.59085
3	C	2.819039	2.831049	2.876522
4	C	2.405837	3.43184	4.172095
5	H	2.085151	4.471252	4.045561
6	H	1.563691	2.879037	4.598657
7	H	3.250806	3.400196	4.860994
8	N	1.955118	2.6824	1.797149
9	N	0.574953	2.716791	1.965772
10	C	-0.15416	3.023523	0.950268
11	H	-1.21834	2.909772	1.158431
12	C	0.190688	3.553502	-0.38078
13	C	1.075987	4.627486	-0.54356
14	C	-0.45347	3.03419	-1.51781
15	C	1.321666	5.17105	-1.80187
16	H	1.564591	5.042918	0.331804
17	C	-0.20666	3.57013	-2.78169
18	C	0.681535	4.635633	-2.92071
19	H	2.005024	6.006834	-1.90887
20	H	-0.70438	3.150741	-3.65297
21	H	0.868913	5.047871	-3.90739
22	C	2.652948	1.993386	0.774811
23	O	2.231832	1.538488	-0.27445
24	C	4.982582	1.135487	0.721629
25	H	5.887805	1.344614	1.296961
26	H	5.162191	1.438808	-0.31416
27	C	4.628454	-0.32998	0.739564
28	N	3.745862	-0.86255	1.528944
29	N	3.671581	-2.17664	1.136594
30	C	4.48664	-2.47414	0.08125
31	S	4.678437	-3.92744	-0.72321
32	N	5.133921	-1.25806	-0.15267
33	O	-1.31194	1.984736	-1.32035
34	H	-1.59295	1.630496	-2.17462
35	C	2.597348	-3.02022	1.660097
36	H	2.630253	-2.92473	2.753793

37	H	2.866173	-4.03929	1.379092
38	N	1.313466	-2.66204	1.100538
39	N	-1.4834	-2.30967	0.673292
40	C	0.547801	-1.71966	1.913056
41	H	1.191396	-0.8763	2.174534
42	H	0.196128	-2.18934	2.851927
43	C	-0.66321	-1.19276	1.142874
44	H	-0.31776	-0.55676	0.311243
45	H	-1.25746	-0.57476	1.821037
46	C	-0.70037	-3.2544	-0.14258
47	H	-0.32444	-2.74531	-1.04481
48	H	-1.33206	-4.08368	-0.44991
49	C	0.488848	-3.7833	0.654694
50	H	0.108707	-4.39014	1.497413
51	H	1.092215	-4.4293	0.009737
52	C	-2.82423	-2.08379	0.362364
53	C	-3.379	-0.79577	0.280036
54	C	-3.72442	-3.16795	0.187089
55	C	-4.75326	-0.59658	0.045185
56	H	-2.71638	0.051576	0.371637
57	C	-5.05337	-2.98717	-0.09562
58	C	-5.60264	-1.69827	-0.17257
59	H	-5.71377	-3.83627	-0.23133
60	C	-6.62662	0.851045	-0.26394
61	H	-6.98784	1.867495	-0.28279
62	C	-7.51167	-0.17077	-0.51085
63	F	-3.26093	-4.43586	0.324591
64	N	-5.30819	0.697805	0.009054
65	C	-7.05197	-1.56511	-0.46968
66	O	-7.75487	-2.55519	-0.65779
67	C	-8.91848	0.166563	-0.80457
68	O	-9.8212	-0.60448	-1.04234
69	O	-9.14444	1.525868	-0.78778
70	H	-10.0897	1.614938	-0.98931
71	C	6.138299	-1.01539	-1.14361
72	C	5.817364	-1.12479	-2.49839
73	C	7.426657	-0.65865	-0.73948
74	C	6.798824	-0.86495	-3.45304
75	H	4.814972	-1.41669	-2.78833
76	C	8.399155	-0.38999	-1.7031
77	H	7.665527	-0.61018	0.318431
78	C	8.086427	-0.49389	-3.05885
79	H	6.5556	-0.95151	-4.50733
80	H	9.401186	-0.11157	-1.39212
81	H	8.845897	-0.29117	-3.80775
82	C	-4.44499	1.85484	0.327577
83	C	-5.13818	3.21224	0.350397
84	H	-3.62411	1.883324	-0.39594

85	H	-3.99576	1.663656	1.309309
86	H	-4.38998	3.966046	0.611541
87	H	-5.55326	3.486928	-0.62341
88	H	-5.93405	3.263071	1.098577

Table S13. Optimized structure for Compound **11** and Cartesian Z-matrix.

**Compound 11**		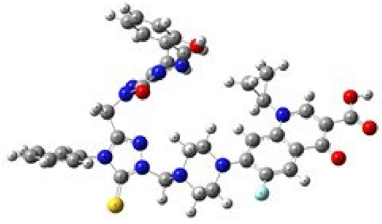		
Center Number	Atom	Standard orientation: Coordinates (Angstroms)		
		X	Y	Z
1	N	-3.57209	1.568724	-1.53996
2	N	-3.18899	1.943363	-2.81964
3	C	-2.09581	2.627619	-2.65281
4	C	-1.30438	3.256968	-3.74269
5	H	-1.2838	4.34712	-3.64189
6	H	-0.26836	2.906456	-3.71545
7	H	-1.75309	2.994547	-4.70152
8	N	-1.76672	2.744843	-1.30876
9	N	-0.49924	3.150286	-0.89235
10	C	-0.39016	3.793015	0.22245
11	H	0.652429	3.964005	0.485707
12	C	-1.3524	4.383372	1.157915
13	C	-2.63203	4.837352	0.79241
14	C	-0.91368	4.628849	2.478813
15	C	-3.44841	5.506265	1.696048
16	H	-2.9762	4.681095	-0.22274
17	C	-1.73872	5.288501	3.393433
18	C	-3.00082	5.724816	3.001653
19	H	-4.4283	5.854575	1.387487
20	H	-1.38865	5.455834	4.409467
21	H	-3.63485	6.237374	3.718644
22	C	-2.67018	1.948509	-0.56824
23	O	-2.65528	1.671688	0.619294
24	C	-4.59957	0.579046	-1.33533
25	H	-5.28464	0.641704	-2.18441
26	H	-5.16017	0.829571	-0.4293
27	C	-4.02706	-0.80706	-1.18329
28	N	-2.79961	-1.12488	-1.46177
29	N	-2.6861	-2.44358	-1.08745
30	C	-3.84	-2.96289	-0.56677
31	S	-4.13726	-4.50584	-0.00711
32	N	-4.71916	-1.87785	-0.64787

33	O	0.332819	4.190369	2.817023
34	H	0.527865	4.447678	3.72776
35	C	-1.39138	-3.10105	-1.13861
36	H	-0.90122	-2.73762	-2.05681
37	H	-1.59139	-4.17039	-1.2293
38	N	-0.60909	-2.86964	0.06233
39	N	1.728068	-2.31298	1.664653
40	C	-0.07893	-1.50383	0.145394
41	H	-0.9067	-0.79196	0.104643
42	H	0.589426	-1.28413	-0.71009
43	C	0.671829	-1.322	1.467496
44	H	-0.04601	-1.44641	2.287555
45	H	1.085771	-0.31485	1.546144
46	C	1.177715	-3.67286	1.558872
47	H	0.465019	-3.8152	2.379772
48	H	1.97243	-4.40722	1.66813
49	C	0.447411	-3.87355	0.225503
50	H	1.18783	-3.83657	-0.59787
51	H	-0.01389	-4.8673	0.214391
52	C	3.033062	-2.04315	1.253225
53	C	3.368996	-0.95968	0.426805
54	C	4.117596	-2.82276	1.736374
55	C	4.703342	-0.6664	0.101886
56	H	2.581127	-0.34914	0.013955
57	C	5.423205	-2.56035	1.409307
58	C	5.751236	-1.47338	0.583096
59	H	6.228157	-3.17124	1.802179
60	C	6.314764	0.638279	-1.09441
61	H	6.464294	1.461541	-1.7792
62	C	7.388919	-0.09224	-0.65353
63	F	3.851879	-3.85523	2.575503
64	N	5.02164	0.421663	-0.73502
65	C	7.179252	-1.23039	0.256975
66	O	8.067069	-1.94197	0.719888
67	C	8.740382	0.277478	-1.12196
68	O	9.780591	-0.28962	-0.87268
69	O	8.725942	1.397	-1.92324
70	H	9.655128	1.527459	-2.17189
71	C	-6.09436	-1.87819	-0.24863
72	C	-6.42479	-2.05338	1.096882
73	C	-7.08816	-1.69024	-1.21158
74	C	-7.76529	-2.02894	1.477167
75	H	-5.63794	-2.2141	1.82456
76	C	-8.42662	-1.65665	-0.81925
77	H	-6.81371	-1.59044	-2.25715
78	C	-8.76551	-1.82619	0.523553
79	H	-8.02773	-2.16755	2.521213
80	H	-9.20155	-1.50987	-1.56505

81	H	-9.80788	-1.8063	0.826519
82	C	3.971464	1.205448	-1.35584
86	C	3.135043	2.149802	-0.52709
84	C	4.144089	2.69325	-1.50511
85	H	3.464007	0.680021	-2.16313
86	H	3.379169	2.225655	0.527591
87	H	2.077658	2.224854	-0.76494
88	H	3.793917	3.145111	-2.42731
89	H	5.040743	3.146449	-1.0956

Table S14. Optimized structure for Compound **12** and Cartesian Z-matrix.

**Compound 12**		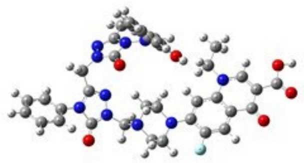		
Center Number	Atom	Standard orientation: Coordinates (Angstroms)		
		X	Y	Z
1	N	-3.84777	2.093955	-1.01951
2	N	-3.8379	2.820888	-2.20094
3	C	-2.66319	3.372554	-2.24646
4	C	-2.17441	4.259172	-3.33487
5	H	-1.95748	5.265436	-2.9617
6	H	-1.24946	3.862979	-3.76385
7	H	-2.94014	4.32135	-4.10908
8	N	-1.90702	3.06201	-1.12037
9	N	-0.53019	3.240964	-1.13251
10	C	0.119968	3.330349	-0.02403
11	H	1.196049	3.365828	-0.19345
12	C	-0.28037	3.466756	1.382878
13	C	-1.37354	4.244041	1.794249
14	C	0.546876	2.900331	2.372094
15	C	-1.63809	4.457047	3.142915
16	H	-2.00765	4.698541	1.040577
17	C	0.28	3.101089	3.726867
18	C	-0.81019	3.88034	4.108621
19	H	-2.48308	5.068638	3.440904
20	H	0.921393	2.645044	4.47748
21	H	-1.01158	4.034424	5.164203
22	C	-2.64535	2.130457	-0.34881
23	O	-2.30138	1.513547	0.646288
24	C	-4.88767	1.12388	-0.77206
25	H	-5.74078	1.423127	-1.38457
26	H	-5.19426	1.175456	0.278341
27	C	-4.42945	-0.28258	-1.07073

28	N	-3.29874	-0.55846	-1.64218
29	N	-3.19536	-1.93529	-1.60072
30	C	-4.25349	-2.53117	-0.94024
31	N	-5.08625	-1.425	-0.63751
32	O	1.600294	2.135068	1.941661
33	H	2.099184	1.811844	2.703913
34	C	-1.9266	-2.59113	-1.85007
35	H	-1.47159	-2.07644	-2.71167
36	H	-2.1588	-3.62067	-2.13174
37	N	-1.07364	-2.61717	-0.67216
38	N	1.279994	-2.48542	0.988262
39	C	-0.46169	-1.32001	-0.36412
40	H	-1.23916	-0.55881	-0.28098
41	H	0.233566	-1.00611	-1.16805
42	C	0.281742	-1.41539	0.970712
43	H	-0.4545	-1.64655	1.750738
44	H	0.742532	-0.46102	1.231145
45	C	0.666681	-3.77226	0.627021
46	H	-0.05654	-4.0363	1.4081
47	H	1.424534	-4.55064	0.588672
48	C	-0.07006	-3.68182	-0.71642
49	H	0.672332	-3.52598	-1.52408
50	H	-0.57576	-4.63492	-0.90926
51	C	2.616409	-2.22103	0.704779
52	C	3.055538	-1.00532	0.152616
53	C	3.632136	-3.15333	1.047629
54	C	4.420179	-0.73254	-0.05092
55	H	2.311384	-0.28167	-0.14066
56	C	4.964324	-2.90928	0.835715
57	C	5.394838	-1.6925	0.284381
58	H	5.714496	-3.63962	1.117673
59	C	6.164208	0.713186	-0.80251
60	H	6.421271	1.666551	-1.23737
61	C	7.17305	-0.16567	-0.488
62	F	3.273363	-4.32262	1.636571
63	N	4.841799	0.489087	-0.61086
64	C	6.850995	-1.47553	0.094886
65	O	7.66834	-2.3378	0.409383
66	C	8.568493	0.23475	-0.75441
67	O	9.569235	-0.41242	-0.54007
68	O	8.654758	1.495111	-1.30529
69	H	9.60652	1.637098	-1.43211
70	C	-6.35054	-1.5323	0.008631
71	C	-6.47674	-2.33974	1.144349
72	C	-7.46196	-0.85201	-0.50055
73	C	-7.71477	-2.44574	1.7749
74	H	-5.61524	-2.88494	1.509608
75	C	-8.69133	-0.95021	0.150559

76	H	-7.37132	-0.27139	-1.41276
77	C	-8.82129	-1.74625	1.288888
78	H	-7.812	-3.0751	2.654161
79	H	-9.55007	-0.41641	-0.24486
80	H	-9.78121	-1.82859	1.789175
81	C	3.821292	1.489685	-0.99005
82	C	4.355046	2.786615	-1.58672
86	H	3.230538	1.718358	-0.09647
84	H	3.143923	1.016873	-1.71044
85	H	3.498107	3.424753	-1.82118
86	H	4.995627	3.334805	-0.89013
87	H	4.905706	2.623712	-2.51734
88	O	-4.44218	-3.70874	-0.6936

Table S15. Optimized structure for Compound **13** and Cartesian Z-matrix.

**Compound 13**		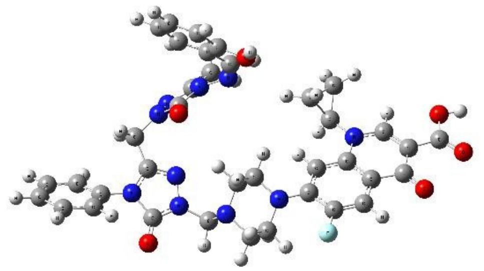		
Center Number	Atom	Standard orientation: Coordinates (Angstroms)		
		X	Y	Z
1	N	-3.62691	1.421788	-1.59585
2	N	-3.22877	1.856527	-2.85081
3	C	-2.14587	2.544282	-2.63804
4	C	-1.34467	3.227341	-3.68776
5	H	-1.33445	4.312158	-3.53902
6	H	-0.30615	2.884042	-3.66225
7	H	-1.77811	3.003832	-4.66336
8	N	-1.8404	2.607841	-1.28493
9	N	-0.58536	3.007697	-0.83012
10	C	-0.49915	3.600012	0.314266
11	H	0.537682	3.769303	0.600216
12	C	-1.47993	4.139273	1.261041
13	C	-2.76022	4.59392	0.898811
14	C	-1.06044	4.33336	2.596652
15	C	-3.59513	5.215285	1.819305
16	H	-3.09025	4.476289	-0.12618
17	C	-1.90403	4.944774	3.527654
18	C	-3.16614	5.383506	3.138515
19	H	-4.5753	5.565094	1.513338
20	H	-1.56842	5.072665	4.554256
21	H	-3.81483	5.858219	3.868339

22	C	-2.74886	1.772436	-0.59369
23	O	-2.75096	1.450504	0.582821
24	C	-4.66401	0.429759	-1.4508
25	H	-5.29999	0.511055	-2.33566
26	H	-5.27228	0.671525	-0.57342
27	C	-4.09989	-0.95916	-1.28372
28	N	-2.87166	-1.26899	-1.56322
29	N	-2.73342	-2.58537	-1.17148
30	C	-3.87911	-3.10173	-0.60031
31	N	-4.78875	-2.02091	-0.7165
32	O	0.186798	3.894382	2.932121
33	H	0.367146	4.114099	3.855625
34	C	-1.44024	-3.23204	-1.14875
35	H	-0.91623	-2.92516	-2.06953
36	H	-1.63287	-4.30676	-1.18941
37	N	-0.68163	-2.94677	0.060891
38	N	1.63161	-2.28825	1.663646
39	C	-0.18213	-1.56697	0.105785
40	H	-1.02571	-0.87646	0.038925
41	H	0.487103	-1.35882	-0.75203
42	C	0.556218	-1.32644	1.425607
43	H	-0.16444	-1.43763	2.245155
44	H	0.948282	-0.3087	1.472945
45	C	1.112844	-3.66222	1.59214
46	H	0.395181	-3.79742	2.410141
47	H	1.922331	-4.37526	1.729956
48	C	0.4006	-3.9159	0.258146
49	H	1.147457	-3.87987	-0.55974
50	H	-0.03078	-4.92361	0.26729
51	C	2.936858	-2.00533	1.262654
52	C	3.265725	-0.94191	0.407736
53	C	4.027895	-2.75068	1.78421
54	C	4.599578	-0.63865	0.088896
55	H	2.473026	-0.35722	-0.03284
56	C	5.332988	-2.47937	1.463077
57	C	5.653899	-1.41495	0.605506
58	H	6.142501	-3.06498	1.884051
59	C	6.205171	0.648835	-1.13367
60	H	6.350204	1.452083	-1.84273
61	C	7.284742	-0.0538	-0.6615
62	F	3.768582	-3.75921	2.654362
63	N	4.912086	0.427418	-0.77783
64	C	7.081623	-1.1632	0.284918
65	O	7.974362	-1.84663	0.779929
66	C	8.635141	0.316952	-1.13183
67	O	9.680158	-0.22921	-0.857
68	O	8.613569	1.409812	-1.969
69	H	9.543017	1.543336	-2.215

70	C	-6.14903	-2.07866	-0.30014
71	C	-6.464	-2.62809	0.947498
72	C	-7.1639	-1.60865	-1.14062
73	C	-7.79568	-2.68691	1.353047
74	H	-5.67121	-3.01498	1.575608
75	C	-8.49139	-1.6549	-0.71558
76	H	-6.91933	-1.23232	-2.12854
77	C	-8.81095	-2.19368	0.530921
78	H	-8.03879	-3.11618	2.320127
79	H	-9.2753	-1.28416	-1.36886
80	H	-9.84569	-2.23714	0.856412
81	O	-4.0756	-4.20914	-0.13207
82	C	3.857574	1.185669	-1.42255
86	C	3.014498	2.146387	-0.62006
84	C	4.019956	2.670115	-1.61231
85	H	3.355203	0.63423	-2.21551
86	H	3.259744	2.251241	0.431976
87	H	1.956322	2.208956	-0.85824
88	H	3.666721	3.093839	-2.54666
89	H	4.913415	3.140975	-1.21597

Table S16. Optimized structure for Compound **14** and Cartesian Z-matrix.

**Compound 14**		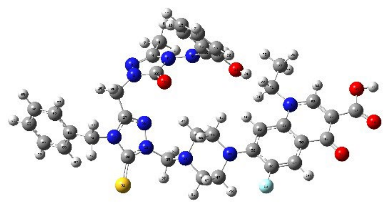		
Center Number	Atom	Standard orientation: Coordinates (Angstroms)		
		X	Y	Z
1	N	-3.63897	2.096108	-0.59674
2	N	-3.74527	2.666337	-1.85773
3	C	-2.5829	3.199027	-2.08375
4	C	-2.20518	3.938675	-3.31652
5	H	-1.95976	4.98244	-3.09475
6	H	-1.32271	3.485919	-3.77759
7	H	-3.04145	3.907856	-4.01598
8	N	-1.71864	3.026239	-1.00565
9	N	-0.34908	3.176736	-1.18191
10	C	0.417223	3.381176	-0.16686
11	H	1.468923	3.373766	-0.45302
12	C	0.172895	3.689033	1.248502
13	C	-0.86633	4.527783	1.678907
14	C	1.100529	3.230019	2.203948

15	C	-0.98239	4.903519	3.012839
16	H	-1.57625	4.900768	0.948413
17	C	0.982352	3.594238	3.545804
18	C	-0.05698	4.431283	3.946312
19	H	-1.78737	5.560694	3.324283
20	H	1.699414	3.219616	4.272619
21	H	-0.14208	4.71263	4.991367
22	C	-2.37478	2.204738	-0.05692
23	O	-1.93581	1.708946	0.967725
24	C	-4.62246	1.142861	-0.14223
25	H	-5.54347	1.33086	-0.6993
26	H	-4.82781	1.320067	0.9195
27	C	-4.14948	-0.27726	-0.31678
28	N	-3.06023	-0.60668	-0.94421
29	N	-2.95967	-1.96812	-0.77871
30	C	-3.98279	-2.49322	-0.04176
31	S	-4.26185	-4.08672	0.393102
32	N	-4.76091	-1.37706	0.245947
33	O	2.099359	2.402458	1.758785
34	H	2.677344	2.167259	2.497065
35	C	-1.72073	-2.64411	-1.13908
36	H	-1.38304	-2.17693	-2.07766
37	H	-1.97715	-3.68899	-1.32245
38	N	-0.74612	-2.57725	-0.06771
39	N	1.758728	-2.33723	1.33518
40	C	-0.09751	-1.26806	0.060118
41	H	-0.8573	-0.48933	0.149588
42	H	0.517124	-1.03807	-0.83271
43	C	0.772482	-1.25696	1.319741
44	H	0.114474	-1.40638	2.184865
45	H	1.263782	-0.2907	1.446847
46	C	1.10013	-3.64103	1.160398
47	H	0.456094	-3.81635	2.030355
48	H	1.842912	-4.43375	1.121814
49	C	0.233637	-3.66445	-0.10628
50	H	0.892808	-3.60347	-0.99453
51	H	-0.30345	-4.61818	-0.15199
52	C	3.060168	-2.12086	0.89245
53	C	3.447382	-0.96558	0.191389
54	C	4.100113	-3.03473	1.211211
55	C	4.785305	-0.73224	-0.17394
56	H	2.681635	-0.2593	-0.08834
57	C	5.404793	-2.83018	0.843383
58	C	5.783543	-1.67336	0.144726
59	H	6.175614	-3.54409	1.111413
60	C	6.451434	0.615315	-1.22531
61	H	6.668119	1.522504	-1.76766
62	C	7.481954	-0.24726	-0.9378

63	F	3.797315	-4.14169	1.935754
64	N	5.154204	0.428868	-0.88074
65	C	7.213796	-1.49576	-0.21063
66	O	8.054073	-2.33897	0.094644
67	C	8.845044	0.10708	-1.37972
68	O	9.858269	-0.53458	-1.21306
69	O	8.882007	1.313606	-2.04478
70	H	9.816399	1.429308	-2.28064
71	C	4.106091	1.405914	-1.24517
72	C	4.584219	2.636557	-2.00677
73	H	3.6083	1.723025	-0.32232
74	H	3.357271	0.880224	-1.84883
75	H	3.711954	3.26441	-2.21039
76	H	5.295277	3.234812	-1.43012
77	H	5.036854	2.383237	-2.96942
78	C	-6.00885	-1.41059	1.013431
79	H	-6.04476	-2.41729	1.441924
80	H	-5.93033	-0.69478	1.837863
81	C	-7.23985	-1.12998	0.175063
82	C	-8.08742	-0.06271	0.489881
86	C	-7.55634	-1.95543	-0.91357
84	C	-9.23388	0.182496	-0.26976
85	H	-7.85536	0.577025	1.338319
86	C	-8.69727	-1.70772	-1.67398
87	H	-6.90984	-2.79512	-1.15356
88	C	-9.53855	-0.63817	-1.35476
89	H	-9.88395	1.013828	-0.01355
90	H	-8.93447	-2.35365	-2.51411
91	H	-10.4278	-0.44877	-1.94845

Table S17. Optimized structure for Compound **15** and Cartesian Z-matrix.

**Compound 15**		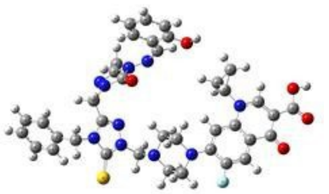		
Center Number	Atom	Standard orientation: Coordinates (Angstroms)		
		X	Y	Z
1	N	-3.85851	1.76939	-0.56938
2	N	-4.169	2.012503	-1.90067
3	C	-3.17342	2.714512	-2.3516
4	C	-3.04645	3.229449	-3.74028
5	H	-3.03909	4.324203	-3.75979
6	H	-2.10883	2.888499	-4.18885
7	H	-3.88911	2.865969	-4.32967

8	N	-2.23516	2.970886	-1.35878
9	N	-0.95688	3.412565	-1.68529
10	C	-0.29017	4.115342	-0.83426
11	H	0.72221	4.310266	-1.1852
12	C	-0.61962	4.757995	0.44346
13	C	-1.90689	5.204134	0.788321
14	C	0.443887	5.068982	1.318012
15	C	-2.13431	5.929203	1.952165
16	H	-2.73233	4.996835	0.11785
17	C	0.217947	5.784574	2.495413
18	C	-1.06888	6.213668	2.809595
19	H	-3.13582	6.270346	2.191733
20	H	1.049651	6.002174	3.161697
21	H	-1.23715	6.771063	3.725938
22	C	-2.6164	2.248277	-0.203
23	O	-2.01149	2.098877	0.845019
24	C	-4.58737	0.770515	0.167883
25	H	-5.63173	0.805712	-0.15253
26	H	-4.54391	1.033627	1.230861
27	C	-4.01737	-0.60977	-0.02998
28	N	-2.91028	-0.85319	-0.66683
29	N	-2.74075	-2.21378	-0.55869
30	C	-3.73572	-2.82423	0.148794
31	S	-3.93774	-4.44957	0.50008
32	N	-4.56714	-1.76221	0.48898
33	O	1.692799	4.632114	0.967694
34	H	2.335223	4.928583	1.625811
35	C	-1.51405	-2.83119	-1.03753
36	H	-1.20177	-2.23611	-1.9102
37	H	-1.77156	-3.84269	-1.35814
38	N	-0.51292	-2.91602	0.006568
39	N	2.027885	-2.85946	1.357591
40	C	0.118481	-1.63398	0.333251
41	H	-0.6538	-0.89084	0.550728
42	H	0.714964	-1.24932	-0.51681
43	C	1.014729	-1.82465	1.56257
44	H	0.381748	-2.15111	2.396821
45	H	1.487844	-0.88751	1.859583
46	C	1.389107	-4.13122	0.983456
47	H	0.778644	-4.46458	1.830968
48	H	2.143309	-4.88818	0.788556
49	C	0.481632	-3.96125	-0.24079
50	H	1.108597	-3.74063	-1.12695
51	H	-0.0432	-4.90449	-0.4277
52	C	3.303295	-2.52548	0.910975
53	C	3.644549	-1.232	0.480038
54	C	4.369584	-3.46609	0.949783
55	C	4.955605	-0.89858	0.105058

56	H	2.875216	-0.47864	0.413357
57	C	5.642747	-3.16438	0.539629
58	C	5.970998	-1.87231	0.100413
59	H	6.426482	-3.91279	0.574449
60	C	6.526544	0.68189	-0.77029
61	H	6.672792	1.696407	-1.11455
62	C	7.560919	-0.21746	-0.82165
63	F	4.129512	-4.71339	1.43059
64	N	5.279892	0.410869	-0.30038
65	C	7.359526	-1.59677	-0.34485
66	O	8.224634	-2.46805	-0.30848
67	C	8.863318	0.226884	-1.35894
68	O	9.852267	-0.45009	-1.53
69	O	8.866318	1.56673	-1.67645
70	H	9.757018	1.729409	-2.02638
71	C	-5.8053	-1.89316	1.263071
72	H	-5.76297	-2.90151	1.687248
73	H	-5.77443	-1.17593	2.089196
74	C	-7.06316	-1.7044	0.438273
75	C	-7.99898	-0.72441	0.786078
76	C	-7.31728	-2.53057	-0.66586
77	C	-9.17146	-0.56564	0.043466
78	H	-7.81519	-0.08576	1.647154
79	C	-8.4846	-2.3684	-1.40915
80	H	-6.60198	-3.30432	-0.93047
81	C	-9.41415	-1.3858	-1.05738
82	H	-9.88977	0.198625	0.325009
86	H	-8.67289	-3.01392	-2.26188
84	H	-10.3234	-1.26323	-1.63832
85	C	4.271209	1.451579	-0.29892
86	C	3.75767	1.977648	1.018715
87	C	4.64183	2.839357	0.15328
88	H	3.554894	1.368058	-1.11411
89	H	4.198508	1.555809	1.916071
90	H	2.704037	2.226154	1.089986
91	H	4.192821	3.668199	-0.38425
92	H	5.65884	3.01381	0.488479

Table S18. Optimized structure for Compound **16** and Cartesian Z-matrix.

**Compound 16**		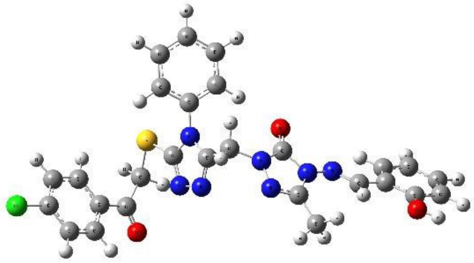		
Center Number	Atom	Standard orientation: Coordinates (Angstroms)		
		X	Y	Z
1	C	9.152755	-0.57417	-2.19368
2	C	7.930772	-0.00961	-2.57374
3	C	6.851185	-0.05785	-1.701
4	C	6.963928	-0.6614	-0.4364
5	C	8.203228	-1.2264	-0.06943
6	C	9.289773	-1.1805	-0.94759
7	H	10.00296	-0.54451	-2.86845
8	H	7.824846	0.461727	-3.54522
9	H	5.892839	0.371295	-1.97267
10	H	10.23928	-1.62086	-0.65092
11	O	8.293481	-1.81206	1.163562
12	H	9.188501	-2.15318	1.28877
13	C	5.841047	-0.7132	0.500083
14	H	6.028592	-1.19461	1.456975
15	N	4.710425	-0.19165	0.178076
16	N	3.662316	-0.26752	1.062854
17	C	3.288871	-1.20175	2.036629
18	C	2.603182	0.656382	0.915694
19	C	4.042913	-2.4211	2.447323
20	H	4.370618	-3.00003	1.578409
21	H	4.924831	-2.18875	3.053336
22	H	3.367805	-3.0364	3.0443
23	N	2.118031	-0.91328	2.523098
24	N	1.71591	0.238291	1.879295
25	O	2.519506	1.601047	0.14724
26	C	0.371367	0.752611	2.057367
27	H	0.014515	0.335124	3.001161
28	H	0.416778	1.83974	2.151921
29	C	-2.00461	0.45777	-0.67982
30	N	-1.29699	1.267074	0.181648
31	N	-0.81589	-0.85953	0.565344
32	C	-0.56238	0.368317	0.940496
33	N	-1.73002	-0.80722	-0.46799
34	C	-1.33108	2.699775	0.253973
35	C	-2.5265	3.336738	0.602196
36	C	-0.17679	3.436341	-0.02814

37	C	-2.56906	4.729471	0.655102
38	H	-3.40611	2.743935	0.831264
39	C	-0.23123	4.828872	0.045379
40	H	0.744839	2.919822	-0.27943
41	C	-1.42224	5.476277	0.378966
42	H	-3.49607	5.22815	0.920674
43	H	0.662872	5.406751	-0.16717
44	H	-1.45687	6.560524	0.426381
45	S	-3.10851	1.08871	-1.90231
46	C	-3.38002	-0.44622	-2.87574
47	H	-4.05036	-0.11747	-3.67899
48	H	-2.43749	-0.76376	-3.32202
49	C	-3.97566	-1.69439	-2.20264
50	O	-3.53594	-2.7757	-2.54832
51	C	-5.11008	-1.6085	-1.23203
52	C	-5.32081	-2.71111	-0.38973
53	C	-6.00142	-0.52842	-1.17302
54	C	-6.38148	-2.73194	0.50812
55	H	-4.63242	-3.54654	-0.45205
56	C	-7.08153	-0.54408	-0.292
57	H	-5.86899	0.33133	-1.81916
58	C	-7.25591	-1.64398	0.546154
59	H	-6.53628	-3.57742	1.168761
60	H	-7.7786	0.28522	-0.25362
61	C l	-8.60541300	-1.66216	1.671377

Table S19. Optimized structure for Compound **17** and Cartesian Z-matrix.

**Compound 17**		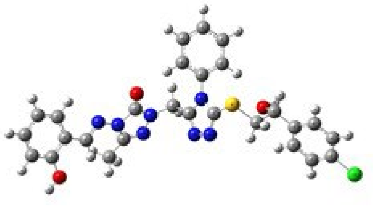		
Center Number	Atom	Standard orientation: Coordinates (Angstroms)		
		X	Y	Z
1	C	-9.2645	-2.107	1.651988
2	C	-9.68172	-0.87211	2.141864
3	C	-8.92104	0.276812	1.901344
4	C	-7.74466	0.180205	1.169059
5	C	-7.29942	-1.05476	0.666326
6	C	-8.07941	-2.2032	0.916821
7	H	-9.85576	-3.00118	1.837217
8	H	-10.6036	-0.80878	2.712128
9	H	-9.24684	1.238311	2.284174
10	H	-7.13595	1.055681	0.970613
11	O	-7.63373	-3.39452	0.414785

12	H	-8.24134	-4.0982	0.676978
13	C	-6.05519	-1.17847	-0.09298
14	H	-5.77889	-2.17977	-0.4163
15	N	-5.33781	-0.13787	-0.33169
16	N	-4.18502	-0.29115	-1.0674
17	C	-3.14512	0.648425	-0.89057
18	C	-3.84109	-1.12431	-2.13654
19	N	-2.67835	-0.79571	-2.61821
20	C	-4.68356	-2.19705	-2.73963
21	H	-4.73032	-3.09451	-2.11441
22	H	-5.70763	-1.85329	-2.91597
23	H	-4.23268	-2.47056	-3.69498
24	N	-2.25024	0.276795	-1.86503
25	O	-3.06667	1.548641	-0.06949
26	C	-0.91987	0.827913	-2.04356
27	H	-0.58174	0.477444	-3.02087
28	H	-0.98614	1.917711	-2.06651
29	C	1.501981	0.383019	0.635745
30	C	0.050486	0.38838	-0.98027
31	N	0.350332	-0.86167	-0.7168
32	N	1.253108	-0.86776	0.320792
33	N	0.761256	1.238472	-0.15484
34	C	0.790137	2.674242	-0.1693
35	C	1.979035	3.325162	-0.5132
36	C	-0.36411	3.393704	0.150418
37	C	2.008408	4.718534	-0.53138
38	H	2.858098	2.733771	-0.74662
39	C	-0.32051	4.788917	0.115026
40	H	-1.27965	2.864946	0.39845
41	C	0.860745	5.451728	-0.2201
42	H	2.928557	5.232432	-0.79336
43	H	-1.21524	5.354699	0.355946
44	H	0.888305	6.537234	-0.23984
45	S	2.58372	0.912807	1.928874
46	C	4.044738	-0.14689	1.55721
47	H	4.513718	-0.35235	2.522452
48	H	3.667182	-1.08539	1.146252
49	C	5.072329	0.493451	0.615692
50	H	5.408375	1.441921	1.062734
51	O	4.416511	0.738737	-0.62746
52	H	5.082676	1.060183	-1.25003
53	C	6.274126	-0.42308	0.44685
54	C	7.438192	-0.2239	1.196685
55	C	6.220488	-1.50367	-0.44355
56	C	8.530348	-1.08212	1.071135
57	H	7.499557	0.614513	1.886251
58	C	7.304134	-2.36842	-0.58411
59	H	5.323732	-1.65926	-1.03519

60	C	8.451504	-2.14947	0.178252
61	H	9.43265	-0.92269	1.650712
62	H	7.261724	-3.20335	-1.27457
63	Cl	9.82535700	-3.23361	0.004521

Table S20. Optimized structure for Compound **18** and Cartesian Z-matrix.

**Compound 18**		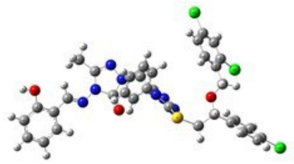		
Center Number	Atom	Standard orientation: Coordinates (Angstroms)		
		X	Y	Z
1	C	-10.3609	-2.04171	0.318744
2	C	-10.1167	-3.34542	-0.10566
3	C	-8.87386	-3.69034	-0.64662
4	C	-7.88222	-2.72418	-0.76047
5	C	-8.10243	-1.40173	-0.33731
6	C	-9.36157	-1.07112	0.205568
7	H	-11.3273	-1.77138	0.739056
8	H	-10.9	-4.09172	-0.01365
9	H	-8.6843	-4.70615	-0.97732
10	H	-6.91052	-2.96654	-1.17711
11	O	-9.55921	0.22148	0.609916
12	H	-10.4548	0.310422	0.960792
13	C	-7.06698	-0.37269	-0.44072
14	H	-7.3285	0.616644	-0.07333
15	N	-5.91638	-0.66893	-0.9331
16	N	-4.95329	0.300446	-1.04283
17	C	-3.60521	-0.12609	-1.19611
18	C	-4.99003	1.689001	-1.1852
19	C	-6.19822	2.563875	-1.20157
20	H	-6.96512	2.17848	-1.88061
21	H	-6.64946	2.676525	-0.21024
22	H	-5.8839	3.549694	-1.54852
23	N	-3.79308	2.160129	-1.37687
24	N	-2.94167	1.073568	-1.34385
25	O	-3.17234	-1.26196	-1.1502
26	C	-1.54529	1.292923	-1.70508
27	H	-1.42424	1.313493	-2.79308
28	H	-1.2962	2.286238	-1.31837
29	C	0.639618	-1.02128	0.044185
30	C	-0.58799	0.260505	-1.20567
31	N	0.237405	-0.38704	-1.98868
32	N	1.015695	-1.20675	-1.19836

33	N	-0.36977	-0.08727	0.114219
34	C	-1.04629	0.37729	1.289767
35	C	-0.65837	1.585753	1.873404
36	C	-2.07577	-0.39007	1.841385
37	C	-1.31375	2.032551	3.020677
38	H	0.153802	2.155252	1.432762
39	C	-2.7186	0.062899	2.993519
40	H	-2.37544	-1.30748	1.346576
41	C	-2.3404	1.271139	3.582508
42	H	-1.01934	2.972371	3.477933
43	H	-3.51949	-0.52809	3.427138
44	H	-2.84554	1.619813	4.478363
45	S	1.263524	-1.84316	1.478803
46	C	2.857543	-2.45269	0.789349
47	H	2.997921	-3.46274	1.18012
48	H	2.734184	-2.50569	-0.29422
49	C	4.065915	-1.58245	1.161716
50	H	4.149277	-1.54038	2.260759
51	O	3.818336	-0.27837	0.656695
52	C	5.339803	-2.19654	0.597036
53	C	6.208554	-2.92877	1.411739
54	C	5.636087	-2.06817	-0.76643
55	C	7.351615	-3.53185	0.885811
56	H	5.99641	-3.03151	2.473306
57	C	6.776132	-2.65916	-1.30672
58	H	4.973218	-1.48909	-1.40158
59	C	7.623628	-3.38956	-0.47315
60	H	8.024788	-4.09756	1.520097
61	H	7.006892	-2.55703	-2.36118
62	Cl	9.06563100	-4.13883	-1.14815
63	C	4.686302	0.719745	1.187378
64	H	4.671287	0.680628	2.287291
65	H	5.71935	0.549974	0.859715
66	C	4.192207	2.069635	0.7334
67	C	4.52918	2.633521	-0.50519
68	C	3.329937	2.805179	1.5566
69	C	4.038916	3.874897	-0.91146
70	C	2.824185	4.047915	1.182886
71	H	3.050431	2.386638	2.519152
72	C	3.187225	4.570004	-0.05729
73	H	4.317423	4.286619	-1.87324
74	H	2.16752	4.605793	1.840382
75	Cl	5.60816100	1.7942	-1.60845
76	Cl	2.56768300	6.135266	-0.55593

Table S21. Optimized structure for Compound **19** and Cartesian Z-matrix.

**Compound 19**		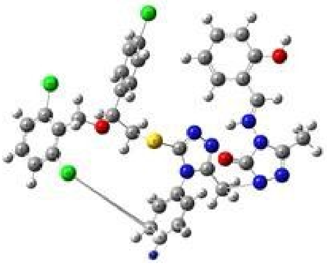		
Center Number	Atom	Standard orientation: Coordinates (Angstroms)		
		X	Y	Z
1	C	5.042759	3.812698	-0.04961
2	C	4.326054	4.997865	-0.2135
3	C	2.952862	4.951132	-0.44565
4	C	2.287366	3.723453	-0.53033
5	C	2.992067	2.538212	-0.37171
6	C	4.379756	2.560866	-0.11174
7	H	4.843664	5.952205	-0.15153
8	H	2.399687	5.877461	-0.56486
9	H	1.222676	3.691682	-0.7315
10	H	2.472588	1.589033	-0.49694
11	O	6.384911	3.79786	0.190613
12	H	6.72545	4.702071	0.183596
13	C	5.176103	1.376475	0.089832
14	H	6.254559	1.457014	0.043961
15	N	4.686568	0.196965	0.34978
16	H	3.680475	0.011941	0.521645
17	N	5.350218	-0.95946	0.516814
18	C	6.54762	-1.48986	0.031466
19	N	6.513953	-2.77722	0.239133
20	C	4.555432	-2.09062	0.989072
21	O	3.401656	-1.87239	1.406722
22	C	7.679322	-0.73651	-0.57804
23	H	7.386546	-0.19449	-1.48586
24	H	8.137263	-0.01896	0.115171
25	H	8.439765	-1.46961	-0.85379
26	N	2.651638	-1.13857	-1.76107
27	N	1.690435	-0.42648	-1.07497
28	N	1.288735	-2.57466	-0.79294
29	C	0.768686	-3.80906	-0.27212
30	C	-0.24456	-4.4771	-0.96152
31	C	1.308995	-4.32432	0.907972
32	C	-0.73069	-5.68338	-0.45751
33	H	-0.64132	-4.05345	-1.87901
34	C	0.814747	-5.53294	1.399339
35	H	2.099547	-3.77174	1.408811
36	C	-0.20092	-6.21073	0.721806
37	H	-1.52011	-6.20942	-0.98575
38	H	1.228024	-5.94511	2.31471

39	H	-0.57959	-7.15109	1.111516
40	C	0.882968	-1.28994	-0.49668
41	S	-0.50043	-0.89051	0.522159
42	C	-1.70814	-0.41496	-0.80312
43	H	-1.1478	0.073526	-1.60254
44	H	-2.17964	-1.3238	-1.18555
45	C	-2.80261	0.528462	-0.28375
46	H	-3.54733	0.593033	-1.09283
47	O	-3.37616	-0.10756	0.854336
48	C	-2.31741	1.940906	0.017022
49	C	-1.85799	2.301401	1.288859
50	C	-2.31445	2.90512	-0.9972
51	C	-1.40134	3.593996	1.545774
52	H	-1.86339	1.564537	2.084055
53	C	-1.85603	4.200925	-0.76121
54	H	-2.68383	2.64883	-1.987
55	C	-1.4034	4.532107	0.515154
56	H	-1.04702	3.870018	2.53254
57	H	-1.86425	4.946131	-1.5488
58	C	-4.6807	0.342965	1.220174
59	H	-4.72894	1.434863	1.226336
60	H	-4.82745	-0.01352	2.242099
61	C	-5.76937	-0.22905	0.32905
62	C	-6.51358	0.543912	-0.57869
63	C	-6.09407	-1.59882	0.376303
64	C	-7.50994	0.003644	-1.3919
65	C	-7.08275	-2.16615	-0.4241
66	C	-7.78843	-1.35703	-1.31149
67	H	-8.05369	0.646993	-2.07343
68	H	-7.29357	-3.22614	-0.34489
69	Cl	-0.83195	6.169449	0.830331
70	Cl	-6.22334	2.279175	-0.72935
71	H	-8.56137	-1.78857	-1.93942
72	N	5.312541	-3.17441	0.814253
73	C	3.257822	-3.54479	-2.0578
74	H	2.652155	-4.3488	-2.48542
75	H	3.936894	-3.161	-2.82076
76	H	3.856815	-3.95573	-1.23651
77	C	2.412151	-2.42068	-1.56725
78	Cl	-5.24952	-2.66129	1.491699

Table S22. Optimized structure for Compound **20** and Cartesian Z-matrix.

**Compound 20**		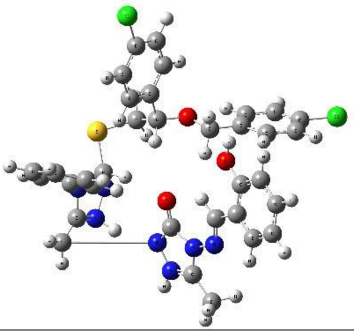		
Center Number	Atom	Standard orientation: Coordinates (Angstroms)		
		X	Y	Z
1	C	4.519732	-1.86541	4.830473
2	C	4.356097	-3.15588	4.315162
3	C	3.499175	-3.36023	3.24203
4	C	2.790216	-2.29547	2.657854
5	C	2.975062	-0.99941	3.186402
6	C	3.835326	-0.79134	4.268808
7	H	5.183788	-1.69278	5.671923
8	H	4.890052	-3.99168	4.75496
9	H	3.350145	-4.35378	2.833688
10	H	3.967859	0.213049	4.664285
11	O	2.301938	0.029642	2.590782
12	H	2.449687	0.845838	3.086779
13	C	1.865067	-2.48599	1.542642
14	H	1.243356	-1.65054	1.23673
15	N	1.799952	-3.63656	0.957517
16	C	-0.10151	-2.87155	-0.65143
17	C	0.846557	-4.94518	-0.75292
18	N	0.901079	-3.77681	-0.07909
19	N	-0.09876	-4.78442	-1.66221
20	H	-0.39512	-5.46837	-2.34203
21	N	-0.70262	-3.55311	-1.64895
22	O	-0.29746	-1.72376	-0.23751
23	C	1.68133	-6.14812	-0.51396
24	H	2.741379	-5.90763	-0.63509
25	H	1.54293	-6.51124	0.508385
26	H	1.412905	-6.94226	-1.21416
27	C	-4.89471	-3.53761	-1.30719
28	H	-4.89076	-4.46255	-1.86907
29	H	-5.43431	-3.49915	-0.37067
30	C	-4.24366	-2.47053	-1.78772
31	C	-3.06833	-0.48692	-1.9048
32	H	-2.12214	-0.67195	-1.38358
33	N	-4.20093	-1.16339	-1.25336
34	N	-3.42747	-2.48448	-2.96673
35	H	-2.57607	-3.01433	-2.77163
36	N	-3.01757	-1.12468	-3.21925

37	H	-3.75513	-0.70851	-3.78893
38	C	-4.50809	-0.94352	0.125122
39	C	-5.82819	-0.65202	0.482371
40	C	-3.51976	-1.02193	1.117213
41	C	-6.16275	-0.43858	1.820103
42	H	-6.57807	-0.5884	-0.29923
43	C	-3.85831	-0.78723	2.450967
44	H	-2.4979	-1.27143	0.845176
45	C	-5.17786	-0.49878	2.806975
46	H	-7.19075	-0.21275	2.088411
47	H	-3.08911	-0.84329	3.216458
48	H	-5.4363	-0.32271	3.84717
49	S	-3.3465	1.32264	-2.01379
50	C	-1.6122	1.83647	-2.32829
51	H	-1.66597	2.835407	-2.76921
52	H	-1.17754	1.159858	-3.0673
53	C	-0.70742	1.849642	-1.08059
54	H	-0.8104	0.886974	-0.56402
55	O	0.625223	1.962629	-1.58817
56	C	-1.0259	2.976773	-0.1085
57	C	-0.44969	4.241125	-0.2836
58	C	-1.91069	2.782287	0.958354
59	C	-0.74798	5.293402	0.580736
60	H	0.252445	4.392541	-1.0972
61	C	-2.22542	3.82648	1.828844
62	H	-2.3678	1.809569	1.112055
63	C	-1.63818	5.074051	1.630929
64	H	-0.29642	6.269939	0.444789
65	H	-2.91475	3.672073	2.651403
66	C	1.604276	1.438266	-0.70526
67	H	1.561867	1.953423	0.267434
68	H	1.392519	0.375363	-0.5131
69	C	2.98455	1.59387	-1.30039
70	C	3.180508	2.063067	-2.6016
71	C	4.103072	1.241918	-0.53396
72	C	4.466588	2.18316	-3.13224
73	H	2.318799	2.335586	-3.19926
74	C	5.392225	1.352814	-1.04947
75	H	3.963051	0.872553	0.479072
76	C	5.56154	1.825925	-2.35065
77	H	4.615905	2.549449	-4.14197
78	H	6.255625	1.079019	-0.45324
79	Cl	-2.01824900	6.396174	2.73333
80	Cl	7.18899300	1.972957	-3.01211

Table S23. Optimized structure for Reference Drug Ampicillin and Cartesian Z-matrix.

**Ampicillin**		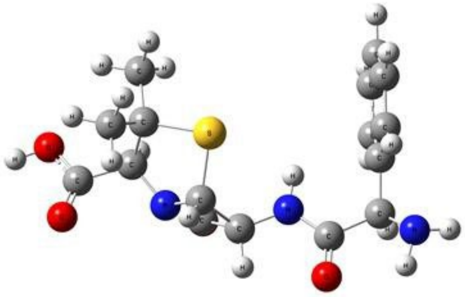		
Center Number	Atom	Standard orientation: Coordinates (Angstroms)		
		X	Y	Z
1	S	-0.89378	0.901927	1.07021
2	O	-1.07698	-1.48623	-2.30507
3	O	-5.24602	0.515388	-1.04199
4	O	-4.8895	-1.37248	0.143211
5	O	2.271097	-2.99264	0.787756
6	N	-2.09164	-1.05631	-0.20069
7	N	1.237278	-1.17577	-0.11692
8	N	4.65545	-1.72501	0.911521
9	C	-1.13889	-0.91379	0.91135
10	C	-2.67986	1.204431	0.572149
11	C	-2.99676	0.047964	-0.46505
12	C	-0.08307	-1.68898	0.051938
13	C	-1.09699	-1.46133	-1.10162
14	C	-3.57227	1.12927	1.822968
15	C	-2.7893	2.583333	-0.08758
16	C	-4.45919	-0.38721	-0.4031
17	C	2.34022	-1.89221	0.254212
18	C	3.715606	-1.27913	-0.11116
19	C	3.710858	0.23609	-0.25499
20	C	3.530923	0.829806	-1.51136
21	C	3.863464	1.059891	0.868747
22	C	3.491737	2.21991	-1.6441
23	C	3.827726	2.446954	0.735758
24	C	3.639378	3.031547	-0.51955
25	H	-1.44754	-1.3671	1.854759
26	H	-2.80269	0.410493	-1.48066
27	H	0.000693	-2.73433	0.358765
28	H	-3.54455	0.141058	2.288883
29	H	-3.24208	1.865543	2.559029
30	H	-4.61347	1.352689	1.564601
31	H	-2.54392	3.37516	0.626227
32	H	-2.11564	2.669619	-0.94421
33	H	-3.81492	2.745589	-0.43337
34	H	1.361467	-0.25132	-0.50721
35	H	3.921373	-1.70295	-1.11322
36	H	-6.15996	0.195058	-0.95212

37	H	5.603829	-1.70383	0.549532
38	H	4.421566	-2.68458	1.156496
39	H	3.430382	0.200599	-2.39314
40	H	4.027953	0.59317	1.834147
41	H	3.356183	2.6647	-2.62566
42	H	3.949051	3.07501	1.613782
43	H	3.614899	4.112691	-0.62023

Table S24. Optimized structure for Reference Drug Cefatrizine and Cartesian Z-matrix.

**Cefatrizine**		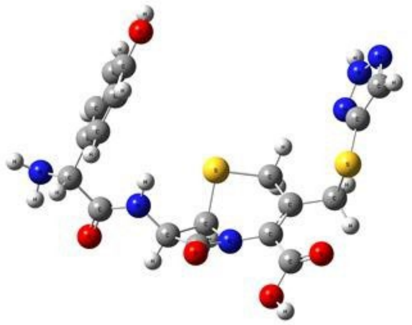		
Center Number	Atom	Standard orientation: Coordinates (Angstroms)		
		X	Y	Z
1	S	-0.09284	-0.02259	-1.46387
2	S	4.326768	0.178025	0.897434
3	O	-0.77883	-2.79611	1.833011
4	O	1.760431	-4.24739	1.111093
5	O	-4.34344	-2.24137	-1.36715
6	O	3.436049	-2.81908	1.581801
7	O	-2.61792	4.613148	1.580067
8	N	0.373732	-2.40354	-0.224
9	N	-2.69956	-1.20668	-0.17906
10	N	-5.97811	-0.08779	-1.18709
11	N	4.235685	2.003461	-1.17616
12	N	4.71271	3.240791	-1.35658
13	N	5.400447	3.763568	-0.35104
14	C	-0.42811	-1.8165	-1.29588
15	C	-1.70855	-2.19108	-0.47274
16	C	-0.7105	-2.55146	0.658494
17	C	1.738191	-2.16322	0.008478
18	C	2.399266	-1.17641	-0.64812
19	C	1.724006	-0.24002	-1.62708
20	C	3.883049	-0.95555	-0.51014
21	C	-3.98227	-1.32474	-0.6391
22	C	2.409658	-3.07408	0.980373
23	C	-4.99784	-0.27685	-0.12172
24	C	-4.38169	1.041468	0.31891
25	C	-4.06133	1.272971	1.664276
26	C	-4.10248	2.049232	-0.61312

27	C	4.657169	1.694176	0.054965
28	C	-3.46873	2.465043	2.074758
29	C	-3.51465	3.246999	-0.2155
30	C	-3.19247	3.45865	1.130838
31	C	5.382445	2.796753	0.566567
32	H	-0.30061	-2.31839	-2.25833
33	H	-2.21802	-3.05959	-0.89737
34	H	-2.45851	-0.40535	0.388577
35	H	2.15693	0.758603	-1.517
36	H	1.955959	-0.56081	-2.65137
37	H	4.290509	-0.50269	-1.41617
38	H	4.414128	-1.87864	-0.29033
39	H	-5.41786	-0.76091	0.781583
40	H	-6.16251	-0.99449	-1.61057
41	H	-6.84591	0.272778	-0.80222
42	H	2.229837	-4.74133	1.804103
43	H	-4.28529	0.511452	2.408092
44	H	-4.37165	1.886269	-1.65131
45	H	-3.2247	2.644938	3.116187
46	H	-3.30683	4.02192	-0.95078
47	H	5.873089	2.915812	1.520351
48	H	4.572305	3.739534	-2.22181
49	H	-2.47936	5.209417	0.832517

Table S25. Optimized structure for Reference Drug Fluconazole and Cartesian Z-matrix.

**Fluconazole**		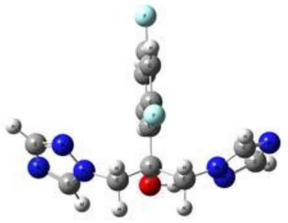		
Center Number	Atom	Standard orientation: Coordinates (Angstroms)		
		X	Y	Z
1	F	0.166126	0.756086	2.32106
2	F	-0.16996	4.4654	-0.54274
3	O	0.003567	-1.84712	-1.0666
4	N	-2.4571	-1.3182	0.312323
5	N	2.527292	-1.19848	0.318424
6	N	-3.26231	-0.3526	0.838751
7	N	2.777941	-1.54869	-0.97425
8	N	-4.16122	-1.19526	-1.07115
9	N	4.463258	-0.27219	-0.16278
10	C	0.036476	-1.16525	0.174791

11	C	-1.20469	-1.66196	0.957251
12	C	1.294612	-1.61511	0.969296
13	C	-0.00184	0.356765	-0.01766
14	C	0.053376	1.249303	1.05576
15	C	-0.13139	0.920663	-1.29166
16	C	0.00293	2.627468	0.914546
17	C	-0.18778	2.300103	-1.48602
18	C	-3.01237	-1.8013	-0.82469
19	C	3.543301	-0.43524	0.775027
20	C	-0.11816	3.131431	-0.37549
21	C	-4.26324	-0.32292	-0.02828
22	C	3.946653	-0.96497	-1.21007
23	H	-1.23696	-1.21372	1.949866
24	H	-1.13739	-2.75026	1.049705
25	H	1.295869	-1.18907	1.972037
26	H	1.287146	-2.70723	1.038785
27	H	0.909886	-1.83603	-1.43626
28	H	-0.20098	0.250679	-2.13984
29	H	0.047569	3.279806	1.777619
30	H	-0.28995	2.730708	-2.47525
31	H	-2.5387	-2.56466	-1.42331
32	H	3.569317	-0.03351	1.778339
33	H	-5.10024	0.349481	0.099405
34	H	4.43809	-1.04863	-2.16921

Table S26. Optimized structure for Reference Drug Gemcitabine and Cartesian Z-matrix.

**Gemcitabine**		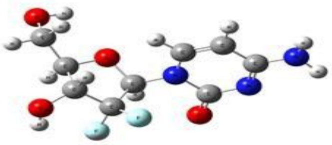		
Center Number	Atom	Standard orientation: Coordinates (Angstroms)		
		X	Y	Z
1	F	-0.13855	-1.74215	1.18939
2	F	-1.4883	-2.32548	-0.4517
3	O	-1.15973	0.895655	-0.71434
4	O	-3.27336	-1.31506	1.221959
5	O	-3.4016	2.669406	-0.91039
6	O	1.796272	-1.6159	-1.61594
7	N	1.027671	0.124736	-0.34334
8	N	3.369062	-0.34949	-0.5191

9	N	4.884304	0.929169	0.615864
10	C	-2.19112	-0.50084	0.861646
11	C	-0.99524	-1.25525	0.260903
12	C	-2.47445	0.520462	-0.24969
13	C	-0.32124	-0.25249	-0.72262
14	C	-3.19661	1.785399	0.173717
15	C	1.272623	1.13142	0.538507
16	C	2.097089	-0.68732	-0.88027
17	C	2.541941	1.436285	0.90925
18	C	3.588566	0.642982	0.323069
19	H	-1.85071	0.021303	1.7635
20	H	-3.03118	0.035819	-1.06584
21	H	-0.25417	-0.72168	-1.70743
22	H	-2.62977	2.261742	0.991468
23	H	-4.18275	1.514581	0.562768
24	H	-3.32234	-2.02992	0.569543
25	H	0.405312	1.677493	0.888159
26	H	-2.53271	2.842725	-1.29891
27	H	2.746174	2.25042	1.592812
28	H	5.120812	1.541096	1.378566
29	H	5.583722	0.287356	0.274809

Table S27. Optimized structure for Reference Drug Itraconazole and Cartesian Z-matrix.

**Itraconazole**		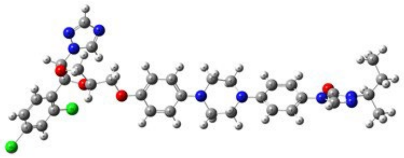		
Center Number	Atom	Standard orientation: Coordinates (Angstroms)		
		X	Y	Z
1	Cl	-7.38515	-1.22628	-2.33063
2	Cl	-11.6682	-3.92628	-0.50454
3	O	-6.54346	0.499128	0.062024
4	O	-8.18911	1.45828	1.317858
5	O	-3.89186	-0.30694	0.992419
6	O	10.66897	0.178665	-2.00687
7	N	1.6983	-0.3194	0.606016
8	N	4.580958	-0.37881	0.463136
9	N	-7.27074	3.02681	-0.9695
10	N	10.2204	-0.45417	0.241151
11	N	-7.61212	4.135729	-0.25604
12	N	12.29949	-0.36074	-0.40291

13	N	-5.48757	4.313473	-1.04924
14	N	12.32638	-0.73798	0.922145
15	C	-7.9145	0.852242	0.061003
16	C	-6.08053	0.500153	1.419474
17	C	-6.97334	1.565144	2.057406
18	C	2.423584	-1.57295	0.479575
19	C	2.483595	0.90312	0.64207
20	C	3.828671	-1.4644	1.077186
21	C	3.814356	0.728184	-0.0929
22	C	-8.15869	1.886443	-1.06638
23	C	-8.82986	-0.37089	-0.11484
24	C	-4.60093	0.823528	1.460848
25	C	0.308896	-0.28916	0.713972
26	C	5.96631	-0.40417	0.40062
27	C	-8.67331	-1.32561	-1.13366
28	C	-9.90305	-0.55006	0.768601
29	C	-0.36772	0.853402	1.17845
30	C	-0.4795	-1.40598	0.35514
31	C	-2.51977	-0.22923	0.926595
32	C	6.720878	-1.30476	1.184124
33	C	6.680993	0.46449	-0.45485
34	C	-1.761	0.888121	1.277845
35	C	-1.86263	-1.37542	0.466692
36	C	8.798564	-0.44559	0.277903
37	C	8.109158	-1.33314	1.108479
38	C	8.069124	0.453142	-0.50748
39	C	-9.53534	-2.41622	-1.25542
40	C	-10.7804	-1.62591	0.666032
41	C	-6.00176	3.154511	-1.42478
42	C	13.52982	-0.21362	-1.18048
43	C	-10.5839	-2.55598	-0.35077
44	C	11.02218	-0.1656	-0.88664
45	C	14.447	0.858818	-0.56868
46	C	-6.51233	4.870352	-0.34091
47	C	11.0767	-0.7808	1.271085
48	C	14.23283	-1.56737	-1.33011
49	C	13.81474	2.250181	-0.48952
50	H	-6.25297	-0.48632	1.870847
51	H	-7.1889	1.374321	3.111858
52	H	-6.55307	2.573261	1.947219
53	H	1.889705	-2.35731	1.026674
54	H	2.501129	-1.90512	-0.56919
55	H	1.928093	1.705002	0.142876
56	H	2.681599	1.240391	1.673665
57	H	3.75117	-1.3429	2.169995
58	H	4.344981	-2.4124	0.891734
59	H	3.622318	0.595456	-1.16986
60	H	4.384576	1.655461	0.022997

61	H	-8.01476	1.412706	-2.03828
62	H	-9.1835	2.251615	-0.99121
63	H	-4.31353	1.060538	2.497933
64	H	-4.40417	1.707566	0.838877
65	H	-10.0369	0.179364	1.557697
66	H	0.188901	1.729432	1.490468
67	H	-0.01219	-2.30076	-0.03977
68	H	6.226907	-1.98445	1.867939
69	H	6.14911	1.146164	-1.10768
70	H	-2.23127	1.7951	1.639578
71	H	-2.45805	-2.23708	0.182641
72	H	8.65396	-2.05937	1.703825
73	H	8.593646	1.115644	-1.18415
74	H	-9.38531	-3.14146	-2.04516
75	H	-11.6009	-1.74119	1.36452
76	H	-5.51097	2.389158	-2.00843
77	H	13.17602	0.12638	-2.1598
78	H	15.35811	0.898845	-1.17831
79	H	14.75203	0.526654	0.430669
80	H	-6.45065	5.848255	0.116445
81	H	10.73577	-1.01998	2.266928
82	H	14.53226	-1.95474	-0.35183
86	H	13.57134	-2.29886	-1.80289
84	H	15.12871	-1.46327	-1.94991
85	H	12.93108	2.248216	0.155729
86	H	14.52316	2.9767	-0.0794
87	H	13.50453	2.604995	-1.4784

Table S28. Optimized structure for Reference Drug Ribavirin and Cartesian Z-matrix.

**Ribavirin**		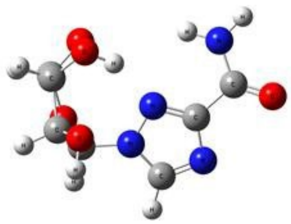		
Center Number	Atom	Standard orientation: Coordinates (Angstroms)		
		X	Y	Z
1	C	-2.34457	0.879964	-0.91994
2	O	-1.96844	-0.42688	-1.42907
3	C	-1.43126	-1.22925	-0.41154
4	C	-2.14337	-0.74009	0.876248
5	C	-2.31163	0.789541	0.639007
6	H	-3.35679	1.045637	-1.31071

7	H	-1.65072	-2.27414	-0.65143
8	H	-3.14265	-1.19933	0.84985
9	H	-3.26916	1.126244	1.045213
10	O	-1.33394	1.571953	1.246914
11	H	-0.46884	1.227562	0.955681
12	O	-1.47674	-0.99488	2.082675
13	H	-1.59892	-1.92432	2.314751
14	O	-1.46572	1.866658	-1.34985
15	H	-1.50396	1.887677	-2.31601
16	C	0.944102	-2.11122	-0.66924
17	H	0.663667	-3.10231	-0.99877
18	C	1.966935	-0.37063	-0.11737
19	N	0.031165	-1.1612	-0.34183
20	N	0.687684	-0.03514	0.014866
21	N	2.171604	-1.65201	-0.53314
22	C	3.076072	0.605248	0.154599
23	O	4.235112	0.250134	0.286748
24	N	2.654289	1.899637	0.240654
25	H	3.348583	2.610199	0.410113
26	H	1.705704	2.166474	0.02748
27	C	-2.34457	0.879964	-0.91994
28	O	-1.96844	-0.42688	-1.42907
29	C	-1.43126	-1.22925	-0.41154

Table S29. Optimized structure for Reference Drug Rufinamide and Cartesian Z-matrix.

**Rufinamide**		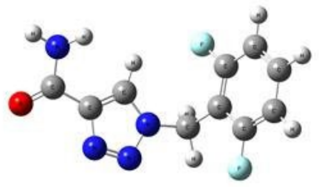		
Center Number	Atom	Standard orientation: Coordinates (Angstroms)		
		X	Y	Z
1	C	3.776957	1.059105	1.060556
2	C	3.606187	-0.32223	1.146922
3	C	2.609795	-0.91862	0.382632
4	C	1.770877	-0.19762	-0.47107
5	C	1.989272	1.180267	-0.51725
6	C	2.967031	1.828645	0.223201
7	H	4.549065	1.541812	1.650469
8	H	4.224878	-0.93919	1.787843
9	H	3.081429	2.902752	0.13721
10	F	1.190689	1.915609	-1.33103

11	F	2.449274	-2.25286	0.452943
12	C	0.700727	-0.86735	-1.3
13	H	0.906054	-1.93345	-1.3906
14	H	0.666583	-0.42675	-2.2995
15	N	-0.63613	-0.78069	-0.70951
16	C	-1.42558	0.3001	-0.51065
17	C	-2.57684	-0.21613	0.054944
18	H	-1.12598	1.294513	-0.79962
19	N	-1.26175	-1.91318	-0.28498
20	N	-2.42868	-1.57251	0.167298
21	C	-3.8306	0.474968	0.467414
22	O	-4.90995	-0.08702	0.531281
23	N	-3.67761	1.825957	0.714067
24	H	-4.49453	2.277174	1.10241
25	H	-2.78527	2.170702	1.035896

Table S30. Optimized structure for Reference Drug Streptomycin and Cartesian Z-matrix.

**Streptomycin**		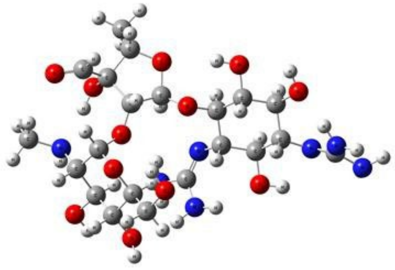		
Center Number	Atom	Standard orientation: Coordinates (Angstroms)		
		X	Y	Z
1	O	0.578067	1.387068	0.449533
2	O	-0.51111	3.260824	-0.34342
3	O	-2.21162	0.197153	0.219474
4	O	-2.55131	-1.24597	2.028424
5	O	-3.39532	2.1898	-1.19849
6	O	2.755032	3.130497	0.880686
7	O	3.618685	-1.89845	-1.32846
8	O	5.246658	2.003096	1.054273
9	O	-4.17574	-3.15649	-1.27365
10	O	-1.76856	-4.15442	-0.13327
11	O	-5.01883	2.76302	0.98918
12	O	-0.37577	-2.1518	3.492833
13	N	1.053612	-0.50817	-1.79038
14	N	5.793768	-0.39136	-0.66776
15	N	-4.74674	-0.38436	-0.85709
16	N	0.878328	-2.85689	-1.3133
17	N	-0.57708	-1.8157	-2.77938
18	N	6.848978	-0.27683	1.464301

19	N	7.94203	-1.19672	-0.38123
20	C	-1.80283	1.511549	0.521238
21	C	1.807479	1.219659	-0.28804
22	C	-2.80504	2.591792	-0.00214
23	C	-0.53106	1.836945	-0.26208
24	C	1.993571	-0.24754	-0.71093
25	C	2.889549	1.73065	0.654045
26	C	3.449467	-0.49929	-1.13581
27	C	-1.84406	3.8007	-0.23537
28	C	4.295845	1.495708	0.112104
29	C	4.502134	-0.00681	-0.11519
30	C	-3.16299	-0.41742	1.056493
31	C	-4.09976	-1.23949	0.149038
32	C	-3.28119	-2.35307	-0.50899
33	C	-2.57669	-3.1863	0.562525
34	C	-1.72753	-2.29387	1.477632
35	C	-2.18601	4.633826	-1.46029
36	C	-3.82438	2.941587	1.077482
37	C	-1.14743	-3.01778	2.687
38	C	0.524145	-1.67427	-1.9214
39	C	-6.12251	0.007849	-0.51591
40	C	6.802122	-0.56731	0.120481
41	H	-1.58562	1.633168	1.591632
42	H	1.786174	1.850504	-1.1843
43	H	-0.55718	1.387009	-1.25966
44	H	1.797515	-0.87317	0.176791
45	H	2.798338	1.185555	1.607995
46	H	3.629802	0.029934	-2.0853
47	H	-1.85131	4.446008	0.655619
48	H	4.413901	2.03046	-0.84258
49	H	4.317461	-0.51944	0.844783
50	H	-3.72734	0.322681	1.632394
51	H	-4.85186	-1.71426	0.798205
52	H	-2.51598	-1.91166	-1.16248
53	H	-3.34451	-3.70388	1.155718
54	H	-0.91222	-1.84791	0.897043
55	H	-1.43644	5.419958	-1.58534
56	H	-2.20785	4.007337	-2.35254
57	H	-3.16908	5.101204	-1.34418
58	H	-3.37749	3.369829	2.0063
59	H	-3.80062	1.293004	-1.0844
60	H	1.808139	3.300778	1.004699
61	H	-4.77083	-0.90996	-1.72763
62	H	4.582888	-2.004	-1.41319
63	H	4.895499	2.85833	1.3456
64	H	-1.97305	-3.47771	3.257381
65	H	-0.48029	-3.81791	2.348553
66	H	-3.62588	-3.82668	-1.70304

67	H	-1.65826	-4.9269	0.434629
68	H	-6.54858	0.561597	-1.35644
69	H	-6.11675	0.683179	0.341774
70	H	-6.77178	-0.85297	-0.29455
71	H	-0.92202	-1.36373	3.627633
72	H	0.133514	-3.46828	-1.00184
73	H	1.769568	-2.87397	-0.83772
74	H	-0.74102	-0.95616	-3.28953
75	H	-0.50362	-2.61931	-3.39277
76	H	6.327894	0.548321	1.744242
77	H	7.712996	-0.45562	1.952516
78	H	8.819257	-0.76355	-0.11861
79	H	7.867756	-1.32891	-1.38194

Table S31. Optimized structure for Reference Drug Tazobactam and Cartesian Z-matrix.

**Tazobactam**		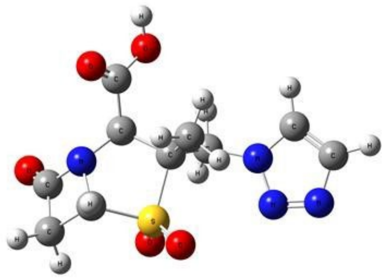		
Center Number	Atom	Standard orientation: Coordinates (Angstroms)		
		X	Y	Z
1	S	-0.28803	-1.52123	0.350901
2	O	0.459769	-2.17414	1.430436
3	O	-0.38052	-2.09131	-1.00563
4	O	-3.70745	-0.12746	-1.71941
5	O	-0.49683	3.239945	-0.442
6	O	-2.00399	2.74982	1.169302
7	N	-2.27112	0.124507	0.154563
8	N	2.589432	0.209297	-0.62229
9	N	3.019344	-1.0653	-0.46116
10	N	4.275257	-1.01573	-0.11799
11	C	0.189213	0.313996	0.15757
12	C	-2.00296	-1.10159	0.898424
13	C	-1.16921	0.973692	-0.28396
14	C	-3.14881	-1.80482	0.135144
15	C	-3.19473	-0.51113	-0.7045
16	C	1.210768	0.475269	-0.99167
17	C	0.706894	0.800904	1.513224
18	C	-1.30465	2.40002	0.25375
19	C	3.597334	1.089656	-0.37614
20	C	4.669034	0.289752	-0.05668

21	H	-2.02434	-1.04249	1.988493
22	H	-1.21098	1.030739	-1.37842
23	H	-4.03832	-2.01266	0.732396
24	H	-2.85319	-2.68199	-0.44329
25	H	1.16441	1.507541	-1.34588
26	H	0.939072	-0.19219	-1.81386
27	H	-0.07853	0.786013	2.27301
28	H	1.526389	0.166786	1.854116
29	H	1.071341	1.829095	1.427218
30	H	-0.61528	4.125689	-0.05734
31	H	3.472421	2.157921	-0.45715
32	H	5.679808	0.56647	0.201826

Table S32 Optimized Structures of Potential Antimicrobial and Anticancer Compounds (**1-20**).

Compound	Name	Structure
Compound **1**	4-{[(1E)-(2-Hydroxyphenyl)methylidene]amino}-5-methyl-2,4-dihydro-3H-1,2,4-triazol-3-one	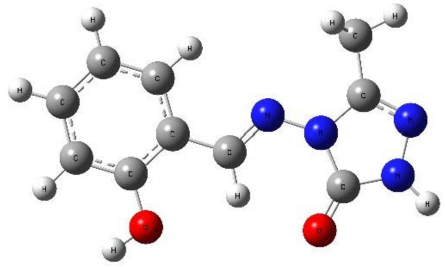
Compound **2**	Ethyl (4-{[(1E)-(2-hydroxyphenyl)methylidene]amino}-3-methyl-5-oxo-4,5-dihydro-1H-1,2,4-triazol-1-yl)acetate	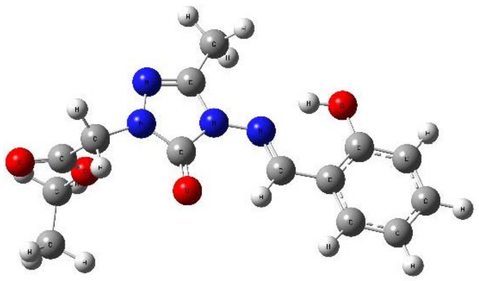
Compound **3**	2-(4-{[(1E)-(2-Hydroxyphenyl)methylidene]amino}-3-methyl-5-oxo-4,5-dihydro-1H-1,2,4-triazol-1-yl)acetohydrazide	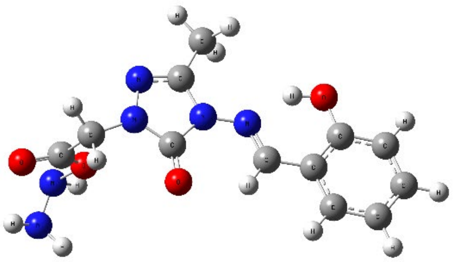

Compoun d **4**	2-[(4-{[(1E)-(2-Hydroxyphenyl)methylidene]amino}-3-methyl-5-oxo-4,5-dihydro-1H-1,2,4-triazol-1-yl)acetyl]-Nphenylhydrazinecarbothioamide	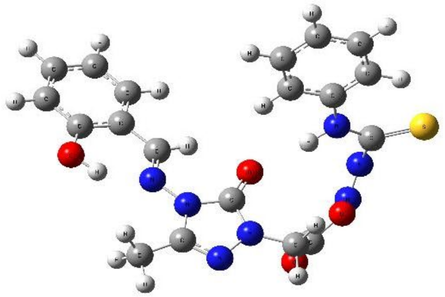
Compoun d **5**	2-[(4-{[(1E)-(2-Hydroxyphenyl)methylidene]amino}-3-methyl-5-oxo-4,5-dihydro-1H-1,2,4-triazol-1-yl)acetyl]-Nphenylhydrazinecarboxamide	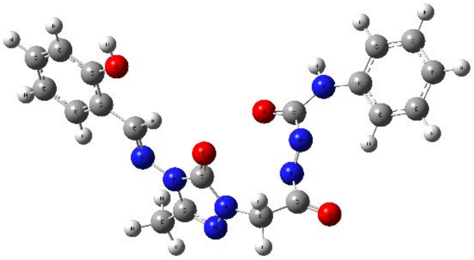
Compoun d **6**	N-Benzyl-2-[(4-{[(1Z)-(2-hydroxyphenyl)methylene]amino}-3-methyl-5-oxo-4,5-dihydro-1H-1,2,4-triazol-1-yl)carbonyl]hydrazinecarbothioamide	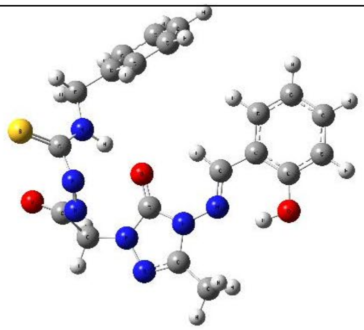
Compoun d **7**	4-{[(1E)-(2-Hydroxyphenyl)methylidene]amino}-5-methyl-2-[(4-phenyl-5-sulfanyl-4H-1,2,4-triazol-3-yl)methyl]-2,4-dihydro-3H-1,2,4-triazol-3-one	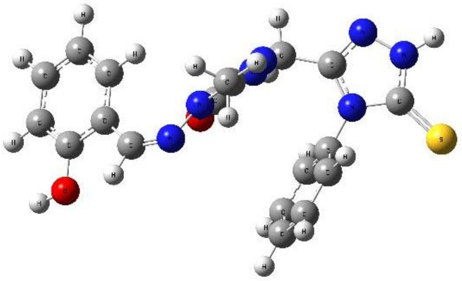
Compoun d **8**	4-{[(1E)-(2-Hydroxyphenyl)methylidene]amino}-5-methyl-2-[(5-oxo-4-phenyl-4,5-dihydro-1H-1,2,4-triazol-3-yl)methyl]-2,4-dihydro-3H-1,2,4-triazol-3-one	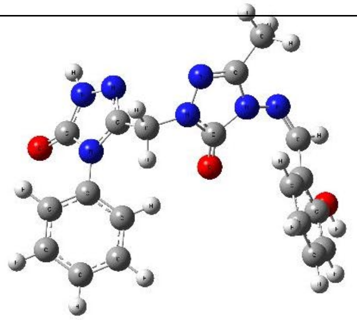

Compound **9**	2-[(4-Benzyl-5-sulfanyl-4H-1,2,4-triazol-3-yl)methyl]-4-{[(1E)-(2hydroxyphenyl) methylidene]amino}-5-methyl-2,4-dihydro-3H-1,2,4-triazol-3-one	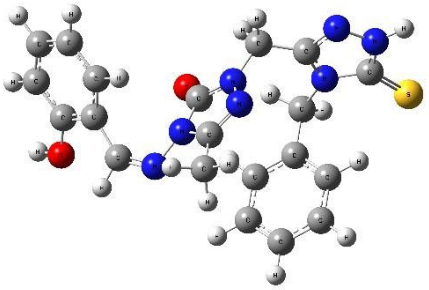
Compound **10**	1-Ethyl-6-fluoro-7-[4-({3-[(4-{[(1Z)-(2-hydroxyphenyl)methylene]amino}-3-methyl-5-oxo-4,5-dihydro-1H-1,2,4-triazol-1-yl)methyl]-4-phenyl-5-thioxo-4,5-dihydro-1H-1,2,4-triazol-1-yl}methyl)piperazin-1-yl]-4-oxo-1,4-dihydroquinoline-3-carboxylic acid	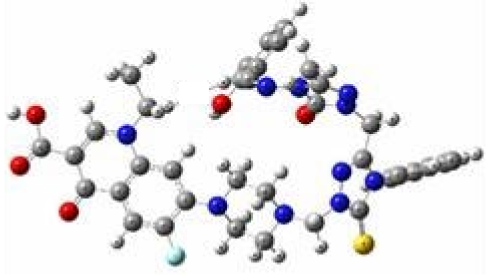
Compound **11**	1-Cyclopropyl-6-fluoro-7-[4-({3-[(4-{[(1Z)-(2-hydroxyphenyl)methylene]amino}-3-methyl _5-oxo-4,5-dihydro-1H-1,2,4-triazol-1-yl)methyl]-4-phenyl-5-thioxo-4,5-dihydro-1H-1,2,4-triazol-1-yl}methyl)piperazin-1-yl]-4-oxo-1,4-dihydro quinoline-3-carboxylic acid	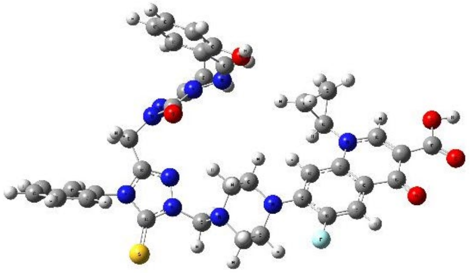
Compound **12**	1-Ethyl-6-fluoro-7-[4-({3-[(4-{[(1Z)-(2-hydroxyphenyl)methylene]amino}-3-methyl-5-oxo-4,5-dihydro-1H-1,2,4-triazol-1-yl)methyl]-5-oxo-4-phenyl-4,5-dihydro-1H-1,2,4-triazol-1-yl}methyl)piperazin-1-yl]-4-oxo-1,4-dihydroquinoline-3-carboxylic acid	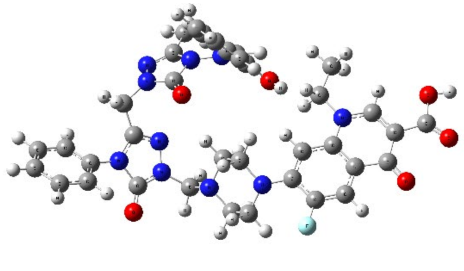
Compound **13**	1-Cyclopropyl-6-fluoro-7-[4-({3-[(4-{[(1Z)-(2-hydroxyphenyl)methylene]amino}-3-methyl _5-oxo-4,5-dihydro-1H-1,2,4-triazol-1-yl)methyl]-5-oxo-4-phenyl-4,5-dihydro-1H-1,2,4-triazol-1-yl}methyl)piperazin-1-yl]-4-oxo-1,4-dihydroquinoline-3-carboxylic acid	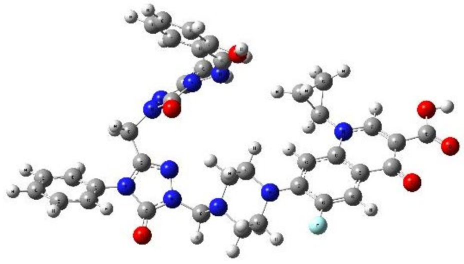

Compound **14**	7-[4-({4-Benzyl-3-[(4-{[(1Z)-(2-hydroxyphenyl)methylene]amino}-3-methyl-5-oxo-4,5-dihydro-1H-1,2,4-triazol-1-yl)methyl]-5-thioxo-4,5-dihydro-1H-1,2,4-triazol-1yl}methyl)piperazin-1-yl]-1-ethyl-6-fluoro-4-oxo-1,4-dihydroquinoline-3-carboxylic acid	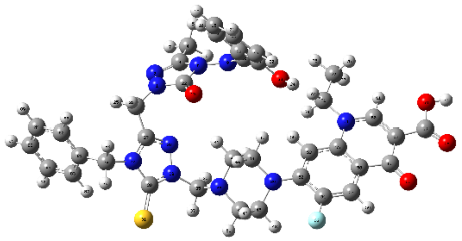
Compound **15**	7-[4-({4-Benzyl-3-[(4-{[(1Z)-(2-hydroxyphenyl)methylene]amino}-3-methyl-5-oxo-4,5-di hydro-1H-1,2,4-triazol-1-yl)methyl]-5-thioxo-4,5-dihydro-1H-1,2,4-triazol-1-yl} methyl)piperazin-1-yl]-1-cyclopropyl-6-fluoro-4-oxo-1,4-dihydroquinoline-3-carboxylic acid	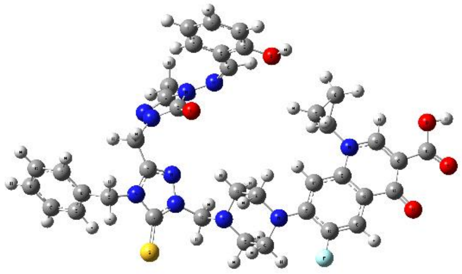
Compound **16**	2-[(5-{[2-(4-Chlorophenyl)-2-oxoethyl]thio}-4-phenyl-4H-1,2,4-triazol-3-yl)methyl]-84-{[(1E)-(2-hydroxyphenyl)methylene]amino}-5-methyl-2,4-dihydro-3H-1,2,4-triazol-3-one	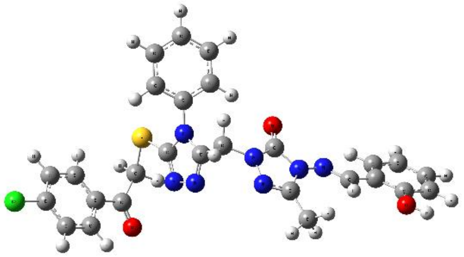
Compound **17**	2-[(5-{[2-(4-Chlorophenyl)-2-hydroxyethyl]thio}-4-phenyl-4H-1,2,4-triazol-3-yl)methyl]-4-{[(1Z)- (2-hydroxyphenyl)methylene]amino}-5-methyl-2,4-dihydro-3H-1,2,4-triazol-3-one	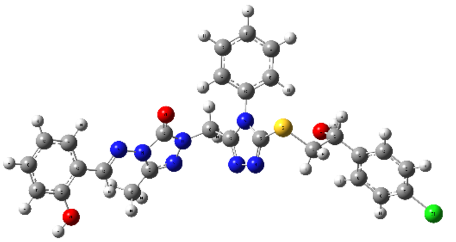
Compound **18**	2-{[5-({2-(4-Chlorophenyl)-2-[(2,4-dichlorobenzyl)oxy]ethyl}thio)-4-phenyl-4H-1,2,4-triazol-3-yl]methyl}-4-{[(1Z)-(2-hydroxyphenyl)methylene]amino}-5-methyl-2,4-dihydro-3H-1,2,4-triazol-3-one	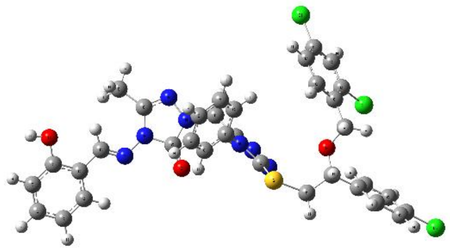

Compound 19	2-{[5-({2-(4-Chlorophenyl)-2-[(2,6-dichlorobenzyl)oxy]ethyl}thio)-4-phenyl-4H-1,2,4-triazol-3-yl]methyl}-4-{[(1Z)-(2-hydroxyphenyl)methylene]amino}-5-methyl-2,4-dihydro-3H-1,2,4-triazol-3-one	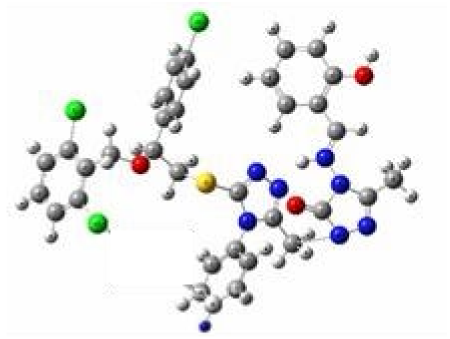
Compound 20	2-[(5-{[2-[(4-chlorobenzyl)oxy]-2-(4-chlorophenyl)ethyl]thio}-4-phenyl-4H-1,2,4-triazol-3-yl)methyl]-4-{[(1Z)-(2-hydroxyphenyl)methylene]amino}-5-methyl-2,4-dihydro-3H-1,2,4-triazol-3-one	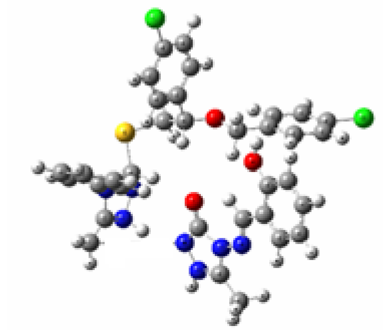

Table S33. Compound-protein interacting amino acid residues of compound **10** against 5CDQ.

Complex	interacting residues	Distance (Å)	type of interaction	2D diagram of interaction
Compound **10**-Topo II	ALA A:89	5.48	Pi-Alkyl	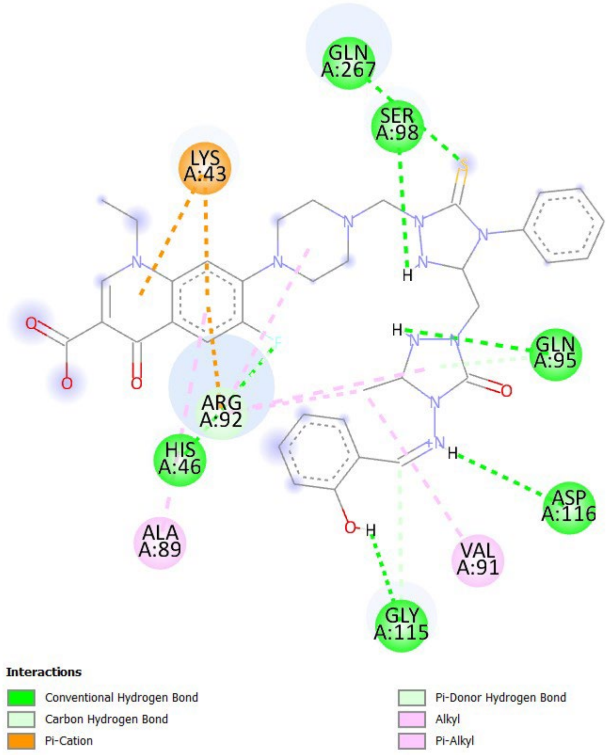
	ARG A:92	2.55. 2.95	Carbon Hydrogen Bond	
		3.66	Pi-Cation	
		4.33, 4.71	Alkyl	
		5.19	Pi-Alkyl	
	ASP A:116	3.05	Conventional Hydrogen Bond	
	GLN A:95	2.52	Conventional Hydrogen Bond	
		2.79	Pi-Donor Hydrogen Bond	
	GLN A:267	2.64	Conventional Hydrogen Bond	
	GLY A:115	2.16	Conventional Hydrogen Bond	
		3.41	Carbon Hydrogen Bond	
	HIS A:46	2.37	Conventional Hydrogen Bond; Halogen (Fluorine)	
	LYS A:43	3.11	Pi-Cation; Pi-Donor Hydrogen Bond	
		4.28	Pi-Cation	
	SER A:98	2.18	Conventional Hydrogen Bond	
	VAL A:91	4.73	Alkyl	

Table S34. Compound-protein interacting amino acid residues of compound **11** against 5CDQ.

Complex	interacting residues	Distance (Å)	type of interaction	2D diagram of interaction
Compound **11**-Topo II	ARG A:33	4.93	Pi-Alkyl	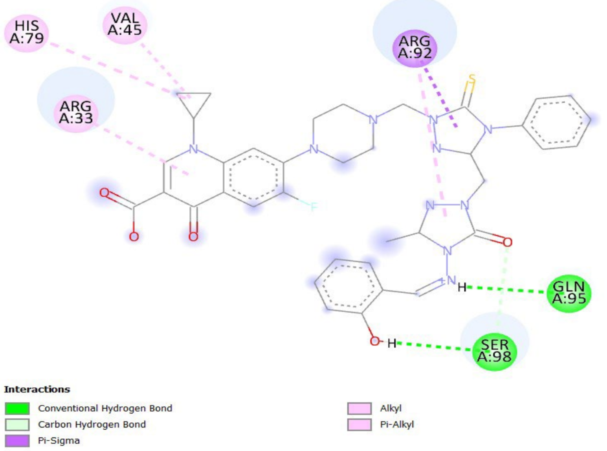
	ARG A:92	2.85	Pi-Sigma	
		4.81	Pi-Alkyl	
	GLN A:95	2.64	Conventional Hydrogen Bond	
	HIS A:79	5.43	Pi-Alkyl	
	VAL A:45	3.93	Alkyl	
	SER A:98	2.39	Conventional Hydrogen Bond	
		3.04	Carbon Hydrogen Bond	

Table S35. Compound-protein interacting amino acid residues of compound **12** against 5CDQ.

Complex	interacting residues	Distance (Å)	type of interaction	2D diagram of interaction
Compound **12**-Topo II	ALA A:118	4.35	Pi-Alkyl	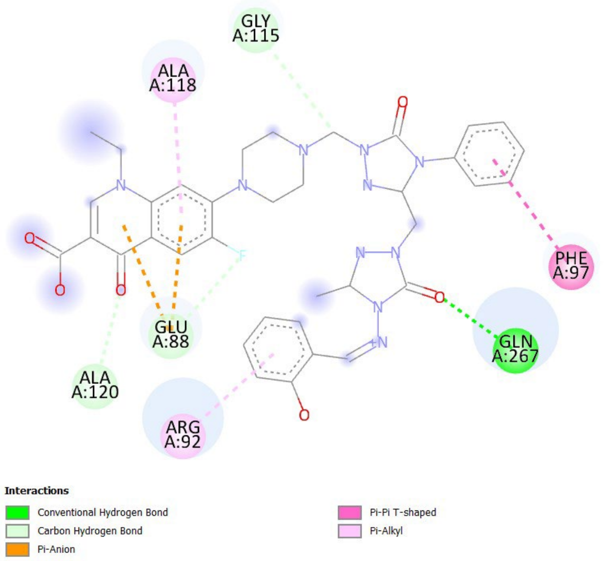
	ALA A:120	2.43	Carbon Hydrogen Bond	
	ARG A:92	4.32	Pi-Alkyl	
	GLN A:267	2.36	Conventional Hydrogen Bond	
	GLU A:88	2.31	Carbon Hydrogen Bond; Halogen (Fluorine)	
		4.35, 4.98	Pi-Anion	
	GLY A:115	3.44	Carbon Hydrogen Bond	
	PHE A:97	5.02	Pi-Pi T-shaped	

Table S36. Compound-protein interacting amino acid residues of compound **13** against 5CDQ.

Complex	interacting residues	Distance (Å)	type of interaction	2D diagram of interaction
Compound **13**-Topo II	ALA A:118	5.04	Pi-Alkyl	
	ALA A:120	2.68	Carbon Hydrogen Bond	
	ARG A:92	4.75	Pi-Alkyl	
	ASP A:116	3.07	Carbon Hydrogen Bond	
	GLN A:95	2.53	Conventional Hydrogen Bond	
	GLN A:267	2.34	Conventional Hydrogen Bond	
	GLU A:88	3.65, 3.69	Pi-Anion	

	GLY A:115	2.89	Conventional Hydrogen Bond	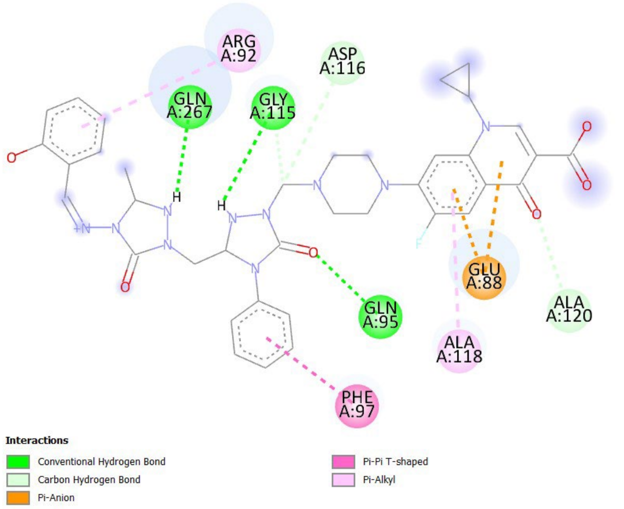
		3.29	Carbon Hydrogen Bond	
	PHE A:97	4.93	Pi-Pi T-shaped	

Table S37. Compound-protein interacting amino acid residues of compound **14** against 5CDQ

Complex	interacting residues	Distance (Å)	type of interaction	2D diagram of interaction
Compound **14**-Topo II	ALA A:180	5.35	Pi-Alkyl	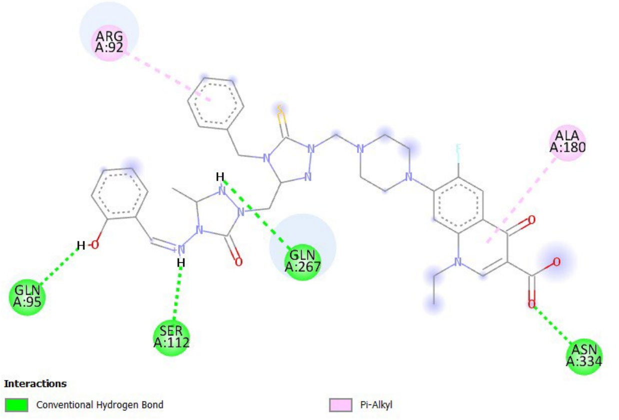
	ARG A:92	4.978	Pi-Alkyl	
	ASN A:334	2.34	Conventional Hydrogen Bond	
	GLN A:95	2.46	Conventional Hydrogen Bond	
	GLN A:267	2.48	Conventional Hydrogen Bond	
	SER A:112	2.24	Conventional Hydrogen Bond	

Table S38. Compound-protein interacting amino acid residues of compound **15** against 5CDQ.

Complex	interacting residues	Distance (Å)	type of interaction	2D diagram of interaction
Compound **15**-Topo II	ALA A:118	4.88	Pi-Alkyl	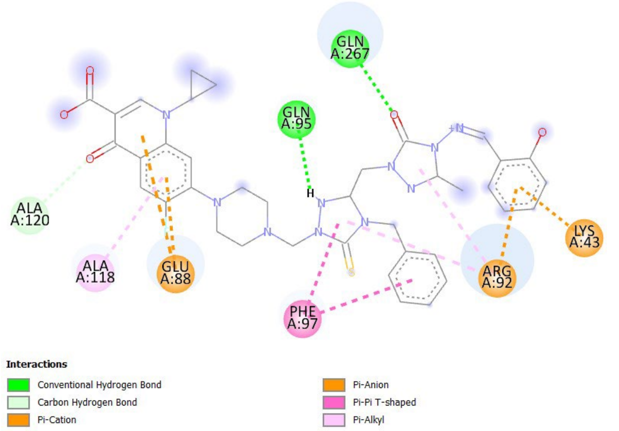
	ALA A:120	2.62	Carbon Hydrogen Bond	
	ARG A:92	4.64	Pi-Cation	
		4.99, 5.37	Pi-Alkyl	
	GLN A:95	2.48	Conventional Hydrogen Bond	
	GLN A:267	2.31	Conventional Hydrogen Bond	
	GLU A:88	3.79, 3.88	Pi-Anion	
	LYS A:43	2.78	Pi-Cation; Pi-Donor Hydrogen Bond	
	PHE A:97	5.08, 5.78	Pi-Pi T-shaped	

Table S39. Compound-protein interacting amino acid residues of compound **16** against 5CDQ.

Complex	interacting residues	Distance (Å)	type of interaction	2D diagram of interaction
Compound **16**-Topo II	ALA A:118	2.82<br>4.21	Pi-Donor Hydrogen Bond<br>Pi-Alkyl	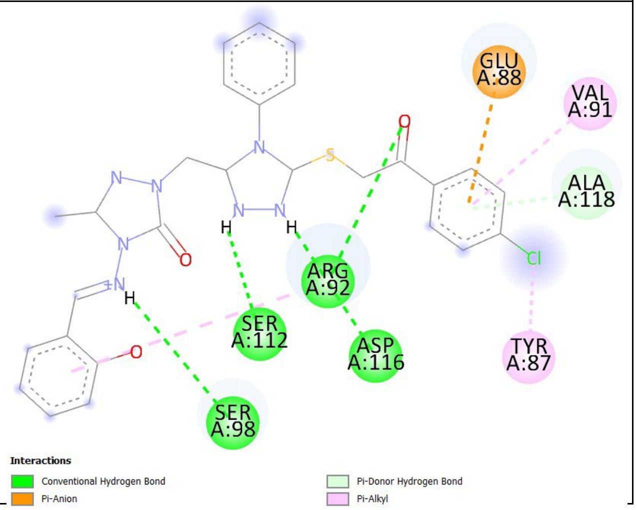
	ARG A:92	2.51,<br>2.75<br>4.62	Conventional Hydrogen Bond<br>Pi-Alkyl	
	ASP A:116	2.64	Conventional Hydrogen Bond	
	GLU A:88	4.37	Pi-Anion	
	SER A:112	2.05	Conventional Hydrogen Bond	
	SER A:98	2.67	Conventional Hydrogen Bond	
	TYR A:87	4.86	Pi-Alkyl	
	VAL A:91	4.91	Pi-Alkyl	

Table S40. Compound-protein interacting amino acid residues of compound **17** against 5CDQ.

Complex	interacting residues	Distance (Å)	type of interaction	2D diagram of interaction
Compound **17**-Topo II	ALA A:118	3.00<br>4.38	Pi-Donor Hydrogen Bond<br>Pi-Alkyl	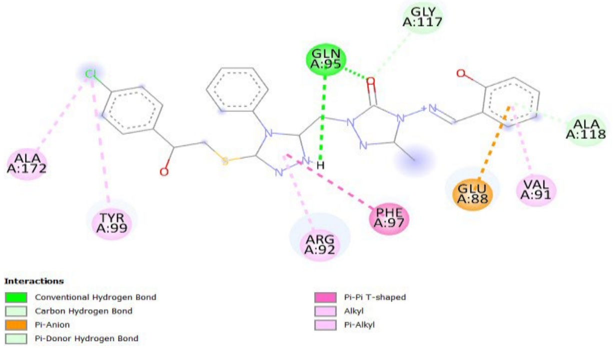
	ALA A:172	4.34	Alkyl	
	ARG A:92	4.71	Pi-Alkyl	
	GLN A:95	2.32,<br>2.53	Conventional Hydrogen Bond	
	GLU A:88	4.19	Pi-Anion	
	GLY A:117	2.46	Carbon Hydrogen Bond	
	PHE A:97	5.93	Pi-Pi T-shaped	
	TYR A:99	5.22	Pi-Alkyl	
	VAL A:91	4.97	Pi-Alkyl	

Table S41. Compound-protein interacting amino acid residues of fluconazole against 5CDQ.

Complex	interacting residues	Distance (Å)	type of interaction	2D diagram of interaction
Fluconazole-Topo II	GLN A:95	2.32142	Conventional Hydrogen Bond	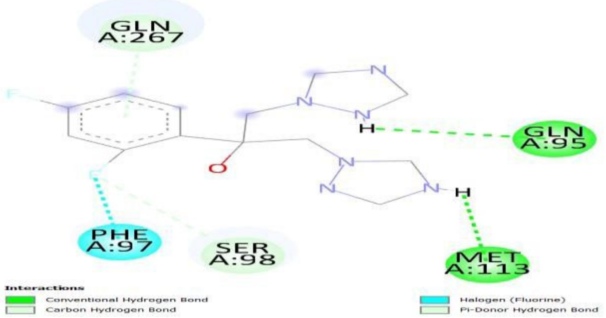
	GLN A:267	3.07789	Pi-Donor Hydrogen Bond	
	MET A:113	2.02286	Conventional Hydrogen Bond	
	PHE A:97	3.52203	Halogen (Fluorine)	
	SER A:98	2.40779	Carbon Hydrogen Bond;Halogen (Fluorine)	

Table S42. Compound-protein interacting amino acid residues of compound **10** against 2Z3Y.

Complex	interacting residues	Distance (Å)	type of interaction	2D diagram of interaction
Compound **10**-LSD1	ALA A:539	3.65<br>4.63<br>4.72	Carbon Hydrogen Bond<br>Alkyl<br>Pi-Alkyl	

	ALA A:809	2.88	Halogen (Fluorine)	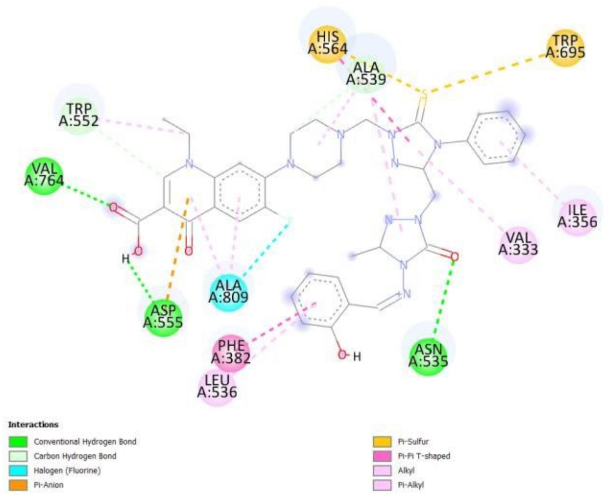
		4.73, 5.17	Pi-Alkyl	
	ASN A:535	2.29	Conventional Hydrogen Bond	
	ASP A:555	2.46	Conventional Hydrogen Bond	
		4.11	Pi-Anion	
	HIS A:564	4.33	Pi-Pi T-shaped	
		5.56	Pi-Sulfur	
	ILE A:356	4.59	Pi-Alkyl	
	LEU A:536	5.34	Pi-Alkyl	
	PHE A:382	4.82	Pi-Pi T-shaped	
	TRP A:552	3.32	Carbon Hydrogen Bond	
		5.25	Pi-Alkyl	
	TRP A:695	4.57	Pi-Sulfur	
	VAL A:333	5.37	Pi-Alkyl	
	VAL A:764	2.43	Conventional Hydrogen Bond	

Table S43. Compound-protein interacting amino acid residues of compound **11** against 2Z3Y.

Complex	interacting residues	Distance (Å)	type of interaction	2D diagram of interaction
Compound **11**-LSD1	ALA A:331	3.81	Pi-Alkyl	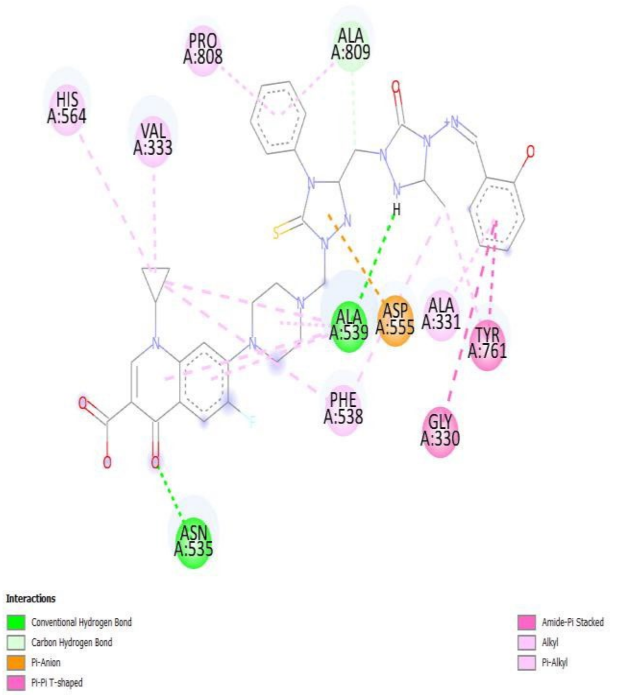
		5.61	Amide-Pi Stacked	
	ALA A:539	1.87	Conventional Hydrogen Bond	
		4.92, 5.16	Alkyl	
		4.47, 5.13	Pi-Alkyl	
	ALA A:809	3.44	Carbon Hydrogen Bond	
		3.94	Pi-Alkyl	
	ASN A:535	2.00	Conventional Hydrogen Bond	
	ASP A:555	4.59	Pi-Anion	
	GLY A:330	5.61	Amide-Pi Stacked	
	HIS A:564	5.36	Pi-Alkyl	
	PHE A:538	4.09, 5.12	Pi-Alkyl	
	PRO A:808	5.17	Pi-Alkyl	
	TYR A:761	5.24	Pi-Pi T-shaped	
		5.33	Pi-Alkyl	
	VAL A:333	4.75	Alkyl	

Table S44. Compound-protein interacting amino acid residues of compound **12** against 2Z3Y.

Complex	interacting residues	Distance (Å)	type of interaction	2D diagram of interaction
Compound **12**-LSD1	ALA A:331	4.67	Pi-Alkyl	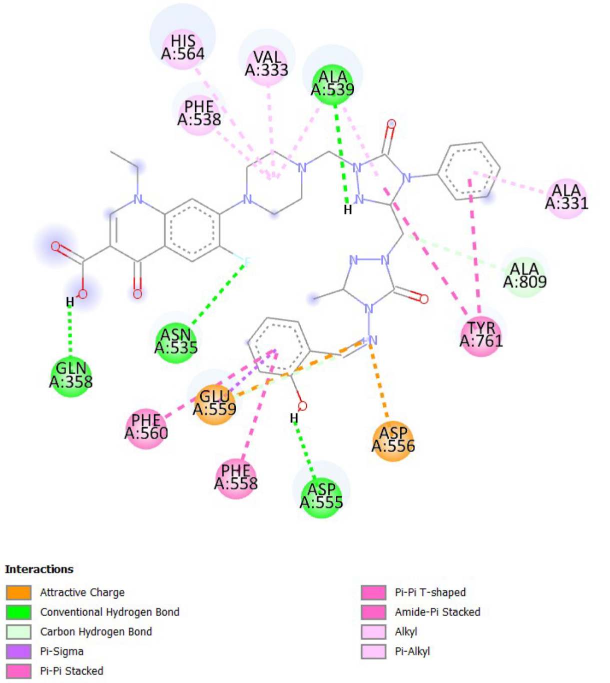
	ALA A:539	2.91	Conventional Hydrogen Bond	
		4.99	Pi-Alkyl	
		5.02	Alkyl	
	ALA A:809	3.46	Carbon Hydrogen Bond	
	ASN A:535	2.83	Conventional Hydrogen Bond; Halogen (Fluorine)	
	ASP A:555	2.38	Conventional Hydrogen Bond	
	ASP A:556	5.44	Attractive Charge	
	GLN A:358	1.89	Conventional Hydrogen Bond	
	GLU A:559	2.63	Pi-Sigma	
		3.69	Carbon Hydrogen Bond	
		4.55	Attractive Charge	
		4.51	Amide-Pi Stacked	
	HIS A:564	5.10	Pi-Alkyl	
	PHE A:538	5.48	Pi-Alkyl	
	PHE A:558	4.51	Amide-Pi Stacked	
	PHE A:560	5.73	Pi-Pi T-shaped	
	TYR A:761	5.30	Pi-Pi T-shaped	
		5.44	Pi-Pi Stacked	
	VAL A:333	5.49	Alkyl	

Table S45. Compound-protein interacting amino residues of compound **13** against 2Z3Y.

Complex	interacting residues	Distance (Å)	type of interaction	2D diagram of interaction
Compound **13**-LSD1	ALA A:331	3.56	Pi-Alkyl	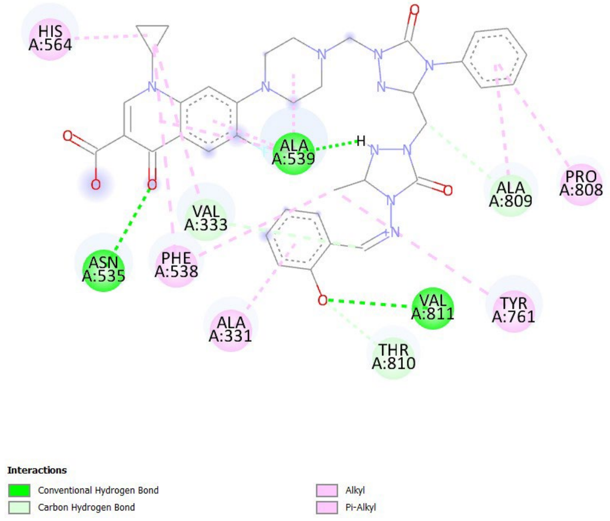
	ALA A:539	2.07	Conventional Hydrogen Bond	
		4.52, 4.99, 5.20	Alkyl, Pi-Alkyl	
	ALA A:809	3.22	Carbon Hydrogen Bond	
		3.98	Pi-Alkyl	
	ASN A:535	2.15	Conventional Hydrogen Bond	
	HIS A:564	5.34	Pi-Alkyl	
	PHE A:538	5.13, 4.17	Pi-Alkyl	
	PRO A:808	5.15	Pi-Alkyl	
	THR A:810	2.51	Carbon Hydrogen Bond	
	TYR A:761	5.48	Pi-Alkyl	
	VAL A:333	3.70	Carbon Hydrogen Bond	
		4.54	Alkyl	
	VAL A:811	2.47	Conventional Hydrogen Bond	

Table S46. Compound-protein interacting amino acid residues of compound **14** against 2Z3Y.

Complex	interacting residues	Distance (Å)	type of interaction	2D diagram of interaction
Compound **14**-LSD1	ALA A:331	3.99<br>4.21, 4.72<br>3.97, 5.01	Alkyl<br>Pi-Alkyl<br>Amide-Pi Stacked	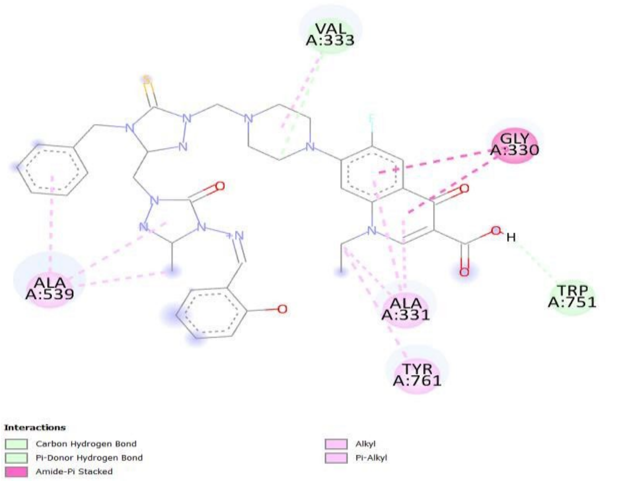
	ALA A:539	3.73<br>4.35, 4.61	Alkyl<br>Pi-Alkyl	
	GLY A:330	3.97, 5.01	Amide-Pi Stacked	
	TRP A:751	2.26, 3.39	Pi-Donor Hydrogen Bond	
	TYR A:761	4.86	Pi-Alkyl	
	VAL A:333	3.53<br>5.08	Carbon Hydrogen Bond<br>Alkyl	

Table S47. Compound-protein interacting amino acid residues of compound **15** against 2Z3Y.

Complex	interacting residues	Distance (Å)	type of interaction	2D diagram of interaction
Compound **15**-LSD1	ARG A:182	2.75<br>4.83, 4.74	Conventional Hydrogen Bond<br>Pi-Alkyl	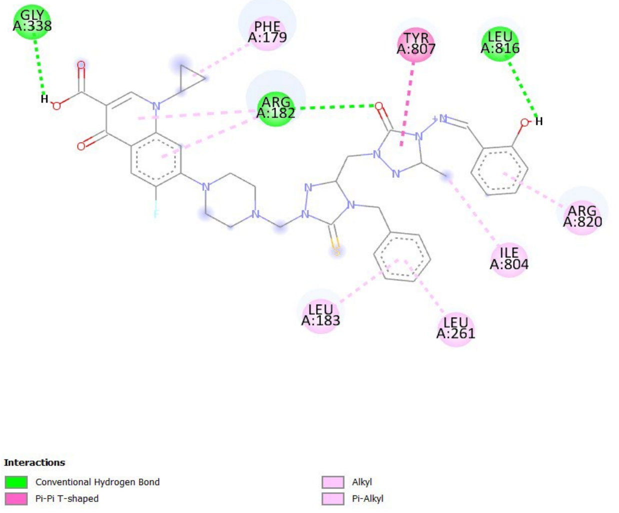
	ARG A:820	3.61	Pi-Alkyl	
	GLY A:338	2.19	Conventional Hydrogen Bond	
	ILE A:804	4.77	Alkyl	
	LEU A:183	5.30	Pi-Alkyl	
	LEU A:261	5.49	Pi-Alkyl	
	LEU A:816	2.75	Conventional Hydrogen Bond	
	PHE A:179	5.23	Pi-Alkyl	
	TYR A:807	5.08	Pi-Pi T-shaped	

Table S48. Compound-protein interacting amino acid residues of compound **16** against 2Z3Y.

Complex	interacting residues	Distance (Å)	type of interaction	2D diagram of interaction
Compound **16**-LSD1	ARG A:182	1.89, 2.58<br>4.19	Conventional Hydrogen Bond<br>Pi-Alkyl	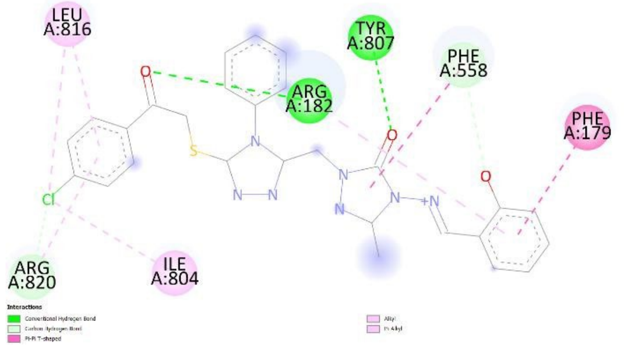
	ARG A:820	2.51<br>3.73<br>5.19	Carbon Hydrogen Bond<br>Alkyl<br>Pi-Alkyl	
	ILE A:804	4.24	Alkyl	
	LEU A:816	4.36<br>4.99	Pi-Alkyl<br>Alkyl	
	PHE A:179	5.09	Pi-Pi T-shaped	

	PHE A:558	2.97	Carbon Hydrogen Bond	
		4.95	Pi-Pi T-shaped	
	TYR A:807	2.62	Conventional Hydrogen Bond	

Table S49. Compound-protein interacting amino acid residues of compound **17** against 2Z3Y.

Complex	interacting residues	Distance (Å)	type of interaction	2D diagram of interaction
Compound **17**-LSD1	ARG A:182	5.49	Pi-Alkyl	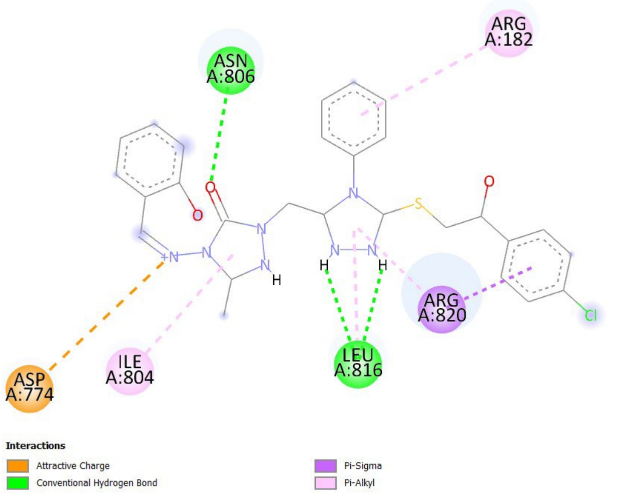
	ARG A:820	2.27	Pi-Sigma	
		4.61	Pi-Alkyl	
	ASN A:806	2.45	Conventional Hydrogen Bond	
	ASP A:774	5.25	Attractive Charge	
	ILE A:804	5.44	Pi-Alkyl	
	LEU A:816	2.56, 2.59	Conventional Hydrogen Bond	
		4.33	Pi-Alkyl	

Table S50. Compound-protein interacting amino acid residues of compound gemcitabine against 2Z3Y.

Complex	interacting residues	Distance (Å)	type of interaction	2D diagram of interaction
Gemcitabine-LSD1	ALA A:539	2.15	Conventional Hydrogen Bond	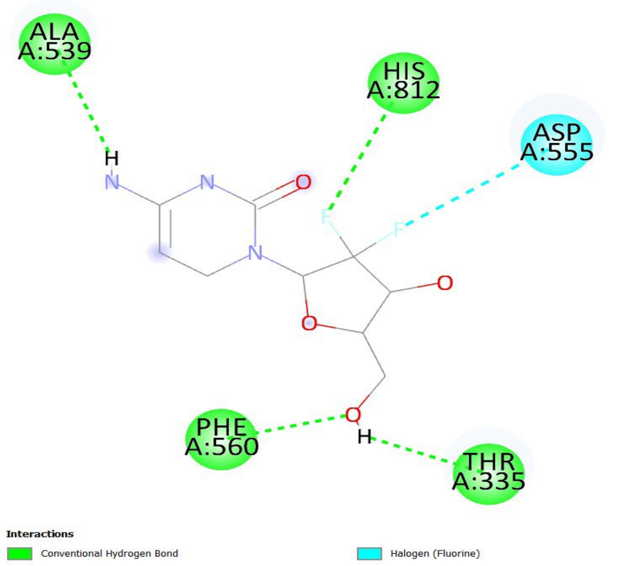
	ASP A:555	3.08	Halogen (Fluorine)	
	HIS A:812	2.79	Conventional Hydrogen Bond; Halogen (Fluorine)	
	PHE A:560	1.82	Conventional Hydrogen Bond	
	THR A:335	1.84	Conventional Hydrogen Bond	

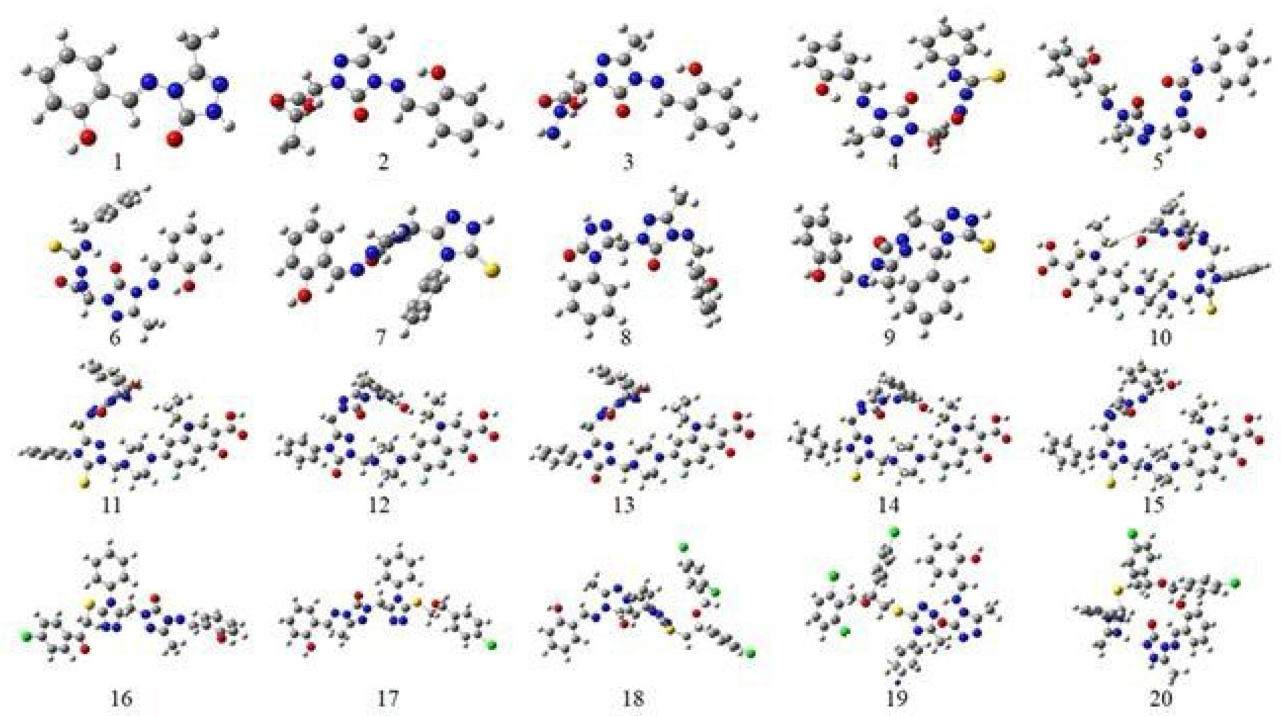

Figure S4. Optimized structures for potential antimicrobial and anticancer compounds (**1**-**20**).

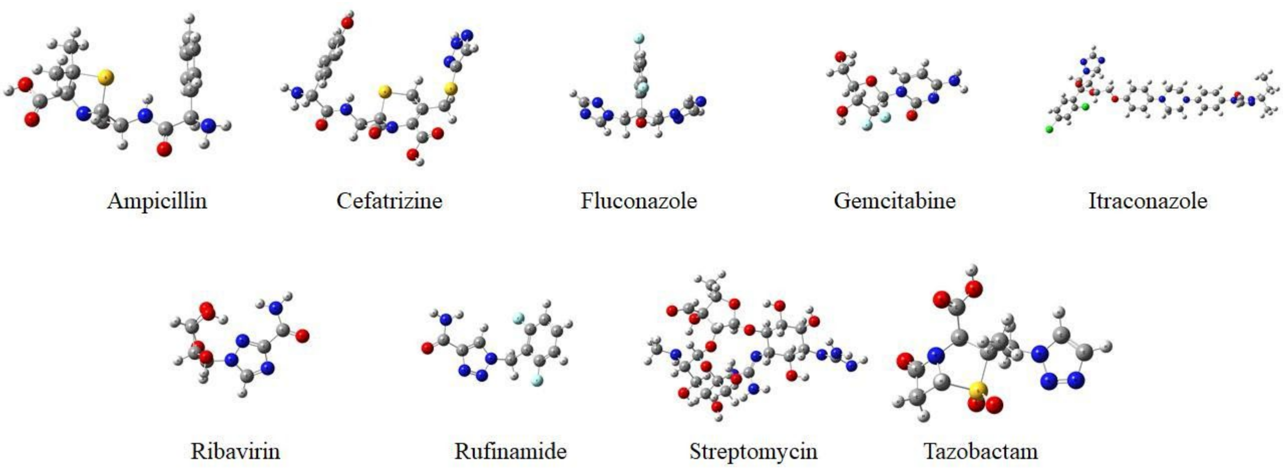

Figure S5. Optimized structures for reference drugs.

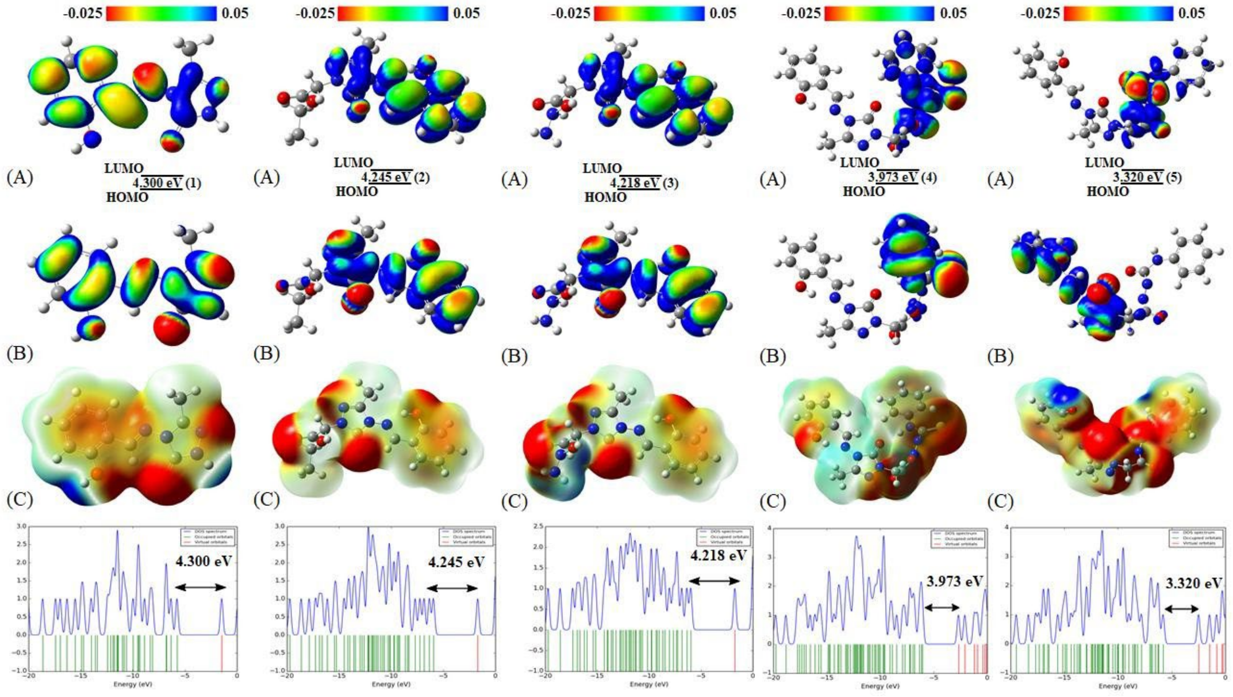

Figure S6. Frontier Molecular orbitals (FMO) of HOMO and LUMO gap for compounds (**1−5**).

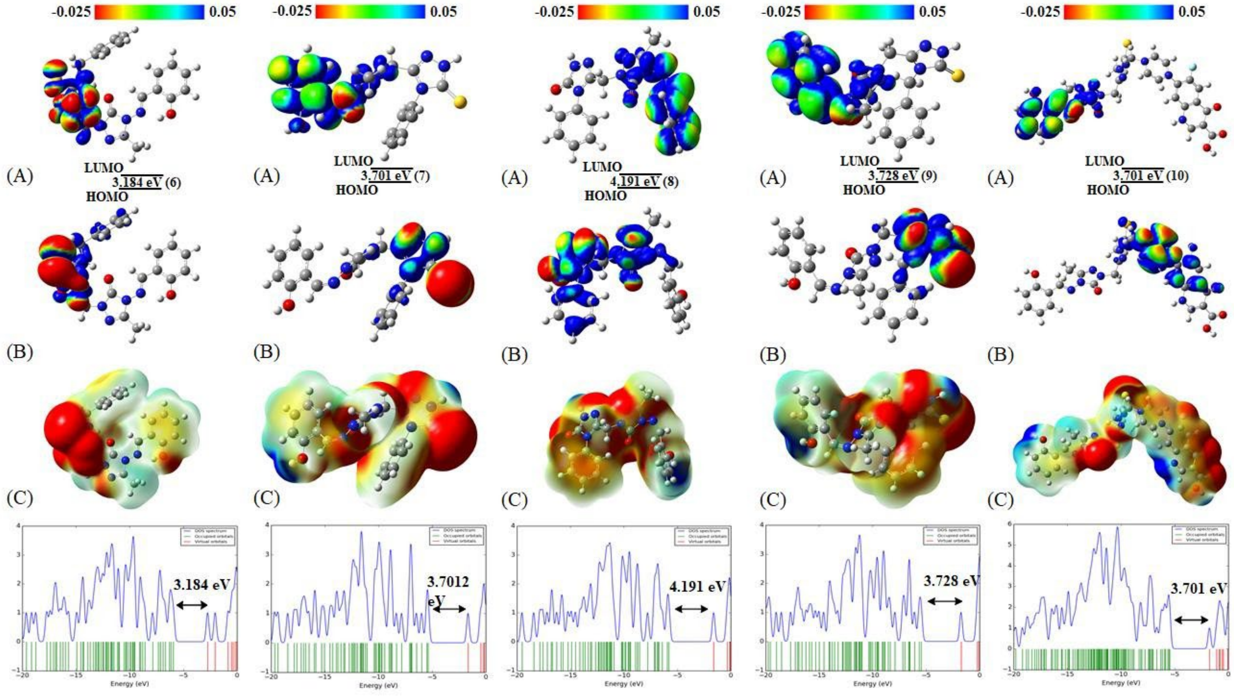

Figure S7. Frontier Molecular orbitals (FMO) of HOMO and LUMO gap for compounds (**610**).

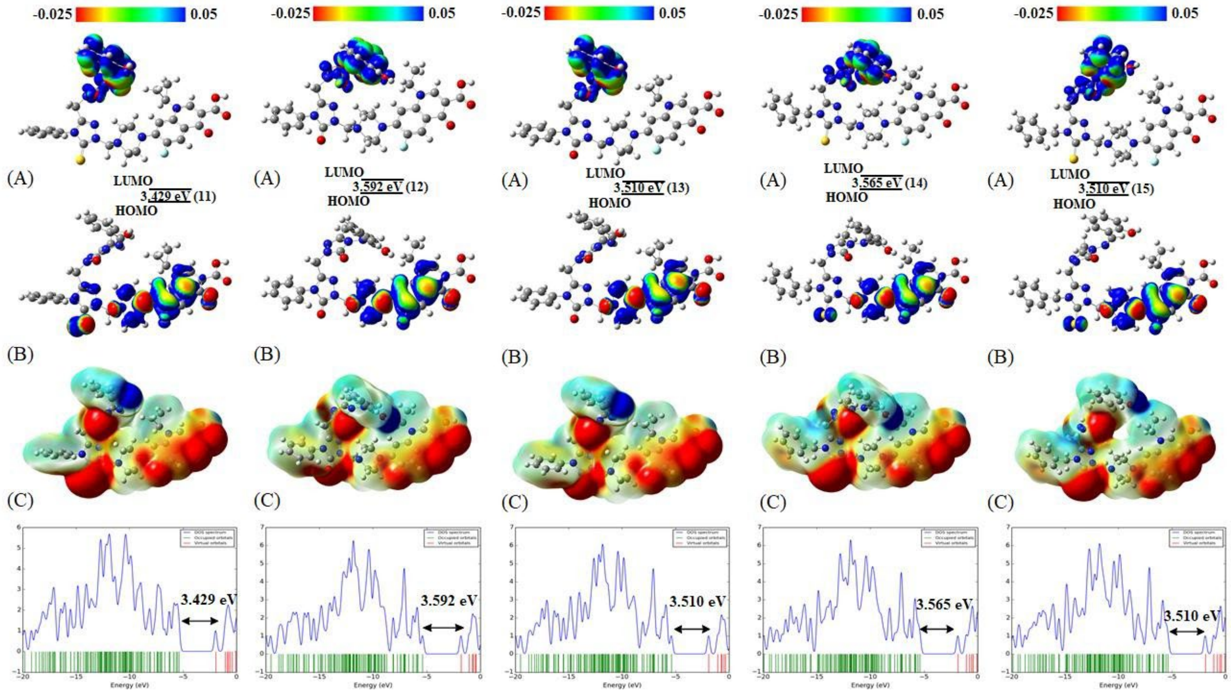

Figure S8. Frontier Molecular orbitals (FMO) of HOMO and LUMO gap for compounds (**11**−**15**).

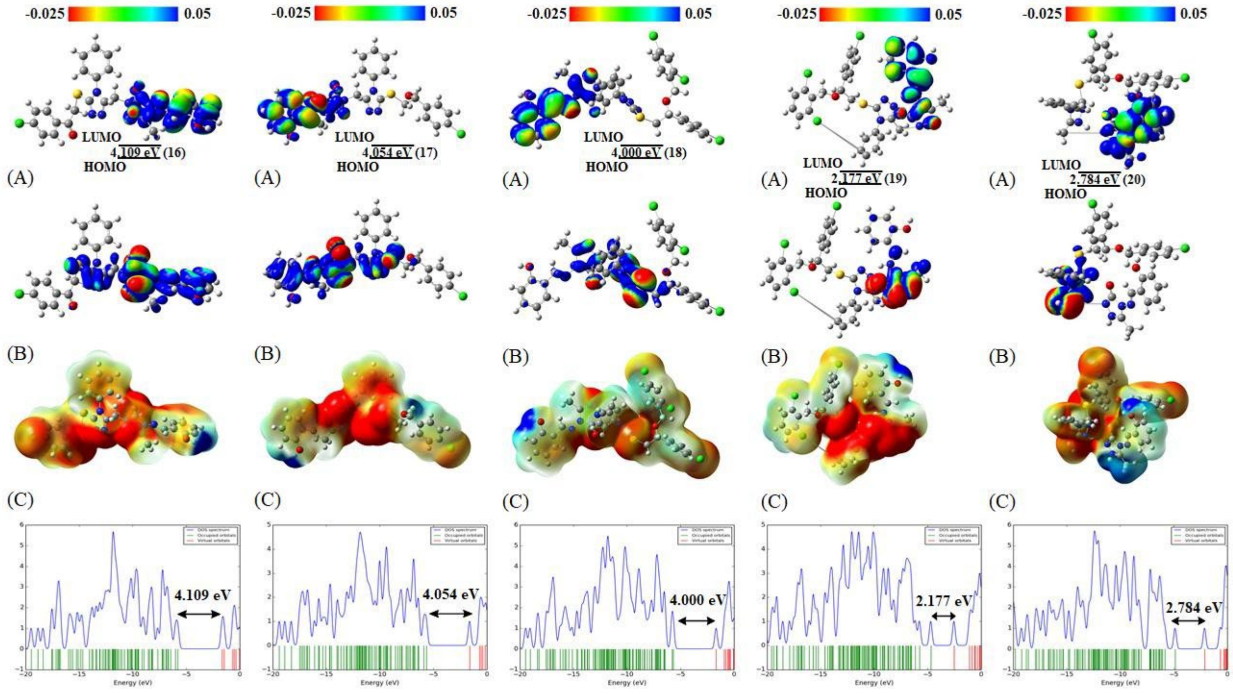

Figure S9. Frontier Molecular orbitals (FMO) of HOMO and LUMO gap for compounds (**16**−**20**).

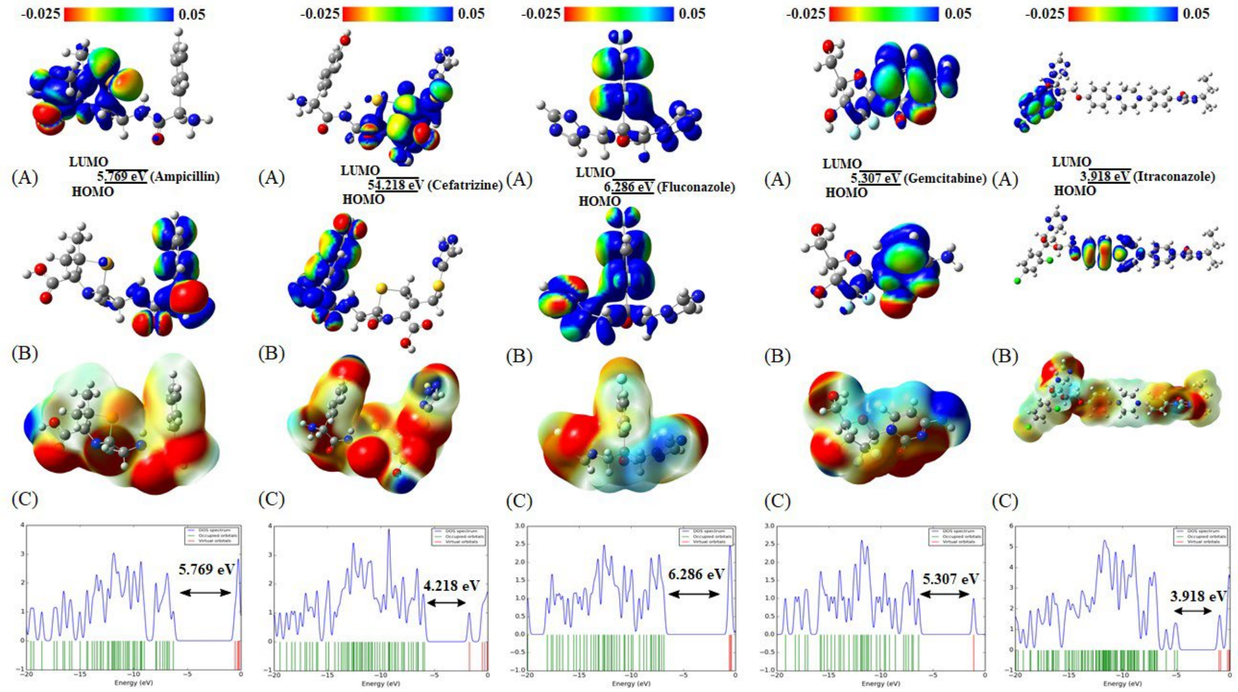

Figure S10. Frontier Molecular orbitals (FMO) of HOMO and LUMO gap for reference drugs.

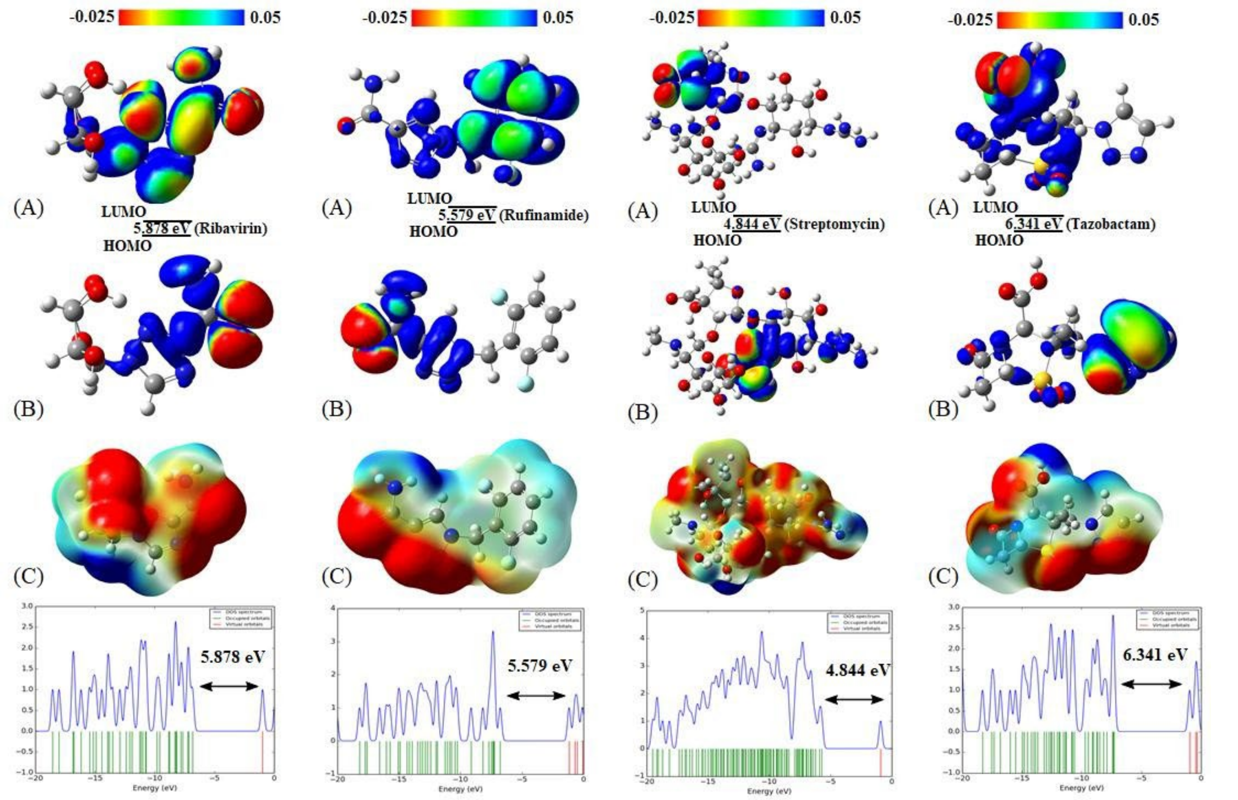

Figure S11. Frontier Molecular orbitals (FMO) of HOMO and LUMO gap for reference drugs.

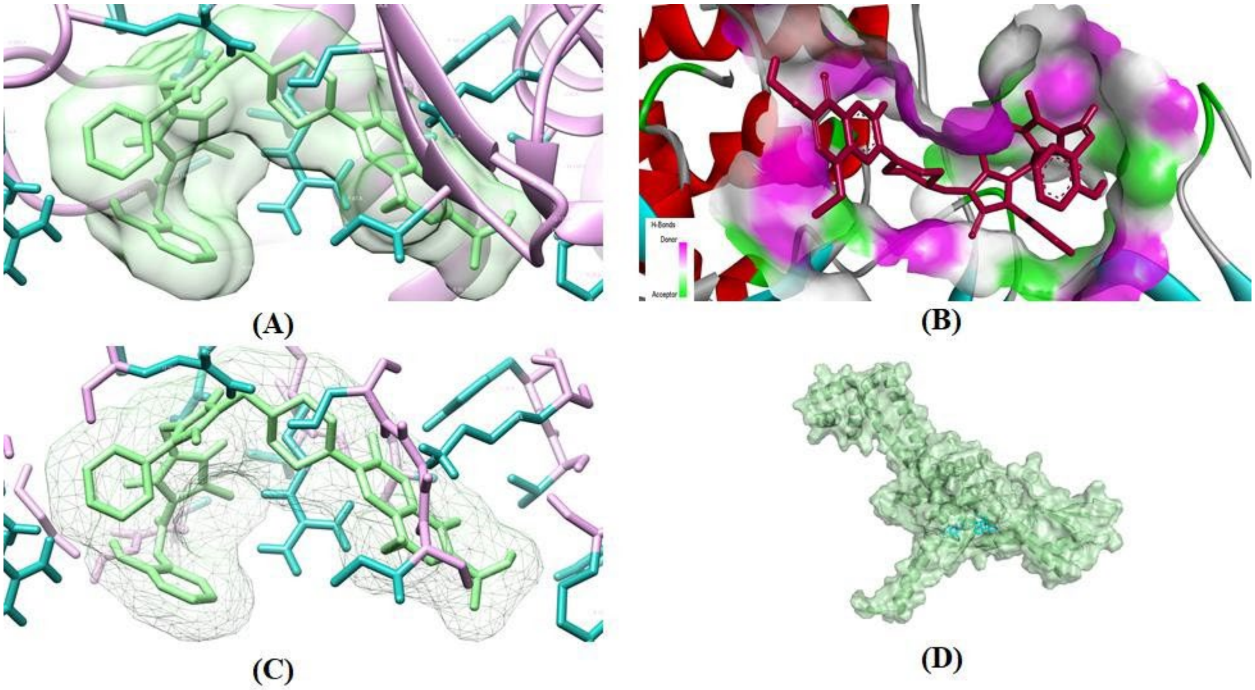

Figure S12. Molecular docking poses: (**A**) Ligand in protein pocket; (**B**) Active site; (**C**) Hydrogen bonding in solid for compound **10**; (**D**) Binding cavity of compound **10** in Topoisomerase II (PDB: 5CDQ).

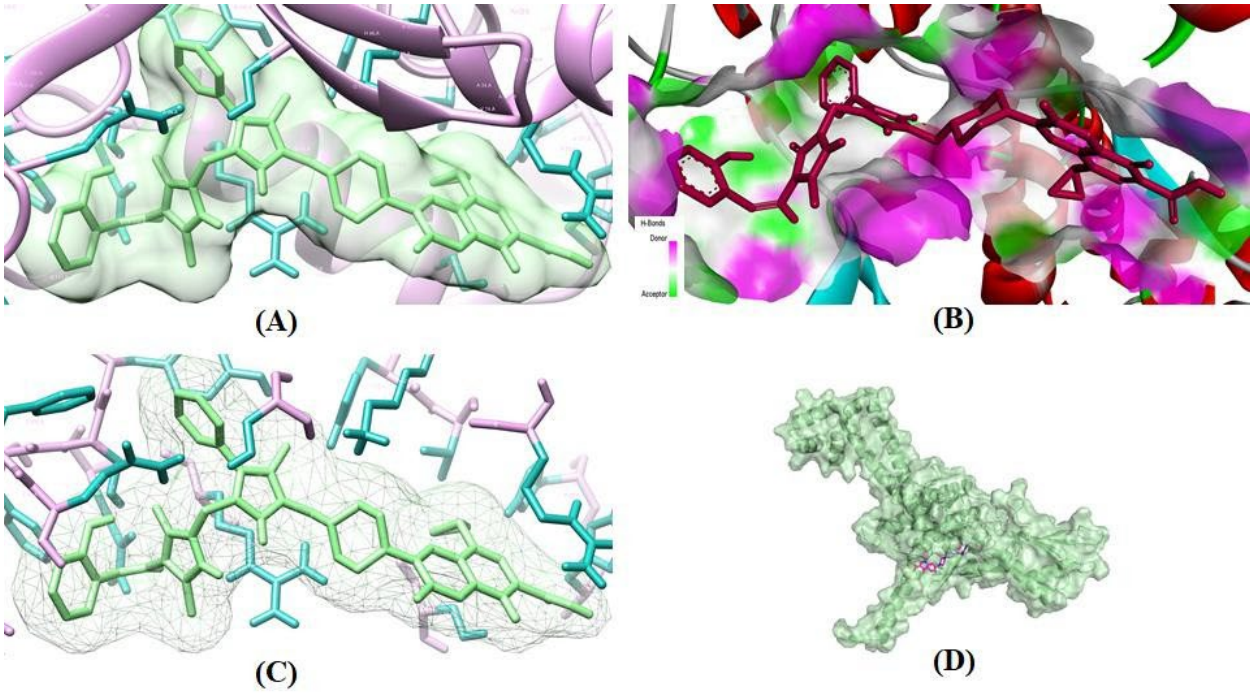

Figure S13. Molecular docking poses: (**A**) Ligand in protein pocket; (**B**) Active site; (**C**) Hydrogen bonding in solid for compound **11**; (**D**) Binding cavity of compound **11** in Topoisomerase II (PDB: 5CDQ).

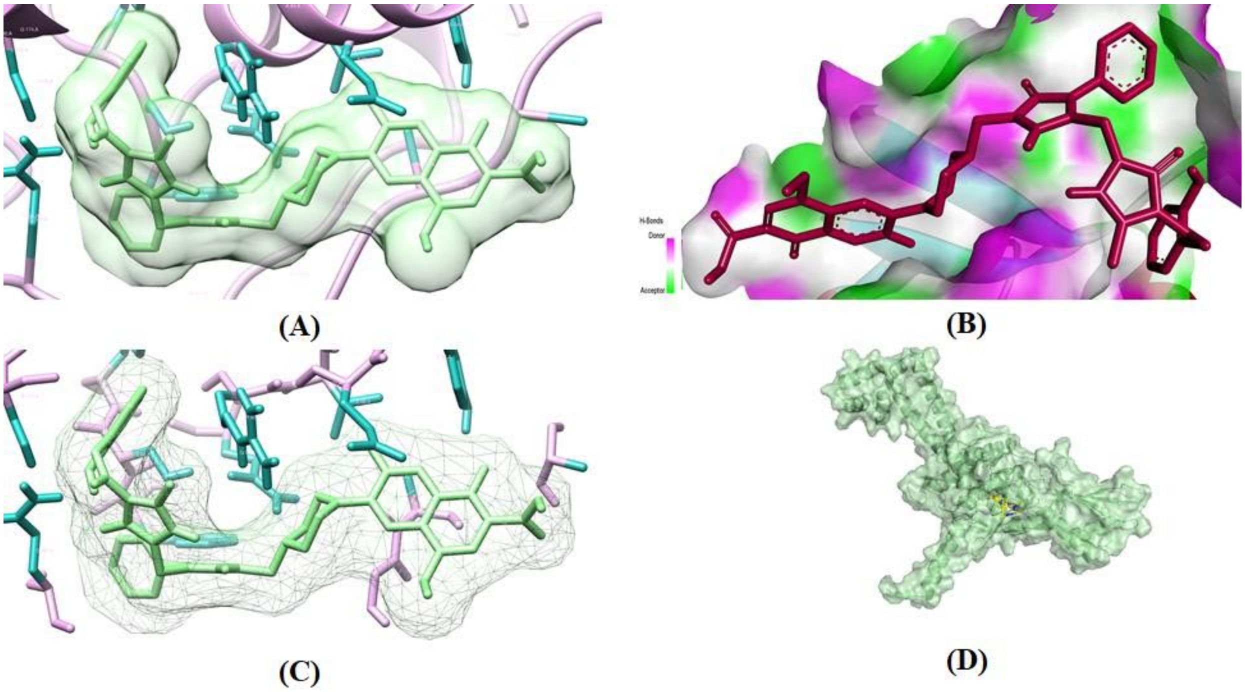

Figure S14. Molecular docking poses: (**A**) Ligand in protein pocket; (**B**) Active site; (**C**) Hydrogen bonding in solid for compound **12**; (**D**) Binding cavity of compound **12** in Topoisomerase II (PDB: 5CDQ).

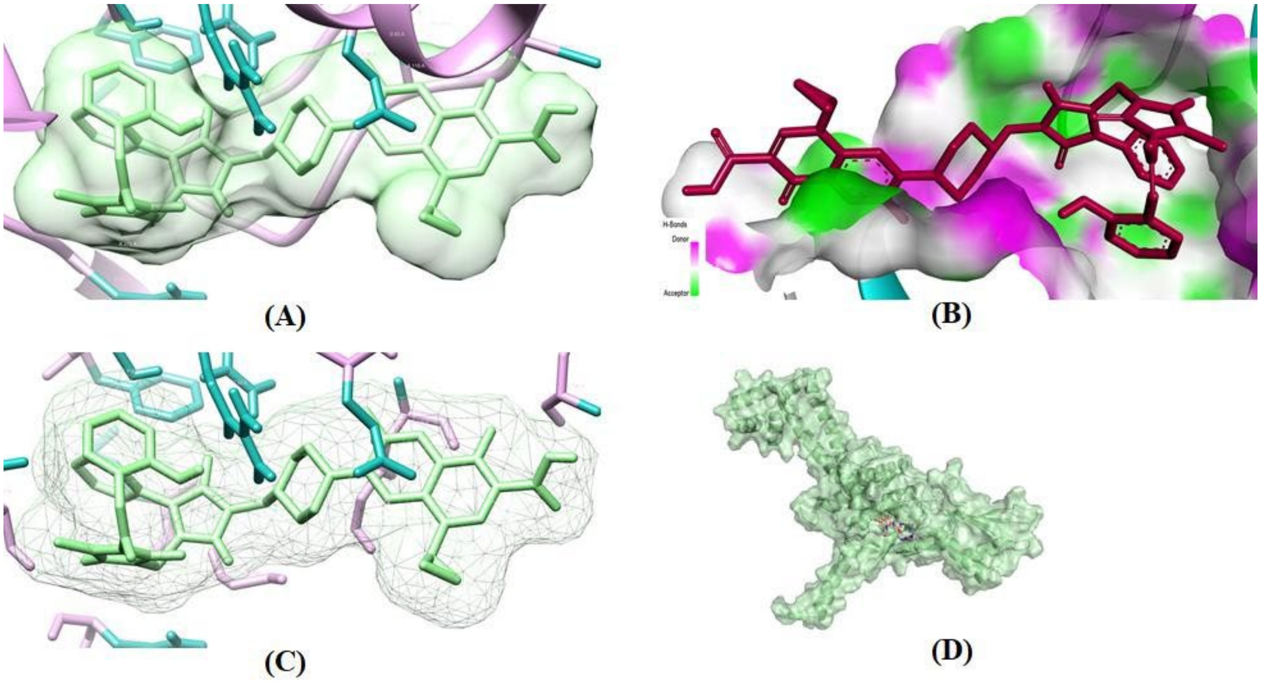

Figure S15. Molecular docking poses: (**A**) Ligand in protein pocket; (**B**) Active site; (**C**) Hydrogen bonding in solid for compound **13**; (**D**) Binding cavity of compound **13** in Topoisomerase II (PDB: 5CDQ).

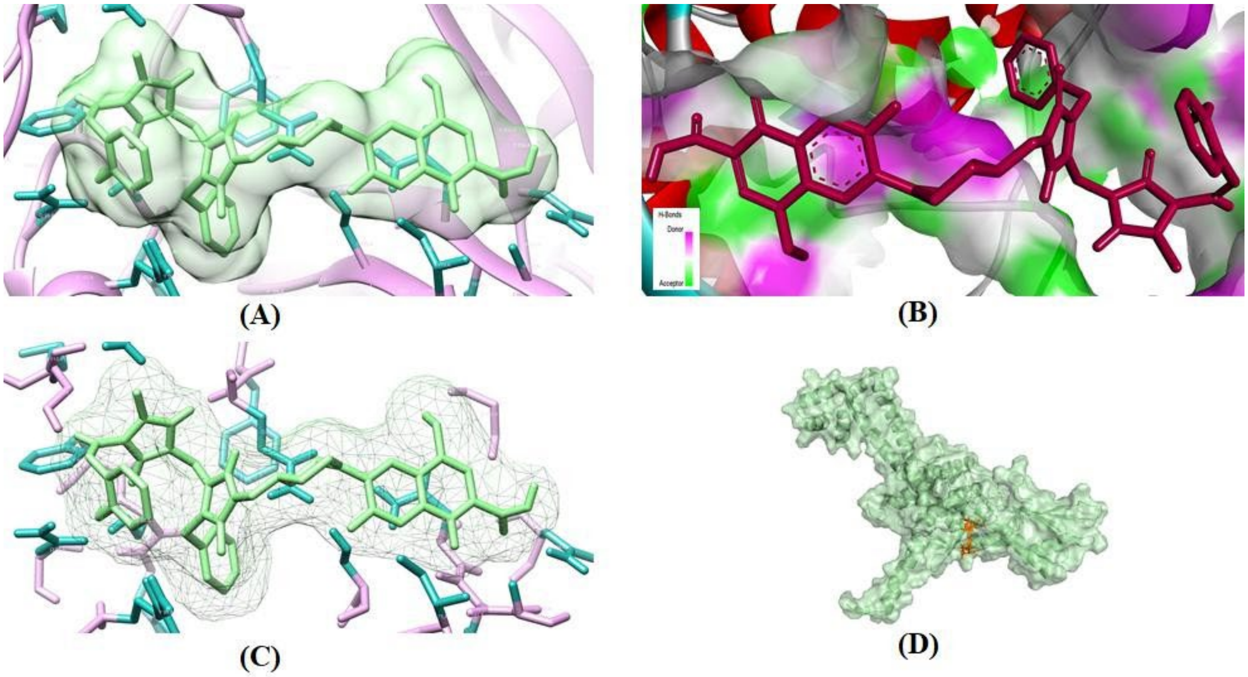

Figure S16. Molecular docking poses: (**A**) Ligand in protein pocket; (**B**) Active site; (**C**) Hydrogen bonding in solid for compound **14**; (**D**) Binding cavity of compound **14** in Topoisomerase II (PDB: 5CDQ).

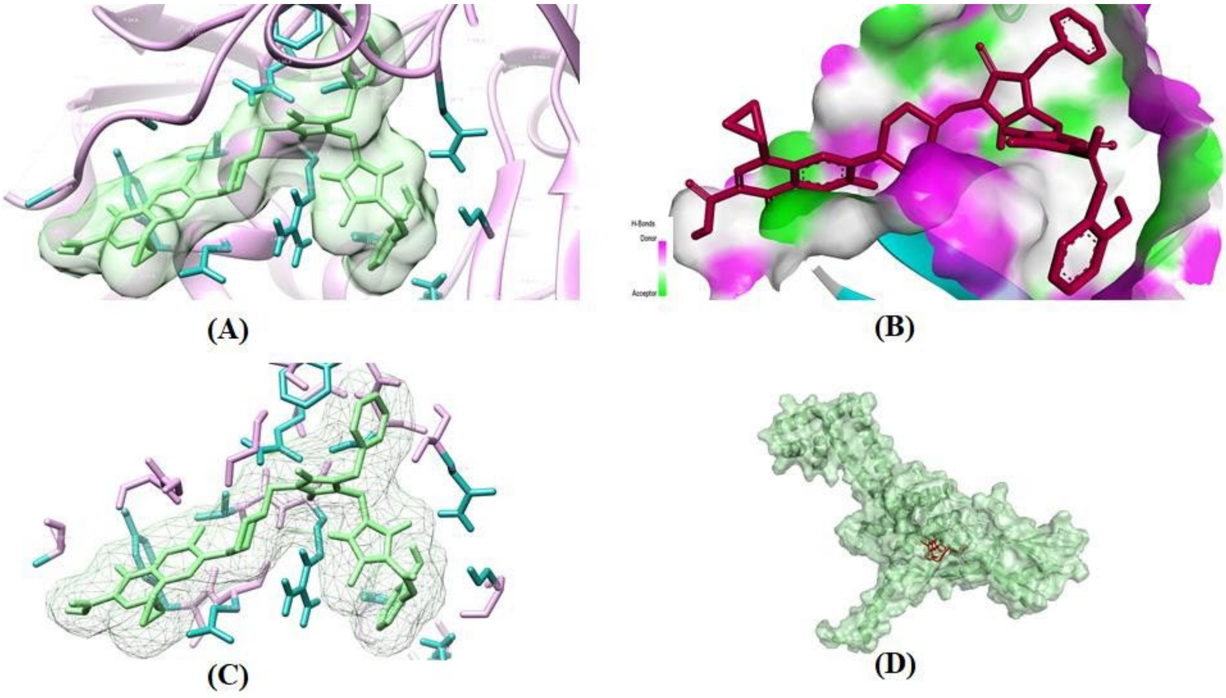

Figure S17. Molecular docking poses: (**A**) Ligand in protein pocket; (**B**) Active site; (**C**) Hydrogen bonding in solid for compound **15**; (**D**) Binding cavity of compound **15** in Topoisomerase II (PDB: 5CDQ).

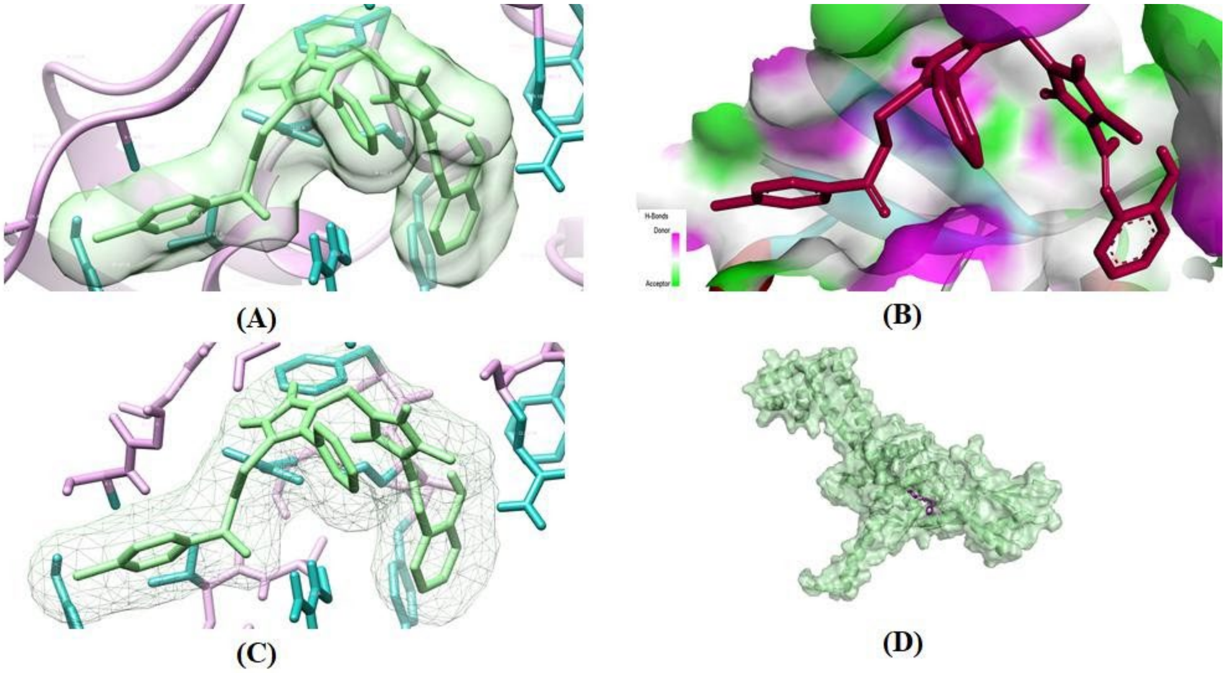

Figure S18. Molecular docking poses: (**A**) Ligand in protein pocket; (**B**) Active site; (**C**) Hydrogen bonding in solid for compound **16**; (**D**) Binding cavity of compound **16** in Topoisomerase II (PDB: 5CDQ).

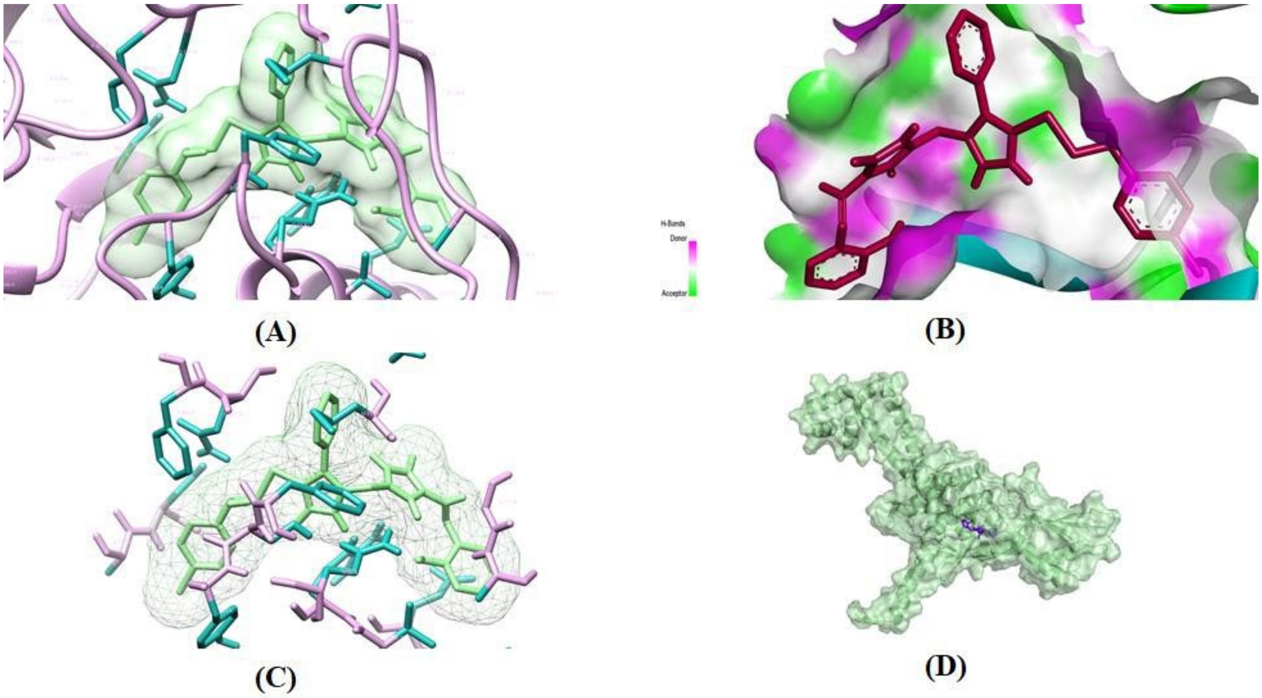

Figure S19. Molecular docking poses: (**A**) Ligand in protein pocket; (**B**) Active site; (**C**) Hydrogen bonding in solid for compound **17**; (**D**) Binding cavity of compound **17** in Topoisomerase II (PDB: 5CDQ).

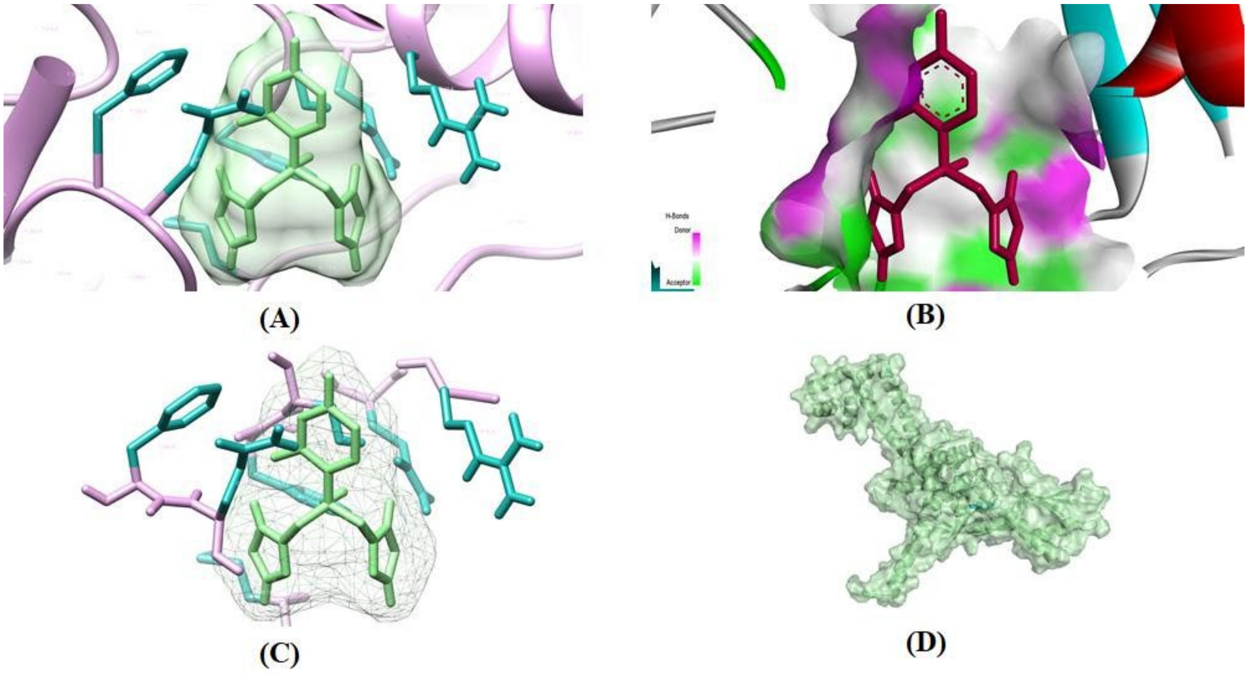

Figure S20. Molecular docking poses: (**A**) Ligand in protein pocket; (**B**) Active site; (**C**) Hydrogen bonding in solid for reference drug Fluconazole; (**D**) Binding cavity of reference drug Fluconazole in Topoisomerase II (PDB: 5CDQ).

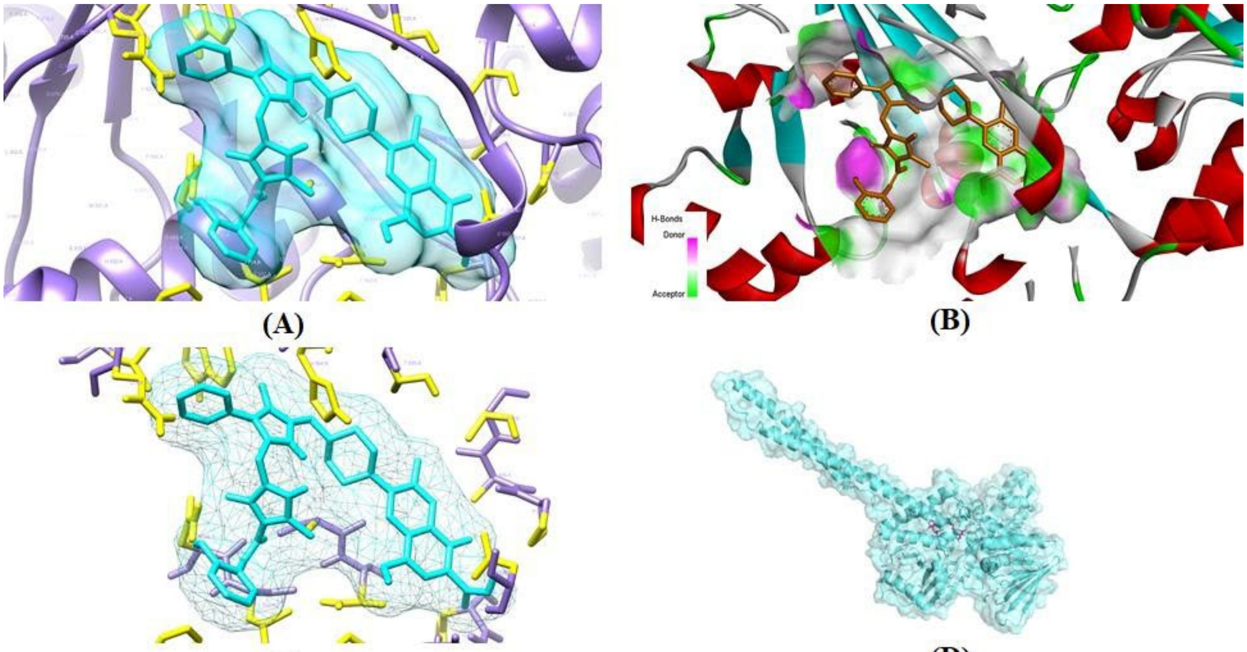

Figure S21. Molecular docking poses: (**A**) Ligand in protein pocket; (**B**) Active site; (**C**) Hydrogen bonding in solid for compound **10**; (**D**) Binding cavity of compound **10** in LSD1 (PDB: 2Z3Y).

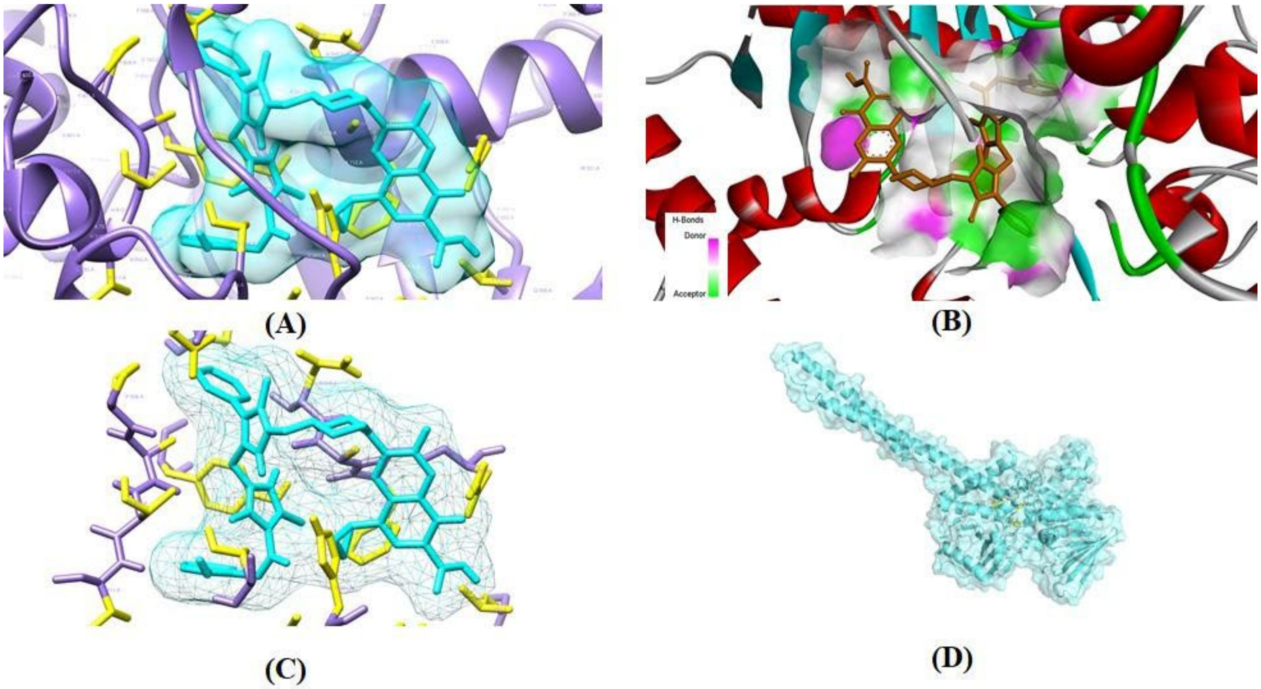

Figure S22. Molecular docking poses: (**A**) Ligand in protein pocket; (**B**) Active site; (**C**) Hydrogen bonding in solid for compound **11**; (**D**) Binding cavity of compound **11** in LSD1 (PDB: 2Z3Y).

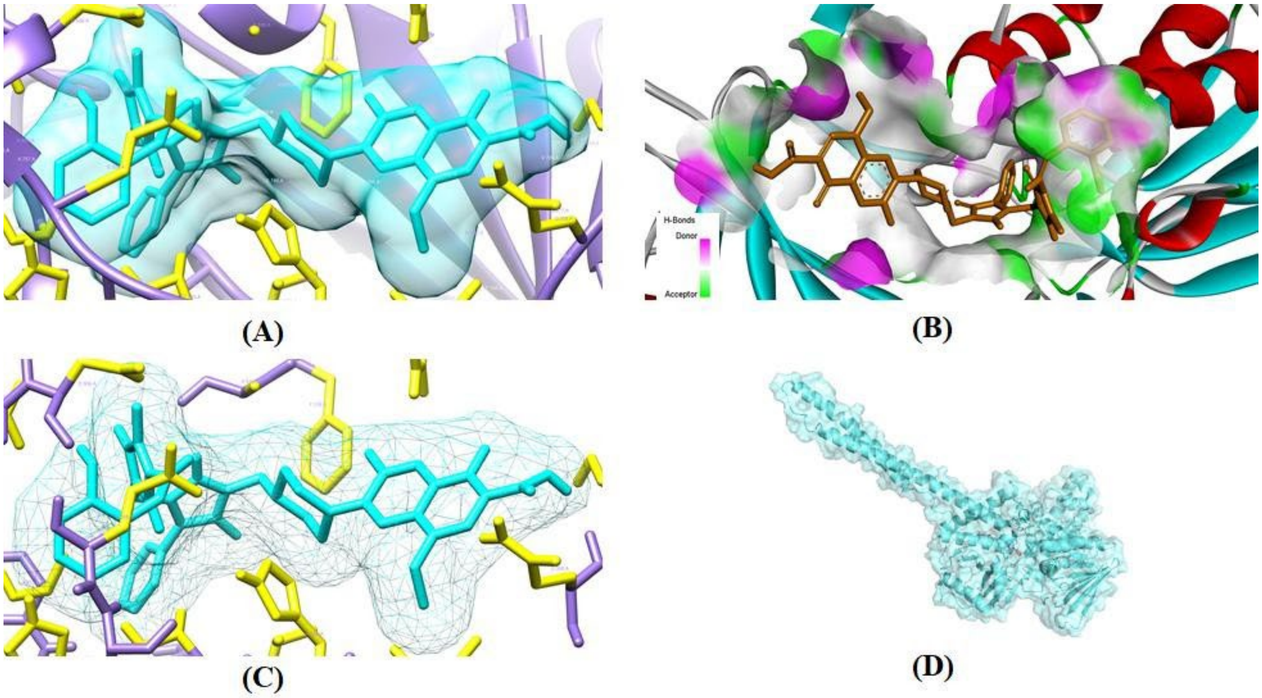

Figure S23. Molecular docking poses: (**A**) Ligand in protein pocket; (**B**) Active site; (**C**) Hydrogen bonding in solid for compound **12**; (**D**) Binding cavity of compound **12** in LSD1 (PDB: 2Z3Y).

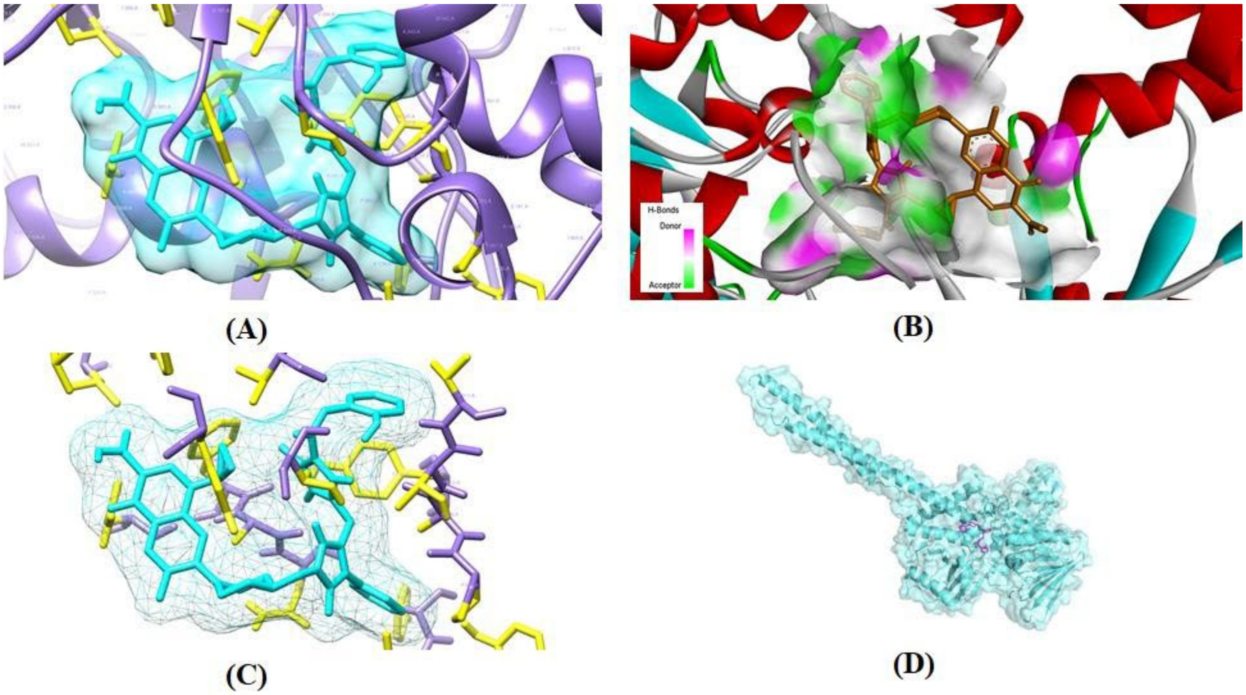

Figure S24. Molecular docking poses: (**A**) Ligand in protein pocket; (**B**) Active site; (**C**) Hydrogen bonding in solid for compound **13**; (**D**) Binding cavity of compound **13** in LSD1 (PDB: 2Z3Y).

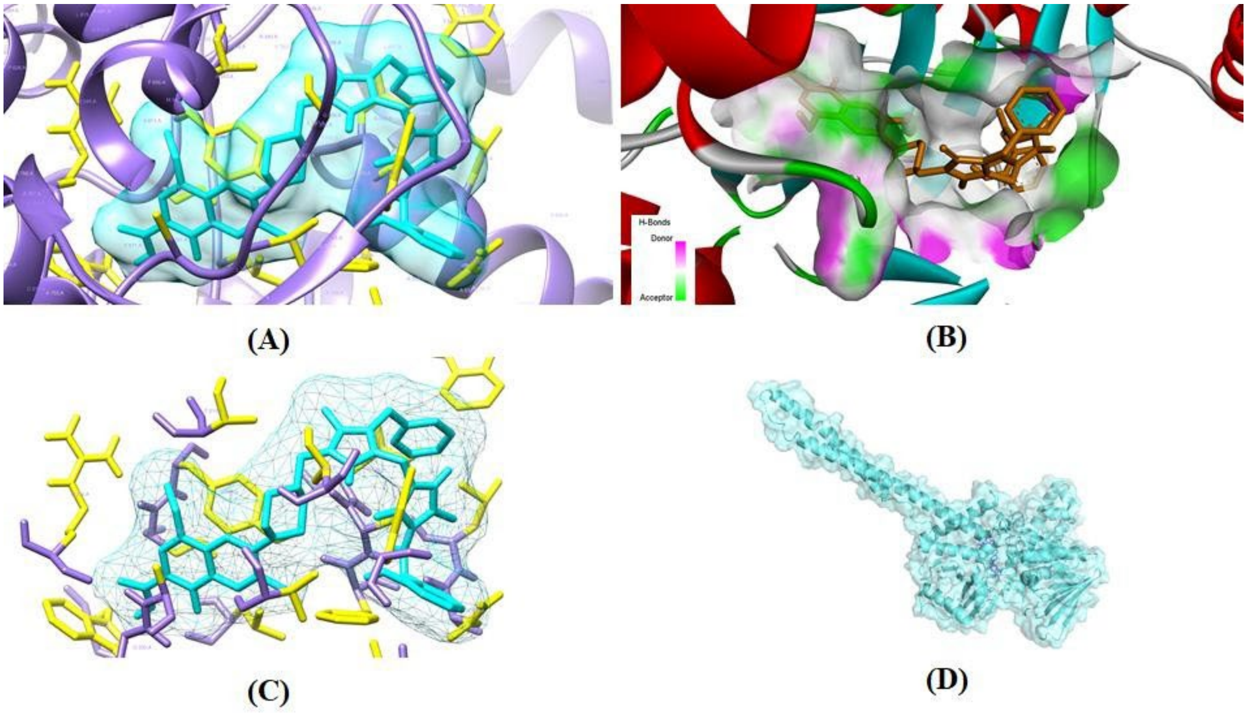

Figure S25. Molecular docking poses: (**A**) Ligand in protein pocket; (**B**) Active site; (**C**) Hydrogen bonding in solid for compound **14**; (**D**) Binding cavity of compound **14** in LSD1 (PDB: 2Z3Y).

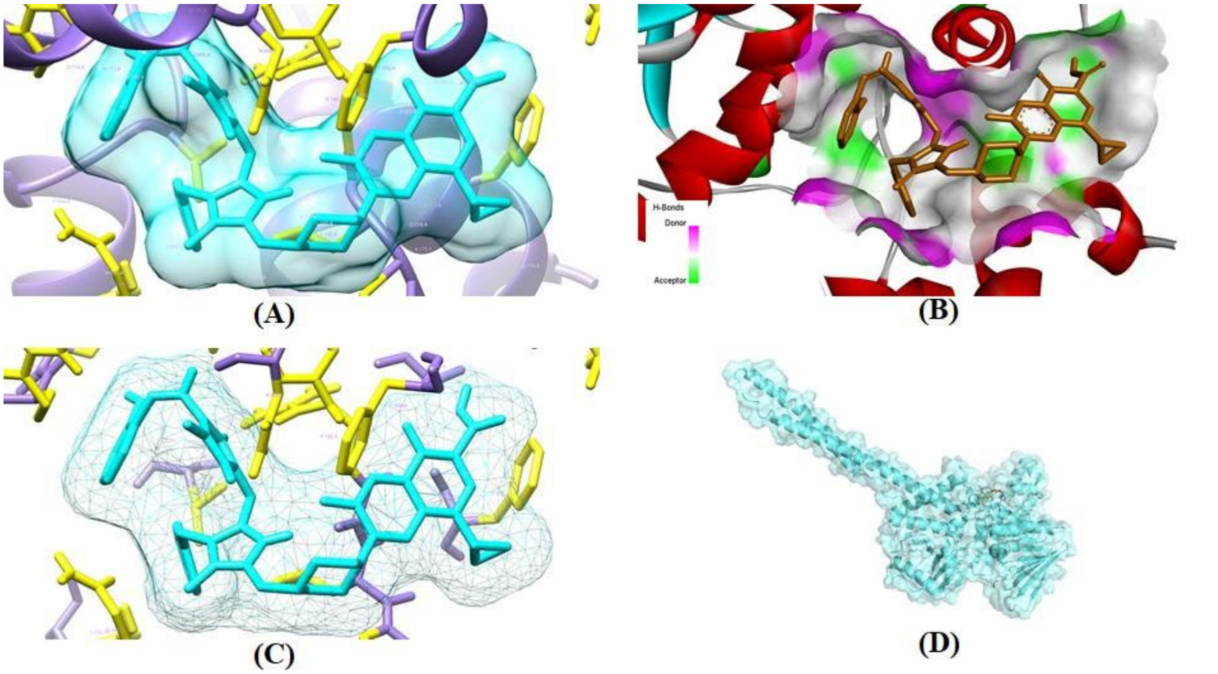

Figure S26. Molecular docking poses: (**A**) Ligand in protein pocket; (**B**) Active site; (**C**) Hydrogen bonding in solid for compound **15**; (**D**) Binding cavity of compound **15** in LSD1 (PDB: 2Z3Y).

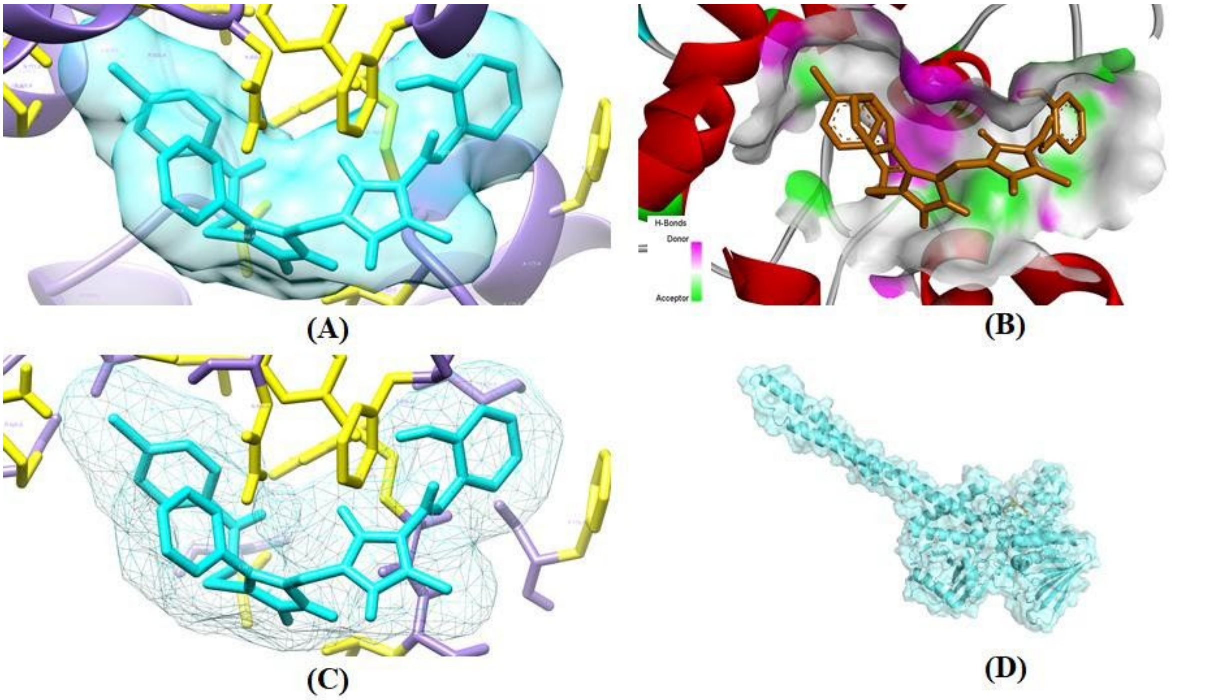

Figure S27. Molecular docking poses: (**A**) Ligand in protein pocket; (**B**) Active site; (**C**) Hydrogen bonding in solid for compound **16**; (**D**) Binding cavity of compound **16** in LSD1 (PDB: 2Z3Y).

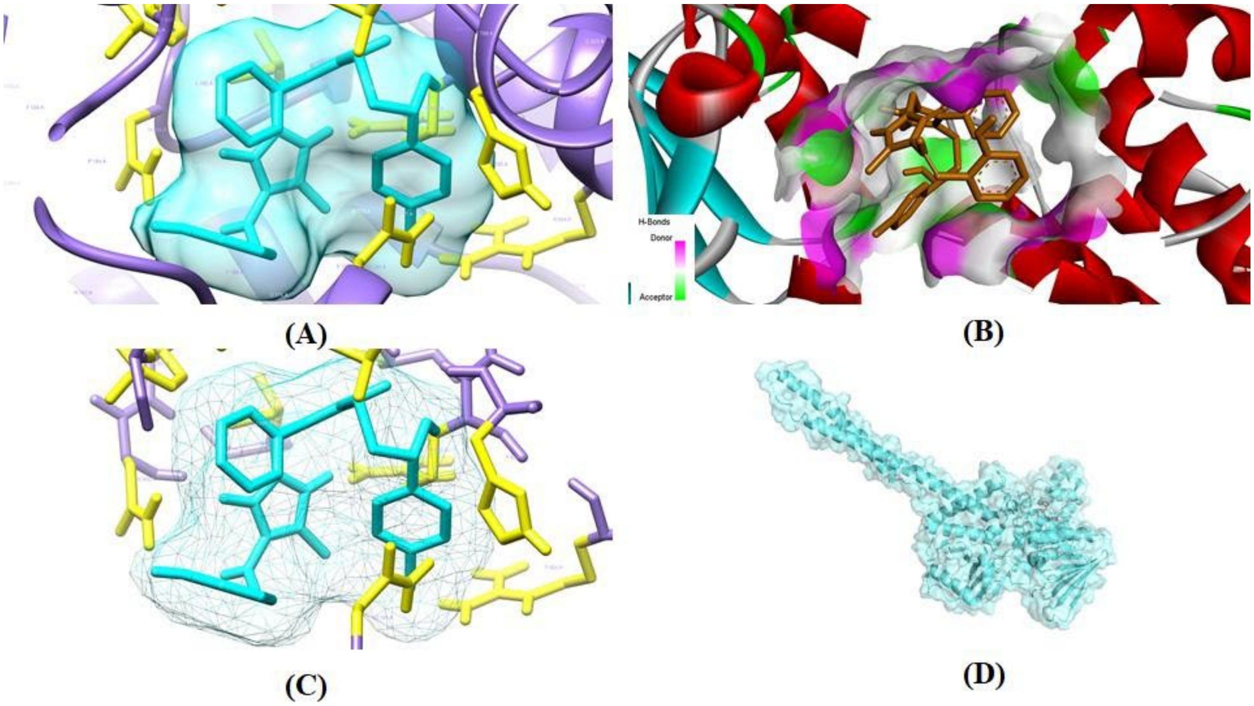

Figure S28. Molecular docking poses: (**A**) Ligand in protein pocket; (**B**) Active site; (**C**) Hydrogen bonding in solid for compound **17**; (**D**) Binding cavity of compound **17** in LSD1 (PDB: 2Z3Y).

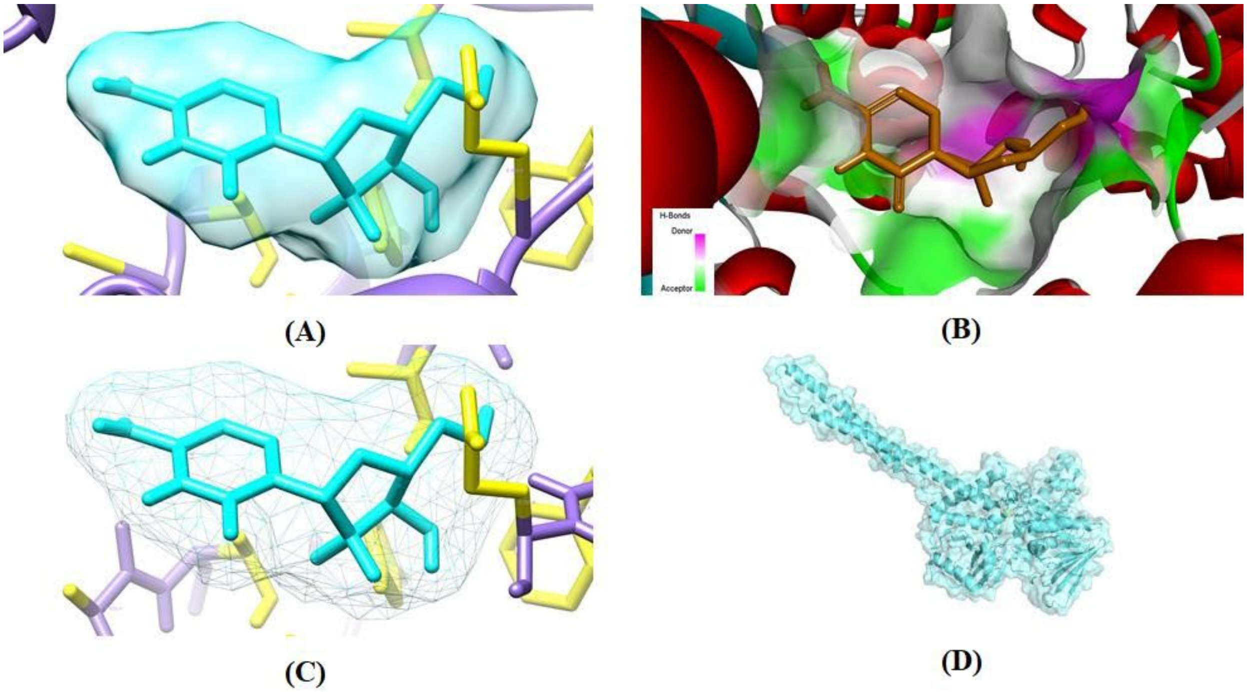

Figure S29. Molecular docking poses: (**A**) Ligand in protein pocket; (**B**) Active site; (**C**) Hydrogen bonding in solid for reference drug Gemcitabine; (**D**) Binding cavity of reference drug Gemcitabine in LSD1 (PDB: 2Z3Y).

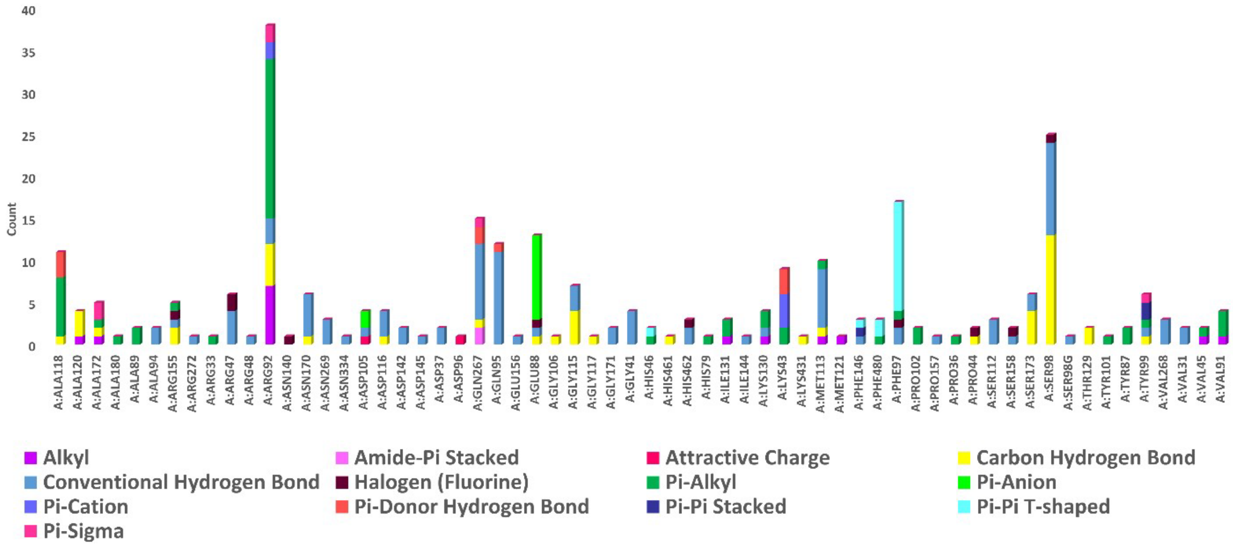

Figure S30. Residues in interaction in antimicrobial and anticancer compounds and the reference drugs with Topoisomerase II (5CDQ).

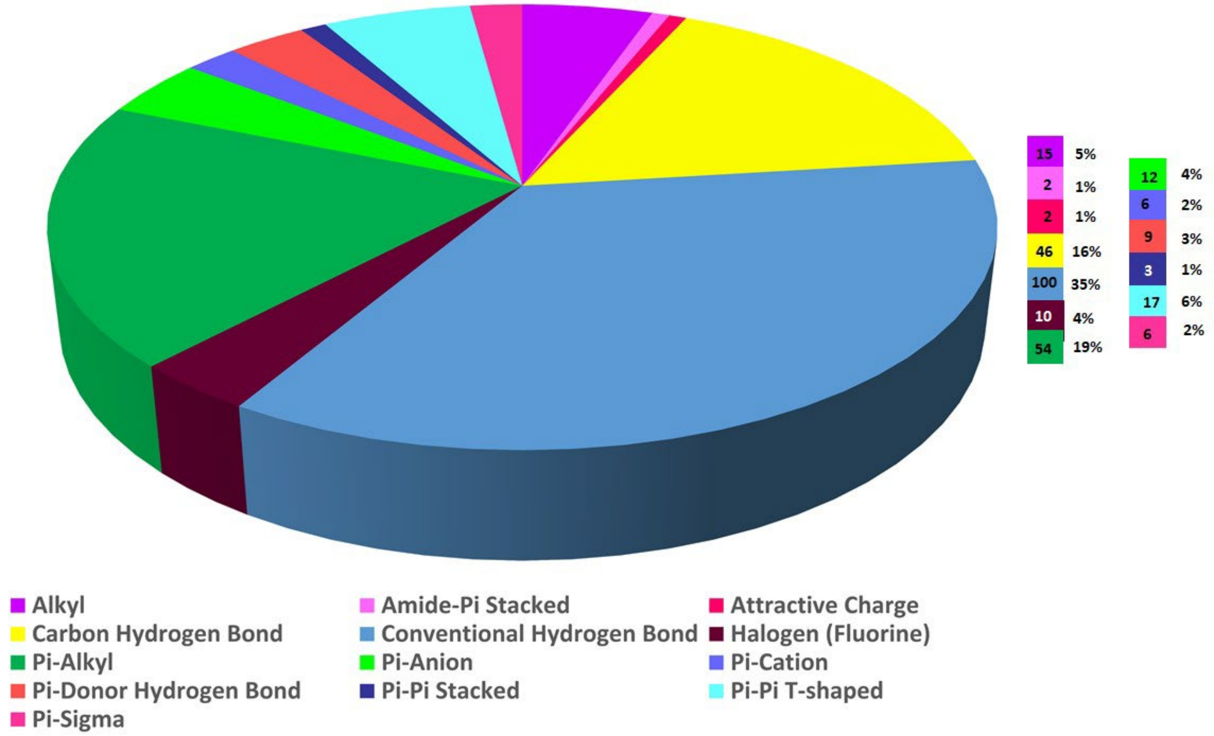

Figure S31. The distribution of non-covalent interactions, in antimicrobial and anticancer compounds and the reference drugs with Topoisomerase II (5CDQ).

	A:ALA118	A:ALA120	A:ALA172	A:ALA180	A:ALA89	A:ALA94	A:ARG155	A:ARG272	A:ARG33	A:ARG47	A:ARG48	A:ARG92	A:ASN140	A:ASN170	A:ASN269	A:ASN334	A:ASP105	A:ASP116	A:ASP142	A:ASP145	A:ASP37	A:ASP96	A:GLN267	A:GLN95	A:GLU156	A:GLU88	A:GLY106	A:GLY115	A:GLY117	A:GLY171	A:GLY41
Compound 1	0	0	0	0	0	0	0	0	0	0	0	1	0	0	0	0	0	0	0	0	0	0	1	0	0	0	0	0	0	0	0
Compound 2	0	0	0	0	0	0	0	0	0	0	0	2	0	1	0	0	0	0	0	0	0	0	0	0	0	0	0	0	0	0	0
Compound 3	0	0	0	0	0	0	0	0	0	0	0	2	0	0	0	0	0	0	0	0	0	0	1	0	0	0	0	0	0	0	0
Compound 4	0	0	0	0	1	0	0	0	0	0	0	4	0	0	0	0	0	0	0	0	0	0	2	0	0	0	0	0	0	0	0
Compound 5	0	0	0	0	0	0	0	1	0	0	0	0	0	0	2	0	0	0	0	0	0	0	0	0	0	0	0	0	0	0	0
Compound 6	0	0	0	0	0	0	0	0	0	0	0	2	0	0	0	0	0	0	0	0	0	0	0	0	0	0	0	0	0	0	0
Compound 7	0	0	0	0	0	0	0	0	0	0	0	1	0	0	0	0	0	0	0	0	0	0	0	0	0	0	0	0	0	0	0
Compound 8	0	0	0	0	0	0	0	0	0	0	0	1	0	0	1	0	0	0	0	0	0	0	1	0	0	0	0	1	0	0	0
Compound 9	0	0	0	0	0	0	0	0	0	0	0	0	0	0	0	0	1	0	0	0	0	0	0	0	0	0	0	0	0	0	0
Compound 10	0	0	0	0	1	0	0	0	0	0	0	6	0	0	0	0	0	1	0	0	0	0	1	2	0	0	0	2	0	0	0
Compound 11	0	0	0	0	0	0	0	0	1	0	0	2	0	0	0	0	0	0	0	0	0	0	0	1	0	0	0	0	0	0	0
Compound 12	1	1	0	0	0	0	0	0	0	0	0	1	0	0	0	0	0	0	0	0	0	0	1	0	0	4	0	1	0	0	0
Compound 13	1	1	0	0	0	0	0	0	0	0	0	1	0	0	0	0	0	1	0	0	0	0	1	1	0	2	0	2	0	0	0
Compound 14	0	0	0	1	0	0	0	0	0	0	0	1	0	0	0	1	0	0	0	0	0	0	1	1	0	0	0	0	0	0	0
Compound 15	1	1	0	0	0	0	0	0	0	0	0	3	0	0	0	0	0	0	0	0	0	0	1	1	0	2	0	0	0	0	0
Compound 16	2	0	0	0	0	0	0	0	0	0	0	3	0	0	0	0	0	1	0	0	0	0	0	0	0	1	0	0	0	0	0
Compound 17	2	0	1	0	0	0	0	0	0	0	0	1	0	0	0	0	0	0	0	0	0	0	0	2	0	1	0	0	1	0	0
Compound 18	0	0	0	0	0	2	0	0	0	0	0	0	0	0	0	0	3	0	0	0	0	1	0	0	0	0	1	0	0	0	0
Compound 19	0	0	1	0	0	0	0	0	0	0	0	1	0	1	0	0	0	0	0	0	0	0	0	0	0	0	0	0	0	0	0
Compound 20	0	0	0	0	0	0	0	0	0	0	0	0	0	0	0	0	0	1	0	0	0	0	0	1	0	0	0	1	0	0	0
Ampicillin	2	0	0	0	0	0	0	0	0	0	0	0	0	0	0	0	0	0	0	0	0	0	2	1	0	2	0	0	0	0	0
Cefatrizine	0	0	2	0	0	0	0	0	0	0	0	1	0	1	0	0	0	0	0	0	0	0	0	1	0	0	0	0	0	1	2
Fluconazole	0	0	0	0	0	0	0	0	0	0	0	0	0	0	0	0	0	0	0	0	0	0	1	1	0	0	0	0	0	0	0
Gemcitabine	0	0	0	0	0	0	0	0	0	6	0	0	0	0	0	0	0	0	0	0	2	0	0	0	0	0	0	0	0	0	0
Itraconazole	2	1	0	0	0	0	0	0	0	0	0	3	0	0	0	0	0	0	0	0	0	0	1	0	0	1	0	0	0	1	0
Ribavirin	0	0	0	0	0	0	0	0	0	0	1	0	0	0	0	0	0	0	0	0	0	0	0	0	1	0	0	0	0	0	0
Rufinamide	0	0	0	0	0	0	5	0	0	0	0	0	1	0	0	0	0	0	2	1	0	0	0	0	0	0	0	0	0	0	0
Streptomycin	0	0	0	0	0	0	0	0	0	0	0	1	0	3	0	0	0	0	0	0	0	0	0	0	0	0	0	0	0	0	2
Tazobactam	0	0	1	0	0	0	0	0	0	0	0	1	0	0	0	0	0	0	0	0	0	0	1	0	0	0	0	0	0	0	0

	A:HIS46	A:HIS461	A:HIS462	A:HIS79	A:ILE131	A:ILE144	A:LYS130	A:LYS43	A:LYS431	A:MET113	A:MET121	A:PHE146	A:PHE480	A:PHE97	A:PRO102	A:PRO157	A:PRO36	A:PRO44	A:SER112	A:SER158	A:SER173	A:SER98	A:SER98G	A:THR129	A:TYR101	A:TYR87	A:TYR99	A:VAL268	A:VAL31	A:VAL45	A:VAL91
Compound 1	0	0	0	0	0	0	0	0	0	3	0	0	0	2	0	0	0	0	0	0	0	1	0	0	0	0	0	1	0	0	0
Compound 2	0	0	0	0	0	0	0	0	0	0	0	0	0	2	0	0	0	0	0	0	1	2	0	0	0	0	0	0	0	0	0
Compound 3	0	0	0	0	0	0	0	0	0	0	0	0	0	2	0	0	0	0	0	0	0	2	0	0	0	0	0	0	0	0	0
Compound 4	0	0	1	0	0	0	0	0	0	0	0	0	0	0	0	0	0	0	0	0	0	1	0	0	0	0	1	0	0	1	0
Compound 5	0	0	0	0	0	0	0	0	0	0	0	0	0	1	0	0	0	0	1	0	0	0	0	0	0	0	0	0	0	0	0
Compound 6	0	0	0	0	0	0	0	3	0	1	0	0	0	1	0	0	0	0	0	0	0	3	0	0	0	0	0	0	0	0	0
Compound 7	0	0	0	0	0	0	0	0	0	1	0	0	0	1	0	0	0	0	0	0	0	1	0	0	0	0	0	0	0	0	0
Compound 8	0	0	0	0	0	0	0	0	0	1	0	0	0	0	0	0	0	0	0	0	0	1	0	0	0	0	0	1	0	0	0
Compound 9	0	0	0	0	1	0	2	0	0	0	0	0	1	0	1	0	0	0	0	0	0	0	0	1	0	0	0	0	0	0	0
Compound 10	0	0	2	0	0	0	0	3	0	0	0	0	0	0	0	0	0	0	0	0	0	1	0	0	0	0	0	0	0	0	1
Compound 11	0	0	0	1	0	0	0	0	0	0	0	0	0	0	0	0	0	0	0	0	0	2	0	0	0	0	0	0	0	1	0
Compound 12	0	0	0	0	0	0	0	0	0	0	0	0	0	1	0	0	0	0	0	0	0	0	0	0	0	0	0	0	0	0	0
Compound 13	0	0	0	0	0	0	0	0	0	0	0	0	0	1	0	0	0	0	0	0	0	0	0	0	0	0	0	0	0	0	0
Compound 14	0	0	0	0	0	0	0	0	0	0	0	0	0	0	0	0	0	0	1	0	0	0	0	0	0	0	0	0	0	0	0
Compound 15	0	0	0	0	0	0	0	2	0	0	0	0	0	2	0	0	0	0	0	0	0	0	0	0	0	0	0	0	0	0	0
Compound 16	0	0	0	0	0	0	0	0	0	0	0	0	0	0	0	0	0	0	1	0	0	1	0	0	0	1	0	0	0	0	1
Compound 17	0	0	0	0	0	0	0	0	0	0	0	0	0	1	0	0	0	0	0	0	0	0	0	0	0	0	1	0	0	0	1
Compound 18	0	0	0	0	2	0	2	0	0	0	0	0	2	0	1	0	0	0	0	0	0	0	0	1	1	0	0	0	0	0	0
Compound 19	0	1	0	0	0	0	0	0	1	0	0	0	0	0	0	0	0	0	0	0	2	0	0	0	0	0	1	0	0	0	0
Compound 20	0	0	0	0	0	0	0	0	0	2	0	0	0	1	0	0	0	0	0	0	0	0	0	0	0	0	0	0	0	0	0
Ampicillin	0	0	0	0	0	0	0	0	0	0	0	0	0	0	0	0	0	0	0	0	0	1	0	0	0	0	0	0	0	0	1
Cefatrizine	1	0	0	0	0	0	0	1	0	1	0	0	0	1	0	0	1	0	0	0	0	4	0	0	0	0	1	1	0	0	0
Fluconazole	0	0	0	0	0	0	0	0	0	1	0	0	0	1	0	0	0	0	0	0	0	2	0	0	0	0	0	0	0	0	0
Gemcitabine	0	0	0	0	0	0	0	0	0	0	0	0	0	0	0	0	0	2	0	1	0	0	0	0	0	0	0	0	0	0	0
Itraconazole	1	0	0	0	0	0	0	0	0	0	1	0	0	0	0	0	0	0	0	0	1	1	0	0	0	1	1	0	0	0	0
Ribavirin	0	0	0	0	0	0	0	0	0	0	0	0	0	0	0	1	0	0	0	1	0	0	0	0	0	0	0	0	2	0	0
Rufinamide	0	0	0	0	0	1	0	0	0	0	0	3	0	0	0	0	0	0	0	0	0	0	0	0	0	0	0	0	0	0	0
Streptomycin	0	0	0	0	0	0	0	0	0	0	0	0	0	0	0	0	0	0	0	0	2	1	1	0	0	0	0	0	0	0	0
Tazobactam	0	0	0	0	0	0	0	0	0	0	0	0	0	0	0	0	0	0	0	0	0	1	0	0	0	0	1	0	0	0	0

Figure S32. A map illustrating the interaction between residues in antimicrobial and anticancer compounds and the reference drugs with Topoisomerase II (5CDQ).

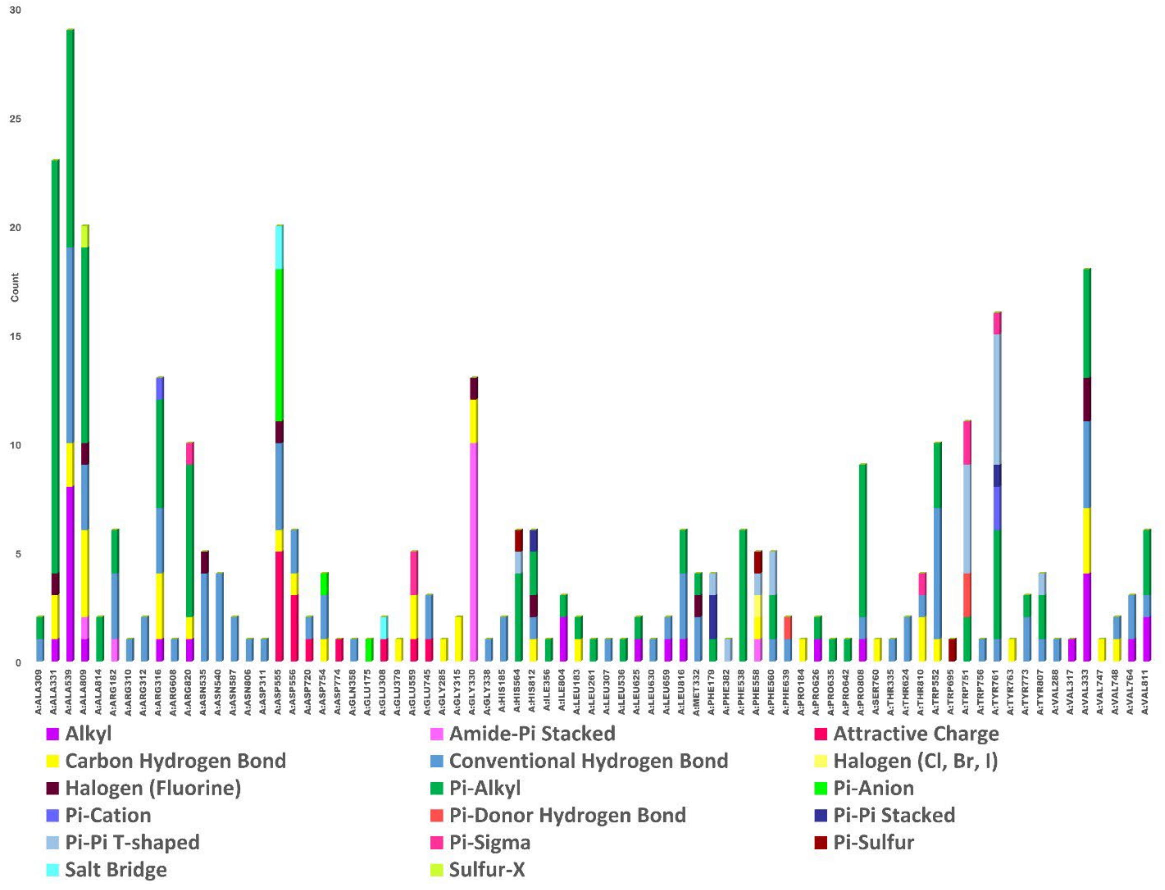

Figure S33. Residues in interaction in antimicrobial and anticancer compounds and the reference drugs with LSD1 (2Z3Y).

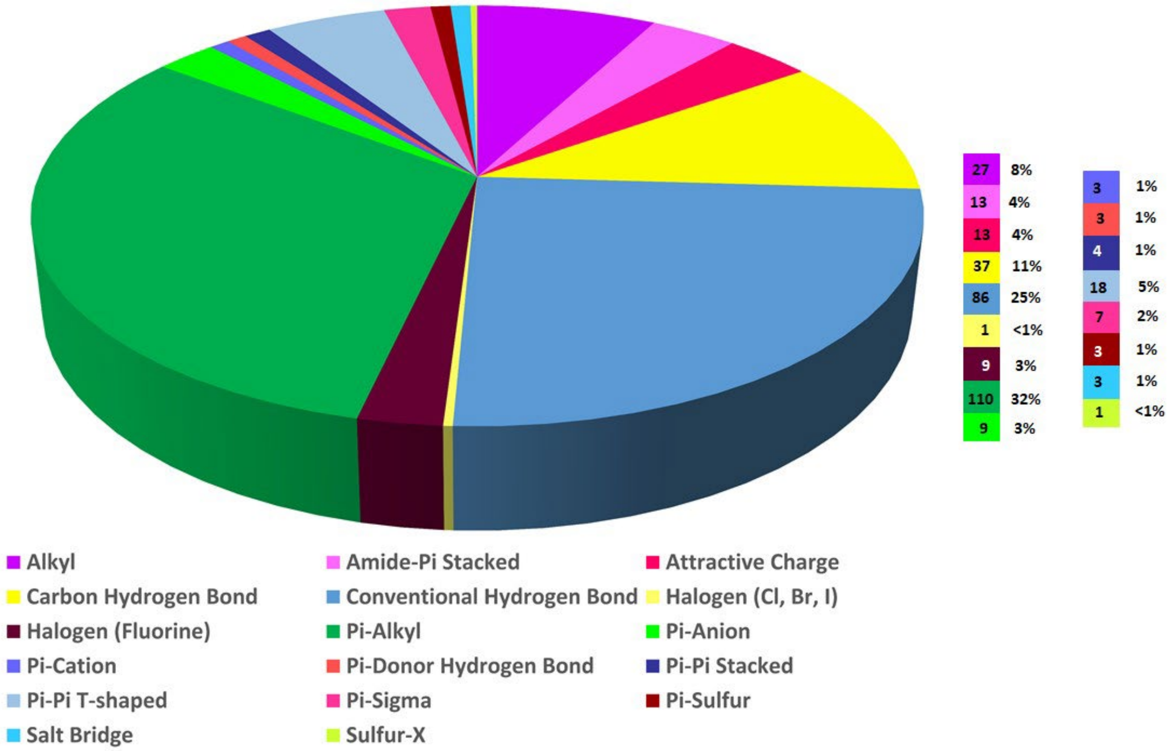

Figure S34. The distribution of non-covalent interactions in antimicrobial and anticancer compounds and the reference drugs with LSD1 (2Z3Y).

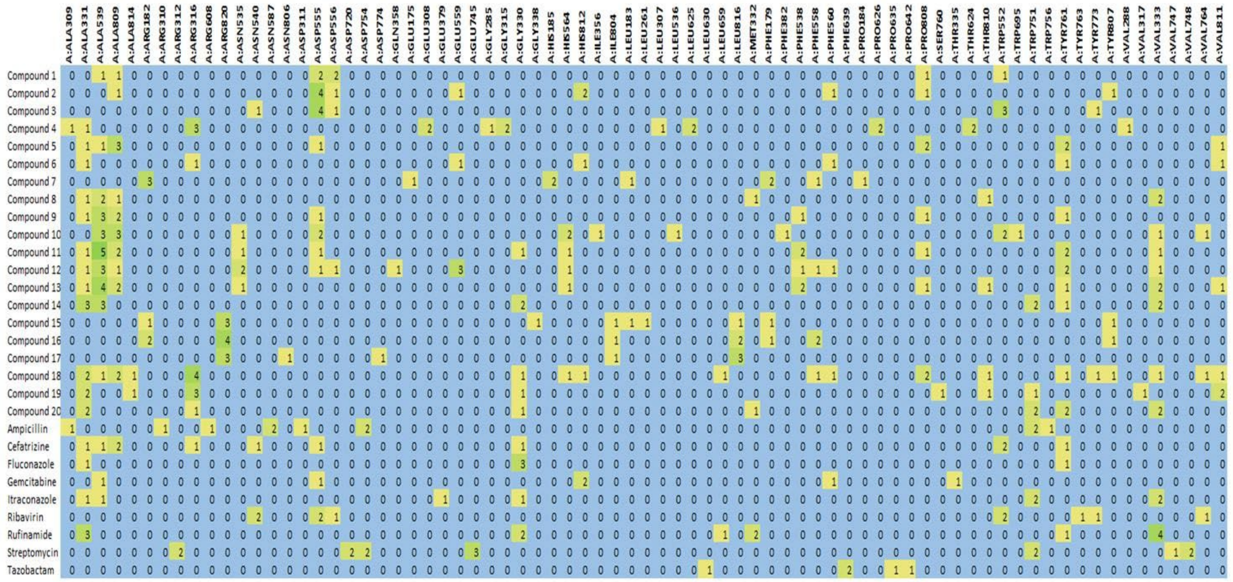

Figure S35. A map illustrating the interaction between residues in antimicrobial and anticancer compounds and the reference drugs with LSD1 (2Z3Y).

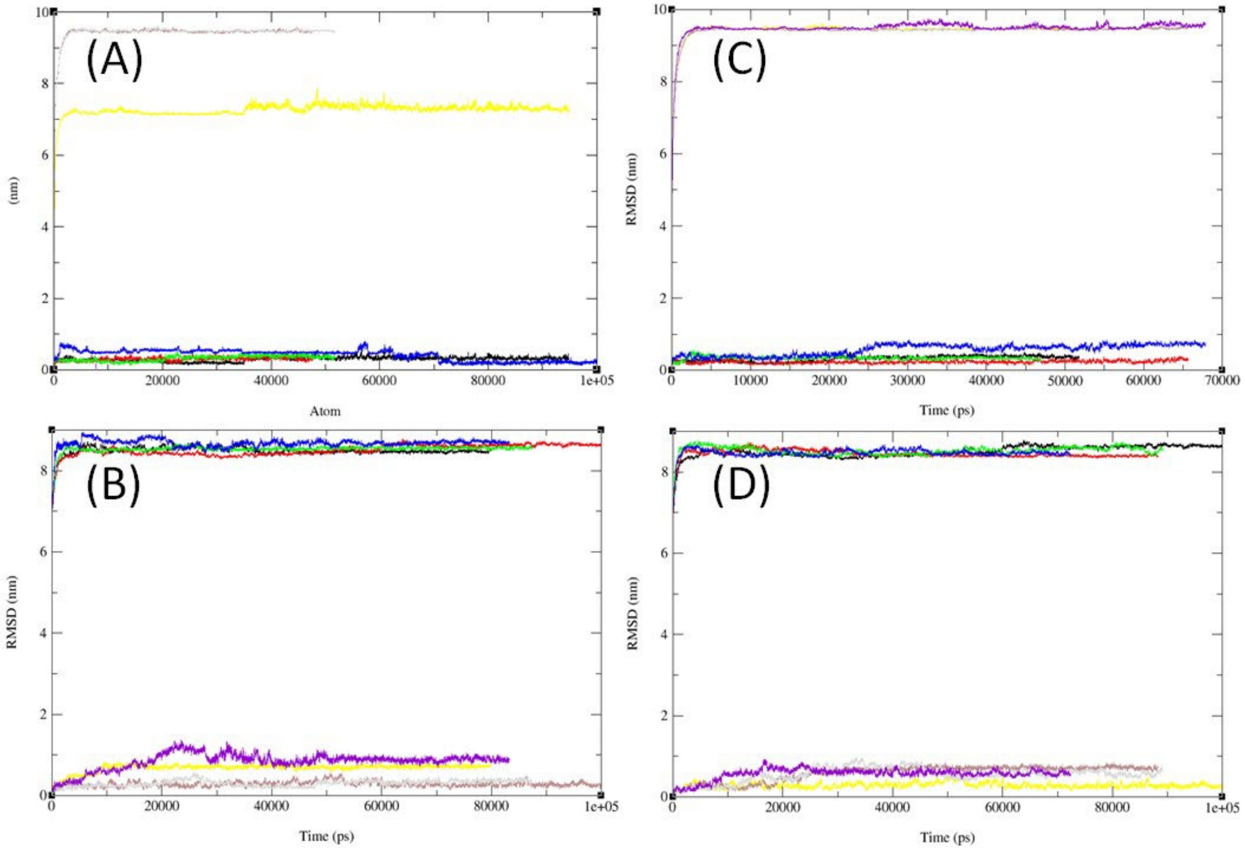

Figure S36. Progression of RMSD for (**A**) Compounds **12**, **14**, **15,** and reference drug fluconazole at 300 K and docked complex of the protein Topoisomerase II (PDB: 5CDQ) with compounds **12**, **14**, **15,** and reference drugs fluconazole at 300 K; (**B**) Compounds **13**, **16**, **17,** and reference drug gemcitabine at 300 K and docked complex of the protein LSD1 (PDB: 2Z3Y) with compounds **13**, **16**, **17,** and reference drugs gemcitabine at 300 K; (**C**) Compound **15** at 300, 305, 310, and 320 K; and target protein Topoisomerase II with compound **15** at 300, 305, 310, and 320 K; (**D**) Compound **16** at 300, 305, 310, and 320 K; and target protein LSD1 with compound **16** at 300, 305, 310, and 320 K.

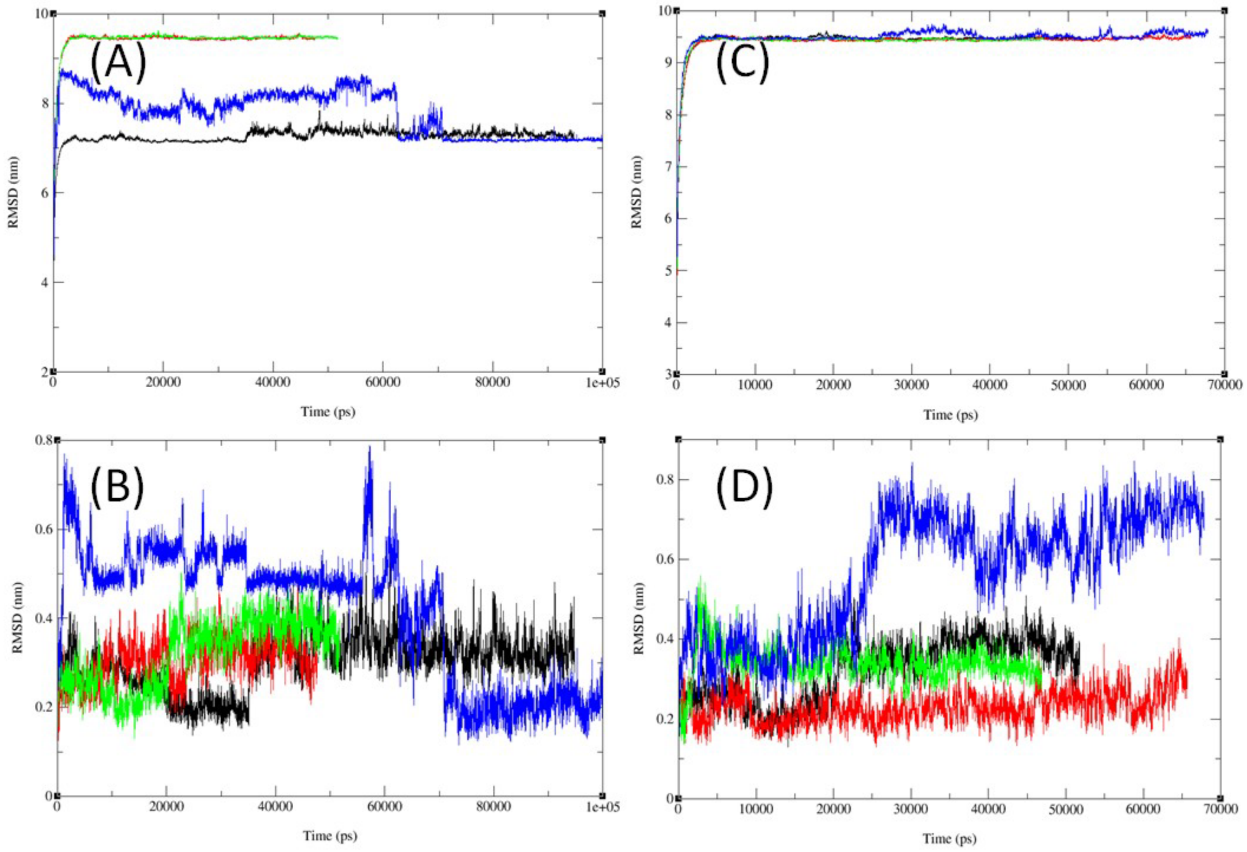

Figure S37. Progression of RMSD for (**A**) Compounds **12**, **14**, **15**, and reference drug fluconazole at 300 K; (**B**) Docked complex of the protein Topoisomerase II (PDB: 5CDQ) with compounds **12**, **14**, **15**, and reference drug fluconazole at 300 K; (**C**) Compound **15** at 300, 305, 310, and 320 K; and (**D**) Target protein Topoisomerase II with compound **15** at 300, 305, 310, and 320 K.

Table S51. RMSD values for compounds **12**, **14**, **15** and reference drug fluconazole in free form and complexed with topoisomerase II (5CDQ).

Compound	Compound RMSD (nm)	5CDQ-Compound RMSD (nm)
Compound **12** (black)	4.5 - 7.8	0.19 - 0.55
Compound **14** (red)	4.6 - 9.5	0.10 - 0.46
Compound **15** (green)	4.8 - 9.6	0.10 - 0.50
Fluconazole (blue)	4.5 - 8.7	0.25 - 0.79

Table S52. RMSD values for compound **15** at 300, 305, 310, and 320K in free from and complexed with topoisomerase II (5CDQ).

Temperature (K)	Compound **15** RMSD (nm)	5CDQ-Compound **15** RMSD (nm)
300 (black)	5.0 - 9.5	0.15 - 0.50
305 (red)	5.0 - 9.5	0.15 - 0.40
310 (green)	5.0 - 9.5	0.15 - 0.40
320 (blue)	5.0 - 9.75	0.18 - 0.83

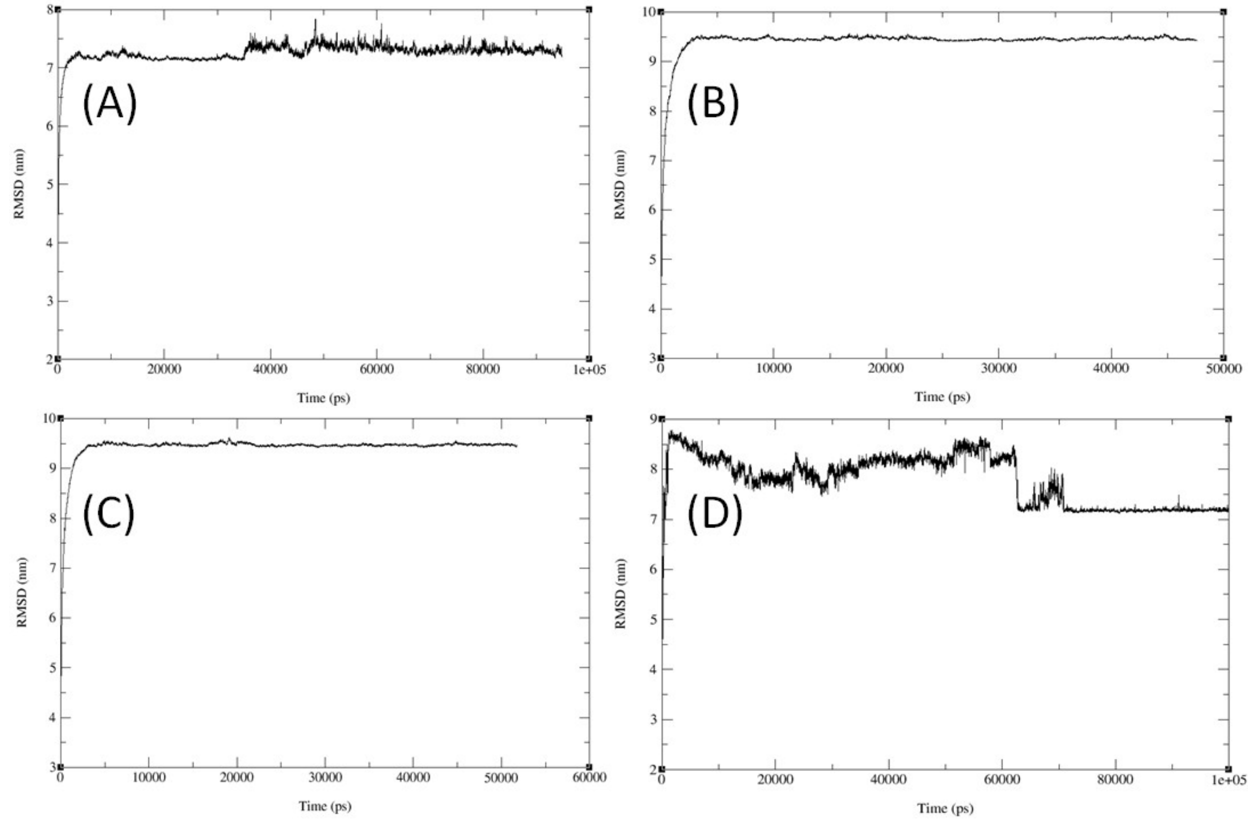

Figure S38. RMSD for (**A**) compound **12**; (**B**) compound **14**; and (**C**) compound **15**; (**D**) reference drug fluconazole during the 20 ns MD simulation.

Table S53. RMSD values for compounds **12**, **14**, **15**.

Compound	Compound RMSD (nm)
Compound **12**	4.5 - 7.8
Compound **14**	4.6 - 9.5
Compound **15**	4.8 - 9.6
Fluconazole	4.5 - 8.7

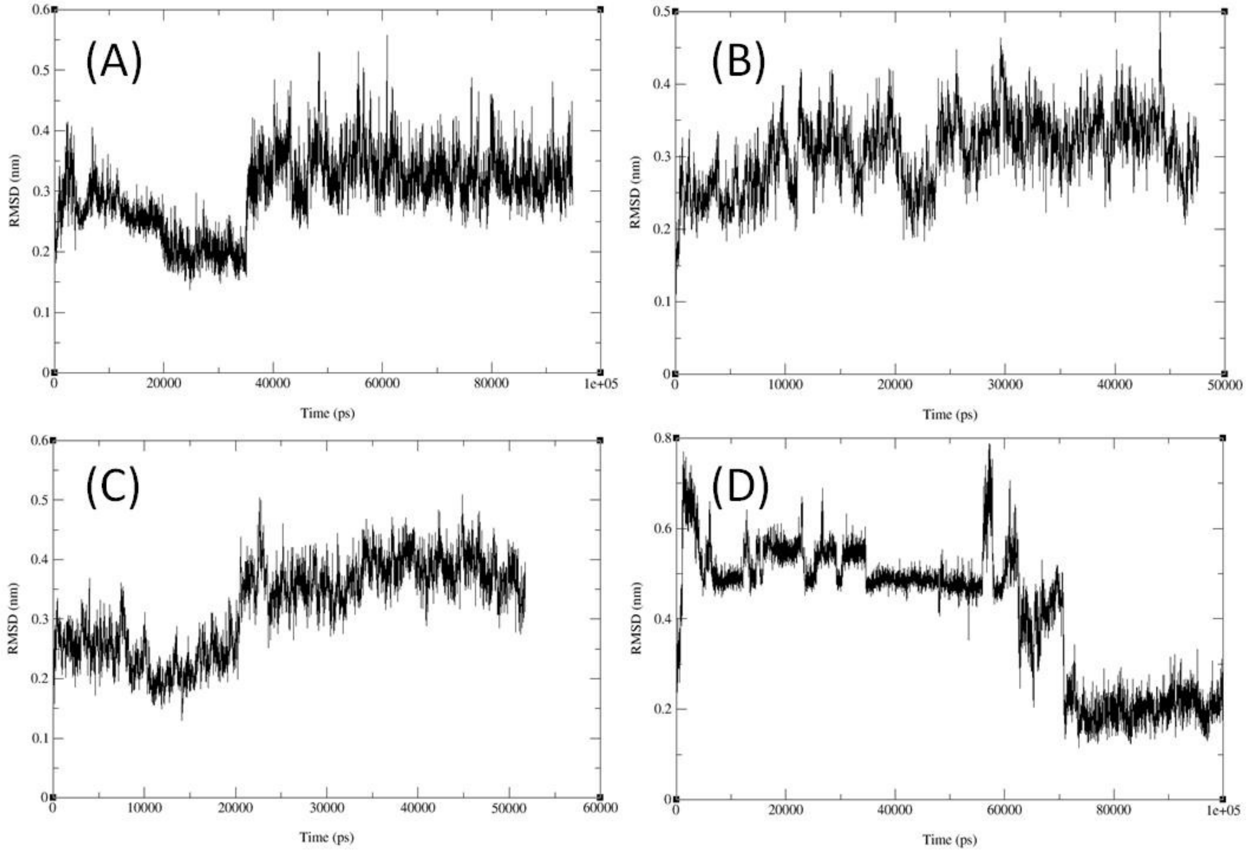

Figure S39. RMSD for protein Topoisomerase II-compound complex for (**A**) Topoisomerase II-compound **12**; (**B**) Topoisomerase II-compound **14**; and (**C**) Topoisomerase II-compound **15**; (**D**) Topoisomerase II-reference drug fluconazole during the 20 ns MD simulation.

Table S54. RMSD values for compounds **12**, **14**, **15** and reference drug fluconazole complexed with topoisomerase II (5CDQ).

Compound	5CDQ-Compound RMSD (nm)
Compound **12**	0.19 - 0.55
Compound **14**	0.10 - 0.46
Compound **15**	0.10 - 0.50
Fluconazole	0.25 - 0.79

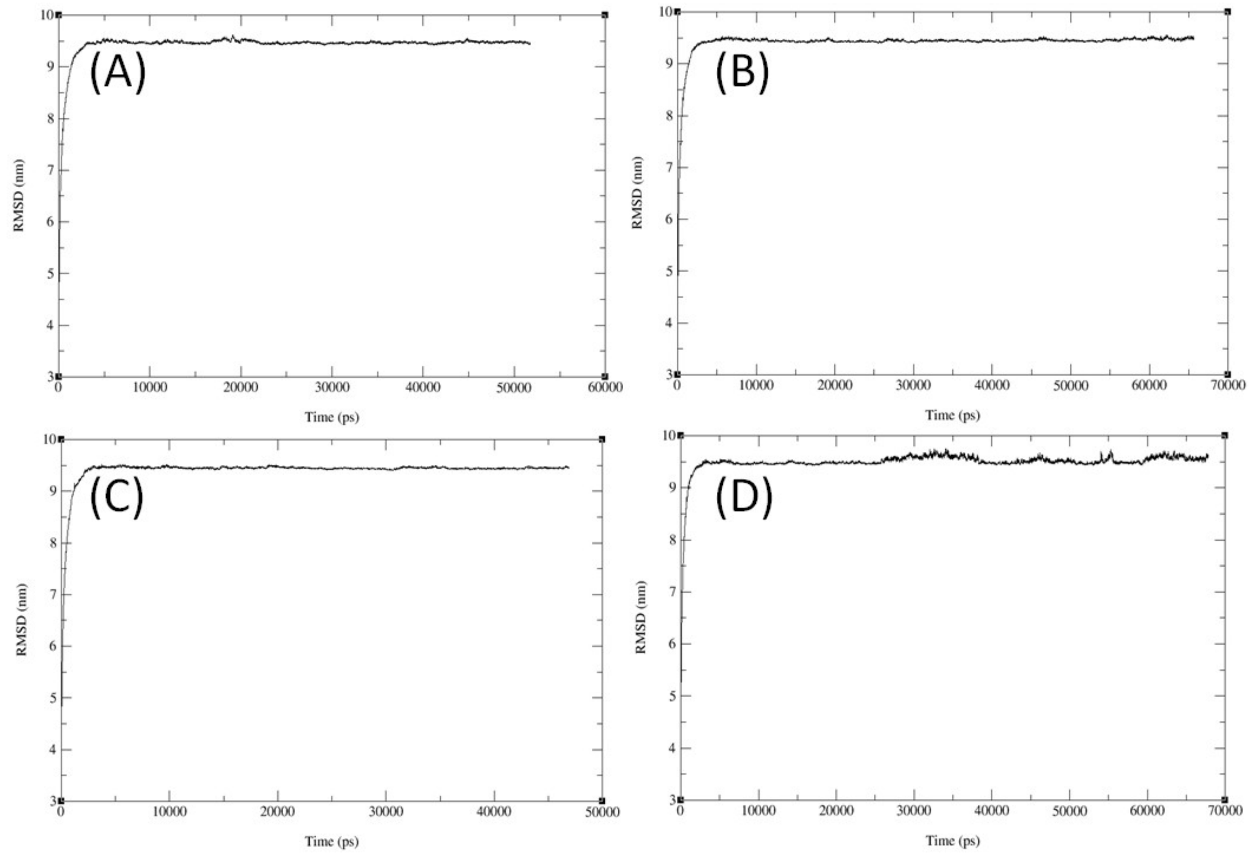

Figure S40. RMSD evolution for compound **15** (**A**) at 300 K; (**B**) at 305 K; (**C**) at 310 K; and (**D**) at 320 K; during the 20 ns MD simulation.

Table S55. RMSD values for compound **15** at 300, 305, 310, and 320K.

Temperature (K)	Compound **15** RMSD (nm)
300	5.0 - 9.5
305	5.0 - 9.5
310	5.0 - 9.5
320	5.0 - 9.75

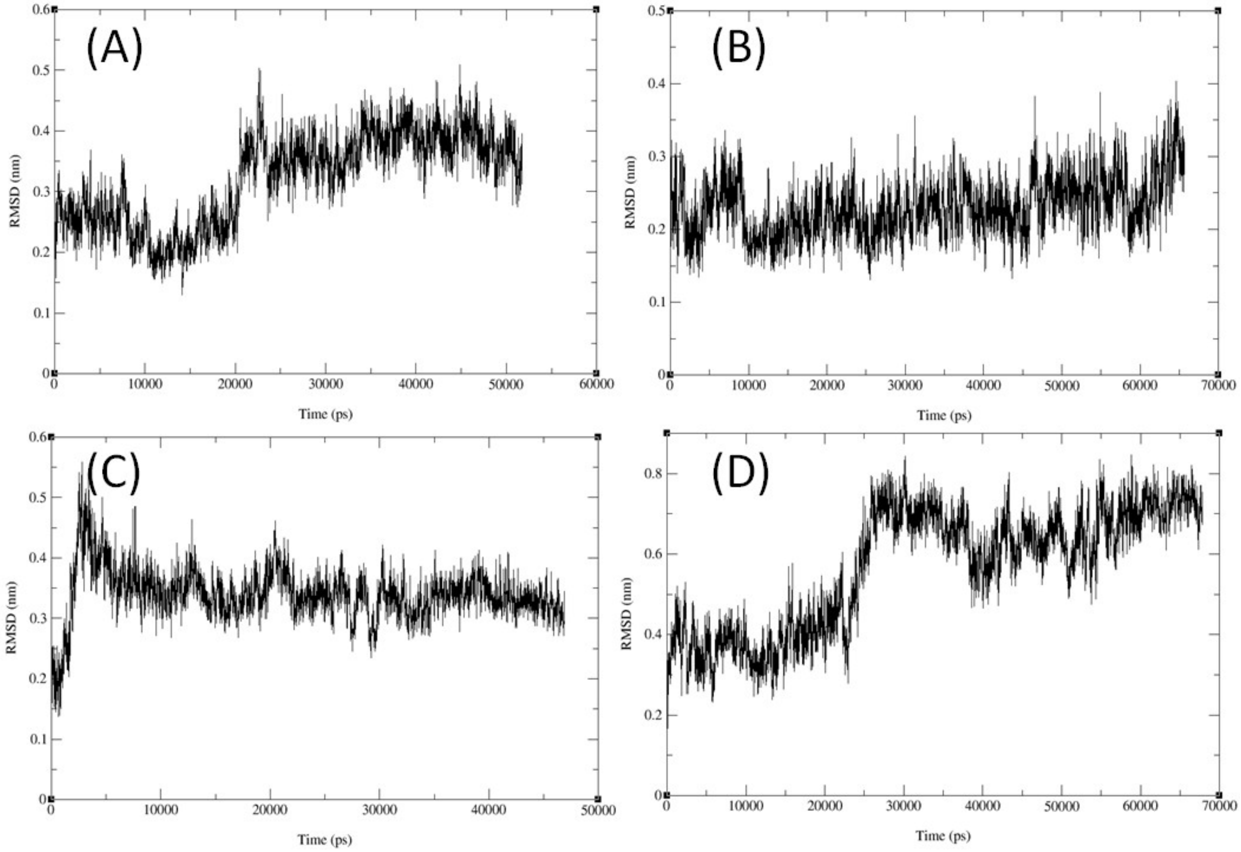

Figure S41. RMSD evolution for Topoisomerase II protein-compound **15** (**A**) at 300 K; (**B**) at 305 K; (**C**) at 310 K; and (**D**) at 320 K; during the 20 ns MD simulation.

Table S56. RMSD values for compound **15** at 300, 305, 310, and 320K complexed with topoisomerase II (5CDQ).

Temperature (K)	5CDQ-Compund 15Complex RMSD (nm)
300	0.15 - 0.50
305	0.15 - 0.40
310	0.15 - 0.40
320	0.18 - 0.83

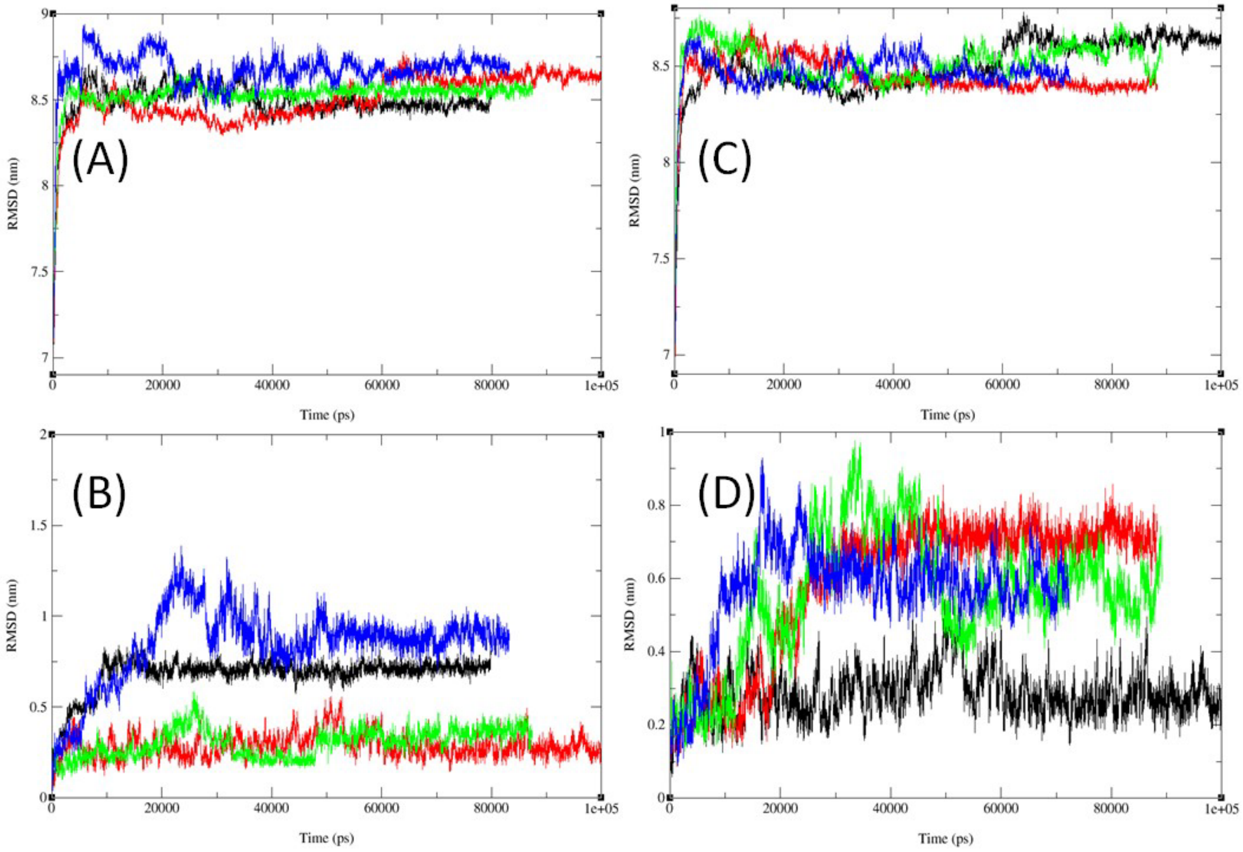

Figure S42. Progression of RMSD for (**A**) Compounds **13**, **16, 17**, and reference drug gemcitabine at 300 K; (**B**) Docked complex of the protein LSD (PDB: 2Z3Y) with compounds **13**, **16, 17**, and reference drug gemcitabine at 300 K; (**C**) Compound **16** at 300, 305, 310, and 320 K; and (**D**) Target protein LSD1 with compound **16** at 300, 305, 310, and 320 K.

Table S57. RMSD values for compounds **13**, **16**, **17** and reference drug gemcitabine in free form and complexed with LSD1 (2Z3Y).

Compound	Compound RMSD (nm)	2Z3Y-Compound RMSD (nm)
Compound **13** (black)	7.07-8.75	0.2-0.8
Compound **16** (red)	7.07-8.75	0.05-0.55
Compound **17** (green)	7.2-8.65	0.10 - 0.59
Gemcitabine (blue)	7.1-8.9	0.10-1.35

Table S58. RMSD values for compound **16** at 300, 305, 310, and 320K in free from and complexed with LSD1 (2Z3Y).

Temperature (K)	Compound **16** RMSD (nm)	2Z3Y-Compound **16** RMSD (nm)
300 (black)	7.1 - 9.0	0.05 - 0.55
305 (red)	7.0 - 9.0	0.10 - 0.84
310 (green)	7.2 - 9.0	0.10 - 0.99
320 (blue)	7.05 - 8.9	0.10 - 0.90

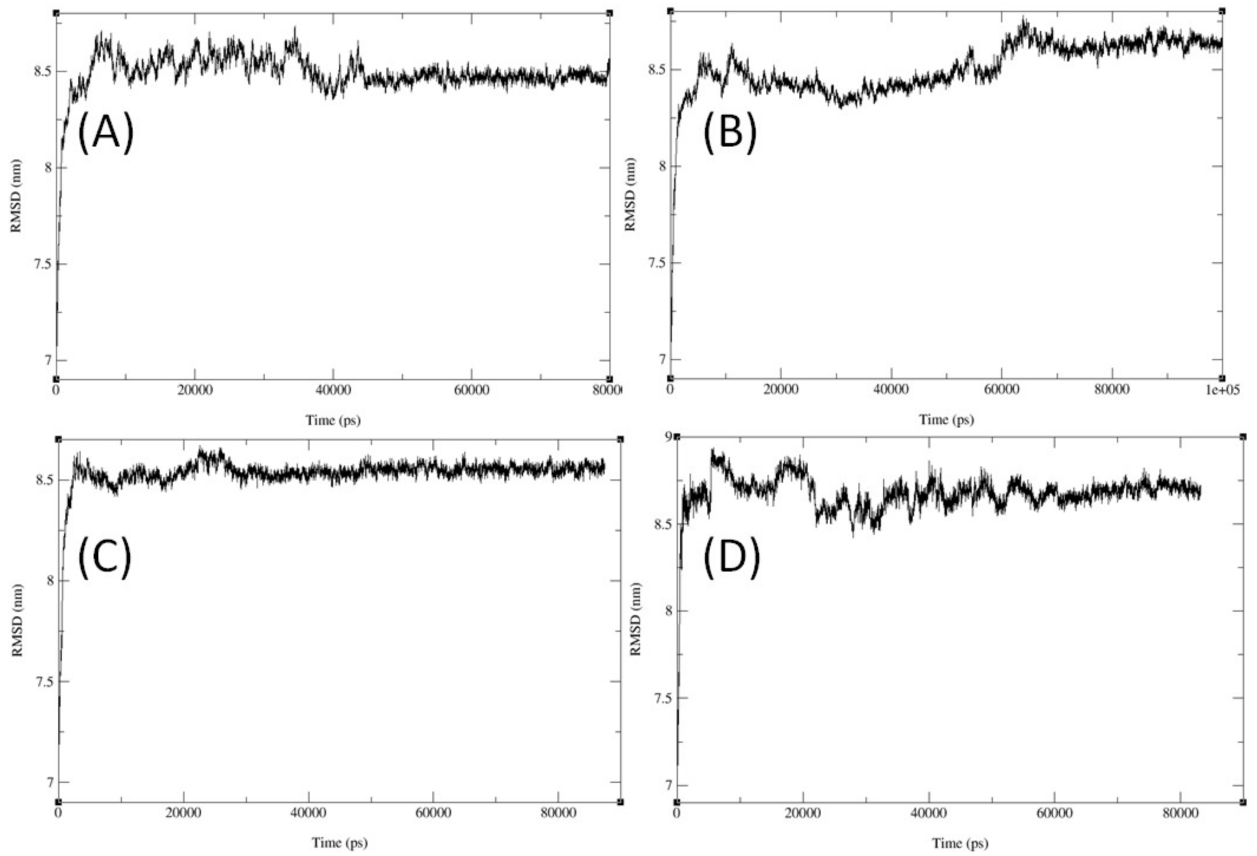

Figure S43. RMSD for (**A**) compound **13**; (**B**) compound **16**; and (**C**) compound **17**; (**D**) reference drug gemcitabine during the 20 ns MD simulation.

Table S59. RMSD values for compounds **13**, **16**, **17** and reference drug gemcitabine.

Compound	Compound RMSD (nm)
Compound **13**	7.07-8.75
Compound **16**	7.07-8.75
Compound **17**	7.2-8.65
Gemcitabine	7.1-8.9

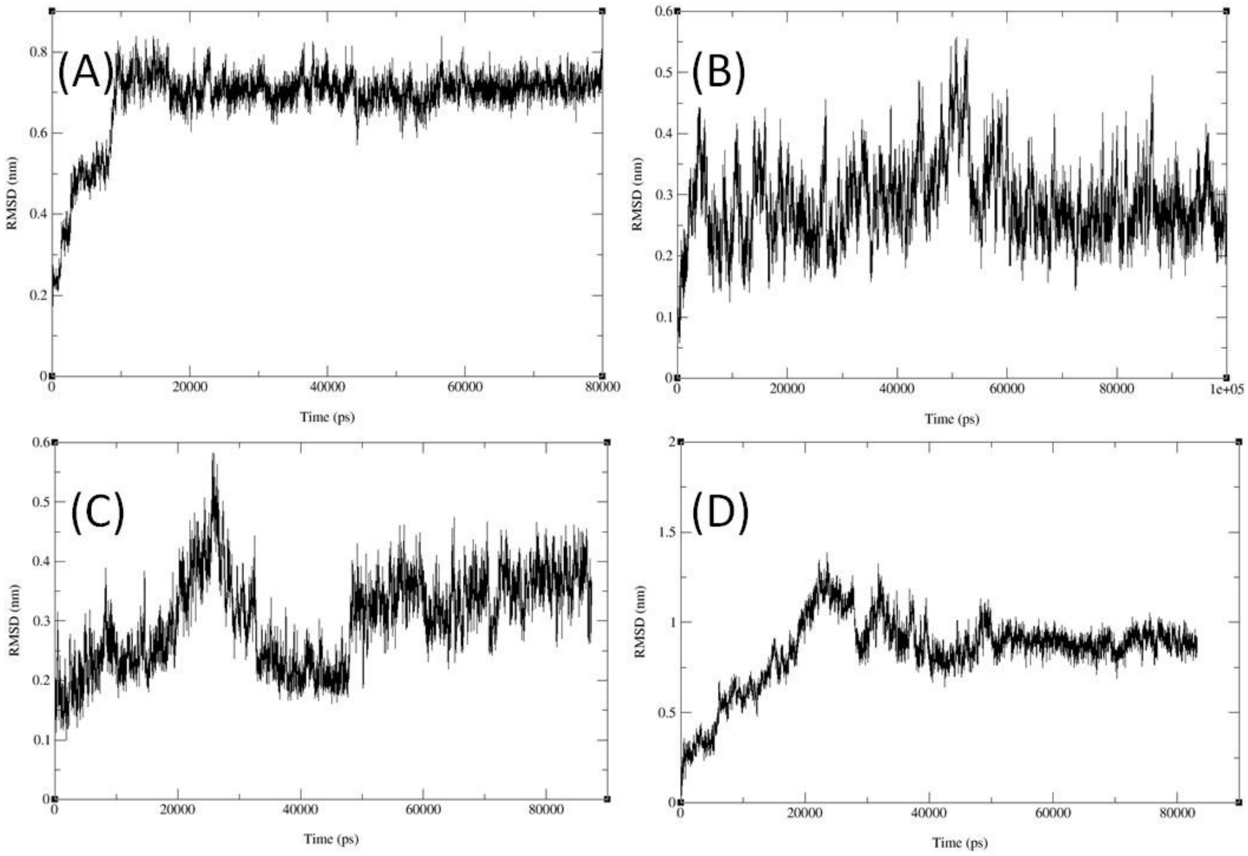

Figure S44. RMSD for protein LSD1-compound complex for (**A**) LSD1-compound **13**; (**B**) LSD1-compound **16**; and (**C**) LSD1-compound **17**; (**D**) LSD1-reference drug gemcitabine during the 20 ns MD simulation.

Table S60. RMSD values for compounds **13**, **16**, **17** and reference drug gemcitabine complexed with LSD1 (2Z3Y).

Compound	2Z3Y-Compound RMSD (nm)
Compound **13**	0.2-0.8
Compound **16**	0.05-0.55
Compound **17**	0.10 - 0.59
Gemcitabine	0.10-1.35

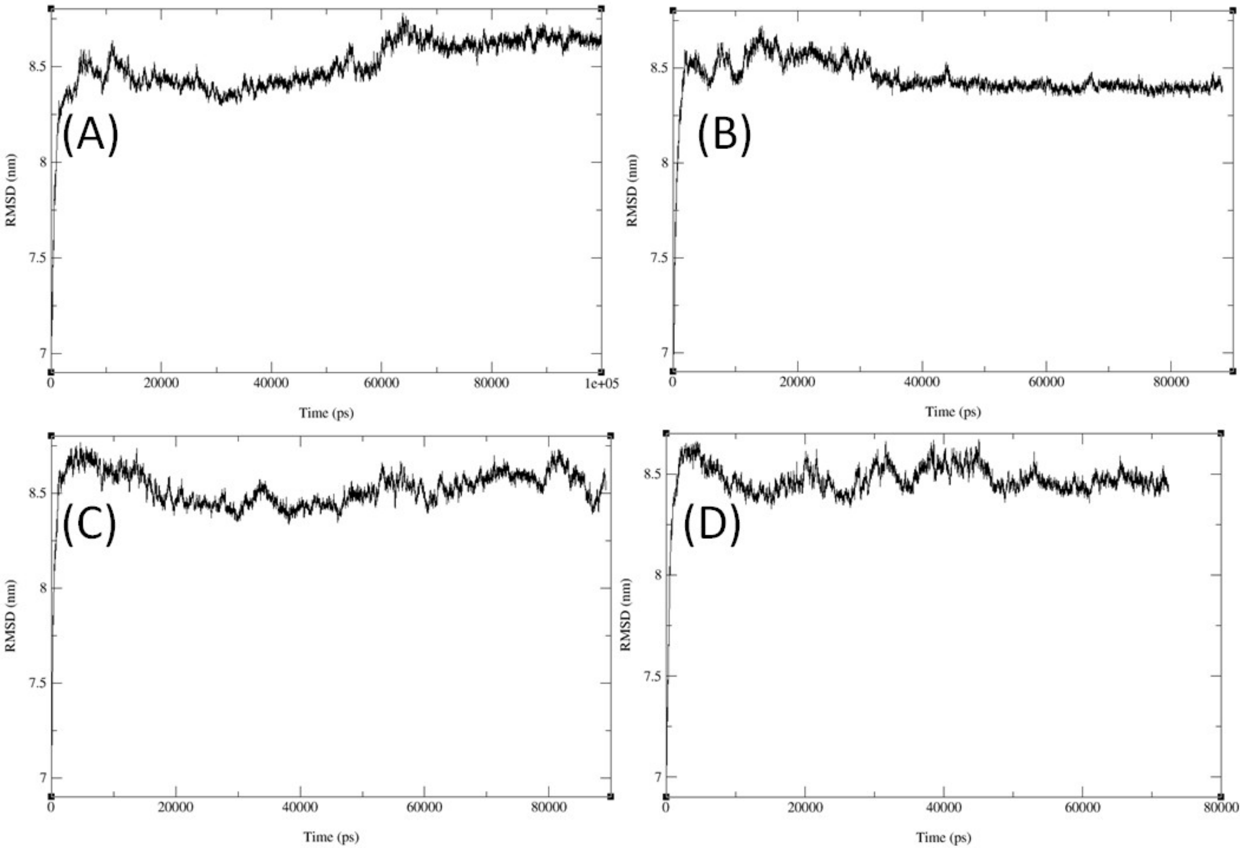

Figure S45. RMSD evolution for compound **16** (**A**) at 300 K; (**B**) at 305 K; (**C**) at 310 K; and (**D**) at 320 K; during the 20 ns MD simulation.

Table S61. RMSD values for compound **16** at 300, 305, 310, and 320K.

Temperature (K)	Compound **16** RMSD (nm)
300	7.1 - 9.0
305	7.0 - 9.0
310	7.2 - 9.0
320	7.05 - 8.9

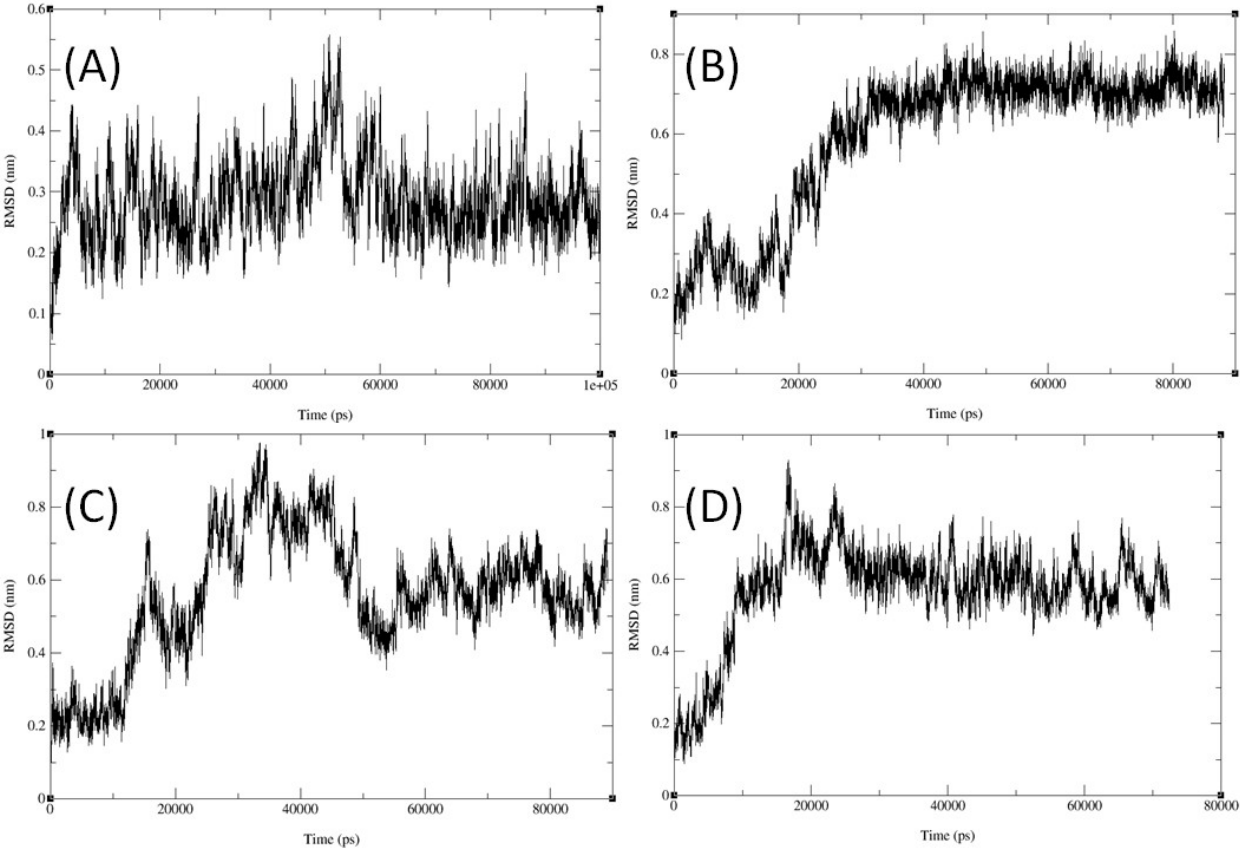

Figure S46. RMSD evolution for LSD1 protein-compound **16** (**A**) at 300 K; (**B**) at 305 K; (**C**) at 310 K; and (**D**) at 320 K; during the 20 ns MD simulation.

Table S62. RMSD values for compound **16** at 300, 305, 310, and 320K in free from and complexed with LSD1 (2Z3Y).

Temperature (K)	2Z3Y-Compound **16** RMSD (nm)
300	0.05 - 0.55
305	0.10 - 0.84
310	0.10 - 0.99
320	0.10 - 0.90

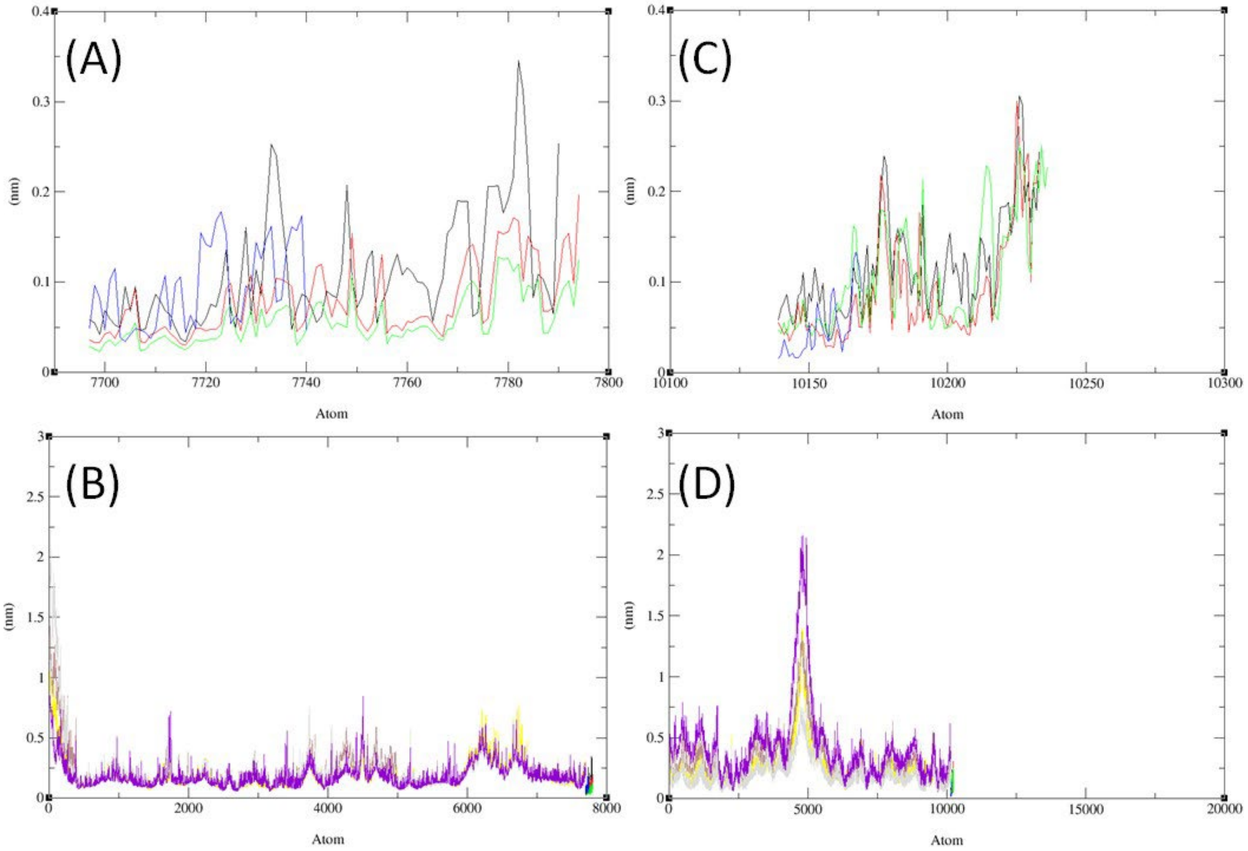

Figure S47. Progression of RMSF for (**A**) Compounds **12**, **14, 15**, and reference drug fluconazole at 300 K; (**B**) Docked complex of the protein topoisomerase II (PDB: 5CDQ) with compounds **12**, **14, 15**, and reference drugs fluconazole at 300 K; (**C**) Compounds **13**, **16, 17**, and reference drug fluconazole at 300 K; (**D**) Docked complex of the protein LSD1 (PDB: 2Z3Y) with compounds **13**, **16, 17**, and reference drugs gemcitabine at 300 K.

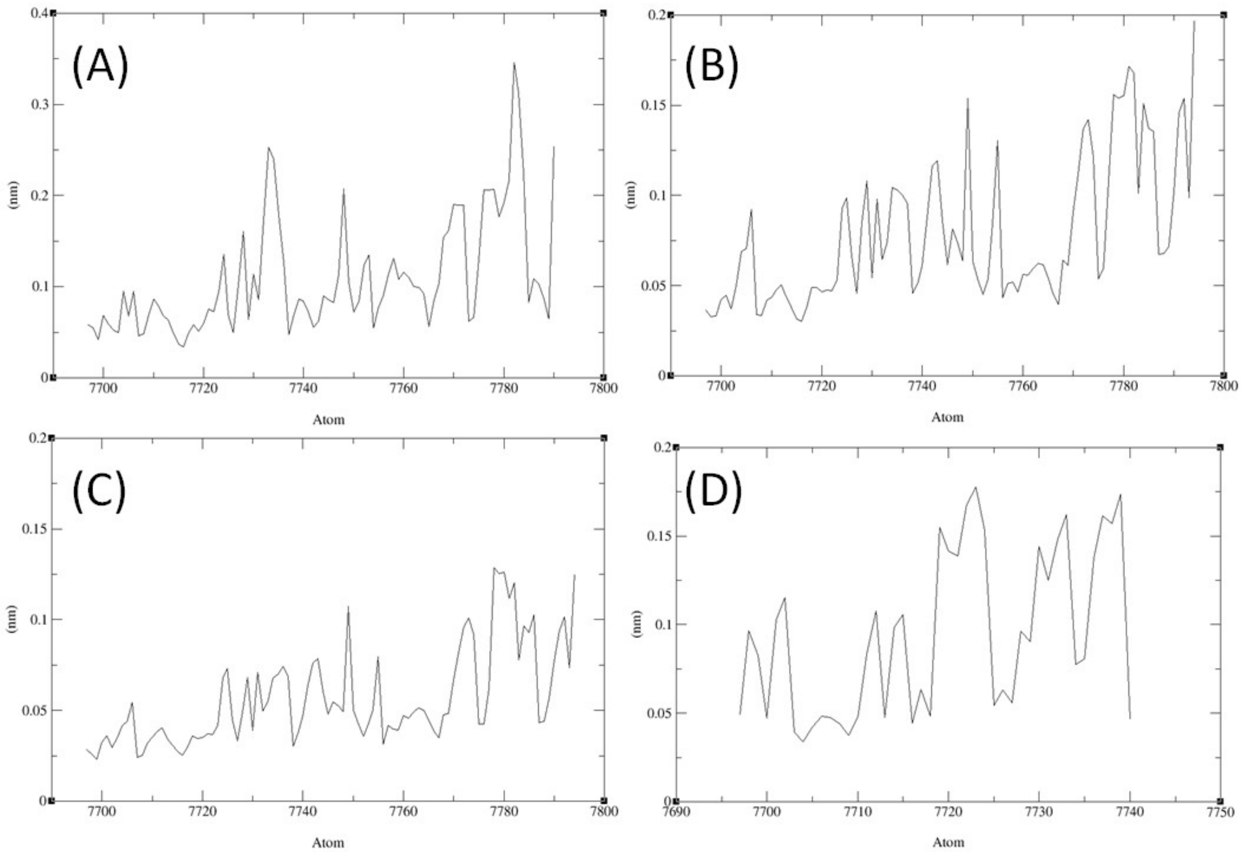

Figure S48. RMSF for (**A**) compound **12**; (**B**) compound **14**; and (**C**) compound **15**; (**D**) reference drug fluconazole during the 20 ns MD simulation.

Table S63. RMSF values for compounds **12**, **14**, **15**, and reference drug fluconazole at different residue position.

Residue Position	Compound **12** (nm)	Compound **14** (nm)	Compound **15** (nm)	Fluconazole (nm)
7700	0.05	0.0375	0.025	0.05
7720	0.06	0.0500	0.0375	0.14
7740	0.08	0.0500	0.0300	0.05
7760	0.10	0.0500	0.0500	-
7780	0.15	0.1000	0.1500	-

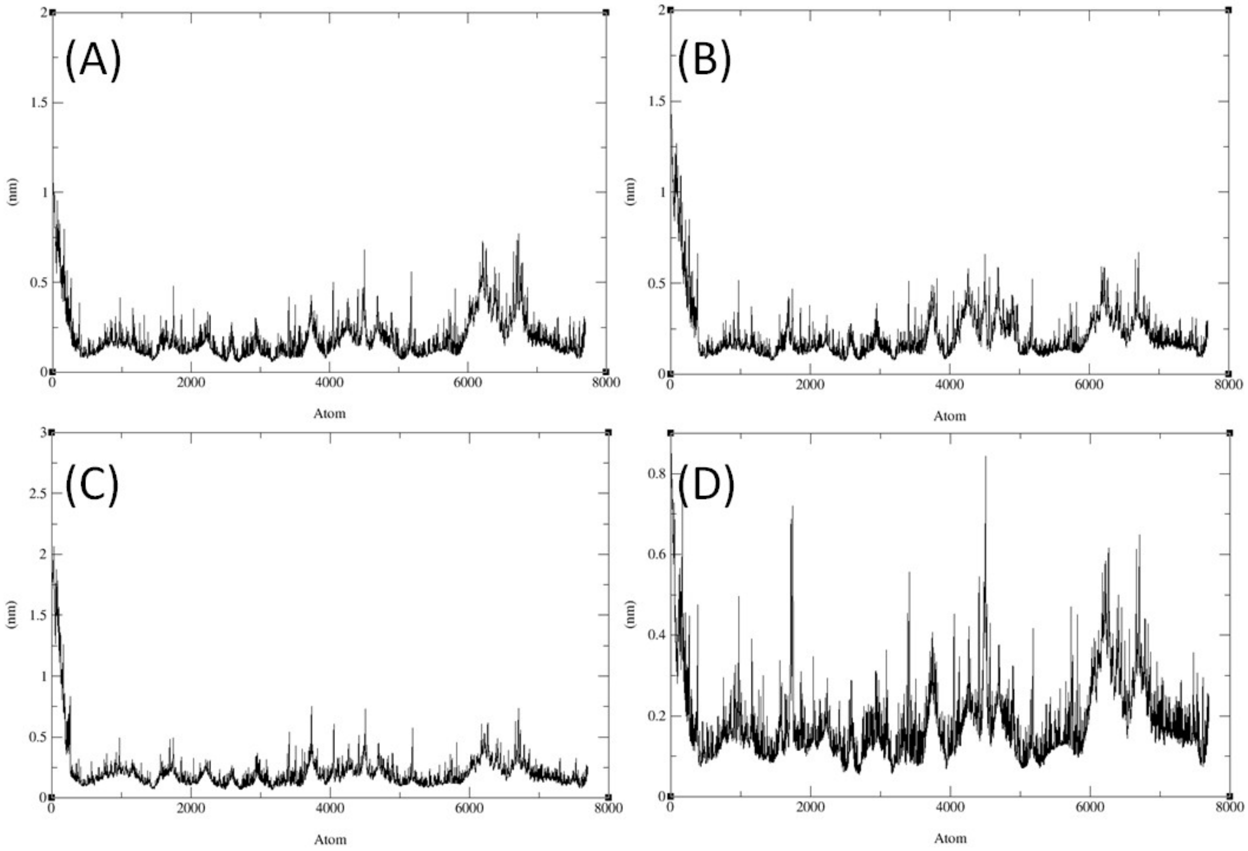

Figure S49. RMSF for protein Topoisomerase II-compound complex for (**A**) Topoisomerase II-compound **12**; (**B**) Topoisomerase II-compound **14**; and (**C**) Topoisomerase II-compound **15**; (**D**) Topoisomerase II-reference drug fluconazole during the 20 ns MD simulation.

Table S64. RMSF values for compounds **12**, **14**, **15**, and reference drug fluconazole complexed with topoisomerase II (5CDQ) at different residue position.

Residue Position	Topo II-Compound **12** (nm)	Topo II-Compound **14** (nm)	Topo II-Compound **15** (nm)	Topo II-Fluconazole (nm)
2000	0.25	0.3	0.25	0.2
4000	0.25	0.3	0.25	0.2
6000	0.25	0.25	0.25	0.2
7800	0.26	0.26	0.25	0.25

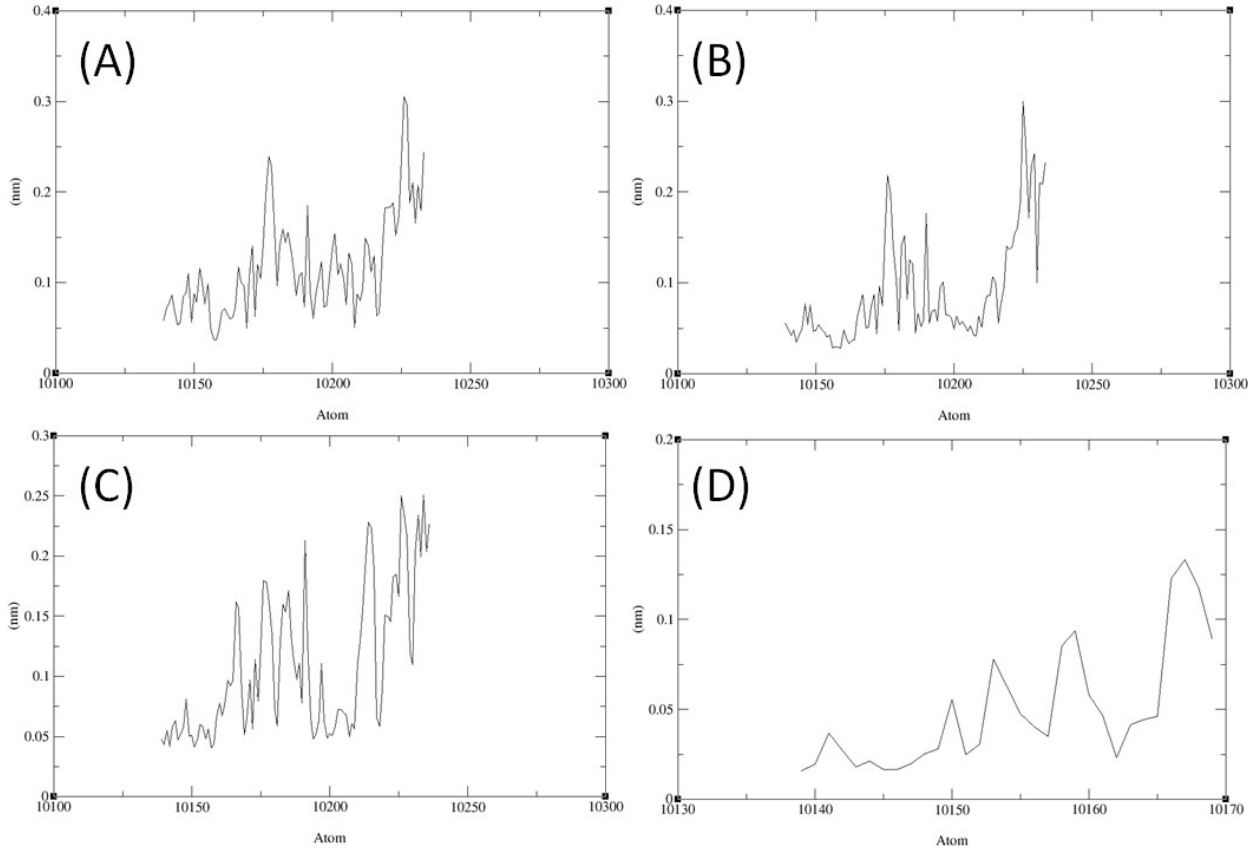

Figure S50. RMSF for (**A**) compound **13**; (**B**) compound **16**; and (**C**) compound **17**; (**D**) reference drug gemcitabine during the 20 ns MD simulation.

Table S65. RMSF values for compounds **13**, **16**, **17**, and reference drug gemcitabine at different residue position.

Residue Position	Compound **13** (nm)	Compound **16** (nm)	Compound **17** (nm)	Gemcitabine (nm)
10140	0.06	0.05	0.0400	0.025
10150	0.12	0.08	0.0500	0.054
10160	0.07	0.05	0.0750	0.060
10175	0.15	0.10	0.0175	-
10200	0.10	0.06	0.0590	-
10225	0.20	0.15	0.1500	-

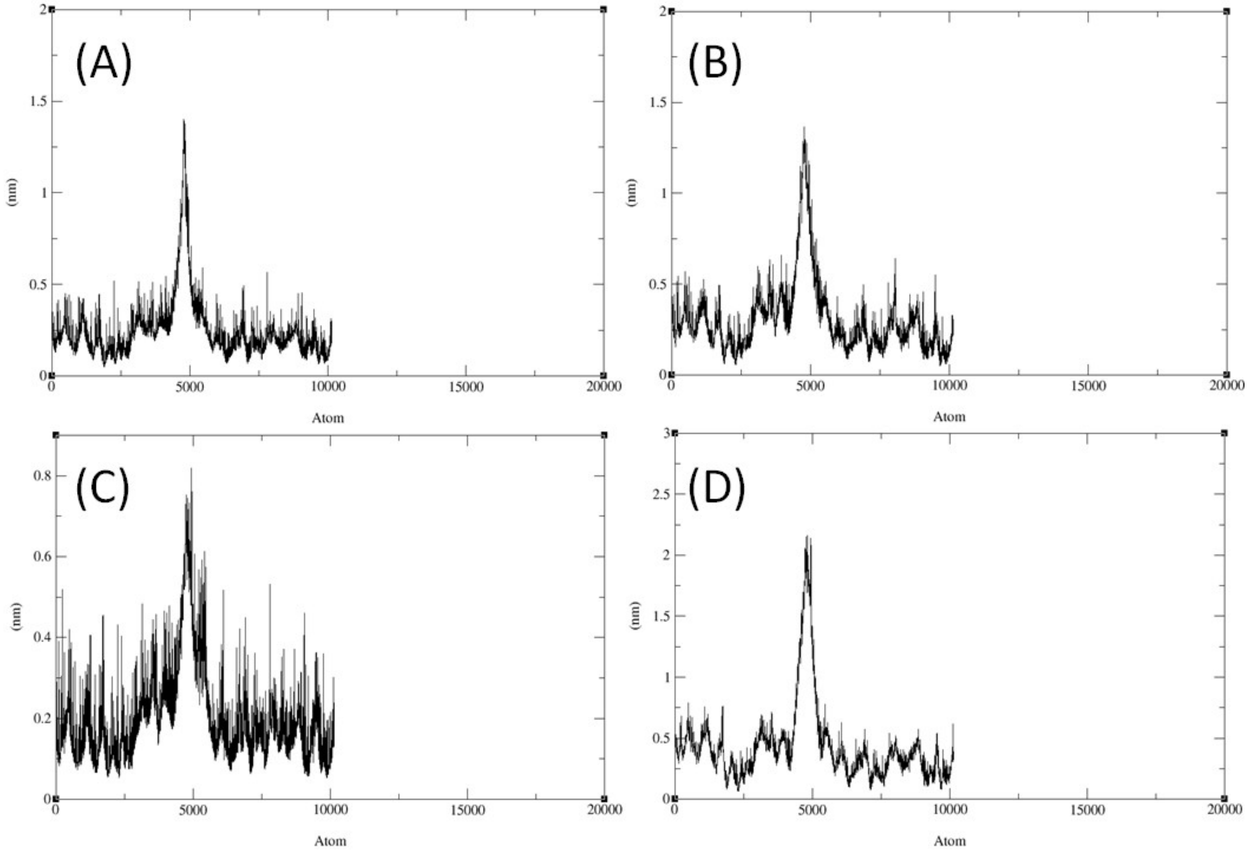

Figure S51. RMSF for protein LSD1-compound complex for (**A**) LSD1-compound **13**; (**B**) LSD1-compound **16**; and (**C**) LSD1-compound **17**; (**D**) LSD1-reference drug gemcitabine during the 20 ns MD simulation.

Table S66. RMSF values for compounds **13**, **16**, **17**, and reference drug gemcitabine complexed with LSD1 (2Z3Y) at different residue position.

Residue Position	LSD1-Compound **13** (nm)	LSD1-Compound **16** (nm)	LSD1-Compound **17** (nm)	LSD1-Gemcitabine (nm)
2500	0.25	0.10	0.08	0.10
5000	0.50	0.75	0.35	1.00
7500	0.20	0.20	0.10	0.25
10000	0.10	0.10	0.09	0.25

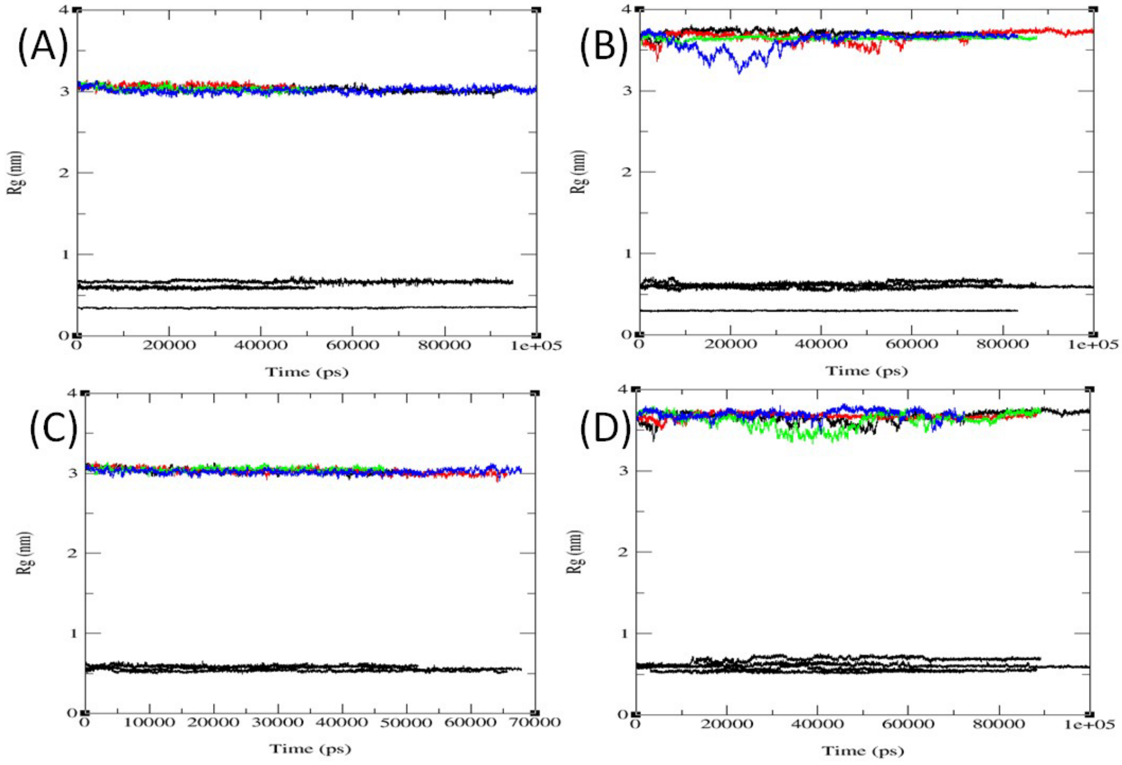

Figure S52. Progression of Rg for (A) Compounds 12, 14, 15, and reference drug fluconazole at 300 K and docked complex of the protein Topoisomerase II (PDB: 5CDQ) with compounds 12, 14, 15, and reference drugs fluconazole at 300 K; (B) Compounds 13, 16, 17, and reference drug gemcitabine at 300 K and docked complex of the protein LSD1 (PDB: 2Z3Y) with compounds 13, 16, 17, and reference drugs gemcitabine at 300 K; (C) Compound 15 at 300, 305, 310, and 320 K; and target protein Topoisomerase II with compound 15 at 300, 305, 310, and 320 K; (D) Compound 16 at 300, 305, 310, and 320 K; and target protein LSD1 with compound 16 at 300, 305, 310, and 320 K.

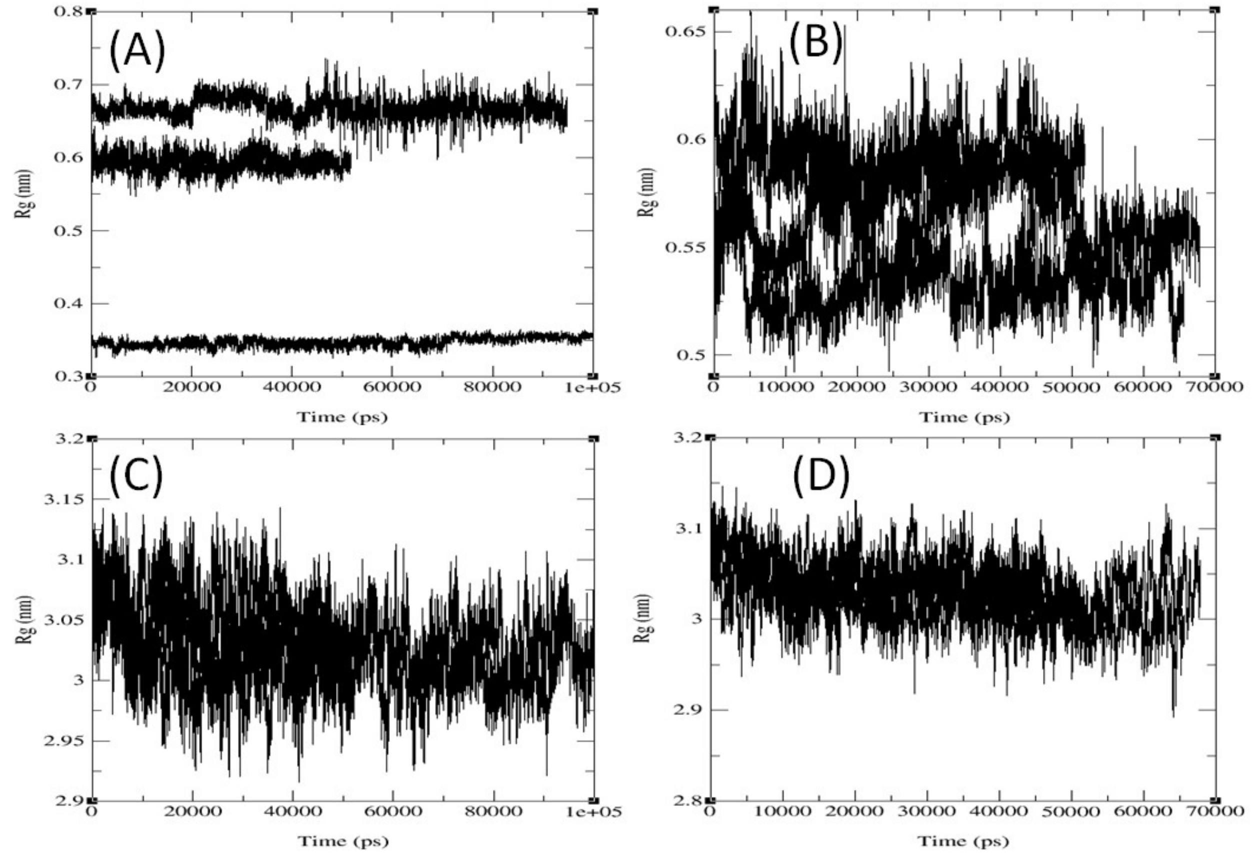

Figure S53. Progression of Rg for (**A**) Compounds **12**, **14, 15**, and reference drug fluconazole at 300 K; (**B**) Docked complex of the protein Topoisomerase II (PDB: 5CDQ) with compounds **12**, **14, 15**, and reference drug fluconazole at 300 K; (**C**) Compound **15** at 300, 305, 310, and 320 K; and (**D**) Target protein Topoisomerase II with compound **15** at 300, 305, 310, and 320 K.

Table S67. Rg values for compounds **12**, **14**, **15** and reference drug fluconazole in free form and complexed with topoisomerase II (5CDQ).

Compound	Compound RMSD (nm)	5CDQ-Compound RMSD (nm)
Compound **12**	0.60 - 0.759	2.96 - 3.13
Compound **14**	0.55 - 0.641	2.975 - 3.15
Compound **15**	0.55 - 0.63	2.92 - 3.126
Fluconazole	0.325 - 0.365	2.925 - 3.128

Table S68. Rg values for compound **15** at 300, 305, 310, and 320K in free from and complexed with topoisomerase II (5CDQ).

Temperature (K)	Compound **15** RMSD (nm)	5CDQ-Compound **15** RMSD (nm)
300	0.55 - 0.63	2.925 - 3.125
305	0.49 - 0.585	2.90 - 3.15
310	0.53 - 0.66	2.95 - 3.15
320	0.49 - 0.65	2.927 - 3.125

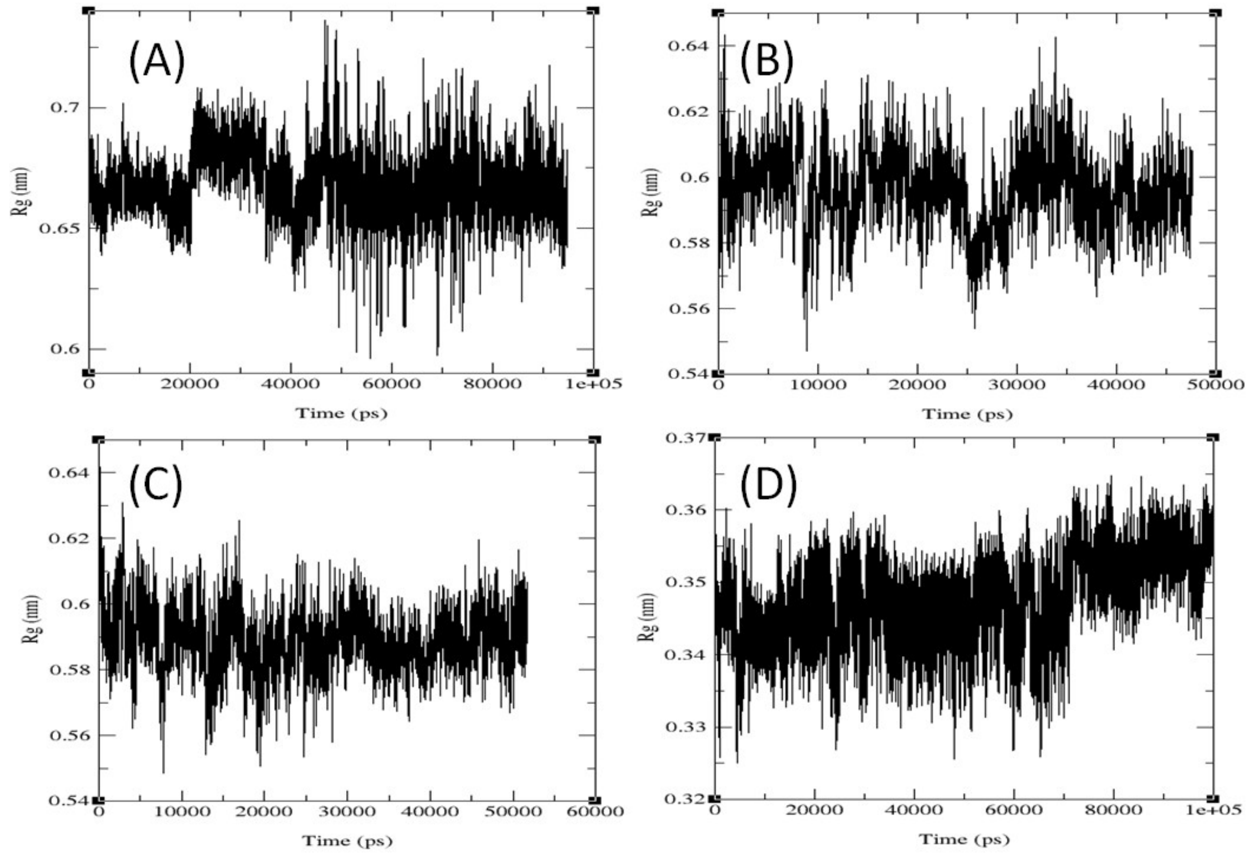

Figure S54. Rg for (**A**) compound **12**; (**B**) compound **14**; and (**C**) compound **15**; (**D**) reference drug fluconazole during the 20 ns MD simulation.

Table S69. Rg values for compounds **12**, **14**, **15** and reference drug fluconazole.

Compound	Compound RMSD (nm)
Compound **12**	0.60 - 0.759
Compound **14**	0.55 - 0.641
Compound **15**	0.55 - 0.63
Fluconazole	0.325 - 0.365

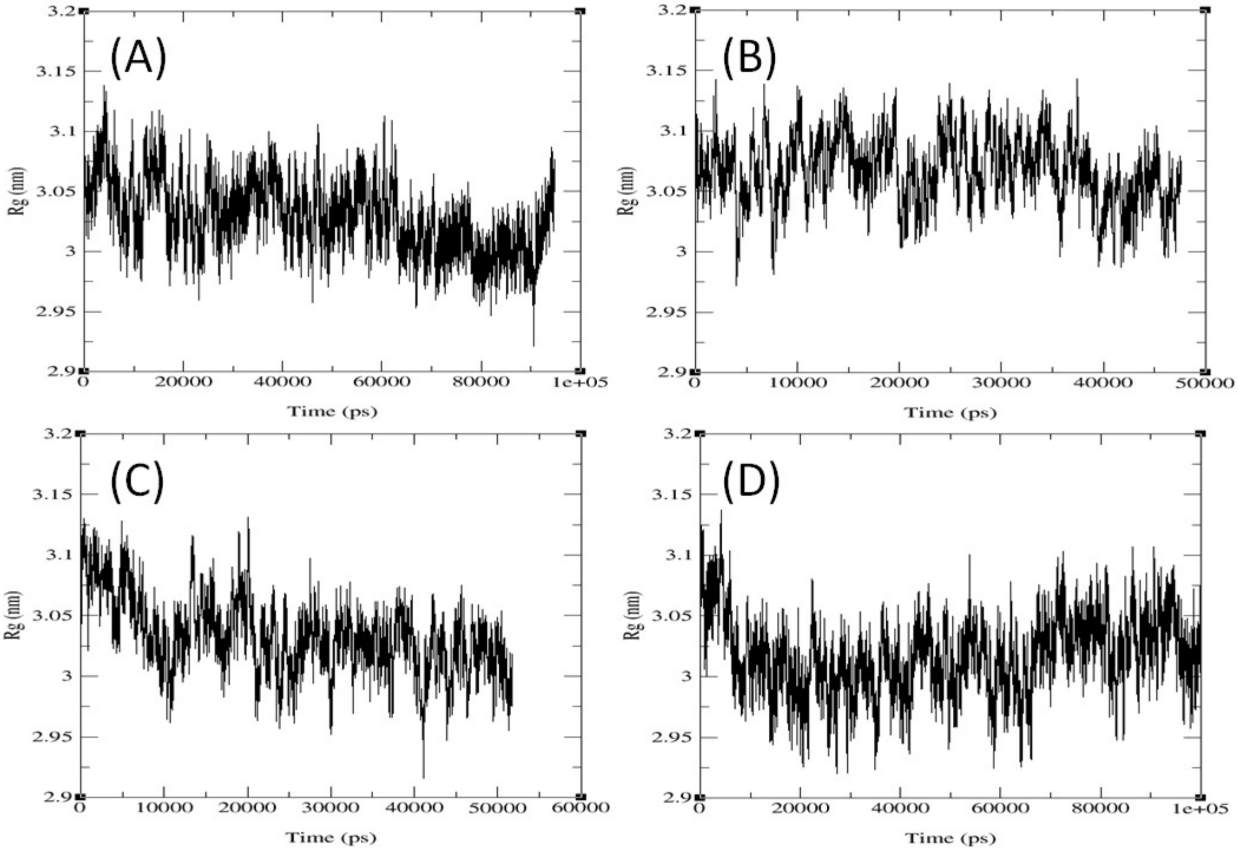

Figure S55. Rg for protein Topoisomerase II-compound complex for (**A**) Topoisomerase II-compound **12**; (**B**) Topoisomerase II-compound **14**; and (**C**) Topoisomerase II-compound **15**; (**D**) Topoisomerase II-reference drug fluconazole during the 20 ns MD simulation.

Table S70. Rg values for compounds **12**, **14**, **15** and reference drug fluconazole complexed with topoisomerase II (5CDQ).

Compound	5CDQ-Compound RMSD (nm)
Compound **12**	2.96 - 3.13
Compound **14**	2.975 - 3.15
Compound **15**	2.92 - 3.126
Fluconazole	2.925 - 3.128

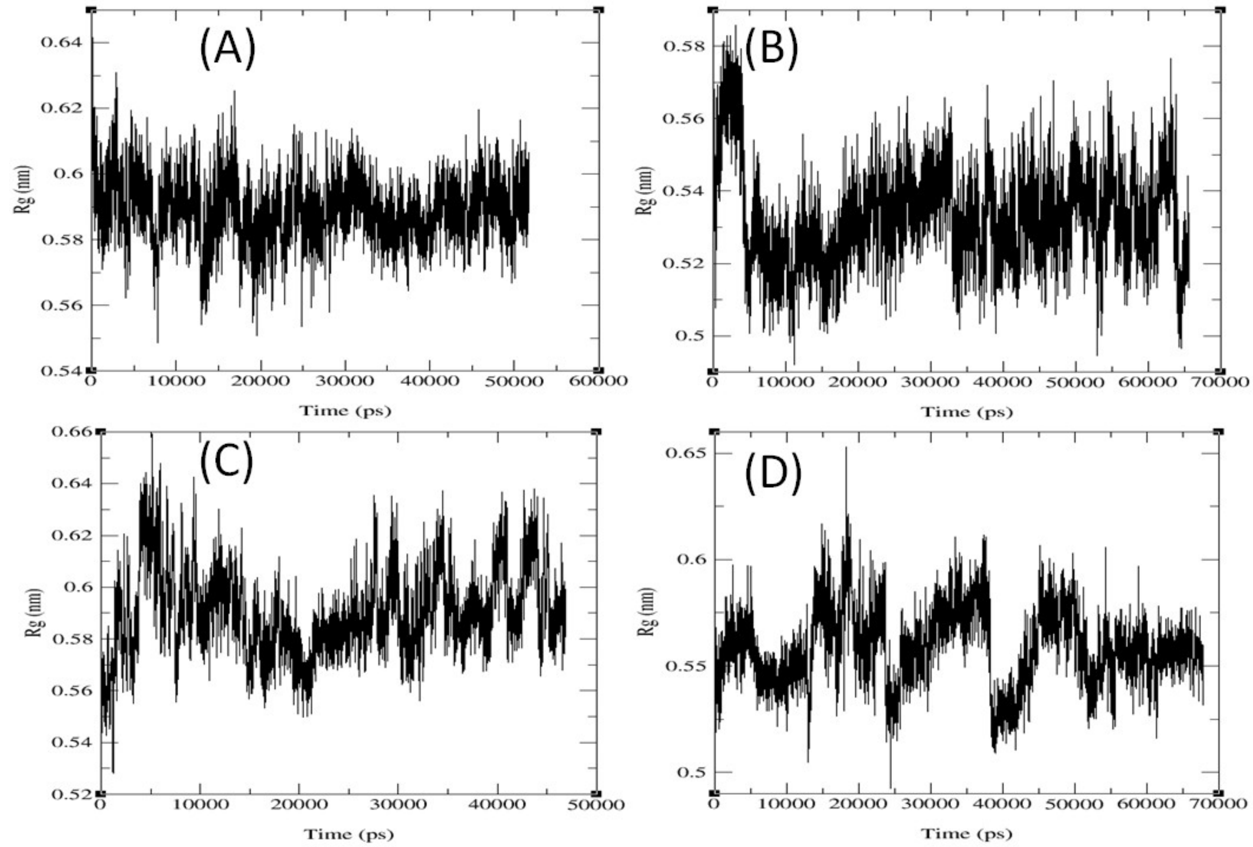

Figure S56. Rg evolution for compound **15** (**A**) at 300 K; (**B**) at 305 K; (**C**) at 310 K; and (**D**) at 320 K; during the 20 ns MD simulation.

Table S71. Rg values for compound **15** at 300, 305, 310, and 320K.

Temperature (K)	Compound **15** RMSD (nm)
300	0.55 - 0.63
305	0.49 - 0.585
310	0.53 - 0.66
320	0.49 - 0.65

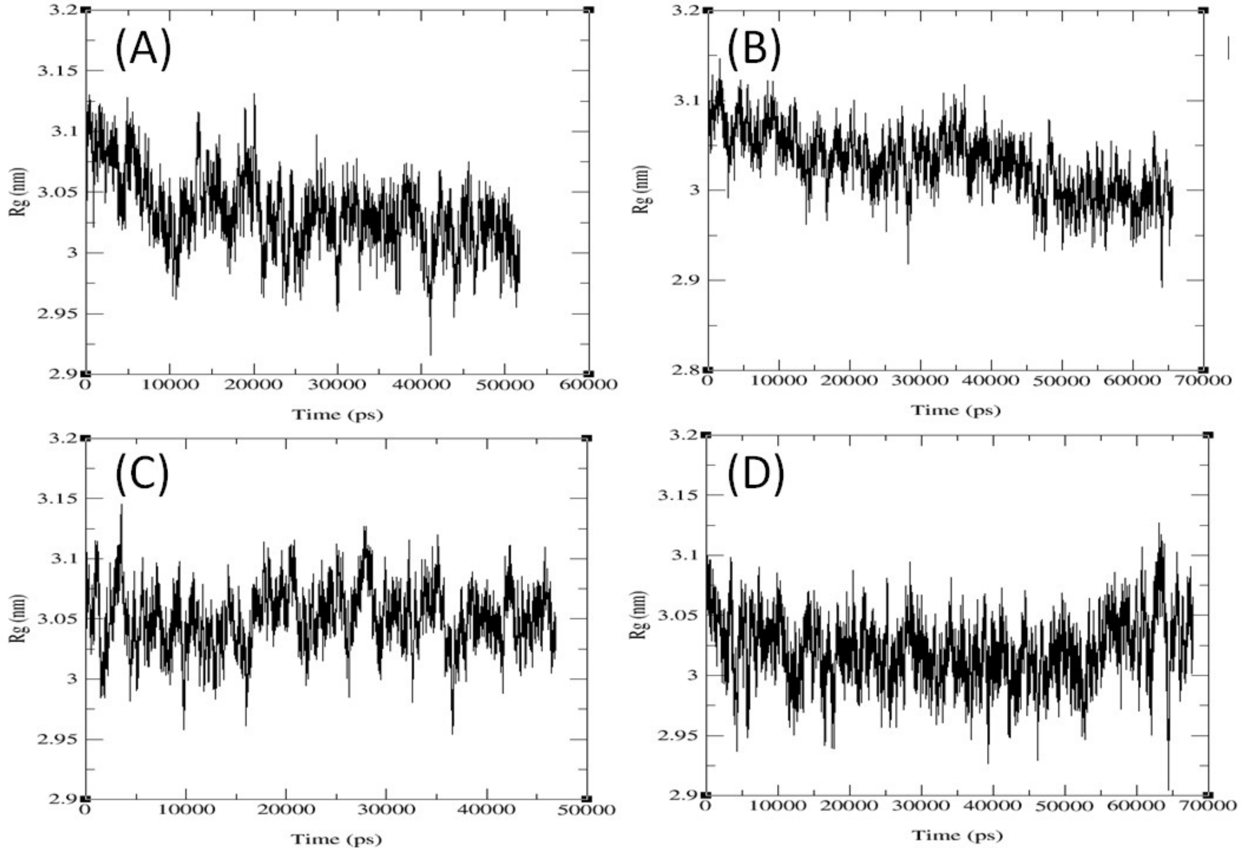

Figure S57. Rg evolution for Topoisomerase II protein-compound **15** (**A**) at 300 K; (**B**) at 305 K; (**C**) at 310 K; and (**D**) at 320 K; during the 20 ns MD simulation.

Table S72. Rg values for compound **15** at 300, 305, 310, and 320K complexed with topoisomerase II (5CDQ).

Temperature (K)	5CDQ-Compound **15** RMSD (nm)
300	2.925 - 3.125
305	2.90 - 3.15
310	2.95 - 3.15
320	2.927 - 3.125

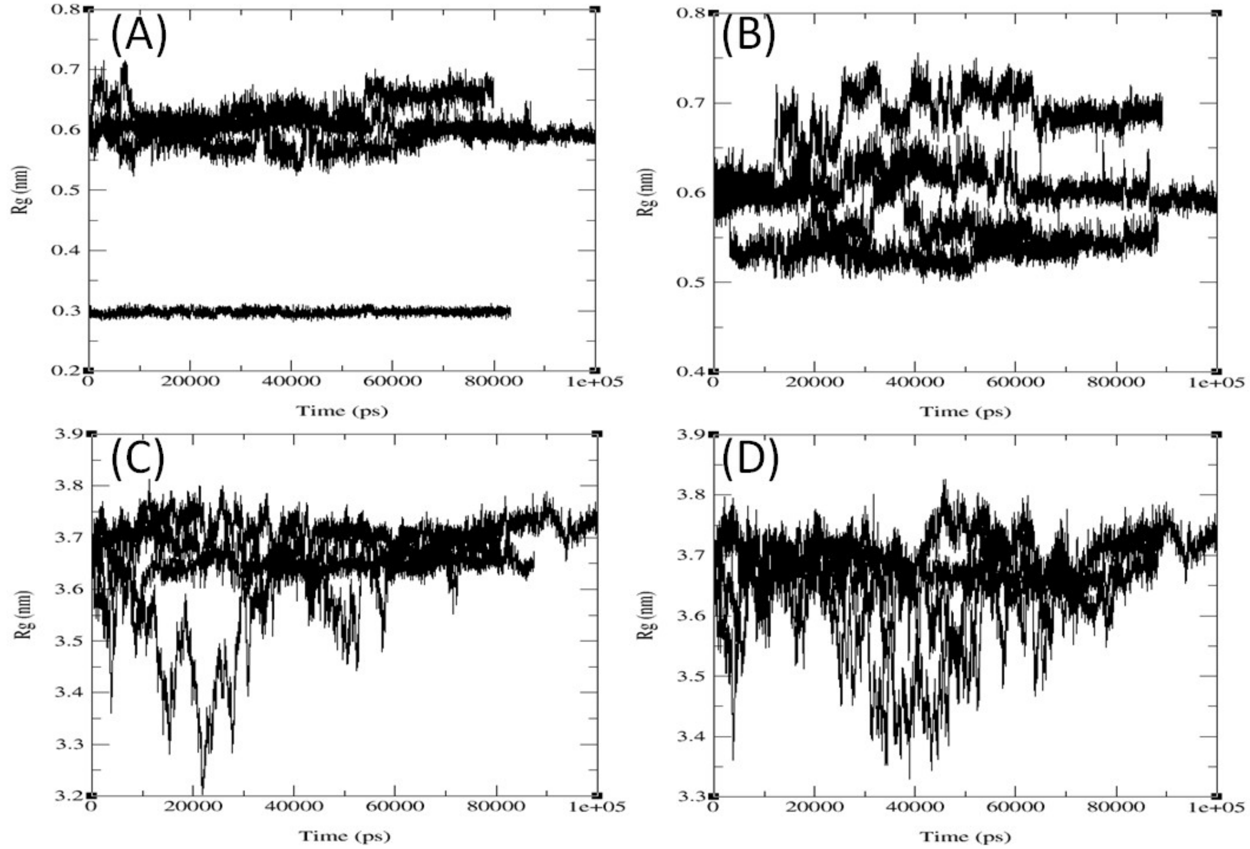

Figure S58. Progression of Rg for (**A**) Compounds **13**, **16, 17**, and reference drug gemcitabine at 300 K; (**B**) Docked complex of the protein LSD (PDB: 2Z3Y) with compounds **13**, **16, 17**, and reference drug gemcitabine at 300 K; (**C**) Compound **16** at 300, 305, 310, and 320 K; and (**D**) Target protein LSD1 with compound **16** at 300, 305, 310, and 320 K.

Table S73. Rg values for compounds **13**, **16**, **17** and reference drug gemcitabine in free form and complexed with LSD1 (2Z3Y).

Compound	Compound RMSD (nm)	2Z3Y-Compound RMSD (nm)
Compound **13**	0.58 - 0.72	3.625 - 3.815
Compound **16**	0.561 - 0.678	3.36 - 3.79
Compound **17**	0.525 - 0.7	3.55 - 3.725
Gemcitabine	0.281 - 0.314	3.21 - 3.756

Table S74. Rg values for compound **16** at 300, 305, 310, and 320K in free from and complexed with LSD1 (2Z3Y).

Temperature (K)	Compound **16** RMSD (nm)	2Z3Y-Compound **16** RMSD (nm)
300	3.35 - 3.79	3.36 - 3.79
305	3.5 - 3.75	3.5 - 3.7
310	3.325 - 3.79	3.32 - 3.79
320	0.5 - 0.64	3.45 - 3.825

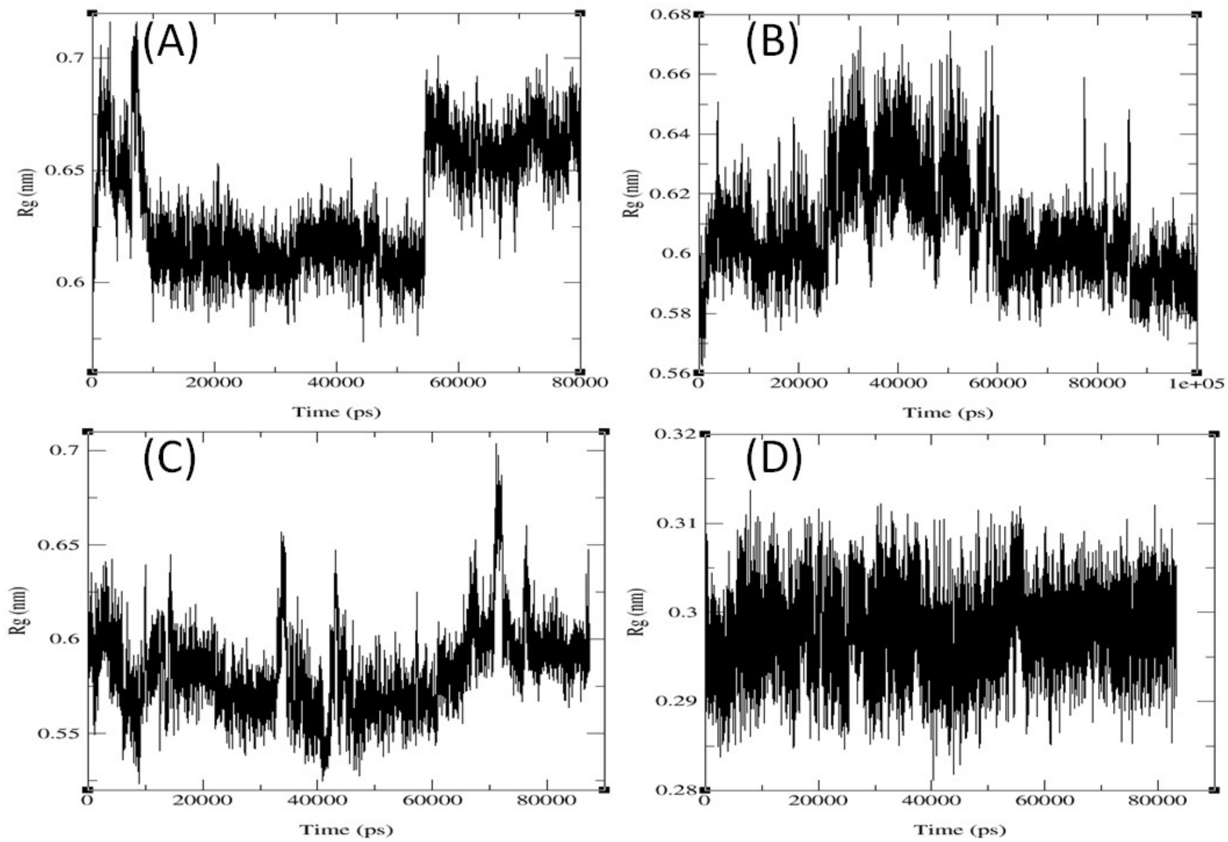

Figure S59. Rg for (**A**) compound **13**; (**B**) compound **16**; and (**C**) compound **17**; (**D**) reference drug gemcitabine during the 20 ns MD simulation.

Table S75. Rg values for compounds **13**, **16**, **17** and reference drug.

Compound	Compound RMSD (nm)
Compound **13**	0.58 - 0.72
Compound **16**	0.561 - 0.678
Compound **17**	0.525 - 0.7
Gemcitabine	0.281 - 0.314

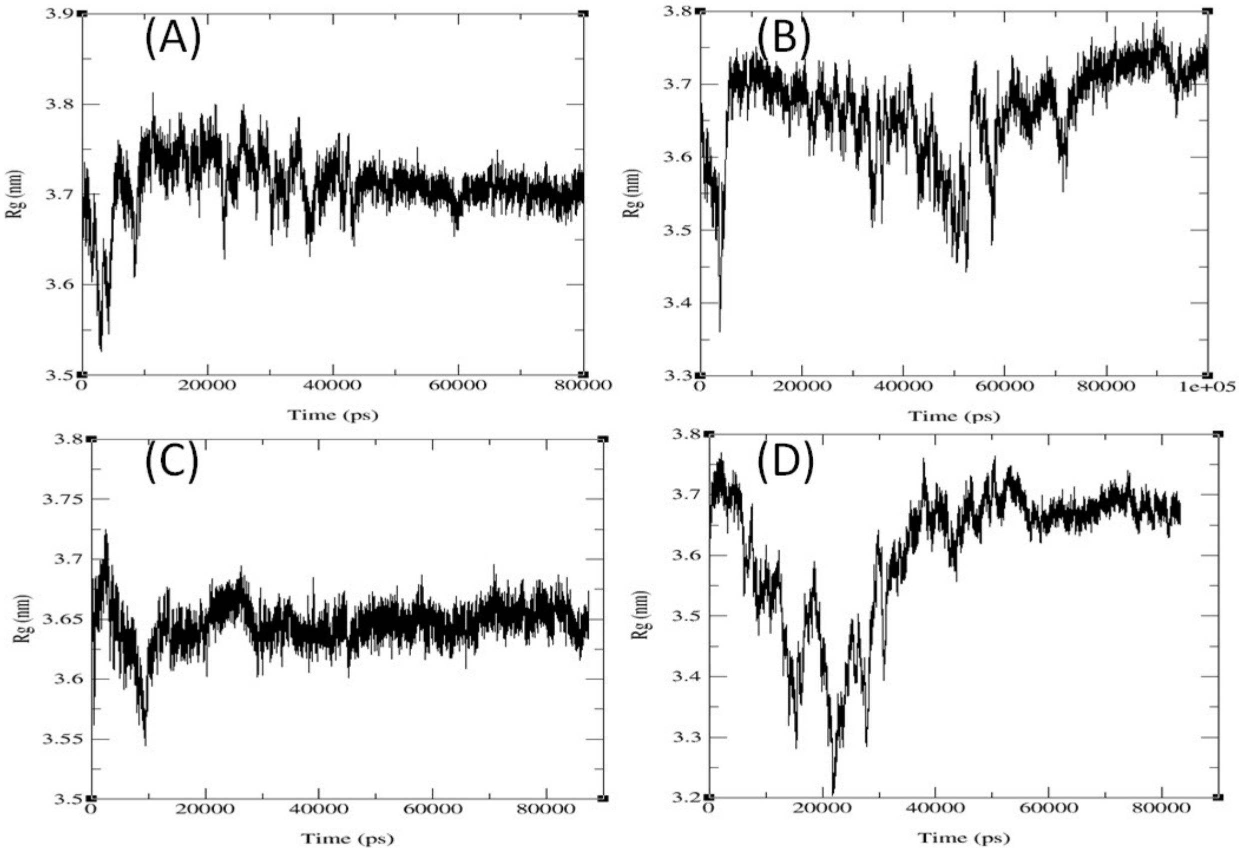

Figure S60. Rg for protein LSD1-compound complex for (**A**) LSD1-compound **13**; (**B**) LSD1-compound **16**; and (**C**) LSD1-compound **17**; (**D**) LSD1-reference drug gemcitabine during the 20 ns MD simulation.

Table S76. Rg values for compounds **13**, **16**, **17** and reference drug gemcitabine complexed with LSD1 (2Z3Y).

Compound	2Z3Y-Compound RMSD (nm)
Compound **13**	3.625 - 3.815
Compound **16**	3.36 - 3.79
Compound **17**	3.55 - 3.725
Gemcitabine	3.21 - 3.756

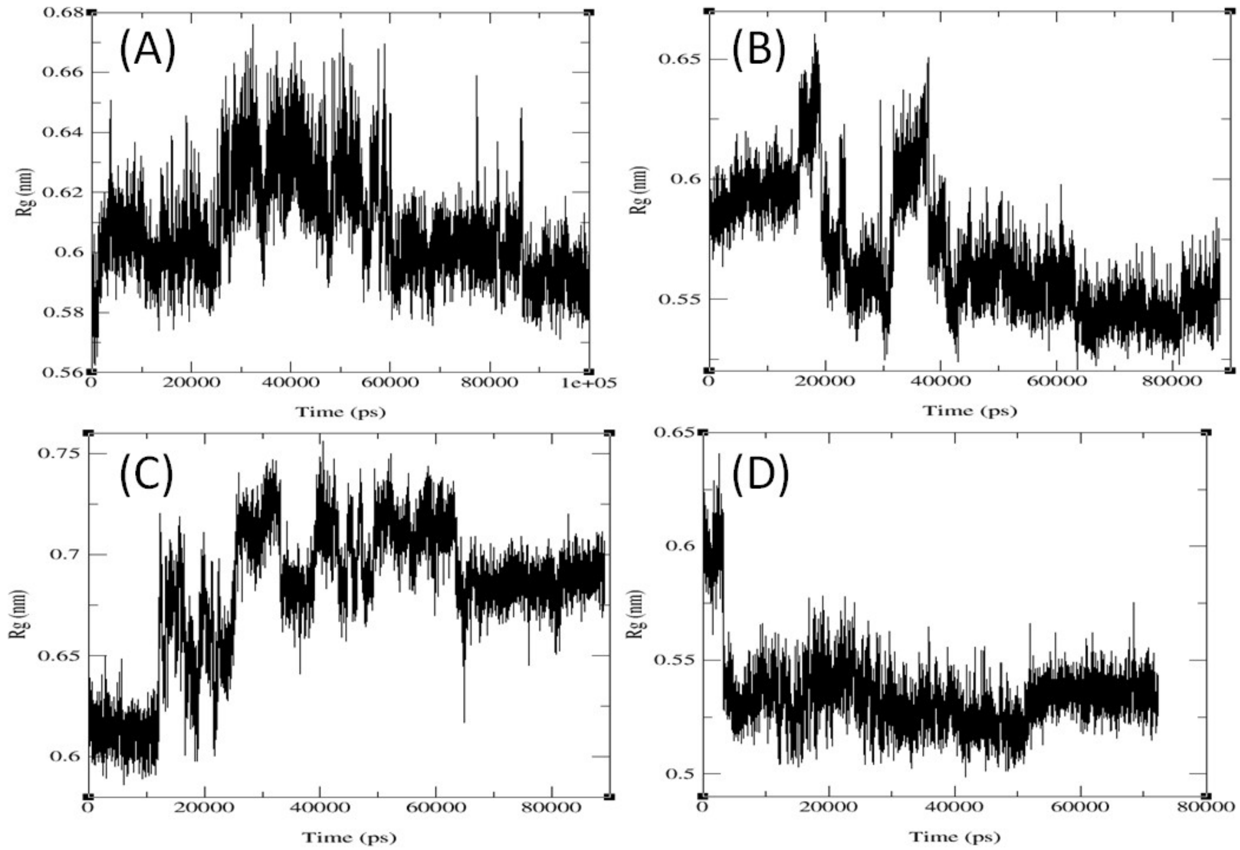

Figure S61. Rg evolution for compound **16** (**A**) at 300 K; (**B**) at 305 K; (**C**) at 310 K; and (**D**) at 320 K; during the 20 ns MD simulation.

Table S77. Rg values for compound **16** at 300, 305, 310, and 320K.

Temperature (K)	Compound **16** RMSD (nm)	2Z3Y-Compound **16** RMSD (nm)
300	3.35 - 3.79	3.36 - 3.79
305	3.5 - 3.75	3.5 - 3.7
310	3.325 - 3.79	3.32 - 3.79
320	0.5 - 0.64	3.45 - 3.825

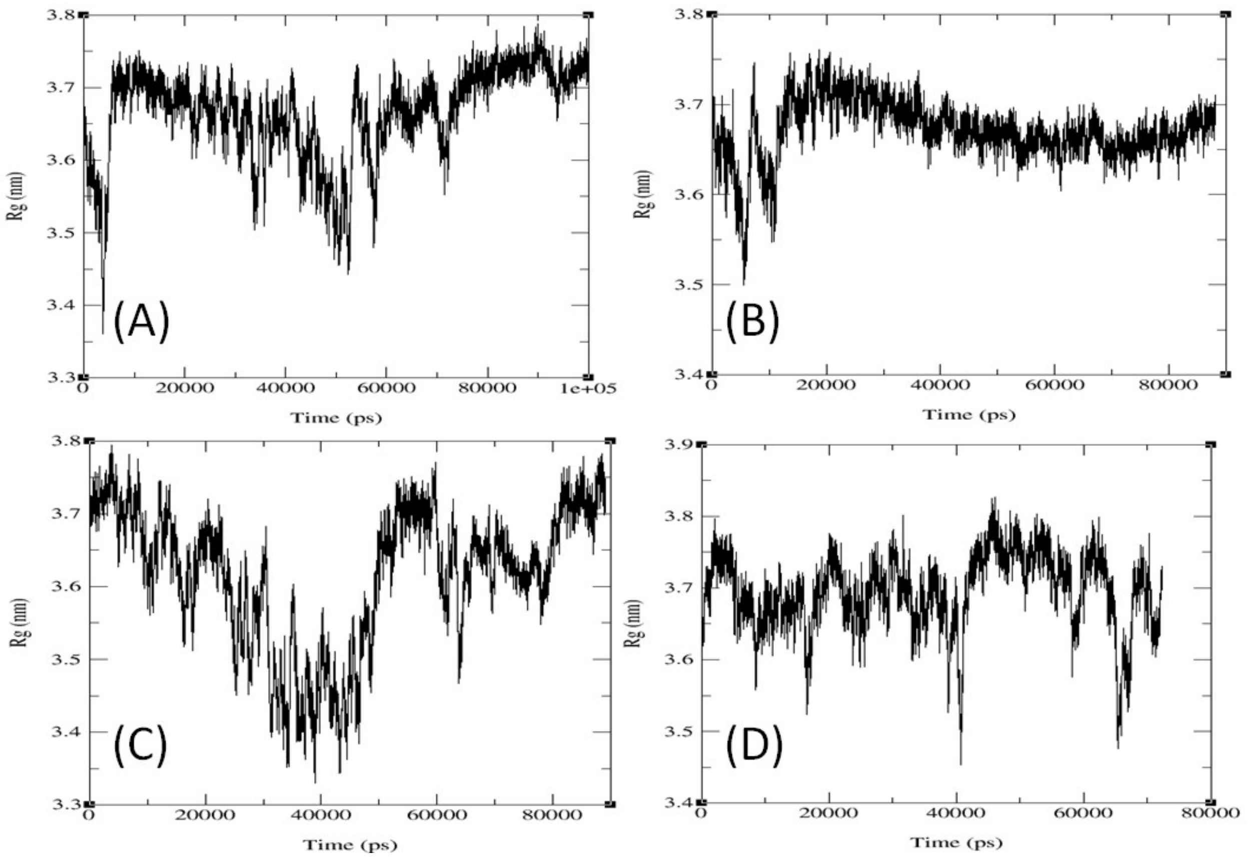

Figure S62. Rg evolution for LSD1 protein-compound **16** (**A**) at 300 K; (**B**) at 305 K; (**C**) at 310 K; and (**D**) at 320 K; during the 20 ns MD simulation.

Table S78. Rg values for compound **16** at 300, 305, 310, and 320K complexed with LSD1 (2Z3Y).

Temperature (K)	2Z3Y-Compound **16** RMSD (nm)
300	3.36 - 3.79
305	3.5 - 3.7
310	3.32 - 3.79
320	3.45 - 3.825

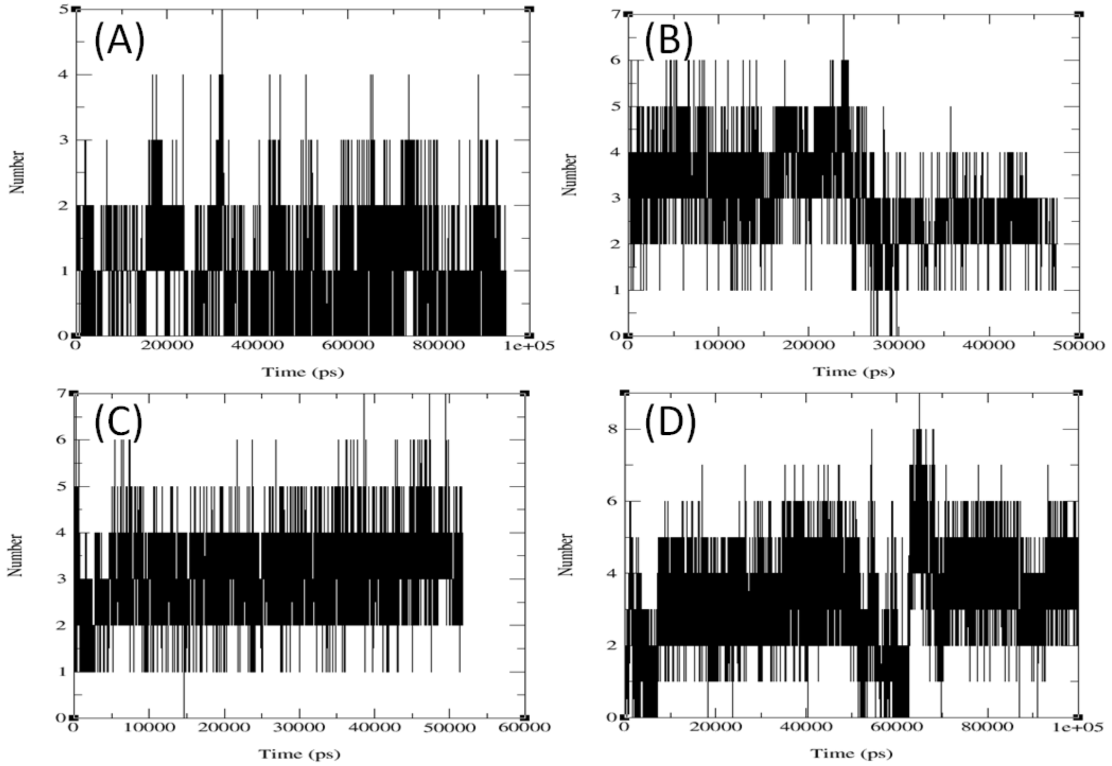

Figure S63. HB analysis for (**A**) compound **12**; (**B**) compound **14**; and (**C**) compound **15**; (**D**) reference drug fluconazole during the 20 ns MD simulation.

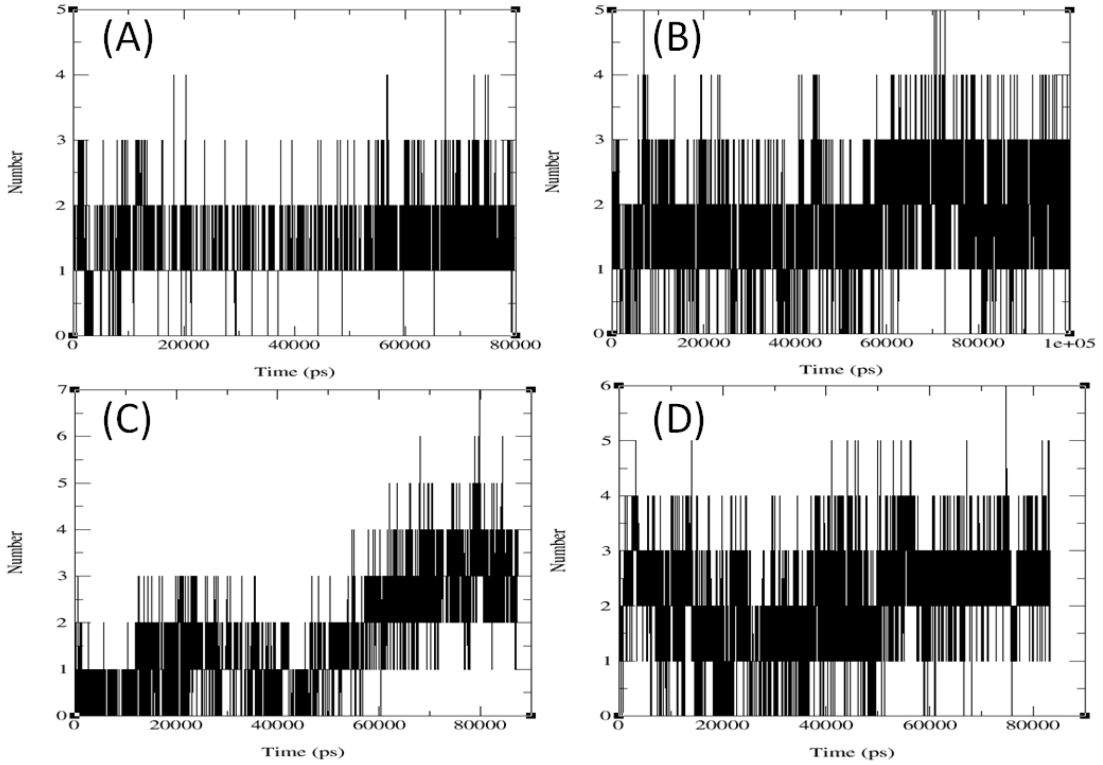

Figure S64. HB analysis for (**A**) compound **13**; (**B**) compound **16**; and (**C**) compound **17**; (**D**) reference drug gemcitabine during the 20 ns MD simulation.

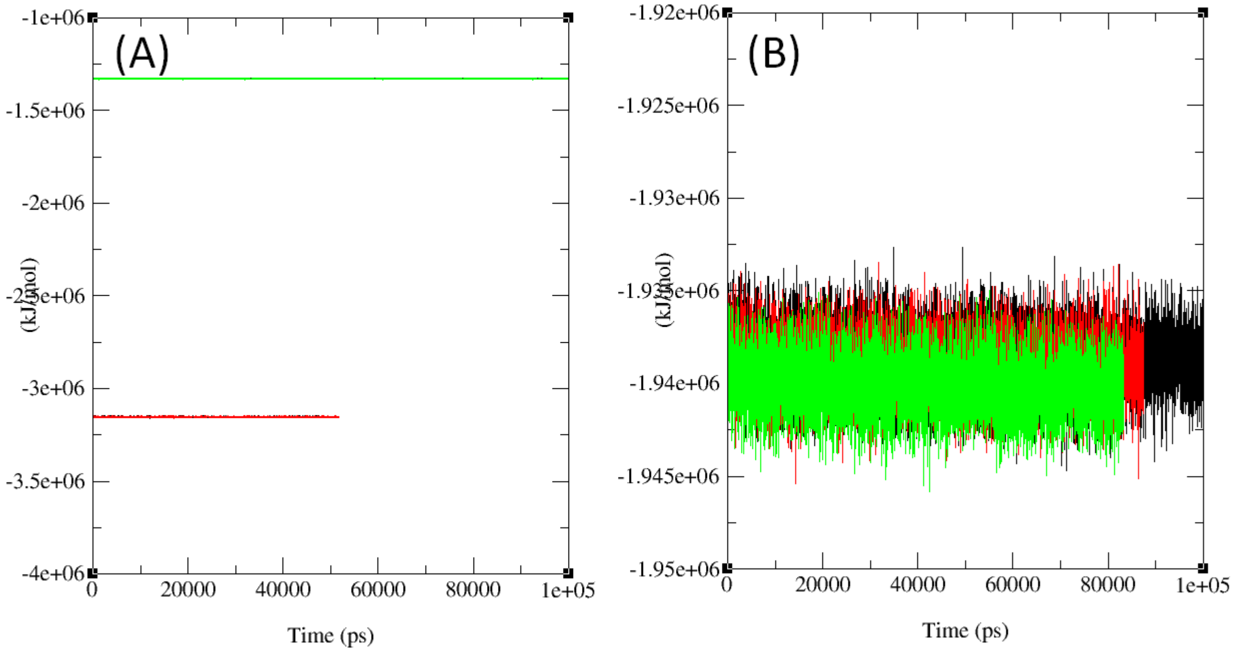

Figure S65. The potential energy curves over the course of the 100 ns MD simulation for (**A**) compounds 12, 14, 15, and reference drug fluconazole with topoisomerase II at 300K; and (**B**) compounds 13, 16, 17, and reference drug gemcitabine with LSD1 at 300K

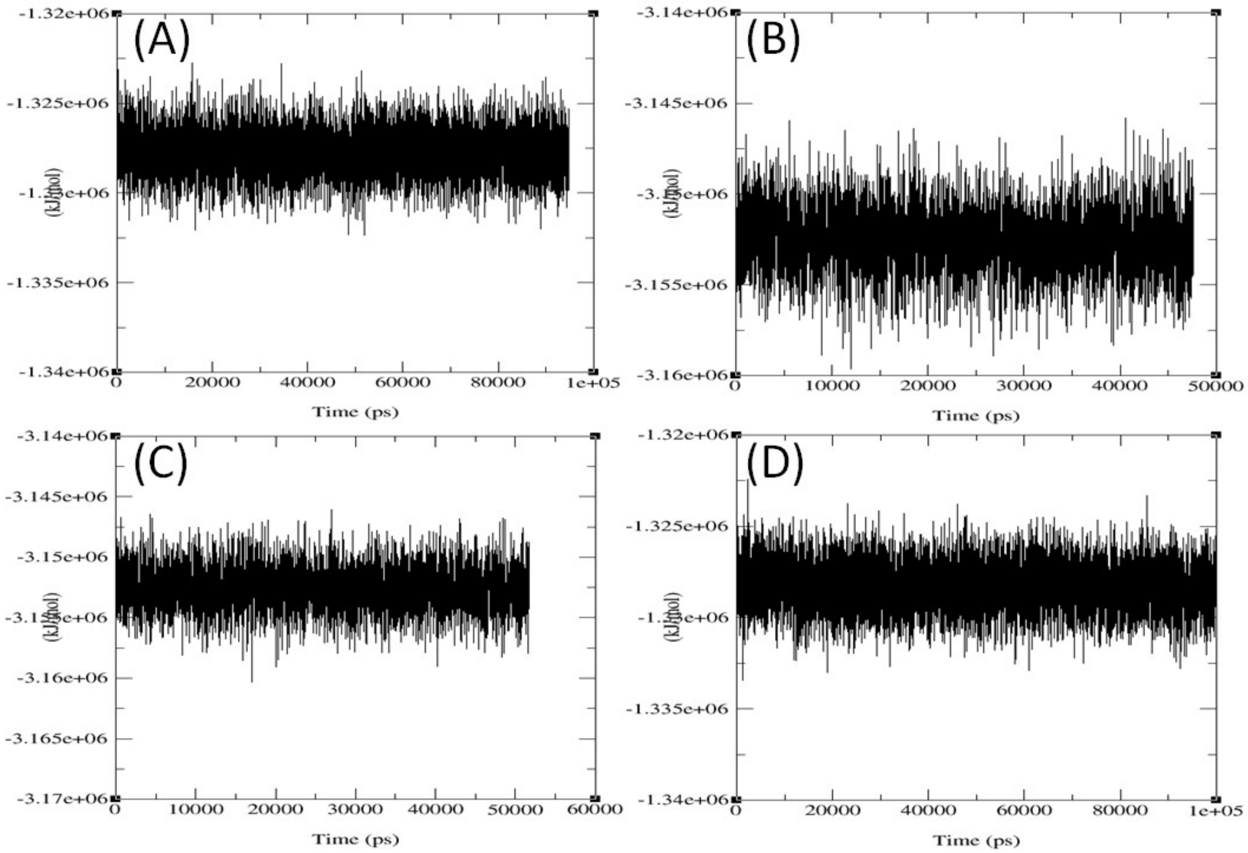

Figure S66. PE analysis for (**A**) compound **12**; (**B**) compound **14**; and (**C**) compound **15**; (**D**) reference drug fluconazole during the 20 ns MD simulation.

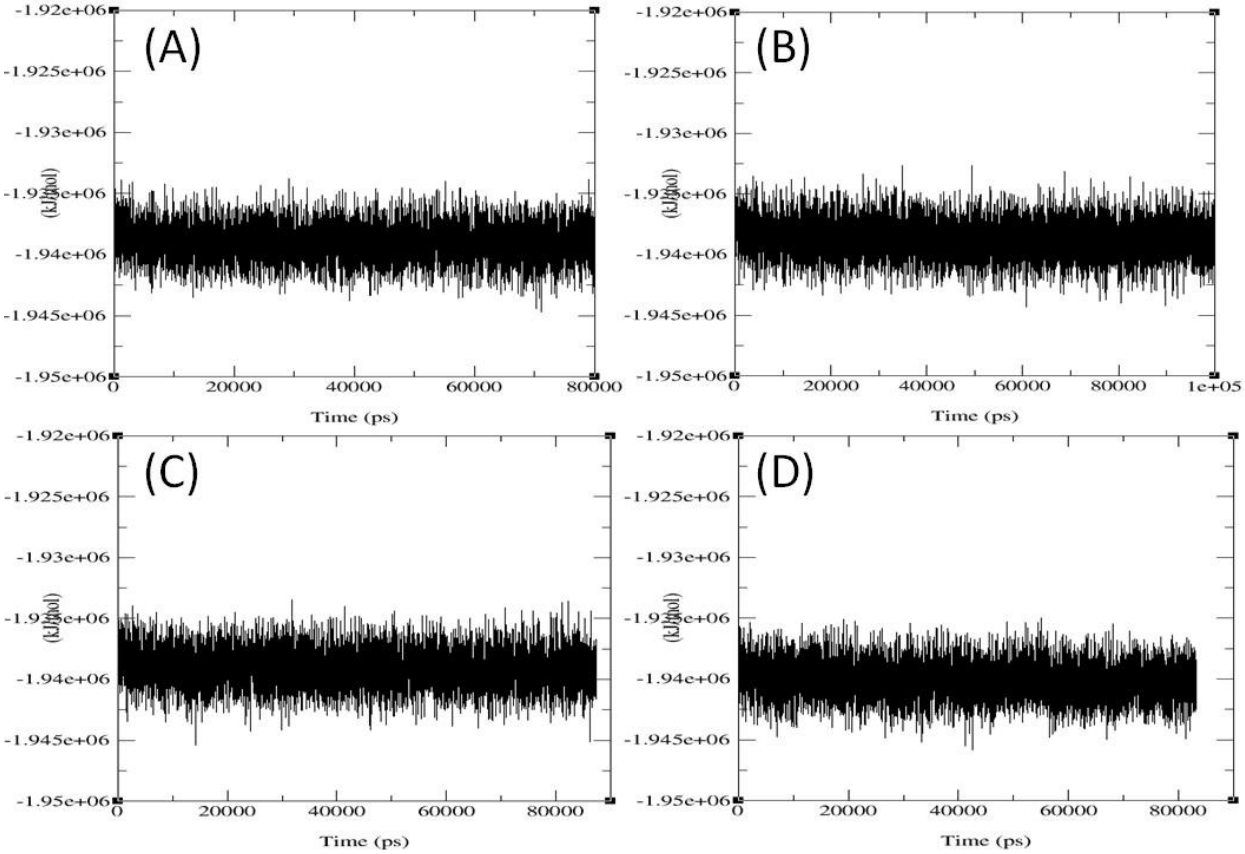

Figure S67. PE analysis for (**A**) compound **13**; (**B**) compound **16**; and (**C**) compound **17**; (**D**) reference drug gemcitabine during the 20 ns MD simulation.

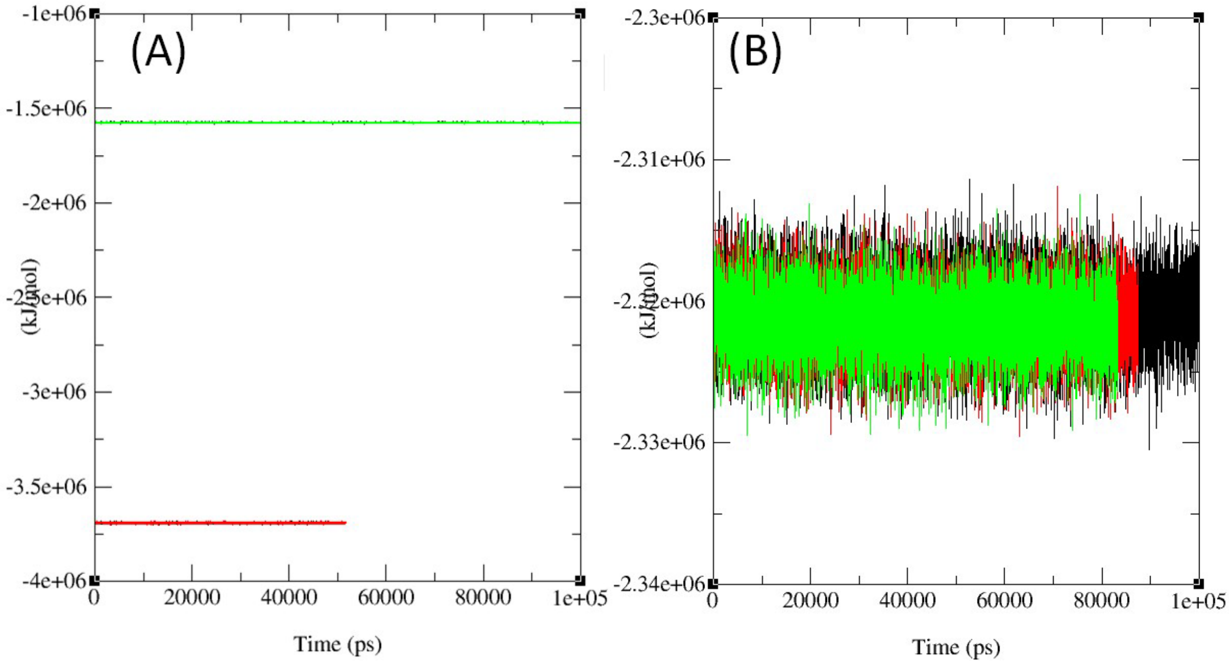

Figure S68. Coulomb-(SR) analysis of (**A**) compounds **12**, **14**, **15**, reference drug fluconazole; and (**B**) compounds **13**, **16**, **17**, and reference drug gemcitabine.

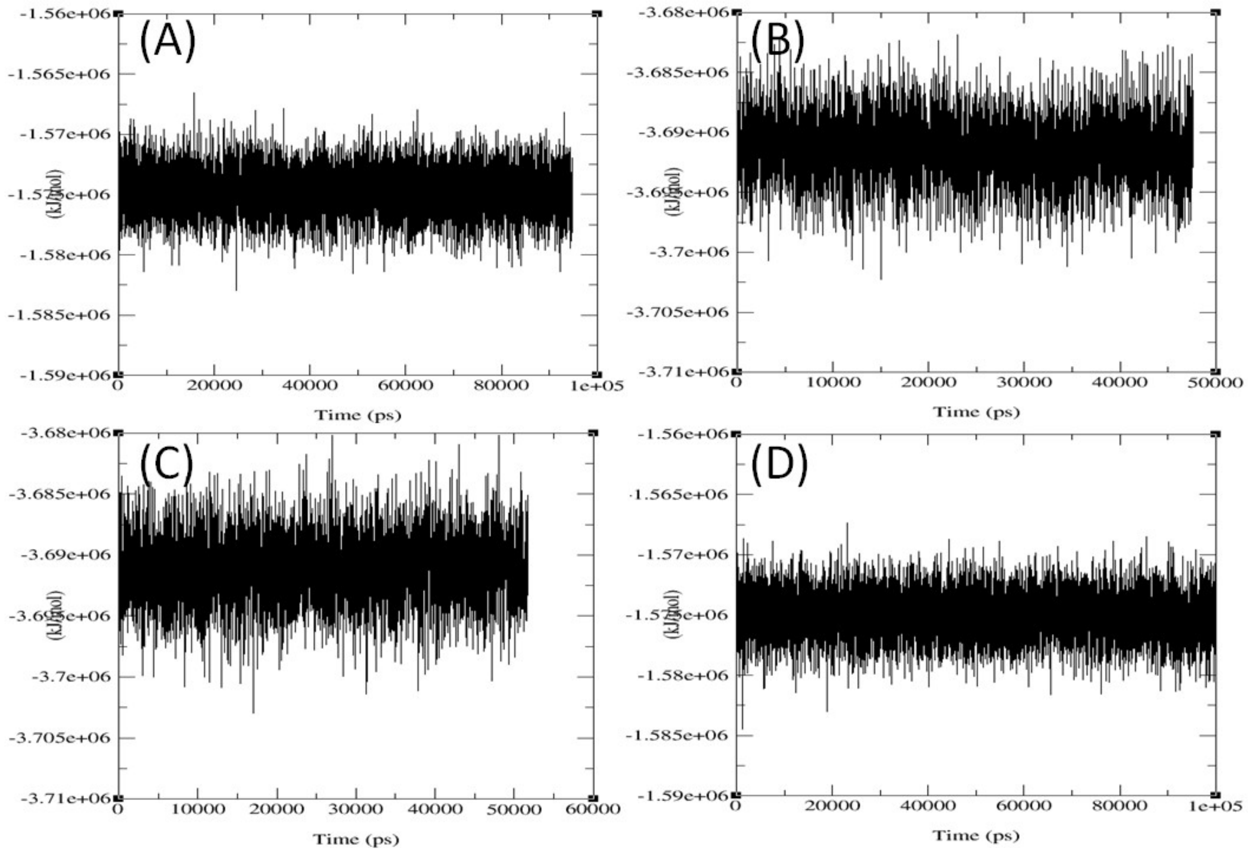

Figure S69. Coulomb-(SR) analysis for (**A**) compound **12**; (**B**) compound **14**; and (**C**) compound **15**; (**D**) reference drug fluconazole during the 20 ns MD simulation.

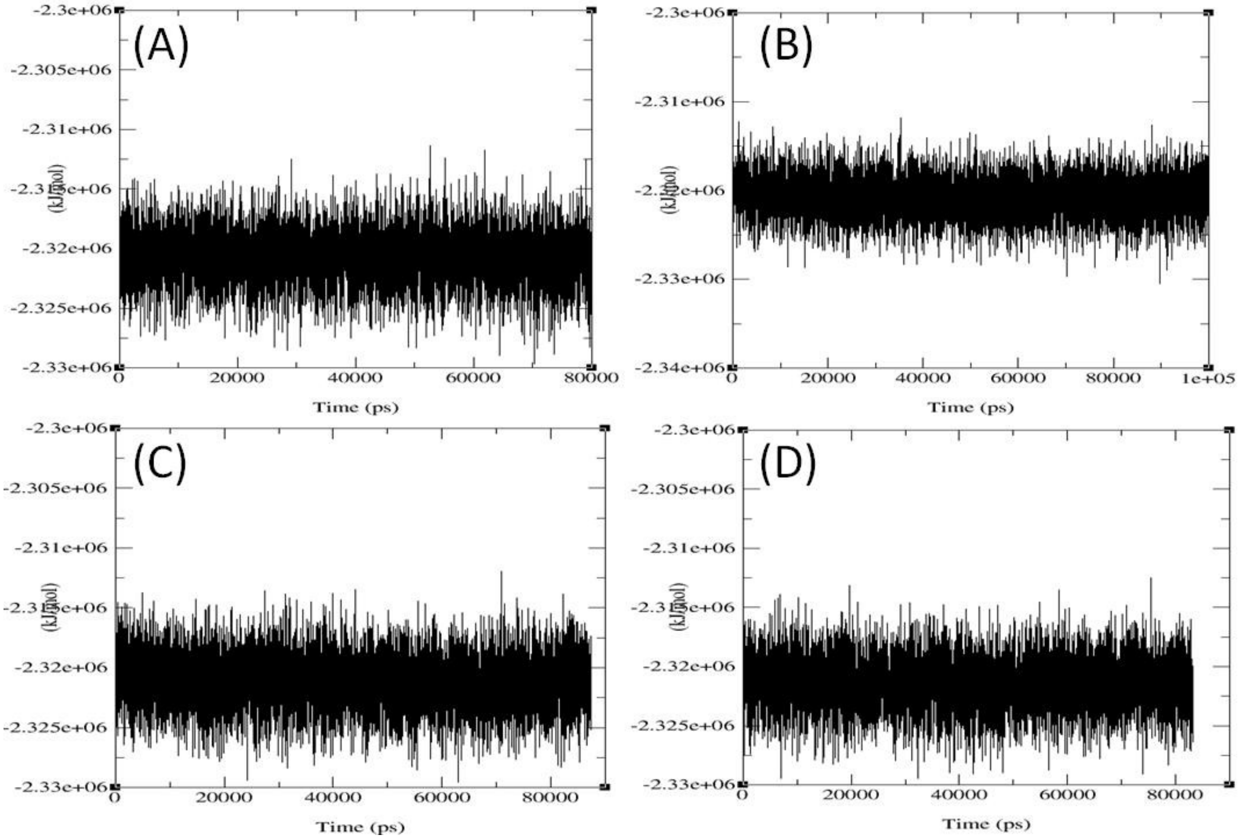

Figure S70. Coulomb-(SR) analysis for (**A**) compound **13**; (**B**) compound **16**; and (**C**) compound **17**; (**D**) reference drug gemcitabine during the 20 ns MD simulation.

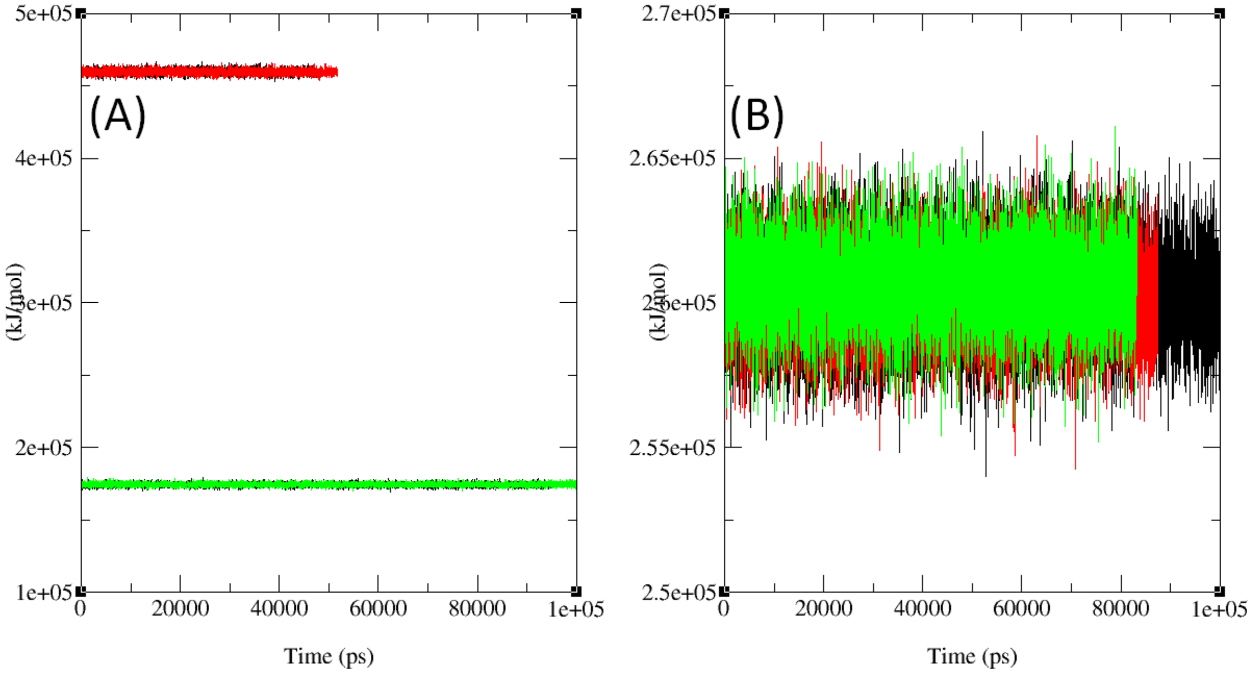

Figure S71. LJ-SR analysis of (**A**) compounds **12**, **14**, **15**, reference drug fluconazole; and (**B**) compounds **13**, **16**, **17**, and reference drug gemcitabine.

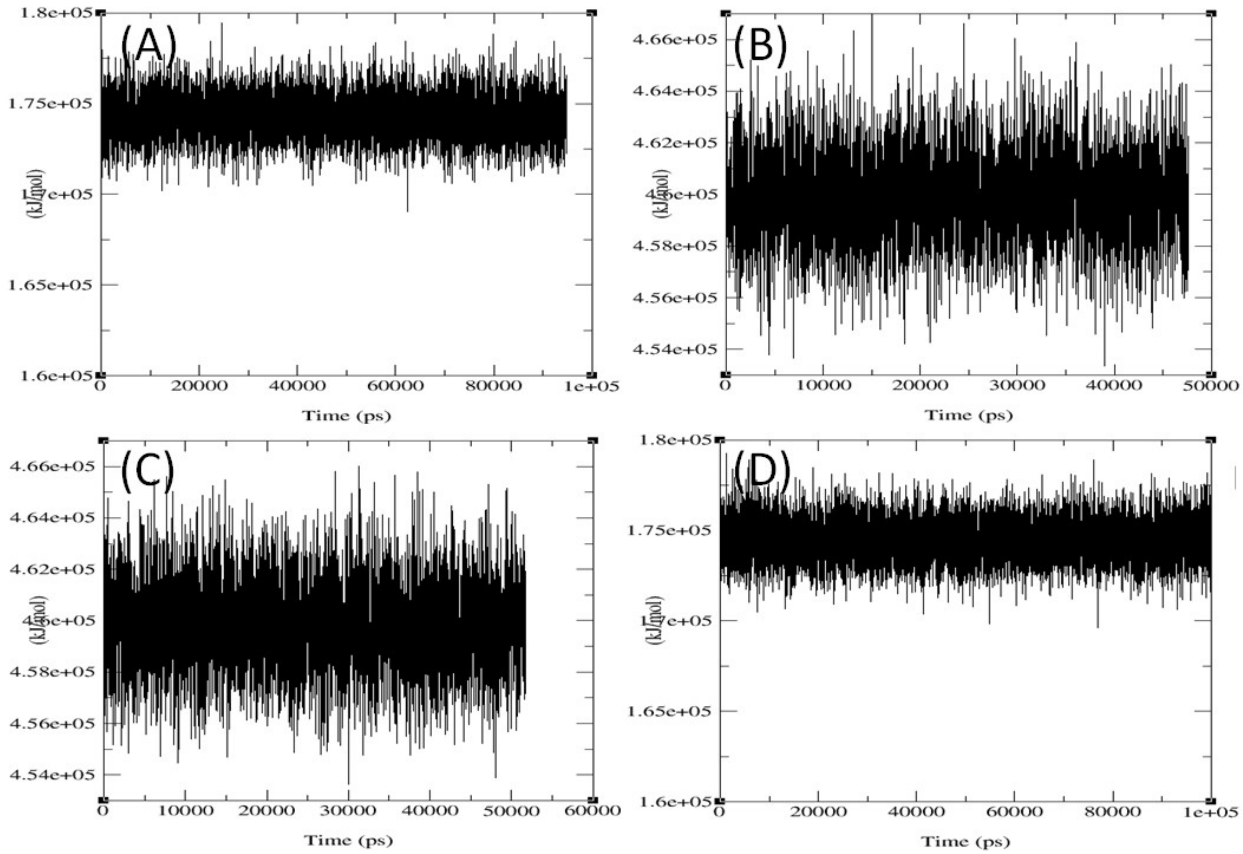

Figure S72. LJ-SR analysis for (**A**) compound **12**; (**B**) compound **14**; and (**C**) compound **15**; (**D**) reference drug fluconazole during the 20 ns MD simulation.

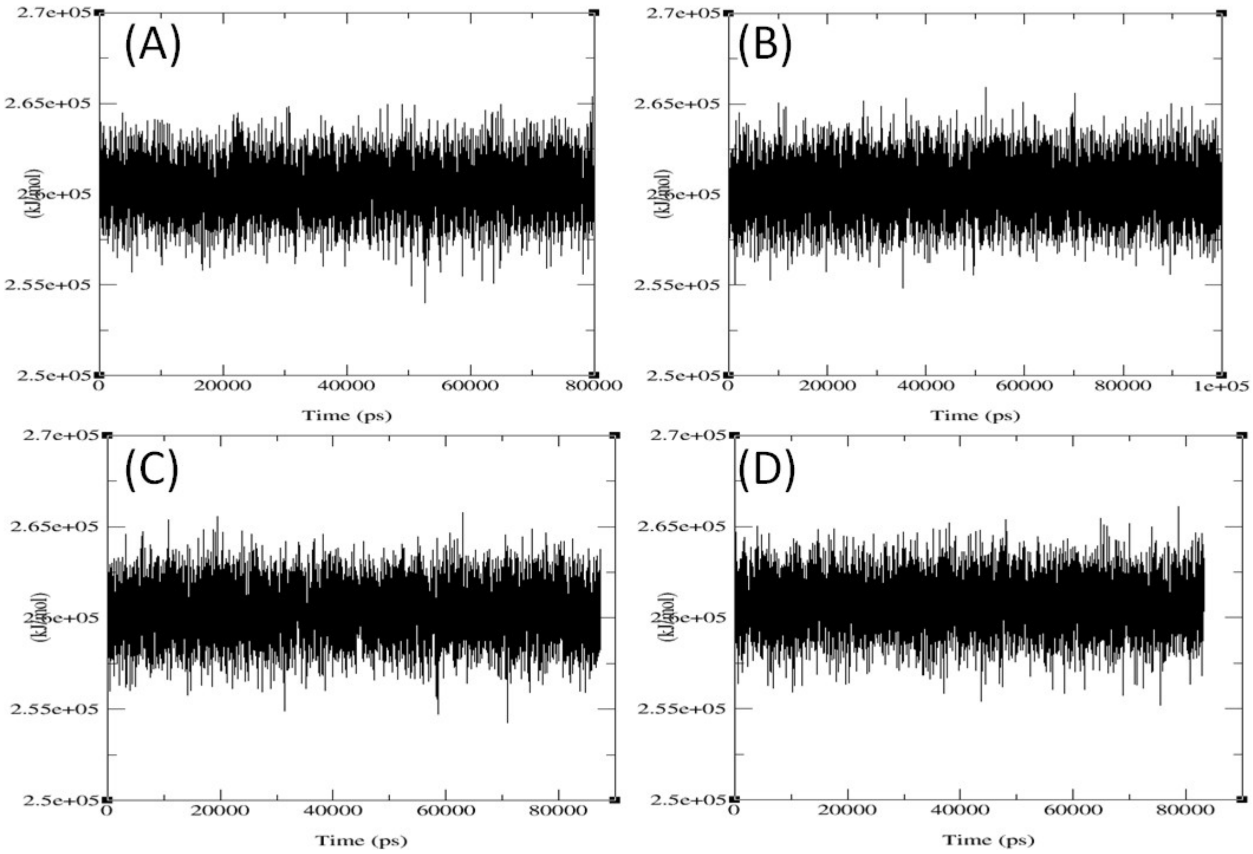

Figure S73. LJ-SR analysis for (**A**) compound **13**; (**B**) compound **16**; and (**C**) compound **17**; (**D**) reference drug gemcitabine during the 20 ns MD simulation.
